# Rapid and Direct Photocatalytic C(sp^3^)−H Acylation and Arylation in Flow

**DOI:** 10.1002/anie.202108987

**Published:** 2021-08-20

**Authors:** Daniele Mazzarella, Antonio Pulcinella, Loïc Bovy, Rémy Broersma, Timothy Noël

**Affiliations:** ^1^ Flow Chemistry Group Van't Hoff Institute for Molecular Sciences (HIMS) University of Amsterdam Science Park 904 1098 XH Amsterdam The Netherlands; ^2^ Signify Research High Tech Campus 7 5656 AE Eindhoven The Netherlands

**Keywords:** acylation, arylation, flow chemistry, hydrogen atom transfer, TBADT

## Abstract

Herein, we report a photocatalytic procedure that enables the acylation/arylation of unfunctionalized alkyl derivatives in flow. The method exploits the ability of the decatungstate anion to act as a hydrogen atom abstractor and produce nucleophilic carbon‐centered radicals that are intercepted by a nickel catalyst to ultimately forge C(sp^3^)−C(sp^2^) bonds. Owing to the intensified conditions in flow, the reaction time can be reduced from 12–48 hours to only 5–15 minutes. Finally, kinetic measurements highlight how the intensified conditions do not change the reaction mechanism but reliably speed up the overall process.

## Graphical abstract

Need for speed: An efficient and fast photocatalytic procedure that enables the acylation/arylation of unfunctionalized alkyl derivatives in flow has been realized by combining decatungstate photocatalysis and nickel catalysis. The methodology shows great functional group tolerance and high regioselectivity, and can be used for the late‐stage functionalization of various biologically‐relevant molecules.

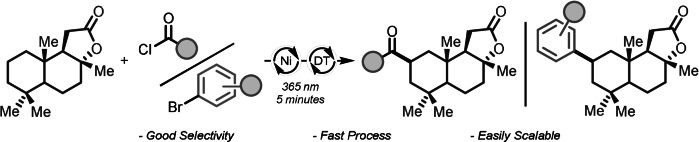

The advent of photoredox catalysis^[1]^ has not only renewed the interest of the synthetic community into radical chemistry,^[2]^ but also stimulated the development of kindred branches such as metallaphotoredox catalysis.^[3]^ In this field, the radical, generated by photocatalytic means, is intercepted by a transition metal catalyst and enables the construction of carbon‐carbon and carbon–heteroatom bonds. While several examples take advantage of the combination of functionalized substrates and matched redox photocatalytic properties (via single electron transfer, SET),^[4]^ fewer examples have been reported where strong C(sp^3^)−H bonds are activated via a photocatalytic hydrogen atom transfer (HAT).[^5^, ^6^, ^7^]

The HAT photocatalyst can, upon photo‐excitation, directly cleave C(sp^3^)−H bonds^[6]^ or generate species that act as HAT reagents,^[7]^ such as amine radical cations, thiyl radicals or halogen radicals, yielding subsequently the targeted carbon‐centered radicals. By tuning the electronic and steric properties of the photocatalyst, as well as of the substrate, high regioselectivity can be achieved even in complex, drug‐like structures (Figure 1 a). For example, the groups of Martin^[6e]^ and MacMillan[^6b^, ^6d^] disclosed two elegant approaches for the direct arylation of C(sp^3^)−H bonds. In both cases, either benzophenone or decatungstate anion (DT; [W_10_O_32_]^4−^) were used to activate very strong alkyl bonds, including activated and non‐activated C(sp^3^)−H bonds (Figure 1 b). Despite these efforts, both methods are still plagued by the requirement of prolonged reaction times, generally 12 to 48 hours, as the C−H abstraction is usually the rate determining step (RDS), leading to difficult‐to‐scale reaction conditions.^[8]^

**Figure 1:**
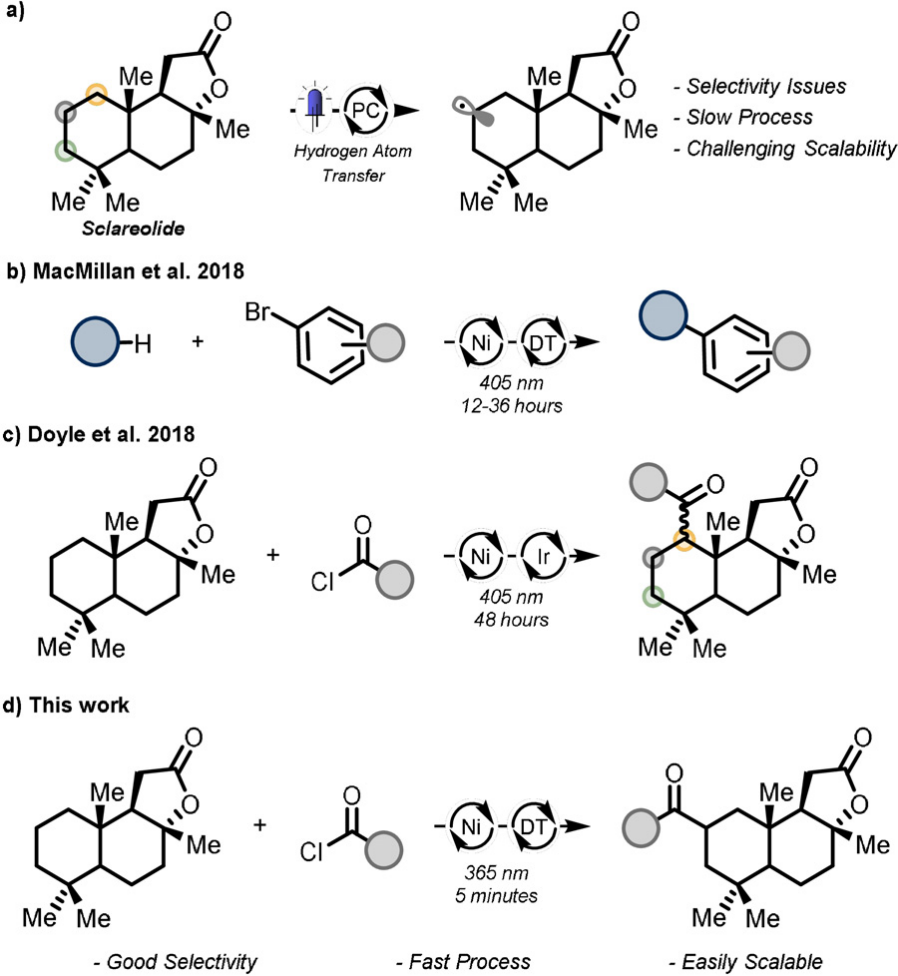
a) Photocatalytic HAT enables the conversion of C−H bonds in complex biologically active molecules; b) Use of TBADT to promote the direct arylation of C(sp^3^)−H bonds; c) Acylation of C(sp^3^)−H bonds through generation of chlorine radical; d) Proposed approach to realize a regioselective C(sp^3^)−H bond acylation by merging TBADT photo‐ and nickel catalysis.

Motivated by our recent interest into developing HAT processes with DT catalysis,^[9]^ we wondered whether we could expand the breadth of DT‐enabled photocatalytic reactions to the nickel‐catalyzed direct acylation of unfunctionalized C(sp^3^)−H bonds (Figure 1 d). In a seminal example, the group of Doyle^[7e]^ reported an acylation procedure (Figure 1 c) using an iridium‐based photocatalyst and a nickel (0) complex as transition metal catalyst. Crucial for the C(sp^3^)−H bond cleavage is the SET of the Ni^II^ complex arising after oxidative addition, followed by photolysis of the Ni^III^−Cl bond to yield a Cl radical which activates the alkyl substrate. In our mechanistic scenario, because the bulky DT anion would be responsible of activating the alkyl derivative, different and higher selectivities can be expected for the targeted C(sp^3^)−H acylation,^[10]^ in contrast to the rather unselective activation enabled by chlorine radicals (Scheme 1 c).[^11^, ^12^] Moreover, to face the drawback of extended reaction times, we speculated that the use of a continuous‐flow environment in combination with high photon flux light sources, would provide an intense and uniform irradiation over the entire reaction mixture. This would substantially accelerate the overall reaction and grant scalable reaction conditions.[^13^, ^14^, ^15^]

**Scheme 1:**
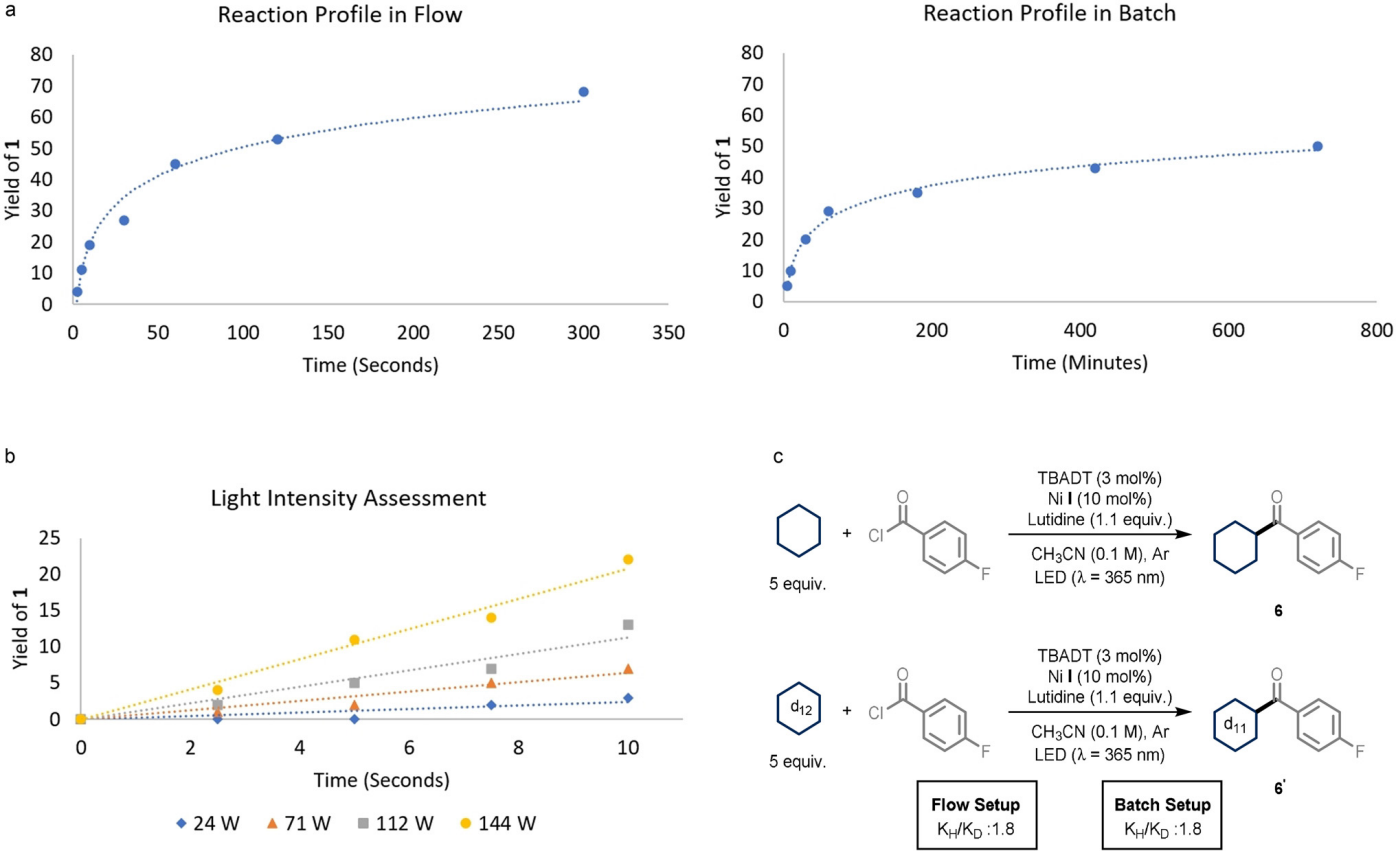
a) Reaction profile of the acylation coupling reaction between 4‐methoxy benzoyl chloride and cyclohexane in flow (left) and batch (right). Note, the difference in time scale (*x*‐axis). b) Light intensity assessment performed under microfluidic conditions. c) Kinetic isotope experiments performed on the reaction involving 4‐fluoro benzoyl chloride and cyclohexane or cyclohexane‐d_12_ in flow and batch.

We started our investigation exploring the coupling of cyclohexane with 4‐methoxy benzoyl chloride in acetonitrile in the presence of TBADT as a HAT photocatalyst, Ni **I** as the transition metal catalyst and 2,6‐lutidine as a homogeneous base to ensure removal of the ensuing hydrochloric acid (Table 1). The solution was introduced in a continuous‐flow microreactor (ID=0.76 mm; 5 mL) irradiated with UV‐A light (Chip‐on‐board LED, λ=365 nm; 144 W optical power) (See Supporting Information for technical details).

**Table 1:** Optimization of reaction conditions.^[a]^ 
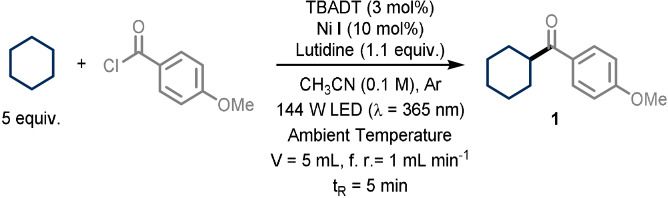

Entry	Variation from conditions	Yield [%]^[b]^
**1**	**None**	**68 (65)**
2	TBADT (1 mol %)	50
3	Ni **I** (5 mol %)	53
4	Ni **II**	39
5^[c]^	Ni **III**	28
6	36 W	52
7	DBU or Pyridine	–
8	2.5 equiv of cyclohexane	41
9	10 equiv of cyclohexane	70
10^[d]^	Batch conditions	50
11	Absence of TBADT, Ni or Light	–
12	Scale up, 5 mmol	55

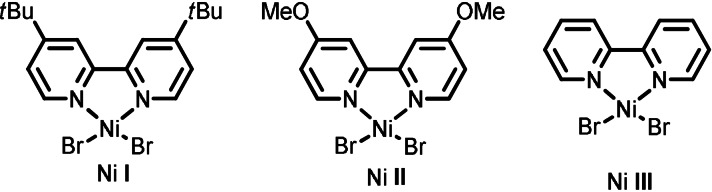		

[a] Cyclohexane (5 equiv), 4‐methoxy benzoyl chloride (0.5 mmol), in CH_3_CN (5 mL). [b] Yields determined by ^1^H NMR spectroscopy using trichloroethylene as external standard, yield of the isolated product is reported in brackets. [c] The reaction mixture is heterogeneous, therefore it was conducted under batch conditions, please see the Supporting Information for further information. [d] The reaction mixture was irradiated for 12 hours, please see the Supporting Information for further information.

After evaluation of potential reaction conditions (see Supporting Information), we found that the target product **1** could be obtained in good yields when employing 3 mol % of TBADT, 10 mol % of the nickel catalyst **I** and 5 equivalents of cyclohexane, requiring only 5 minutes residence/reaction time (Entry 1). Reducing the catalyst loading of either TBADT (Entry 2) or Ni **I** (Entry 3) afforded product **1** in reduced yields. Moreover, the use of a different nickel complex, such as Ni **II** (Entry 4) or Ni **III** (Entry 5), also proved detrimental in terms of efficiency. Reducing the light intensity resulted in lower product yields (Entry 6). Other nitrogen‐based bases, such as 1,8‐diazabicyclo(5.4.0)undec‐7‐ene (DBU) or pyridine, completely shut down the reactivity (Entry 7). The reaction can be performed using only 2.5 equivalents of the alkyl partner (Entry 8), albeit with a reduced yield, while the use of 10 equivalents (Entry 9) did not produce a significant change in terms of efficiency. Notably, when running the very same transformation under batch conditions, we found out that after 5 minutes only traces of the product were formed. To reach full conversion in batch, the reaction time had to be extended to 12 hours (Entry 10); but also in this case, only 50 % of the product was observed, demonstrating that our flow conditions not only accelerate the reaction kinetics but also enable a higher level of efficiency. Control experiments proved that light, Ni **I** and TBADT are all crucial to ensure product formation (Entry 11). Finally, the model reaction can be readily scaled to a 5 mmol scale (Entry 12). With optimal reaction conditions in hand, we investigated the generality of this photocatalytic acylation protocol (Figure 2 a). First, we combined cyclohexane with a diverse set of benzoyl chlorides (**1**–**7**). Substitution at the meta position is well tolerated and the corresponding product was isolated in good yield (**2**, 63 % yield), while substitution at the ortho position afforded the target compound with lower efficiency (**3**, 24 % yield). Benzoyl chloride (**4**, 40 % yield) and its para‐substituted derivatives bearing a phenyl (**5**, 57 % yield) or electron‐withdrawing groups (e.g. fluoro **6** and trifluoromethyl **7,** 42 % and 35 % yield, respectively) proved to be competent substrates as well. Also acyl chlorides derived from the corresponding alkyl carboxylic acids, served as suitable reaction partners yielding the corresponding ketones upon coupling with cyclohexane (**8**–**13**). For example, tertiary (**8**, 65 % yield), secondary (**9**, 43 % yield) and primary acyl chloride derivatives (**10**, 40 % yield) furnished selectively the desired products without functionalizing other C−H bonds within the molecular scaffold. To further highlight the synthetic utility of this process, the late‐stage C−H acylation using acyl chlorides derived from in nature‐occurring carboxylic acids was pursued. Hereto, the acyl chloride of stearic acid (**11**, 37 % yield), dehydrocholic acid (**12**, 38 % yield) and gibberellic acid (**13**, 34 % yield) were smoothly converted into the corresponding ketones in only 5 minutes residence time.

Next, we evaluated the scope of the H‐donors amenable to the developed acylation methodology. Our protocol enables selective modification of various five‐membered rings, including cyclopentane (**14**, 81 % yield), sulfolane (**15**, 61 % yield) and cyclopentanone (**16**, 79 % yield). Also, seven‐membered rings, such as cycloheptanone (**17**, 66 % yield, 1:1 r. r.), or fused bicyclic scaffolds (**18**, 37 % yield, 4:1 r.r.) are readily acylated under the reaction conditions. The method can also be used to convert acyclic substrates; as an example, subjecting pentane to our nickel/decatungstate cross‐coupling protocol resulted into the corresponding product (**19**, 38 % yield, 3:1 r.r.). Interestingly, biased positions in organic molecules, such as an α‐to‐O C−H bond (**20**, 52 % yield), led to selective functionalization. Bicylic structures, for example, norbornane (**21**, 51 % yield) or 7‐oxabicyclo[2.2.1]heptane (**22**, 48 % yield), could also be selectively functionalized at the secondary C−H bond, leaving untouched the more reactive tertiary position. This can be ascribed to the bulky size of the decatungstate anion which allows to discriminate different C(sp^3^)−H bonds based on steric hindrance, which sets it apart from halogen radical HAT catalysis. Furthermore, despite the presence of a transition metal catalyst, this protocol tolerates well bromide substituents (**23**, 54 % yield) or exocyclic double bonds (**24**, 40 % yield, 1:1 r.r.), which should serve as entry points for further decoration of the organic scaffold. Finally, to showcase the high selectivity and the synthetic applicability of this method, we performed the late‐stage functionalization of complex organic or natural scaffolds such as eucalyptol (**25**, 40 % yield, 3.5:1 r.r.) and sclareolide (**26**, 38 % yield). The latter examples serve as a highlight of the superior selectivity that can be obtained using TBADT as HAT photocatalyst. Indeed, in contrast to the use of halogen radicals, no erosion of the selectivity was observed by functionalization of other C−H bonds within these complex organic scaffolds.^[7e]^

After having established the acylation procedure, we wondered whether we could improve the aforementioned slow reaction kinetics in the arylative process reported by MacMillan and co‐workers.^[6d]^ After a brief optimization process to translate the protocol to flow (see Supporting Information), including the use of the homogeneous base lutidine to avoid microreactor clogging, we were able to drastically reduce the reaction times from 12–48 hours to 15–30 minutes in comparable and scalable yields (Figure 2 b). Various arenes bearing electron‐withdrawing substituents (**27**–**29**, 69–43 % yield), heteroarenes (**30**–**32**, 76–40 % yield) and electron‐donating substituents^[16]^ (**33** and **34**, 36 % and 33 % yield, respectively) all swiftly reacted to yield the corresponding products. Concerning the scope of the H‐donors, both α‐to‐O (**35**, 55 % yield), α‐to‐N (**36**, 60 % yield) and benzylic (**37**, 70 % yield) C−H bonds could be readily arylated. Similarly to the acylation procedure, norbornane proved to be a competent substrate (**38**, 65 % yield). Finally, late‐stage functionalization of sclareolide (**39**, 46 % yield) and ambroxide (**40**, 59 % yield) confirmed the synthetic utility of this process.

**Figure 2:**
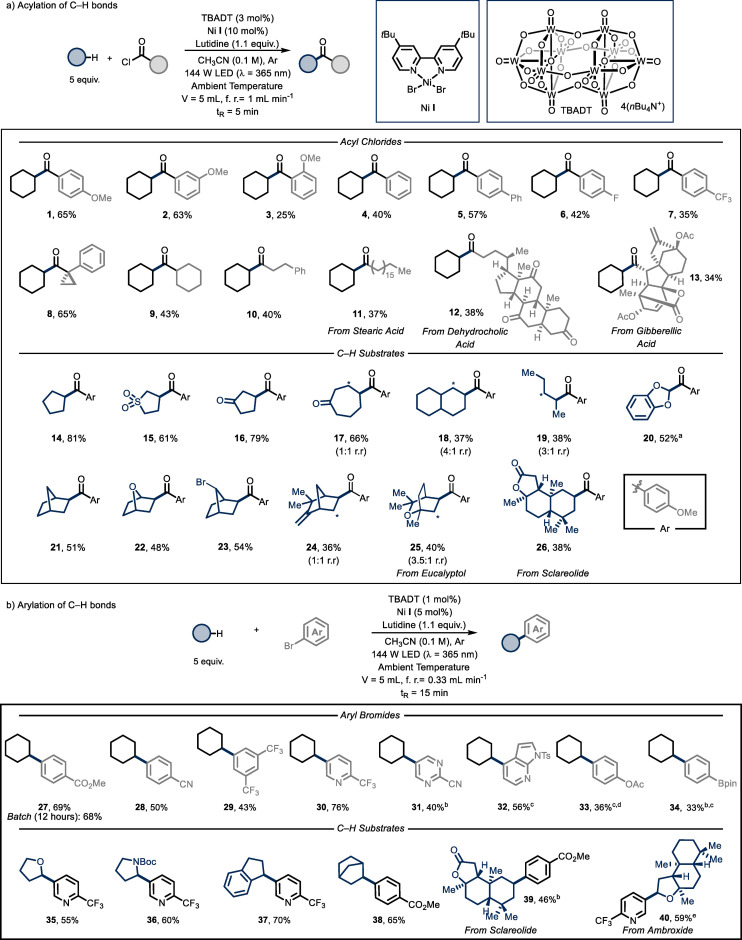
a) Survey of the acyl chlorides and C−H substrate that can participate to the C(sp^3^)−H bond acylation with TBADT catalysis; Reactions performed on a 0.5 mmol scale using 5 equivalents of C−H substrate in 5 mL of CH_3_CN. (*λ*=365 nm 500 W, reactor volume: 5 mL, flow rate: 1 mL min^−1^, *t*_r_: 5 minutes). Yields refer to isolated product.^[a]^ Flow rate: 0.33 mL min^−1^, *t*_r_: 15 minutes. b) Survey of the aryl bromides and C−H substrate that can participate to the C(sp^3^)−H bond arylation with TBADT catalysis; Reactions performed on a 0.5 mmol scale using 5 equivalents of C−H substrate in 5 mL of CH_3_CN. (*λ*=365 nm 500 W, reactor volume: 5 mL, flow rate: 0.33 mL min^−1^, *t*_r_: 15 minutes). Yields refer to isolated product.^[b]^ Flow rate: 0.17 mL min^−1^, *t*_r_: 30 minutes.^[c]^ TBADT (3 mol %), Ni **I** (10 mol %).^[d]^ Flow rate: 0.11 mL min^−1^, *t*_r_: 45 minutes.^[e]^ Concentration 0.05 M.

To understand the difference in reaction rates between the flow and batch setups and its potential effect on the developed methodology, we monitored the kinetic profiles of the two reactions (Scheme 1 a). While the process in flow is completed within merely five minutes, the protocol in batch requires at least 12 hours. Moreover, when measuring the initial rates of the model reaction in the microfluidic reactor (Scheme 1 b), we observed that the increase of the light power is accompanied with a faster rate, this further highlighting the crucial effect of the light intensity on the reaction kinetics. Finally, we also performed studies to quantify the magnitude of a possible kinetic isotope effect (KIE, Scheme 1 c).^[17]^ Interestingly, in both the flow and the batch setup, we observed a KIE of 1.8, in accordance with HAT being involved in the rate‐determining step. Taken together, these experiments also suggest that the use of a higher light intensity in our microfluidic setup increases the rate of the overall reaction by boosting the photoactivation of the C(sp^3^)−H bonds, but has no apparent influence on the reaction mechanism.

In conclusion, we have reported a practical procedure that allows the acylation and arylation of strong aliphatic bonds in flow. Thanks to the microfluidic setup, this transformation takes only 5–15 minutes and is readily scalable. Due to the mild reaction conditions, the methodology shows great functional group tolerance and high regioselectivity, and can be used for the late‐stage functionalization of various complex biologically‐relevant molecules. Kinetic studies show that the combination of a microfluidic environment and powerful light sources has influence on the overall rate of the reaction without affecting the mechanism.

## Conflict of interest

The authors declare no conflict of interest.

## Supporting information

As a service to our authors and readers, this journal provides supporting information supplied by the authors. Such materials are peer reviewed and may be re‐organized for online delivery, but are not copy‐edited or typeset. Technical support issues arising from supporting information (other than missing files) should be addressed to the authors.

Supporting Information
