# Supplementary material for: Rapid and Direct Photocatalytic C(sp3)−H Acylation and Arylation in Flow
Source: Angew Chem Int Ed Engl. 2021 Aug 20;60(39):21277–82. doi: 10.1002/anie.202108987 (PMC8518495; doi:10.1002/anie.202108987)

Angewandte Chemie
Eine Zeitschrift der Gesellschaft Deutscher Chemiker

Supporting Information

Rapid and Direct Photocatalytic C(sp^3)−H Acylation and Arylation in Flow

*Daniele Mazzarella[+], Antonio Pulcinella[+], Loïc Bovy, Rémy Broersma, and Timothy Noël**

anie_202108987_sm_miscellaneous_information.pdf

Table of Contents

1. General information S2

Reactor Design S3

2. Optimization of the reaction conditions S5

2.1 Optimization of the Acylation Protocol in Flow S5

2.2 Translation of the Arylation Protocol in Flow S7

3. Experimental procedure S8

3.1 General Procedure A, Acylation S8

3.2 General Procedure B, Arylation S17

4. Limitation of the scope S22

5. Kinetic Studies S23

5.1 Reaction Profile S23

5.2 Light Intensity Assessment S25

5.3 Kinetic Isotopic Effect (KIE) S26

6. NMR Spectra S28

1. General Information

^{1}H (400 and 300 MHz), ^{11}B (128 MHz), ^{13}C (101 and 128 MHz) and ^{19}F (376 MHz) spectra were recorded at ambient temperature using Bruker AV 300-I, AV 400 and AV 500-NEO. ^{1}H NMR spectra are reported in parts per million (ppm) downfield relative to $CDCl_3$ (7.26 ppm) and all ^{13}C NMR spectra are reported in ppm relative to $CDCl_3$ (77.16 ppm) unless stated otherwise. The multiplicities of signals are designated by the following abbreviations: s (singlet), d (doublet), t (triplet), q (quartet), m (multiplet), dd (doublet of doublets), dt (doublet of triplets), td (triplet of doublets), ddd (doublet of doublet of doublets). Coupling constants (J) are reported in hertz (Hz). NMR data was processed using the MestReNova 14 software package. High resolution mass spectra (HRMS) were collected on an AccuTOF LC, JMS-T100LP Mass spectrometer (JEOL, Japan) or on an AccuTOF GC v 4g, JMS-T100GCV Mass spectrometer (JEOL, Japan). Disposable syringes were purchased from Laboratory Glass Specialist. Syringe pumps were purchased from Chemix Inc. model Fusion 200 Touch. Product isolation was performed manually, using silica (P60, SILICYCLE). TLC analysis was performed using Silica on aluminum foils TLC plates (F254, SILICYCLE) with visualization under ultraviolet light (254 nm and 365 nm) or appropriate TLC staining (Cerium Ammonium Molybdate). Organic solutions were concentrated under reduced pressure on a Büchi rotary evaporator (in vacuo at 40 ºC, ~5 mbar).

Materials All reagents and solvents were used as received without further purification. Reagents and solvents were bought from Sigma Aldrich, TCI and Fluorochem. Technical solvents were bought from VWR International and used as received. The TBADT[1] and nickel[2] catalysts were prepared according to reported procedures. The majority of acyl chlorides, aryl bromides and C–H substrates are commercially available. When not available, acyl chlorides have been synthetized from the corresponding carboxylic acids following a reported procedure and used directly without purification.[3] The synthesis of cedrol acetate,[4] gibberellic acid acetate[5] and 4-bromo-1-tosyl-1*H*-pyrrolo[2,3-*b*]pyridine[6] was performed according to literature procedures.

Determination of Regioisomeric and Diastereomeric Ratio The regioisomeric and diastereomeric ratios were determined by ^{1}H NMR analysis of the crude reaction mixture through integration of diagnostic signals. For cases where the integration of diagnostic signals is not possible, the ratio is calculated after the purification step.

[1] T. Wan, L. Capaldo, G. Laudadio, A. V. Nyuchev, J. A. Rincón, P. García-Losada, C. Mateos, M. O. Frederick, M. Nuño, T. Noël *Angew. Chem. Int. Ed.* **2021**, *10.1002/anie.202104682*.
[2] I. B. Perry, T. F. Brewer, P. J. Sarver, D. M. Schultz, D. A. DiRocco, D. W. C. MacMillan *Nature* **2018**, *560*, 70–75.
[3] A. C. Sun, E. J. McClain, J. W. Beatty, C. R. J. Stephenson *Org. Lett.* **2018**, *20*, 3487–3290.
[4] T. Barber, S. P. Argent, L. T. Ball *ACS Catal.* **2020**, *10*, 5454–5461.
[5] A. Fawcett, J. Pradeilles, Y. Wang, T. Mutsuga, E. L. Myers, V. K. Aggarwal *Science* **2017**, *357*, 283–286.
[6] R. Y. Nimje, D. Vytla, P. Kuppusamy, R. Velayuthaperumal, L. B. Jarugu, C. A. Reddy, N. K. Chikkananjaiah, R. A. Rampulla, C. L. Cavallaro, J. Li, A. Mathur, A. Gupta, A. Roy *J. Org. Chem.* **2020**, *85*, 11519–11530.

Reactor Design

- Vapourtec System

For the final optimization experiments and the evaluation of the scope, a Vapourtec device with a UV-150 photochemical reactor was used, equipped with 60 W 365 nm LED.

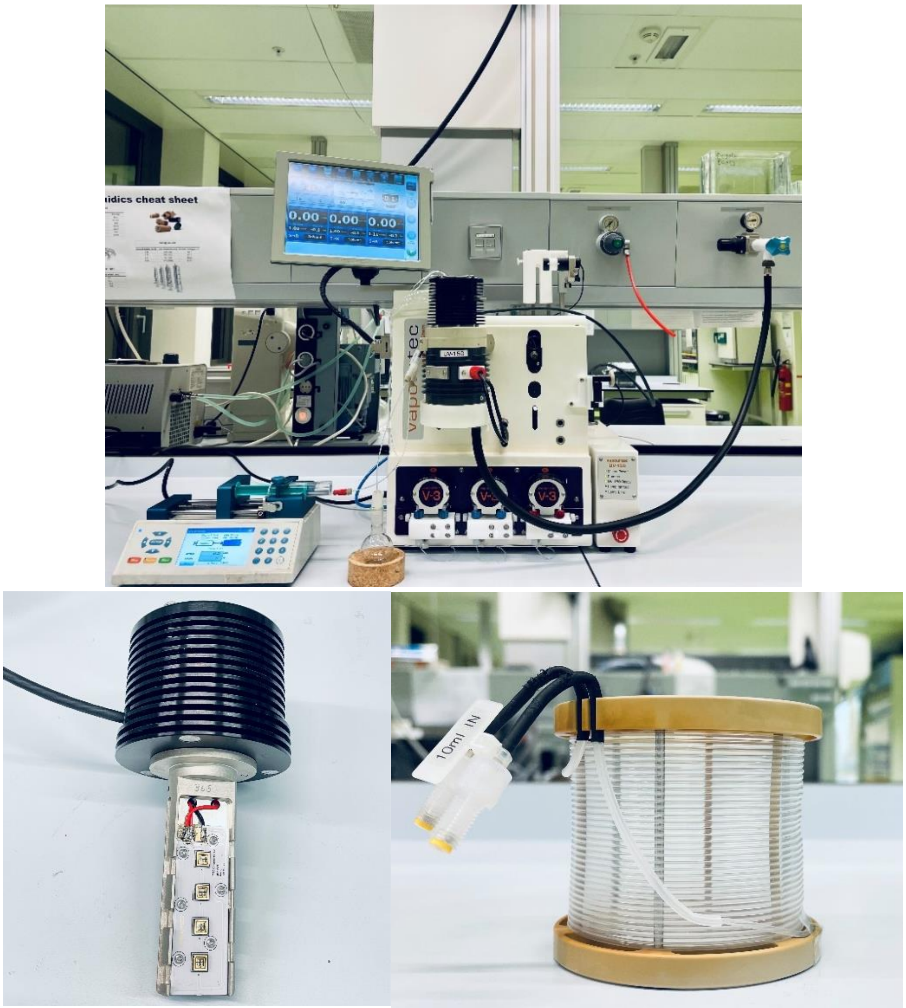

Figure S1: Overview and details of the Vapourtec system (flow setup 2): in particular, LEDs and the PFA coil (internal diameter 1.3 mm, external diameter 1.6 mm) are shown.

- Signify Eagle Reactor

The reactor system for photo(chemical) processing of the fluid consists of a central cylindrical reactor support, holding an interchangeable flexible transparent or translucent reactor tube in a helical shape, an ensemble of 6 light source modules surrounding the reactor tube, a head cap assembly, and a base support assembly. The head cap assembly contains blowers to cool the interior of the reactor system, in particular the irradiance emitting side of the light source module and the reactor tube containing the reaction liquid, to reduce undesired thermal side-reactions. The air flow provided by the blowers can be controlled and adjusted if desired.

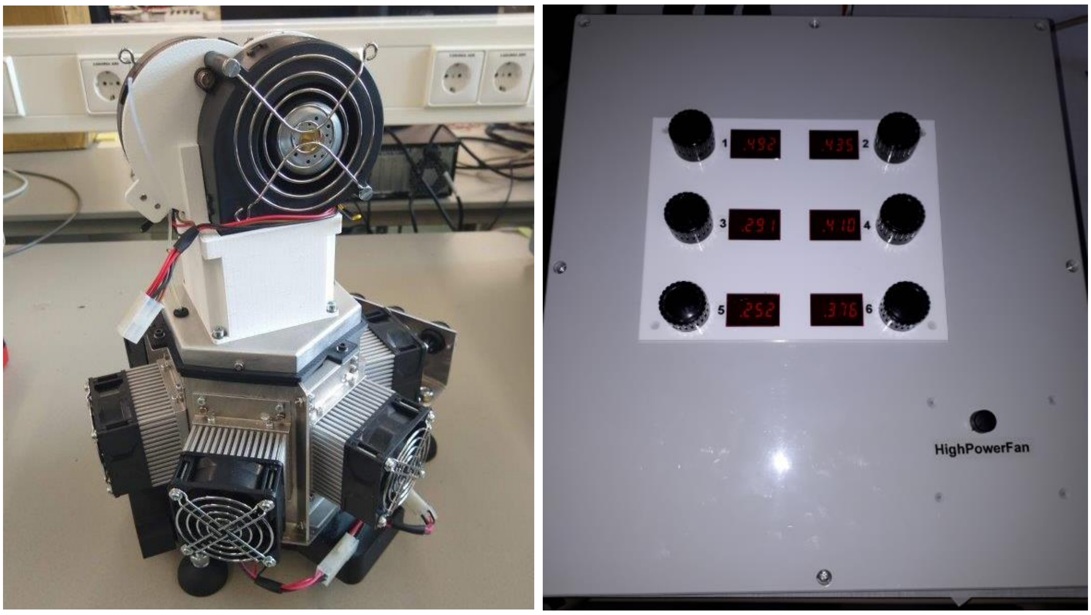

Figure S2: Signify's photo reactor system with 6 LED light source modules (left); control unit for the 6 light source modules and the high-power fan (right).

The LED light source modules in this case have a UVA 365 nm chip-on-board (COB) protected by a quartz exit window and are attached to a heat dissipation element containing cooling fins and a fan for an optimized irradiance efficiency. The UVA light source modules can be driven up to a maximum current of 2 amperes (A), and each module is individually controllable and dimmable down to 1%. The total optical output of the 6 light source modules combined at the maximum current condition is 144 W. Due to thermal effects on the spectral emission of LEDs, the actual peak wavelength at the drive current of 2 A for these UVA LED's is 371 nm, whereas at drive currents below 0.5 A, the peak wavelength is 367 nm. The total electrical power consumed at this maximum drive condition is 504W. The light source modules can be freely interchanged and controlled with modules having a different irradiance wavelength or color.

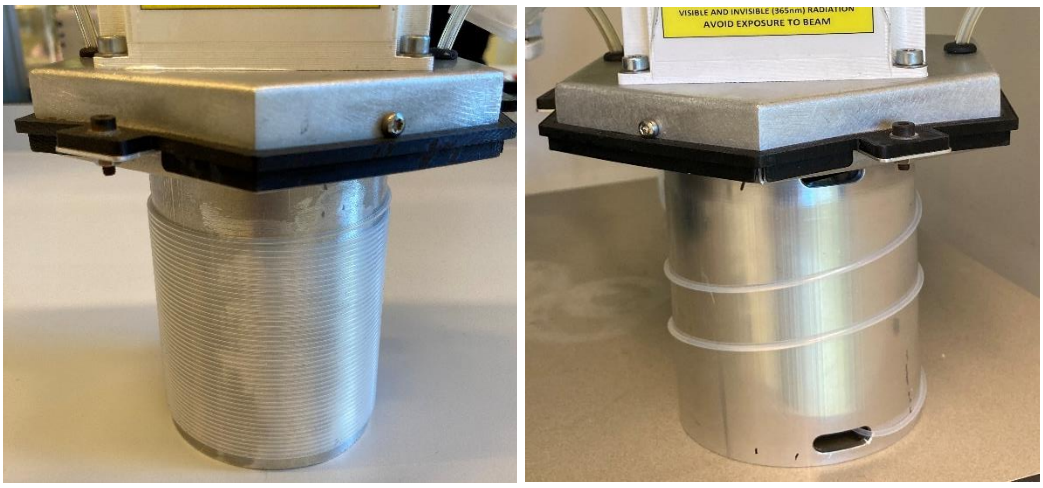

Figure S3: PFA coils (internal diameter 0.76 mm, 5 mL on the left, 0.23 mL on the right) are shown.

2. Optimization

2.1. Optimization of the Acylation Protocol in Flow

Table S1. Optimization of the catalysts loadings.

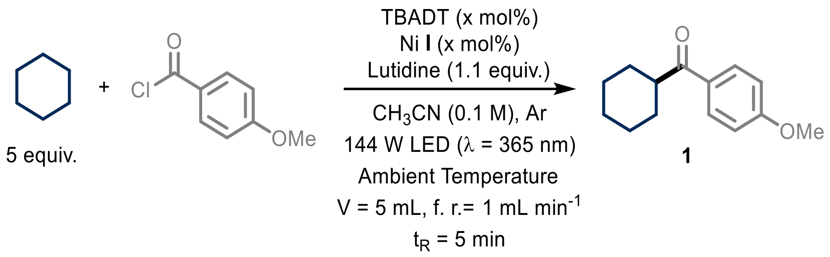

Entry	Catalysts Loading	Yield
1	TBADT (1 mol%), Ni **I** (5 mol%)	42%
2	TBADT (3 mol%), Ni **I** (5 mol%)	53%
3	TBADT (1 mol%), Ni **I** (10 mol%)	50%
4	TBADT (2 mol%), Ni **I** (10 mol%)	62%
5	TBADT (3 mol%), Ni **I** (10 mol%)	68%

Table S2. Optimization of the base.

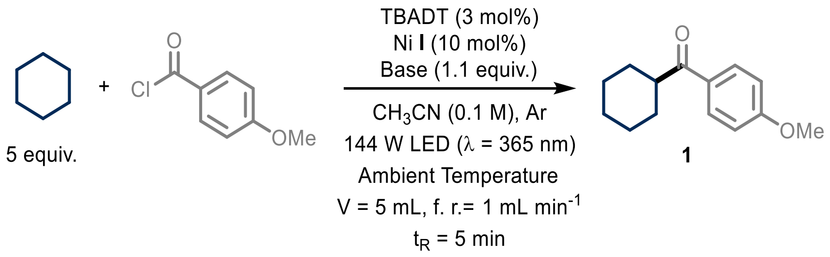

Entry	Base	Yield
1	Lutidine	68%
2	Pyridine	-
3	DBU	-
4	TMG	-
5	2,6-Pyridinedicarbonitrile	-

Table S3. Optimization of the nickel complex.

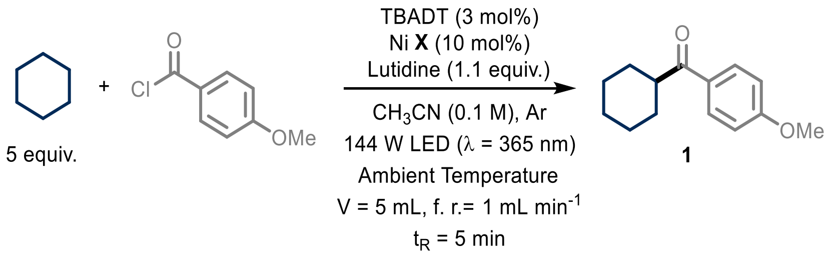

Entry	Complex	Yield
1	Ni I	68%
2	Ni **II**	-
3	Ni **III**	-

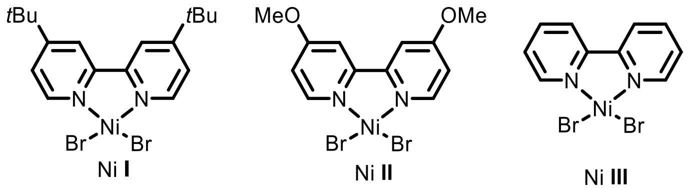

Table S4. Optimization of the residence time.

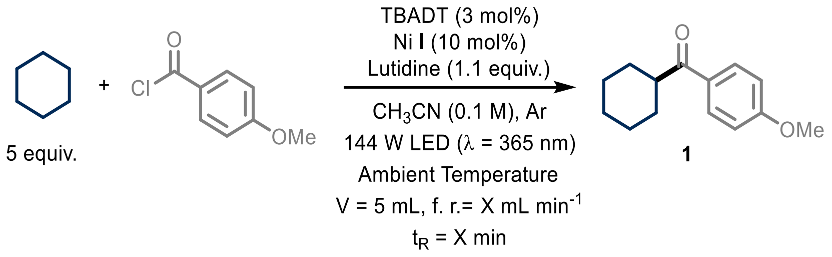

Entry	Residence Time	Yield
1	5 minutes	68%
2	1 minute	41%
3	10 minutes	58%

Table S5. Optimization of the equivalents of cyclohexane.

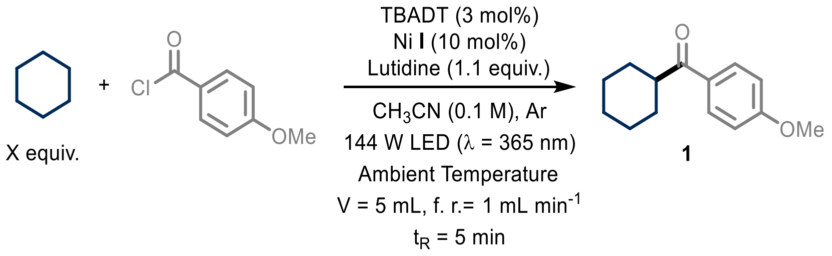

Entry	Equivalents of cyclohexane	Yield
1	5	68%
2	2.5	41%
3	10	70%

Table S6. Control experiments.

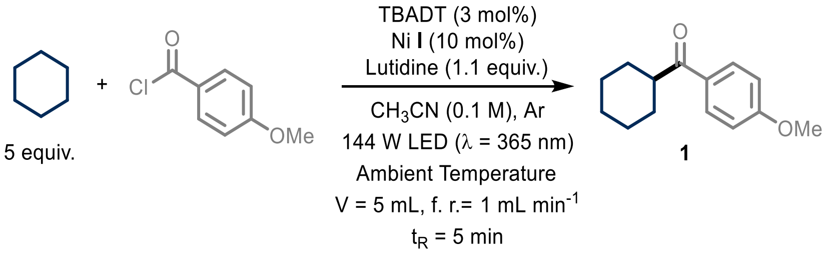

Entry	Control Experiment	Yield
1	No Light	-
2	No TBADT	-
3	No Ni **I**	-

2.2. Translation of the Arylation Protocol in Flow

Table S7. Translation of the arylation process in flow.

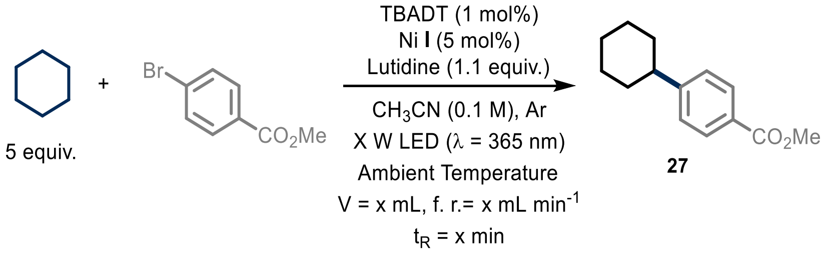

Entry	Reactor and Light Intensity	Residence Time (min)	Reactor Volume	Yield
1	Vapourtec 16 W	60	10 mL	45%
2	Vapourtec 16 W	120	10 mL	55%
3	Signify Eagle 144 W	15	5 mL	69%
4	Signify Eagle 144 W	5	5 mL	45%

Table S8. Optimization of the base.

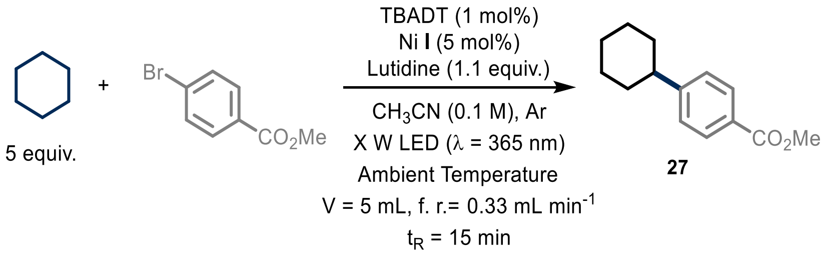

Entry	Base	Yield
1	DBU	-
2	2,6-Pyridinedicarbonitrile	-
3	2,6-Di-tert-butylpyridine	12%
4	Pyridine	-

3. Experimental Procedures

3.1. General Procedure A (Photochemical Acylation of C–H Bonds in Flow)

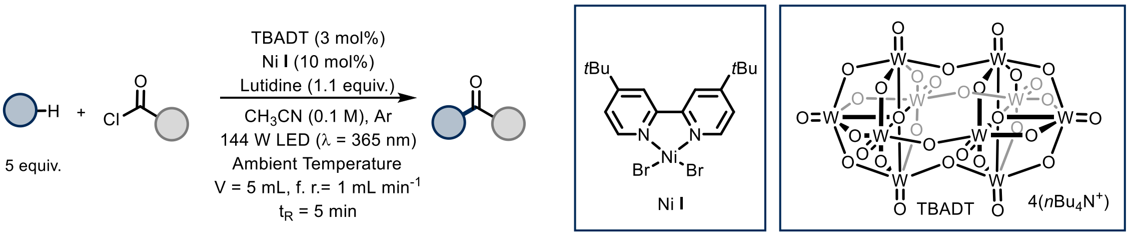

To a flame-dried argon-purged screw-capped vial, fitted with a rubber septum, charged with the nickel complex **I** (24.4 mg, 10 mol%), TBADT (49.8 mg, 3 mol%) and the acyl chloride (0.5 mmol, 1 equiv.) in CH_3CN (2.5 mL, 0.20 M), the alkane derivative was added (5 equiv.). The resulting solution was sonicated for 1 minute to ensure full homogeneity. A second flame-dried argon-purged screw-capped vial fitted with a rubber septum was charged with 2,6-dimethylpyridine (64 µL, 0.55 mmol, 1.1 equiv.) and CH_3CN (2.5 mL, 0.22 M). These two solutions were taken separately with a 6 mL syringe (12.4 mm of diameter), positioned on a syringe pump and connected to a T-mixer. The latter was connected to the Signify reactor , set at 144 W. The two solutions were pumped, unless specified somewhere else, with a total 1.00 ml/min flow rate and collected at the end of the reactor. The solvent of the resulting reaction mixture was removed under reduced pressure and purification by flash column chromatography on silica gel gave the corresponding products in the stated yield.

- Characterization Data of Products

Cyclohexyl(4-methoxyphenyl)methanone (1)

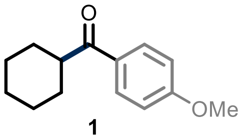

1

Prepared according to general procedure A, using cyclohexane (270 µL, 2.5 mmol, 5 equiv.) and 4-methoxybenzoyl chloride (68 µL, 0.5 mmol, 1 equiv.). The crude mixture was purified by flash column chromatography (100% hexane to hexane 95:5 AcOEt) to afford product **1** (71 mg, 65% yield) as a colorless wax. The spectroscopic data are consistent with those reported previously.[7]

^{1}H NMR (400 MHz, $CDCl_3$) δ 7.94 (d, *J* = 8.9 Hz, 2H), 6.93 (d, *J* = 8.9 Hz, 2H), 3.86 (s, 3H), 3.22 (tt, *J* = 11.4, 3.2 Hz, 1H), 1.85 (td, *J* = 8.7, 3.6 Hz, 4H), 1.77 – 1.67 (m, 1H), 1.60 – 1.16 (m, 5H).

^{13}C NMR (101 MHz, $CDCl_3$) δ 202.6, 163.4, 130.6, 129.4, 113.9, 55.6, 45.5, 29.7, 26.1, 26.1.

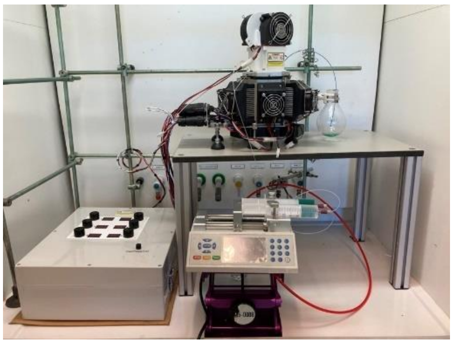

5 mmol scale reaction The model reaction was repeated on a more synthetically useful scale without changing the experimental setup or reaction conditions. To a flame-dried argon-purged 50 mL round-bottom flask, fitted with a rubber septum, charged with the nickel complex **I** (244 mg, 10 mol%), TBADT (498 mg, 3 mol%) and 4-methoxybenzoyl chloride (680 µL, 5 mmol, 1 equiv.) in CH_3CN (25 mL, 0.2 M), cyclohexane (2.7 mL, 25 mmol, 5 equiv.) was

[7] A. Trofimova, A. Holownia, C. Tien, M. J. Širvinskas, A. K. Yudin, *Org. Lett.* **2021**, 23, 9, 3294–3299.

added. The resulting solution was sonicated for 1 minute to ensure full homogeneity. A second flame-dried argon-purged 50 mL round-bottom flask, fitted with a rubber septum was charged with 2,6-dimethylpyridine (640 µL, 5.5 mmol, 1.1 equiv.) and CH_3CN (25 mL, 0.22 M). These two solutions were taken separately with a 60 mL syringe (28.8 mm of diameter), positioned on a syringe pump and connected to a T-mixer. The latter was connected to the Signify reactor, set at 144 W. The two solutions were pumped with a total 1.00 ml/min flow rate and collected at the end of the reactor.

Cyclohexyl(3-methoxyphenyl)methanone (2)

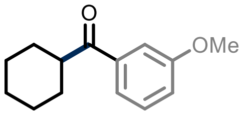

2

Prepared according to general procedure A, using cyclohexane (270 µL, 2.5 mmol, 5 equiv.) and 3-methoxybenzoyl chloride (70 µL, 0.5 mmol, 1 equiv.). The crude mixture was purified by flash column chromatography (100% hexane to hexane 95:5 AcOEt) to afford product **2** (69 mg, 63% yield) as a colorless liquid. The spectroscopic data are consistent with those reported previously.[8]

^{1}H NMR (400 MHz, $CDCl_3$) δ 7.52 (dt, *J* = 7.7, 1.3 Hz, 1H), 7.47 (dd, *J* = 2.7, 1.5 Hz, 1H), 7.36 (t, *J* = 7.9 Hz, 1H), 7.09 (ddd, *J* = 8.2, 2.7, 1.0 Hz, 1H), 3.85 (s, 3H), 3.23 (tt, *J* = 11.5, 3.3 Hz, 1H), 1.96 – 1.79 (m, 4H), 1.77 – 1.68 (m, 1H), 1.56 – 1.16 (m, 5H).
^{13}C NMR (101 MHz, $CDCl_3$) δ 203.8, 160.0, 137.9, 129.7, 129.6, 120.9, 119.1, 112.9, 55.5, 45.9, 29.6, 26.1, 26.0.

Cyclohexyl(2-methoxyphenyl)methanone (3)

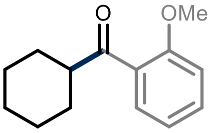

3

Prepared according to general procedure A, using cyclohexane (270 µL, 2.5 mmol, 5 equiv.) and 2-methoxybenzoyl chloride (68 µL, 0.5 mmol, 1 equiv.). The crude mixture was purified by flash column chromatography (100% hexane to hexane 98:2 AcOEt) to afford product **3** (27 mg, 25% yield) as a colorless wax. The spectroscopic data are consistent with those reported previously.[9]

^{1}H NMR (300 MHz, $CDCl_3$) δ 7.48 (dd, *J* = 7.6, 1.8 Hz, 1H), 7.41 (ddd, *J* = 8.4, 7.5, 1.8 Hz, 1H), 7.03 – 6.89 (m, 2H), 3.88 (s, 3H), 3.17 (ddt, *J* = 11.0, 6.8, 3.3 Hz, 1H), 1.96 – 1.59 (m, 5H), 1.47 – 1.17 (m, 5H).
^{13}C NMR (101 MHz, $CDCl_3$) δ 207.5, 157.8, 132.5, 129.9, 129.4, 120.8, 111.5, 55.7, 50.2, 28.9, 26.2, 26.1.

Cyclohexyl(phenyl)methanone (4)

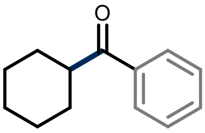

4

Prepared according to general procedure A, using cyclohexane (270 µL, 2.5 mmol, 5 equiv.) and benzoyl chloride (59 µL, 0.5 mmol, 1 equiv.). The crude mixture was purified by flash column chromatography (100% hexane to hexane 95:5 AcOEt) to afford product **4** (38 mg, 40% yield) as a colorless wax. The spectroscopic data are consistent with those reported previously.[10]

^{1}H NMR (400 MHz, $CDCl_3$) δ 7.97 – 7.91 (m, 2H), 7.57 – 7.52 (m, 1H), 7.46 (t, *J* = 7.5 Hz, 2H), 3.32 – 3.20 (m, 1H), 1.95 – 1.69 (m, 5H), 1.58 – 1.15 (m, 5H).
^{13}C NMR (101 MHz, $CDCl_3$) δ 204.0, 136.5, 132.8, 128.7, 128.4, 45.8, 29.6, 26.1, 26.0.

[8] W. G. Shuler, R. A. Swyka, T. T. Schempp, B. J. Spinello, M. J. Krische *Chem. Eur. J.* **2019**, *25*, 12517–12520.
[9] X. Zhang, Z. Wang, X. Fan, J. Wang, *J. Org. Chem.* **2015**, *80*, *21*, 10660–10667.
[10] W. Sun, L. Wang, Y. Hu, X. Wu, C. Xiao, C. Liu, *Nat. Commun*. **2020**, *11*, 3113.

[1,1'-biphenyl]-4-yl(cyclohexyl)methanone (5)

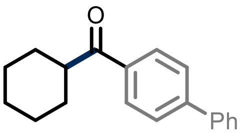

5

Prepared according to general procedure A, using cyclohexane (270 µL, 2.5 mmol, 5 equiv.) and biphenyl-4-carbonyl chloride (108 mg, 0.5 mmol, 1 equiv.). The crude mixture was purified by flash column chromatography (100% hexane to hexane 95:5 AcOEt) to afford product **5** (75 mg, 57% yield) as a white solid. The spectroscopic data are consistent with those reported previously.[11]

^{1}H NMR (400 MHz, $CDCl_3$) δ 8.09 – 8.01 (m, 2H), 7.75 – 7.68 (m, 2H), 7.67 – 7.62 (m, 2H), 7.55 – 7.47 (m, 2H), 7.46 – 7.38 (m, 1H), 3.32 (tt, J = 11.4, 3.3 Hz, 1H), 1.99 – 1.84 (m, 4H), 1.78 (dtt, J = 12.7, 3.2, 1.5 Hz, 1H), 1.64 – 1.21 (m, 5H).
^{13}C NMR (101 MHz, $CDCl_3$) δ 203.3, 145.2, 139.8, 134.8, 128.8, 128.7, 128.0, 127.1, 45.5, 29.3, 25.8, 25.7.

Cyclohexyl(4-fluorophenyl)methanone (6)

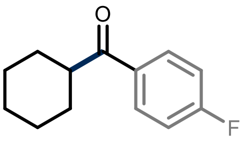

6

Prepared according to general procedure A, using cyclohexane (270 µL, 2.5 mmol, 5 equiv.) and 4-fluorobenzoyl chloride (59 µL, 0.5 mmol, 1 equiv.). The crude mixture was purified by flash column chromatography (100% hexane to hexane 95:5 AcOEt) to afford product **6** (43 mg, 42% yield) as a colorless wax. The spectroscopic data are consistent with those reported previously.[12]

^{1}H NMR (400 MHz, $CDCl_3$) δ 7.97 (dd, J = 8.8, 5.5 Hz, 2H), 7.12 (t, J = 8.6 Hz, 2H), 3.21 (tt, J = 11.4, 3.1 Hz, 1H), 1.89 – 1.70 (m, 5H), 1.58 – 1.19 (m, 5H).
^{13}C NMR (101 MHz, $CDCl_3$) δ 202.4, 165.7 (d, J = 254.1 Hz), 132.8 (d, J = 3.0 Hz), 131.0 (d, J = 9.2 Hz), 115.8 (d, J = 21.7 Hz), 45.7, 29.6, 26.1, 25.9.
^{19}F NMR (376 MHz, $CDCl_3$) δ -106.01 (tt, J = 8.4, 5.5 Hz).

Cyclohexyl(4-(trifluoromethyl)phenyl)methanone (7)

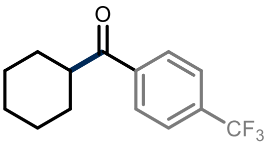

7

Prepared according to general procedure A, using cyclohexane (270 µL, 2.5 mmol, 5 equiv.) and 4-(trifluoromethyl)benzoyl chloride (74 µL, 0.5 mmol, 1 equiv.). The crude mixture was purified by preparative TLC (hexane 70:1 acetone) to afford product **7** (45 mg, 35% yield) as a white solid. The spectroscopic data are consistent with those reported previously.[13]

^{1}H NMR (400 MHz, $CDCl_3$) δ 8.03 (d, J = 8.1 Hz, 2H), 7.72 (d, J = 8.2 Hz, 2H), 3.30 – 3.19 (m, 1H), 1.91 – 1.81 (m, 4H), 1.79 – 1.71 (m, 1H), 1.55 – 1.23 (m, 5H).
^{13}C NMR (101 MHz, $CDCl_3$) δ 203.0, 139.3, 134.2 (q, J = 32.5 Hz), 128.7, 125.8 (q, J = 3.8 Hz), 123.8 (q, J = 272.8 Hz), 46.1, 29.4, 26.0, 25.9.
^{19}F NMR (376 MHz, $CDCl_3$) δ -63.10.

Cyclohexyl(1-phenylcyclopropyl)methanone (8)

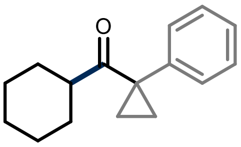

8

Prepared according to general procedure A, using cyclohexane (270 µL, 2.5 mmol, 5 equiv.) and 1-phenylcyclopropane-1-carbonyl chloride (105 mg, 0.5 mmol, 1 equiv.). The crude mixture was purified by flash column chromatography (hexane 30:1 AcOEt) to afford product **8** (84 mg, 65% yield) as a colorless wax. This compound was previously unreported.

[11] F. Picard, T. Schulz, R. W. Hartmann *Bioorg. Med. Chem.* **2002**, *10*, 437–448.
[12] F. H. Lutter, L. Grokenberger, M. S. Hofmayer, P. Knochel, *Chem. Sci*. **2019**, *10*, 8241–8245.
[13] L.Wang, T. Wang, G. Cheng, X. Li, J. Wie, B. Guo, C. Zheng, G. Chen, C. Ran, C. Zheng, *ACS Catal*. **2020**, *10*, *14*, 7543–7551.

^{1}H NMR (400 MHz, $CDCl_3$) δ 7.30 – 7.18 (m, 5H), 2.42 (tt, *J* = 11.5, 3.2 Hz, 1H), 1.76 – 1.65 (m, 1H), 1.61 – 1.53 (m, 4H), 1.46 (q, *J* = 3.6 Hz, 3H), 1.25 – 1.13 (m, 2H), 1.05 (q, *J* = 3.6 Hz, 2H), 0.97 – 0.85 (m, 2H).
^{13}C NMR (101 MHz, $CDCl_3$) δ 213.6, 141.0, 130.8, 128.5, 127.3, 47.2, 36.6, 29.0, 28.6, 25.5, 17.9.
HRMS (FI) *m/z* calcd for $C_{16}H_{20}O$: 228.1514; found: 228.1513.

Dicyclohexylmethanone (9)

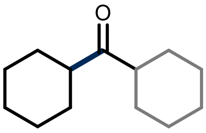

9

Prepared according to general procedure A, using cyclohexane (270 µL, 2.5 mmol, 5 equiv.) and cyclohexanecarbonyl chloride (67 µL, 0.5 mmol, 1 equiv.). The crude mixture was purified by flash column chromatography (100% hexane to hexane 99:1 AcOEt) to afford product **1** (42 mg, 43% yield) as a colorless wax. The spectroscopic data are consistent with those reported previously.[14]
^{1}H NMR (400 MHz, $CDCl_3$) δ 2.47 (td, *J* = 11.1, 2.9 Hz, 2H), 1.84 – 1.69 (m, 8H), 1.66 (d, *J* = 9.5 Hz, 2H), 1.37 – 1.16 (m, 10H).
^{13}C NMR (101 MHz, $CDCl_3$) δ 217.3, 49.3, 28.7, 26.0, 25.9.

1-cyclohexyl-3-phenylpropan-1-one (10)

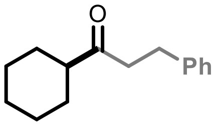

10

Prepared according to general procedure A, using cyclohexane (270 µL, 2.5 mmol, 5 equiv.) and 3-phenylpropanoyl chloride (74 µL, 0.5 mmol, 1 equiv.). The crude mixture was purified by flash column chromatography (100% hexane to hexane 95:5 AcOEt) to afford product **10** (43 mg, 40% yield) as a colorless liquid. The spectroscopic data are consistent with those reported previously.[15]
^{1}H NMR (400 MHz, $CDCl_3$) δ 7.28 (ddd, *J* = 8.6, 5.9, 1.7 Hz, 2H), 7.19 (td, *J* = 5.4, 2.8 Hz, 3H), 2.88 (dd, *J* = 8.1, 6.5 Hz, 2H), 2.76 (ddd, *J* = 8.2, 6.9, 1.0 Hz, 2H), 2.31 (td, *J* = 11.1, 5.5 Hz, 1H), 1.90 – 1.72 (m, 4H), 1.71 – 1.60 (m, 1H), 1.41 – 1.12 (m, 5H).
^{13}C NMR (101 MHz, $CDCl_3$) δ 213.3, 141.5, 128.6, 128.4, 126.1, 51.1, 42.4, 29.8, 28.5, 26.0, 25.8.

1-cyclohexyloctadecan-1-one (11)

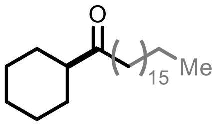

11

Prepared according to general procedure A, using cyclohexane (270 µL, 2.5 mmol, 5 equiv.) and stearoyl chloride (151 mg, 0.5 mmol, 1 equiv.). The crude mixture was purified by flash column chromatography (100% hexane to hexane 95:5 AcOEt) to afford product **11** (65 mg, 37% yield) as a white solid. This compound was previously unreported.
^{1}H NMR (400 MHz, $CDCl_3$) δ 2.43 (t, *J* = 7.4 Hz, 2H), 2.34 (tt, *J* = 11.2, 3.4 Hz, 1H), 1.91 – 1.76 (m, 4H), 1.70 – 1.64 (m, 1H), 1.59 – 1.50 (m, 2H), 1.42 – 1.19 (m, 33H), 0.90 (t, *J* = 6.7 Hz, 3H).
^{13}C NMR (101 MHz, $CDCl_3$) δ 214.7, 51.0, 49.3, 40.8, 32.1, 29.8, 29.8, 29.8, 29.8, 29.6, 29.6, 29.5, 29.5, 28.7, 28.6, 26.0, 25.9, 25.8, 23.9, 22.8, 14.3.
HRMS (FI) *m/z* calcd for $C_{24}H_{46}O$: 350.3549; found: 350.3511.

---

[14] W. Zhang, K. L. Carpenter, S. Lin, *Angew. Chem. Int. Ed.* **2019**, *59*, 409–417
[15] X.-N. Cao, X.-M. Wan, F.-L. Yang, K. Li, X.-Q. Hao, T. Shao, X. Zhu, M.-P. Song *J. Org. Chem.* **2018**, *83*, 3657–3688.

(5S,8R,9S,10S,13R,14S,17R)-17-((R)-5-cyclohexyl-5-oxopentan-2-yl)-10,13-dimethyldodecahydro-3H-cyclopenta[a]phenanthrene-3,7,12(2H,4H)-trione (12)

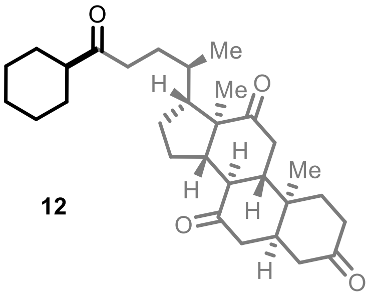

12

Prepared according to general procedure A, using cyclohexane (270 μL, 2.5 mmol, 5 equiv.) and (R)-4-((5S,8R,9S,10S,13R,14S,17R)-10,13-dimethyl-3,7,12-trioxohexadecahydro-1H-cyclopenta[a]phenanthren-17-yl)pentanoyl chloride (210 mg, 0.5 mmol, 1 equiv.). The crude mixture was purified by flash column chromatography (100% hexane to hexane 80:20 AcOEt) to afford product **12** (89 mg, 38% yield) as a white solid. The spectroscopic data are consistent with those reported previously.[16]

^{1}H NMR (400 MHz, $CDCl_3$) δ 3.01 – 2.81 (m, 3H), 2.52 (ddd, *J* = 17.1, 9.4, 4.5 Hz, 1H), 2.45 – 2.12 (m, 10H), 2.11 – 1.94 (m, 3H), 1.93 – 1.57 (m, 8H), 1.47 – 1.16 (m, 14H), 1.08 (s, 3H), 0.85 (d, *J* = 6.0 Hz, 2H).

^{13}C NMR (101 MHz, $CDCl_3$) δ 214.6, 212.0, 209.0, 208.7, 56.9, 51.8, 50.9, 49.0, 46.9, 45.6, 45.6, 45.0, 42.8, 38.6, 37.4, 36.5, 36.0, 35.3, 35.3, 29.0, 28.6, 28.5, 27. 6, 25.8, 25.7, 25.7, 25.2, 21.9, 18.8, 11.9.

(1S,2S,4aR,4bR,7S,9aS,10S,10aR)-10-(cyclohexanecarbonyl)-1-methyl-8-methylene-13-oxo-1,2,5,6,8,9,10,10a-octahydro-4a,1-(epoxymethano)-7,9a-methanobenzo[a]azulene-2,7(4bH)-diyl diacetate (13)

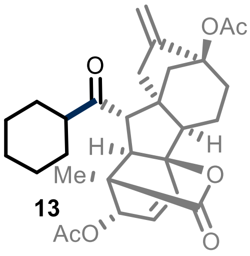

13

Prepared according to general procedure A, using cyclohexane (270 μL, 2.5 mmol, 5 equiv.) and (1S,2S,4aR,4bR,7S,9aS,10S,10aR)-10-(cyclohexanecarbonyl)-1-methyl-8-methylene-13-oxo-1,2,5,6,8,9,10,10a-octahydro-4a,1-(epoxymethano)-7,9a-methanobenzo[a]azulene-2,7(4bH)-diyl diacetate (170 mg, 0.5 mmol, 1 equiv.). The crude mixture was purified by flash column chromatography (100% hexane to hexane 80:20 AcOEt) to afford product **13** (64 mg, 34% yield) as a white solid. This compound was previously unreported.

^{1}H NMR (400 MHz, $CDCl_3$) δ 6.36 (d, *J* = 9.3 Hz, 1H), 5.85 (dd, *J* = 9.3, 3.8 Hz, 1H), 5.30 (d, *J* = 3.8 Hz, 1H), 5.15 (dd, *J* = 3.2, 1.6 Hz, 1H), 4.99 (t, *J* = 2.0 Hz, 1H), 3.45 (d, *J* = 10.3 Hz, 1H), 3.11 (d, *J* = 10.4 Hz, 1H), 2.47 – 2.28 (m, 4H), 2.20 – 1.63 (m, 15H), 1.58 – 1.44 (m, 1H), 1.37 – 1.11 (m, 6H), 1.06 (s, 3H).

^{13}C NMR (101 MHz, $CDCl_3$) δ 211.9, 177.6, 170.3, 170.2, 153.3, 134.4, 129.2, 108.5, 90.3, 84.3, 70.6, 54.4, 52.7, 52.6, 52.2, 51.6, 51.6, 43.0, 40.8, 36.4, 30.3, 27.1, 26.3, 25.8, 25.2, 22.2, 21.0, 16.8, 15.1.

HRMS (FD) *m/z* calcd for $C_{29}H_{36}O_7$: 496.2461; found: 496.2479.

Cyclopentyl(4-methoxyphenyl)methanone (14)

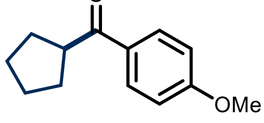

14

Prepared according to the general procedure A, using cyclopentane (233 μL, 2.5 mmol, 5 equiv.) and 4-methoxybenzoyl chloride (68 μL, 0.5 mmol, 1 equiv.). The crude mixture was purified by flash column chromatography (100% hexane to hexane 98:2 AcOEt) to afford product **14** (83 mg, 81% yield) as a colorless liquid. The spectroscopic data are consistent with those reported previously.[17]

[16] J. Amani, G. A. Molander *Org. Lett.* **2017**, *19*, 3612–3615.
[17] X. Zhang, Z. Wang, X. Fan, J. Wang *J. Org. Chem.* **2015**, *80*, 10660–10667.

^{1}H NMR (400 MHz, $CDCl_3$) δ 7.95 (d, J = 8.9 Hz, 2H), 6.92 (d, J = 8.9 Hz, 2H), 3.84 (s, 3H), 3.65 (p, J = 7.9 Hz, 1H), 1.95 – 1.81 (m, 4H), 1.80 – 1.55 (m, 4H).
^{13}C NMR (101 MHz, $CDCl_3$) δ 201.4, 163.2, 130.7, 130.0, 113.7, 55.5, 46.1, 30.1, 26.4.

(1,1-dioxidotetrahydrothiophen-3-yl)(4-methoxyphenyl)methanone (15)

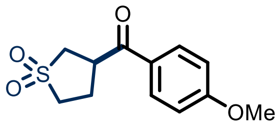

15

Prepared according to the general procedure A, using tetrahydrothiophene 1,1-dioxide (238 µL, 2.5 mmol, 5 equiv.) and 4-methoxybenzoyl chloride (68 µL, 0.5 mmol, 1 equiv.). The crude mixture was purified by two consecutive run of flash column chromatography (first column, hexane 2:1 AcOEt, second column toluene 20:1 AcOEt) to afford product **15** (78 mg, 61% yield) as a white solid. This compound was previously unreported.

^{1}H NMR (400 MHz, $CDCl_3$) δ 7.94 (d, J = 9.1 Hz, 2H), 6.98 (d, J = 9.0 Hz, 2H), 4.26 (dtd, J = 9.8, 8.3, 6.9 Hz, 1H), 3.90 (s, 3H), 3.47 (dd, J = 13.4, 9.7 Hz, 1H), 3.32 – 3.20 (m, 2H), 3.13 (dtd, J = 13.3, 7.9, 1.3 Hz, 1H), 2.60 – 2.46 (m, 1H), 2.38 (dq, J = 13.8, 8.4 Hz, 1H).
^{13}C NMR (101 MHz, $CDCl_3$) δ 195.1, 164.5, 131.1, 127.7, 114.5, 55.8, 52.6, 50.8, 41.5, 26.0.
HRMS (FI) m/z calcd for $C_{12}H_{14}O_4S$: 254.0613; found: 254.0611.

3-(4-methoxybenzoyl)cyclopentan-1-one (16)

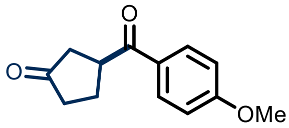

16

Prepared according to the general procedure A, using cyclopentanone (221 µL, 2.5 mmol, 5 equiv.) and 4-methoxybenzoyl chloride (68 µL, 0.5 mmol, 1 equiv.). The crude mixture was purified by flash column chromatography (100% hexane to hexane 90:10 AcOEt) to afford product **16** (86 mg, 79% yield) as a colorless liquid. The spectroscopic data are consistent with those reported previously.[18]

^{1}H NMR (400 MHz, $CDCl_3$) δ 7.97 (d, J = 8.9 Hz, 2H), 6.96 (d, J = 8.9 Hz, 2H), 4.13 – 4.02 (m, 1H), 3.88 (s, 3H), 2.87 – 2.53 (m, 1H), 2.48 – 2.06 (m, 5H).
^{13}C NMR (101 MHz, $CDCl_3$) δ 217.3, 198.8, 163.9, 130.9, 128.7, 114.1, 55.6, 42.8, 41.2, 37.5, 27.2.

4-(4-methoxybenzoyl)cycloheptan-1-one (17a) and 3-(4-methoxybenzoyl)cycloheptan-1-one (17b)

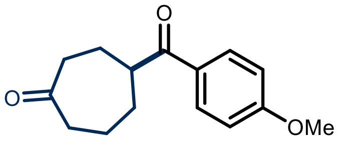

17a

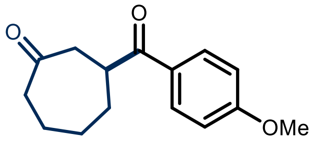

17b

Prepared according to general procedure A, using cycloheptanone (300 µL, 2.5 mmol, 5 equiv.) and 4-methoxybenzoyl chloride (68 µL, 0.5 mmol, 1 equiv.). The crude mixture was purified by column chromatography (hexane 4:1 AcOEt) to afford product **17a** (49 mg, 35% yield) and **17b** (42 mg, 31% yield) as a separable mixture of regioisomers (1:1 ratio determined by ^{1}H NMR analysis of the crude reaction mixture). These compounds were previously unreported.

*Characterization data for 4-(4-methoxybenzoyl)cycloheptan-1-one (**17a**)*

^{1}H NMR (400 MHz, $CDCl_3$) δ 7.91 (d, J = 8.7 Hz, 2H), 6.95 (d, J = 8.8 Hz, 2H), 3.87 (s, 3H), 3.41 – 3.29 (m, 1H), 2.71 – 2.50 (m, 4H), 2.20 – 2.01 (m, 3H), 1.93 (dtd, J = 14.9, 11.0, 3.7 Hz, 1H), 1.71 (t, J = 9.3 Hz, 2H).
^{13}C NMR (101 MHz, $CDCl_3$) δ 214.1, 201.3, 163.7, 130.7, 128.6, 114.1, 55.6, 48.1, 43.6, 42.2, 33.4, 26.6, 23.5.

[18] S. Dong, G. Wu, X. Yuan, C. Zou, J. Ye *Org. Chem. Front.* **2017**, *4*, 2230–2234.

*Characterization data for 3-(4-methoxybenzoyl)cycloheptan-1-one (***17b***)*
^{1}H NMR (400 MHz, $CDCl_3$) δ 7.92 (d, *J* = 8.8 Hz, 2H), 6.95 (d, *J* = 8.8 Hz, 2H), 3.87 (s, 3H), 3.59 – 3.50 (m, 1H), 2.93 (dd, *J* = 14.3, 10.9 Hz, 1H), 2.72 – 2.45 (m, 3H), 2.10 (d, *J* = 14.4 Hz, 1H), 2.06 – 1.88 (m, 2H), 1.82 – 1.68 (m, 2H), 1.58 – 1.44 (m, 1H).
^{13}C NMR (101 MHz, $CDCl_3$) δ 213.3, 200.1, 163.8, 130.8, 128.2, 114.1, 55.6, 45.7, 44.1, 43.0, 34.1, 28.3, 24.0.
HRMS (FI) *m/z* calcd for $C_{15}H_{18}O_3$: 246.1256; found: 246.1248.

(decahydronaphthalen-2-yl)(4-methoxyphenyl)methanone (18a) and (decahydronaphthalen-1-yl)(4-methoxyphenyl)methanone (18b)

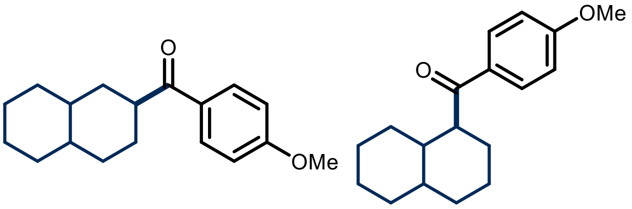

18a **18b**

Prepared according to general procedure A, using decahydronaphthalene (400 μL, 2.5 mmol, 5 equiv.) and 4-methoxybenzoyl chloride (68 μL, 0.5 mmol, 1 equiv.). The crude mixture was purified by flash column chromatography (hexane 50:1 AcOEt to hexane 10:1 AcOEt) to afford products **18a** and **18b** as an inseparable mixture of regioisomers (4:1 regioisomeric ratio after column cromatography), (50 mg, 37% yield). These compounds were previously unreported.
^{1}H NMR (400 MHz, $CDCl_3$) δ 7.93 (d, *J* = 8.9 Hz, 2H), 6.93 (d, *J* = 8.9 Hz, 2H), 3.86 (s, 3H), 3.29 (tt, *J* = 11.9, 3.3 Hz, 0.8H), 3.13 – 3.03 (m, 0.2H), 1.90 – 1.78 (m, 1H), 1.78 – 1.42 (m, 8H), 1.33 – 1.79 (m, 3H), 1.16 – 0.92 (m, 5H).
^{13}C NMR (101 MHz, $CDCl_3$) δ 203.5, 202.4, 163.5, 163.4, 131.0, 130.6, 130.5, 129.5, 113.8, 113.8, 55.6 (two carbons overlapped) 50.6, 45.6, 44.9, 42.9, 42.9, 42.5, 36.8, 34.3, 33.9, 33.9, 33.7, 33.4, 31.9, 31.4, 29.5, 26.7, 26.7 (two carbons overlapped), 26.5, 26.0.
HRMS (FI) *m/z* calcd for $C_{18}H_{24}O_2$: 272.1776; found: 272.1778.

1-(4-methoxyphenyl)-2-methylpentan-1-one (19a) and 2-ethyl-1-(4-methoxyphenyl)butan-1-one (19b)

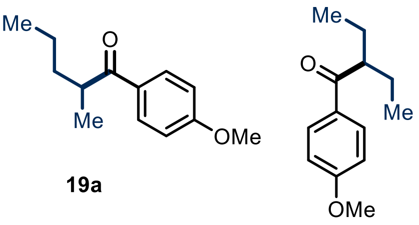

19a **19b**

Prepared according to the general procedure A, using pentane (228 μL, 2.5 mmol, 5 equiv.) and 4-methoxybenzoyl chloride (68 μL, 0.5 mmol, 1 equiv.). The crude mixture was purified by flash column chromatography (100% hexane to hexane 98:2 AcOEt) to afford product **19a** and **19b** as an inseparable mixture of regioisomers (1:1 ratio determined by ^{1}H NMR analysis of the crude reaction mixture), (46 mg, 38% yield). This compound was previously unreported.
^{1}H NMR (300 MHz, $CDCl_3$) δ 7.95 (d, *J* = 9.0 Hz, 2H), 6.94 (d, *J* = 8.9 Hz, 2H), 3.87 (s, 3H), 3.44 (q, *J* = 6.7 Hz, 0.75H), 3.31 – 3.18 (m, 0.25H), 1.89 – 1.70 (m, 1H), 1.63 – 1.50 (m, 1H), 1.45 – 1.25 (m, 2H), 1.17 (d, *J* = 6.8 Hz, 2H), 0.88 (dt, *J* = 10.1, 7.3 Hz, 4H).
^{13}C NMR (101 MHz, $CDCl_3$) δ 203.3, 203.2, 163.4, 130.6, 130.6, 129.9, 113.9, 113.8, 55.6, 48.9, 40.1, 36.3, 25.2, 20.8, 17.5, 14.3, 12.1.
HRMS (FI) *m/z* calcd for $C_{13}H_{18}O_2$: 206.1307; found: 206.1305.

Benzo[d][1,3]dioxol-2-yl(4-methoxyphenyl)methanone (20)

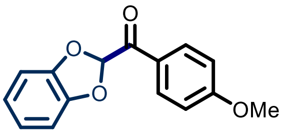

20

Prepared according to the general procedure A, using benzodioxole (288 μL, 2.5 mmol, 5 equiv.) and 4-methoxybenzoyl chloride (68 μL, 0.5 mmol, 1 equiv.). The total flow rate was adjusted to 0.33 ml/min to obtain a 15 minutes residence time. The crude mixture was purified by flash column chromatography (100% hexane to hexane 90:10 AcOEt) to afford product **20** (67 mg, 52% yield) as a light orange solid. This compound was previously unreported.

^{1}H NMR (400 MHz, $CDCl_3$) δ 8.11 – 8.03 (m, 2H), 7.04 – 6.95 (m, 2H), 6.93 – 6.83 (m, 5H), 3.89 (s, 3H).

^{13}C NMR (101 MHz, $CDCl_3$) δ 188.1, 164.6, 146.9, 132.0, 126.2, 122.3, 114.3, 109.2, 105.5, 55.7.

HRMS (FI) *m/z* calcd for $C_{15}H_{12}O_4$: 256.0736; found: 256.0729.

Bicyclo[2.2.1]heptan-2-*exo*-yl(4-methoxyphenyl)methanone (21)

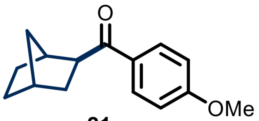

21

Prepared according to the general procedure A, using bicyclo[2.2.1]heptane (240 mg, 2.5 mmol, 5 equiv.) and 4-methoxybenzoyl chloride (68 μL, 0.5 mmol, 1 equiv.). The crude mixture was purified by flash column chromatography (100% hexane to hexane 95:5 AcOEt) to afford product **21** (59 mg, 51% yield) as a colorless liquid. This compound was previously unreported.

^{1}H NMR (400 MHz, $CDCl_3$) δ 8.00 – 7.90 (m, 2H), 6.96 – 6.88 (m, 2H), 3.86 (s, 3H), 3.16 (ddd, J = 9.0, 5.5, 1.2 Hz, 1H), 2.52 – 2.46 (m, 1H), 2.33 (dd, J = 5.1, 3.0 Hz, 1H), 2.06 – 1.94 (m, 1H), 1.69 – 1.52 (m, 2H), 1.50 – 1.37 (m, 3H), 1.32 – 1.22 (m, 1H), 1.13 (ddq, J = 9.7, 2.9, 1.4 Hz, 1H).

^{13}C NMR (101 MHz, $CDCl_3$) δ 200.2, 163.2, 130.8, 129.7, 113.7, 55.5, 49.3, 41.2, 36.4, 36.4, 33.9, 29.9, 29.2.

HRMS (FI) *m/z* calcd for $C_{15}H_{18}O_2$: 230.1277; found: 230.1276.

7-oxabicyclo[2.2.1]heptan-2-*exo*-yl)(4-methoxyphenyl)methanone (22)

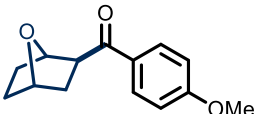

22

Prepared according to the general procedure A, using 7-oxabicyclo[2.2.1]heptane (253 μL, 2.5 mmol, 5 equiv.) and 4-methoxybenzoyl chloride (68 μL, 0.5 mmol, 1 equiv.). The crude mixture was purified by flash column chromatography (100% hexane to hexane 80:20 AcOEt) to afford product **22** (56 mg, 48% yield) as a pale yellow wax. This compound was previously unreported.

^{1}H NMR (400 MHz, $CDCl_3$) δ 7.95 – 7.89 (m, 2H), 6.98 – 6.89 (m, 2H), 4.81 (d, J = 4.8 Hz, 1H), 4.69 (t, J = 5.0 Hz, 1H), 3.87 (s, 3H), 2.32 – 2.21 (m, 1H), 2.02 – 1.46 (m, 6H).

^{13}C NMR (101 MHz, $CDCl_3$) δ 197.1, 163.5, 130.8, 129.2, 113.9, 78.6, 76.6, 55.6, 50.6, 34.3, 30.0, 29.8.

HRMS (FI) *m/z* calcd for $C_{15}H_{16}O_2$: 232.1099; found: 232.1092.

7-*anti*-bromobicyclo[2.2.1]heptan-2-*exo*-yl)(4-methoxyphenyl)methanone (23)

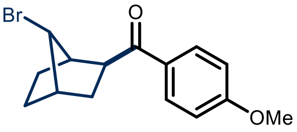

23

Prepared according to general procedure A, using 7-bromobicyclo[2.2.1]heptane (317 μL, 2.5 mmol, 5 equiv.) and 4-methoxybenzoyl chloride (68 μL, 0.5 mmol, 1 equiv.). The crude mixture was purified by flash column chromatography (hexane 20:1 AcOEt to hexane 5:1 AcOEt) to afford product **23** (83 mg, 54% yield) as a colorless oil. This compound was previously unreported.

^{1}H NMR (400 MHz, $CDCl_3$) δ 7.90 (d, *J* = 8.9 Hz, 2H), 6.93 (d, *J* = 8.9 Hz, 2H), 4.14 (s, 1H), 3.87 (s, 3H), 3.34 (dd, *J* = 10.0, 5.4 Hz, 1H), 2.56 (d, *J* = 3.9 Hz, 1H), 2.41 (t, *J* = 3.9 Hz, 1H), 2.22 – 2.02 (m, 3H), 1.65 (dd, *J* = 12.3, 10.1 Hz, 1H), 1.55 – 1.38 (m, 2H).
^{13}C NMR (101 MHz, $CDCl_3$) δ 198.7, 163.6, 130.8, 128.9, 113.9, 56.0, 55.6, 47.5, 46.8, 42.6, 31.8, 27.6, 27.2.
HRMS (FI) *m/z* calcd for $C_{15}H_{17}BrO_2$: 308.0397; found: 308.0412.

6,6-dimethyl-5-methylenebicyclo[2.2.1]heptan-2-*exo*-yl)(4-methoxyphenyl)methanone (24a) and 5,5-dimethyl-6-methylenebicyclo[2.2.1]heptan-2-exo-yl)(4-methoxyphenyl)methanone (24b)

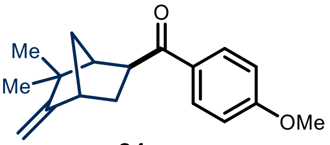

24a

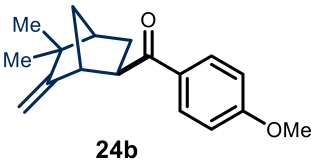

24b

Prepared according to general procedure A, using camphene (341 mg, 2.5 mmol, 5 equiv.) and 4-methoxybenzoyl chloride (68 µL, 0.5 mmol, 1 equiv.). The crude mixture was purified by flash column chromatography (hexane 20:1 AcOEt to hexane 5:1 AcOEt) to afford product **24a** and **24b** (48 mg, 36% yield) as an inseparable mixture of regioisomers (1:1 ratio determined by ^{1}H NMR analysis of the crude reaction mixture). This compound was previously unreported.

^{1}H NMR (400 MHz, $CDCl_3$) δ 7.95 (dd, *J* = 8.9, 2.1 Hz, 2H), 6.94 (d, *J* = 8.7 Hz, 2H), 4.91 (s, 0.5H), 4.83 (s, 0.5H), 4.65 (s, 0.5H), 4.60 (s, 0.5H), 3.87 (s, 3H), 3.70 (dd, *J* = 8.1, 4.6 Hz, 0.5H), 3.26 (t, *J* = 7.4 Hz, 0.5H), 2.92 (s, 0.5H), 2.76 (d, *J* = 3.5 Hz, 0.5H), 2.29 (dt, *J* = 12.0, 4.6 Hz, 0.5H), 2.19 (s, 0.5H), 2.03 (s, 0.5H), 1.95 (dd, *J* = 6.8, 3.0 Hz, 1H), 1.63 (t, *J* = 9.5 Hz, 1H), 1.53 – 1.37 (m, 1.5H), 1.31 (s, 1.5H), 1.16 (s, 1.5H), 1.09 (s, 1.5H), 1.08 (s, 1.5H).
^{13}C NMR (101 MHz, $CDCl_3$) δ 200.3, 199.3, 164.9, 164.7, 163.4, 163.3, 130.8, 130.8, 129.7, 129.5, 113.8, 113.8, 100.6, 100.5, 55.6, 52.9, 51.5, 48.2, 48.2, 46.6, 43.5, 42.8, 42.1, 35.2, 34.6, 32.3, 29.5, 29.4, 28.0, 26.0, 25.8.
HRMS (FI) *m/z* calcd for $C_{18}H_{22}O_2$: 270.1620; found: 270.1617.

(4-methoxyphenyl)(1,3,3-trimethyl-2-oxabicyclo[2.2.2]octan-5-yl)methanone (25a) and (4-methoxyphenyl)(1,3,3-trimethyl-2-oxabicyclo[2.2.2]octan-6-yl)methanone (25b)

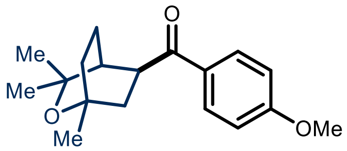

25a

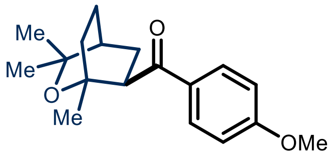

25b

Prepared according to general procedure A, using eucalyptol (419 µL, 2.5 mmol, 5 equiv.) and 4-methoxybenzoyl chloride (68 µL, 0.5 mmol, 1 equiv.). The crude mixture was purified by flash column chromatography (hexane 6:1 AcOEt to hexane 4:1 AcOEt) to afford product **25a** (40 mg, 28% yield) and product **25b** (17 mg, 12% yield) as a separable mixture of regioisomers (3.5: 1 regioisomeric ratio after column cromatography). These compounds were previously unreported.

*Characterization data for (4-methoxyphenyl)(1,3,3-trimethyl-2-oxabicyclo[2.2.2]octan-5-yl)methanone (***25a***)*

^{1}H NMR (400 MHz, $CDCl_3$) δ 7.91 (d, *J* = 8.9 Hz, 2H), 6.95 (d, *J* = 8.9 Hz, 2H), 4.06 (ddt, *J* = 10.0, 4.7, 2.3 Hz, 1H), 3.87 (s, 3H), 2.32 (dd, *J* = 13.5, 4.8 Hz, 1H), 1.82 – 1.69 (m, 2H), 1.68 – 1.52 (m, 3H), 1.48 (s, 3H), 1.42 – 1.32 (m, 1H), 1.29 (s, 3H), 1.15 (s, 3H).
^{13}C NMR (101 MHz, $CDCl_3$) δ 200.5, 163.4, 130.7, 129.5, 114.0, 73.7, 71.0, 55.6, 40.7, 37.8, 32.1, 30.9, 29.1, 28.8, 27.5, 17.2.

*Characterization data for (4-methoxyphenyl)(1,3,3-trimethyl-2-oxabicyclo[2.2.2]octan-6-yl)methanone (***25b***)*

^{1}H NMR (400 MHz, $CDCl_3$) δ 7.96 (d, *J* = 9.0 Hz, 2H), 6.94 (d, *J* = 8.9 Hz, 2H), 3.87 (s, 3H), 3.88 – 3.79 (m, 1H), 2.23 (ddt, *J* = 14.4, 11.1, 3.5 Hz, 1H), 2.15 – 2.07 (m, 1H), 2.09 – 1.95 (m,

1H), 1.81 (ddd, J = 13.3, 6.9, 2.2 Hz, 1H), 1.77 – 1.65 (m, 1H), 1.64 – 1.46 (m, 2H), 1.33 (s, 3H), 1.31 (s, 3H), 1.00 (s, 3H).

^{13}C NMR (101 MHz, $CDCl_3$) δ 201.2, 163.6, 131.2, 130.8, 113.9, 74.1, 72.6, 55.6, 47.8, 33.6, 29.2 (two carbons overlapped), 28.5, 27.9, 26.8, 22.6.

HRMS (EI) *m/z* calcd for $C_{18}H_{24}O_3$: 288.1725; found: 288.1739.

(3aR,5aS,8S,9aS,9bR)-8-(4-methoxybenzoyl)-3a,6,6,9a-tetramethyldecahydronaphtho[2,1-b]furan-2(1H)-one (26)

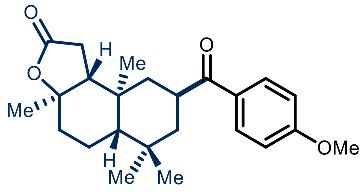

26

Prepared according to general procedure A, using sclareolide (626 mg, 2.5 mmol, 5 equiv.) and 4-methoxybenzoyl chloride (68 µL, 0.5 mmol, 1 equiv.). The crude mixture was purified by flash column chromatography (hexane 6:1 AcOEt to hexane 3:1 AcOEt) to afford product **26** (74 mg, 38% yield) as a white solid. This compound was previously unreported.

^{1}H NMR (400 MHz, $CDCl_3$) δ 7.92 (d, J = 8.8 Hz, 2H), 6.96 (d, J = 8.8 Hz, 2H), 3.88 (s, 3H), 3.70 (ddd, J = 12.4, 9.0, 3.3 Hz, 1H), 2.51 – 2.34 (m, 1H), 2.26 (dd, J = 16.2, 6.5 Hz, 1H), 2.11 (dd, J = 12.0, 3.3 Hz, 1H), 2.04 (dd, J = 14.7, 6.5 Hz, 1H), 1.94 (dd, J = 14.2, 3.5 Hz, 1H), 1.79 – 1.67 (m, 1H), 1.65 (dt, J = 13.6, 2.8 Hz, 1H), 1.62 – 1.53 (m, 1H), 1.52 – 1.38 (m, 2H), 1.36 (s, 3H), 1.25 (s, 1H), 1.17 (dd, J = 12.7, 2.8 Hz, 1H), 1.07 (s, 3H), 1.00 (s, 3H), 0.96 (s, 3H).

^{13}C NMR (101 MHz, $CDCl_3$) δ 201.4, 176.5, 163.6, 130.7, 129.2, 114.1, 86.2, 58.9, 56.4, 55.6, 45.1, 41.9, 38.7, 37.2, 36.6, 34.0, 33.0, 28.8, 21.8, 21.6, 20.6, 16.1.

HRMS (EI) *m/z* calcd for $C_{24}H_{32}O_4$: 384.230; found: 384.2305.

3.2. General Procedure B (Photochemical Arylation of C–H Bonds in Flow)

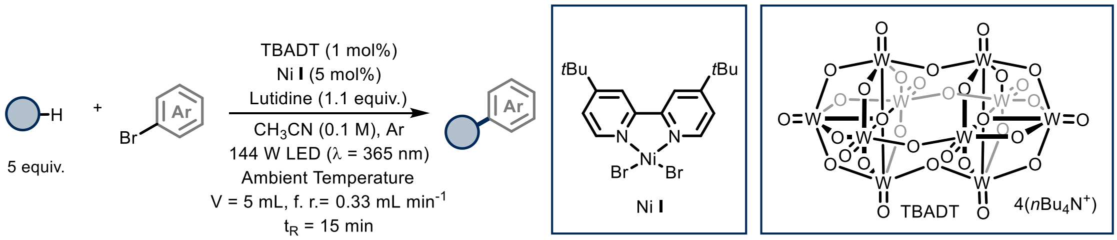

To a flame-dried argon-purged screw-capped vial, fitted with a rubber septum, charged with nickel complex **I** (12.2 mg, 5 mol%), TBADT (16.6 mg, 1 mol%) and the aryl bromide (0.5 mmol, 1 equiv.) in CH_3CN (2.5 mL, 0.20 M), the alkane derivative was added (5 equiv.). The resulting solution was sonicated for 1 minute to ensure full homogeneity. A second flame-dried argon-purged screw-capped vial fitted with a rubber septum was charged with 2,6-dimethylpyridine (64 µL, 0.55 mmol, 1.1 equiv.) and CH_3CN (2.5 mL, 0.22 M). These two solutions were taken separately with a 6 mL syringe (12.4 mm of diameter), positioned on a syringe pump and connected to a T-mixer. The latter was connected to the Signify reactor set at 144W. The two solutions were pumped, unless specified somewhere else, with a total 0.33 ml/min flow rate and collected at the end of the reactor. The solvent of the resulting reaction mixture was removed under reduced pressure and purification by flash column chromatography on silica gel gave the corresponding products in the stated yield.

- Characterization Data of Products

Methyl 4-cyclohexylbenzoate (27)

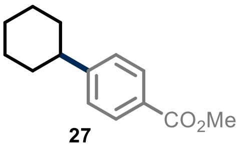

27

Prepared according to the general procedure B, using cyclohexane (270 μL, 2.5 mmol, 5 equiv.) and methyl 4-bromobenzoate (108 mg, 0.5 mmol, 1 equiv.). The crude mixture was purified by flash column chromatography (100% hexane to hexane 98:2 AcOEt) to afford product **27** (75 mg, 69% yield) as a colorless liquid. The spectroscopic data are consistent with those reported previously.[2]

^{1}H NMR (400 MHz, $CDCl_3$) δ 7.98 (d, J = 8.4 Hz, 2H), 7.29 (d, J = 8.4 Hz, 2H), 3.92 (s, 3H), 2.58 (ddd, J = 11.5, 8.1, 3.5 Hz, 1H), 1.88 (tt, J = 7.4, 4.4 Hz, 4H), 1.82 –1.73 (m, 1H), 1.54 – 1.36 (m, 4H), 1.30 – 1.26 (m, 1H).
^{13}C NMR (75 MHz, $CDCl_3$) δ 167.3, 153.6, 129.8, 127.9, 127.0, 52.0, 44.8, 34.3, 26.9, 26.2.

4-Cyclohexylbenzonitrile (28)

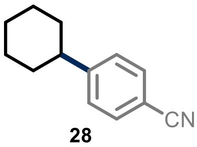

28

Prepared according to the general procedure B, using cyclohexane (270 μL, 2.5 mmol, 5 equiv.) and 4-bromobenzonitrile (91 mg, 0.5 mmol, 1 equiv.). The crude mixture was purified by flash column chromatography (100% hexane to hexane 90:10 AcOEt) to afford product **28** (46 mg, 50% yield) as a white solid. The spectroscopic data are consistent with those reported previously.[2]

^{1}H NMR (400 MHz, $CDCl_3$) δ 7.55 (m, 2H), 7.32 – 7.27 (m, 2H), 2.61 – 2.49 (m, 1H), 1.93 – 1.82 (m, 4H), 1.82 – 1.71 (m, 1H), 1.49 – 1.32 (m, 4H), 1.31 – 1.19 (m, 1H).
^{13}C NMR (101 MHz, $CDCl_3$) δ 153.6, 132.3, 127.8, 119.3, 109.7, 44.9, 34.1, 26.7, 26.0.

1-Cyclohexyl-3,5-bis(trifluoromethyl)benzene (29)

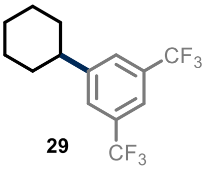

29

Prepared according to general procedure B, using cyclohexane (270 μL, 2.5 mmol, 5 equiv.) and 1-bromo-3,5-bis(trifluoromethyl)benzene (146 mg, 0.5 mmol, 1 equiv.). The crude mixture was purified by flash column chromatography (100% hexane) to afford product **29** (64 mg, 43% yield) as a white solid. The spectroscopic data are consistent with those reported previously.[19]

^{1}H NMR (400 MHz, $CDCl_3$) δ 7.70 (s, 1H), 7.65 (s, 2H), 2.65 (tt, J = 11.4, 3.3 Hz, 1H), 1.90 (td, J = 10.5, 5.0 Hz, 4H), 1.84 – 1.74 (m, 1H), 1.57 – 1.33 (m, 4H), 1.33 – 1.18 (m, 1H).
^{13}C NMR (101 MHz, $CDCl_3$) δ 150.4, 131.5 (q, J = 32.8 Hz), 127.1 (d, J = 4.2 Hz), 123.7 (q, J = 273.6 Hz), 120.0 (p, J = 3.9 Hz), 44.5, 34.3, 26.7, 26.0.
^{19}F NMR (376 MHz, $CDCl_3$) δ -62.82.

5-cyclohexyl-2-(trifluoromethyl)pyridine (30)

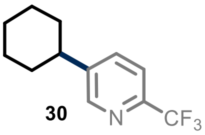

30

Prepared according to general procedure B, using cyclohexane (270 μL, 2.5 mmol, 5 equiv.) and 2-bromo-5-(trifluoromethyl)pyridine (113 mg, 0.5 mmol, 1 equiv.). The crude mixture was purified by flash column chromatography (hexane 10:1 AcOEt) to afford product **30** (87 mg, 76% yield) as a white solid. The spectroscopic data are consistent with those reported previously.[2]

[19] J. S. Bair, Y. Schramm, A. G. Sergeev, E. Clot, O. Eisenstein, J. F. Hartwig *J. Am. Chem. Soc.* **2014**, *136*, 13098–13101.

^{1}H NMR (400 MHz, $CDCl_3$) δ 8.57 (d, *J* = 1.6 Hz, 1H), 7.67 (dd, *J* = 8.1, 2.0 Hz, 1H), 7.60 (d, *J* = 8.1 Hz, 1H), 2.69 – 2.57 (m, 1H), 1.91 – 1.85 (m, 4H), 1.82 – 1.74 (m, 1H), 1.50 – 1.35 (m, 4H), 1.29 (ddd, *J* = 12.8, 9.7, 3.1 Hz, 1H).
^{13}C NMR (101 MHz, $CDCl_3$) δ 149.4, 146.5, 146.0 (q, *J* = 34.6 Hz), 135.4, 121.9 (q, *J* = 273.6 Hz), 120.3 (q, *J* = 2.7 Hz), 42.1, 34.0, 26.7, 25.9.
^{19}F NMR (376 MHz, $CDCl_3$) δ -67.68.

5-Cyclohexylpyrimidine-2-carbonitrile (31)

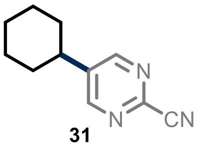

31

Prepared according to general procedure B, using cyclohexane (270 μL, 2.5 mmol, 5 equiv.) and 5-bromopyrimidine-2-carbonitrile (92 mg, 0.5 mmol, 1 equiv.). The crude mixture was purified by flash column chromatography (hexane 15:1 AcOEt to hexane 5:1 AcOEt) to afford product **31** (37 mg, 40% yield) as a white solid. This compound was previously unreported.
^{1}H NMR (400 MHz, $CDCl_3$) δ 8.67 (s, 2H), 2.63 (ddd, *J* = 11.7, 8.3, 3.4 Hz, 1H), 1.98 – 1.84 (m, 4H), 1.79 (ddt, *J* = 14.0, 6.0, 2.2 Hz, 1H), 1.53 – 1.34 (m, 4H), 1.34 – 1.20 (m, 1H).
^{13}C NMR (101 MHz, $CDCl_3$) δ 156.8, 143.2, 143.1, 116.0, 40.2, 33.5, 26.3, 25.6.
HRMS (FI) *m/z* calcd for $C_{11}H_{13}N_3$: 187.1109; found: 187.1099.

4-Cyclohexyl-1-tosyl-1*H*-pyrrolo[2,3-*b*]pyridine (32)

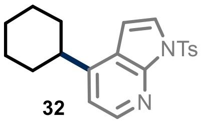

32

Prepared according to general procedure B, using nickel complex **I** (24.4 mg, 10 mol%), TBADT (49.8 mg, 3 mol%), cyclohexane (270 μL, 2.5 mmol, 5 equiv.) and 4-bromo-1-tosyl-1H-pyrrolo[2,3-b]pyridine (176 mg, 0.5 mmol, 1 equiv.). The crude mixture was purified by flash column chromatography (100% hexane to hexane 5:1 AcOEt) to afford product **32** (100 mg, 56% yield) as a pale-yellow solid. This compound was previously unreported.
^{1}H NMR (400 MHz, $CDCl_3$) δ 8.36 (d, *J* = 5.0 Hz, 1H), 8.13 – 8.04 (m, 2H), 7.70 (d, *J* = 4.1 Hz, 1H), 7.32 – 7.23 (m, 2H), 7.02 (d, *J* = 5.1 Hz, 1H), 6.67 (d, *J* = 4.0 Hz, 1H), 2.85 (tt, *J* = 11.8, 3.1 Hz, 1H), 2.36 (s, 3H), 1.94 – 1.83 (m, 4H), 1.83 – 1.75 (m, 1H), 1.57 – 1.29 (m, 5H).
^{13}C NMR (101 MHz, $CDCl_3$) δ 150.5, 147.3, 145.3, 145.1, 135.7, 129.7, 128.1, 125.4, 121.7, 115.98, 103.8, 41.5, 33.0, 26.7, 26.1, 21.7.
HRMS (FD) *m/z* calcd for $C_{20}H_{22}N_2O_2S$: 354.1402; found: 354.1404.

4-Cyclohexylphenyl acetate (33)

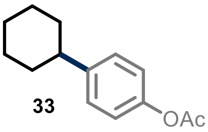

33

Prepared according to general procedure B, using nickel complex **I** (24.4 mg, 10 mol%), TBADT (49.8 mg, 3 mol%), cyclohexane (270 μL, 2.5 mmol, 5 equiv.) and 4-bromophenyl acetate (108 mg, 0.5 mmol, 1 equiv.). The total flow rate was adjusted to 0.111 ml/min to obtain a 45 minutes residence time. The crude mixture was purified by flash column chromatography (hexane 40:1 AcOEt to hexane 20:1 AcOEt) to afford product **33** (40 mg, 36% yield) as a colorless oil. The spectroscopic data are consistent with those reported previously.[2]
^{1}H NMR (400 MHz, $CDCl_3$) δ 7.21 (d, *J* = 8.7 Hz, 2H), 7.00 (d, *J* = 8.6 Hz, 2H), 2.50 (ddt, *J* = 11.4, 6.6, 4.0 Hz, 1H), 2.29 (s, 3H), 1.95 – 1.79 (m, 4H), 1.75 (dtd, *J* = 12.7, 3.3, 1.8 Hz, 1H), 1.45 – 1.36 (m, 5H).
^{13}C NMR (101 MHz, $CDCl_3$) δ 169.9, 148.7, 145.7, 127.8, 121.3, 44.1, 34.6, 29.8, 26.9, 26.2, 21.3.

2-(4-Cyclohexylphenyl)-4,4,5,5-tetramethyl-1,3,2-dioxaborolane (34)

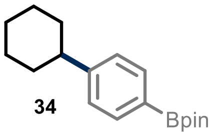

Prepared according to general procedure B, using nickel complex **I** (24.4 mg, 10 mol%), TBADT (49.8 mg, 3 mol%), cyclohexane (270 µL, 2.5 mmol, 5 equiv.) and 2-(4-bromophenyl)-4,4,5,5-tetramethyl-1,3,2-dioxaborolane (141 mg, 0.5 mmol, 1 equiv.). The total flow rate was adjusted to 0.167 ml/min to obtain a 30 min residence time. The crude mixture was purified by flash column chromatography (100% hexane to hexane 30:70 toluene) to afford product **34** (47 mg, 33% yield) as a white solid. The spectroscopic data are consistent with those reported previously.[20]

^{1}H NMR (400 MHz, $CDCl_3$) δ 7.76 (d, *J* = 7.8 Hz, 2H), 7.24 (d, *J* = 7.8 Hz, 2H), 2.52 (tt, *J* = 11.7, 3.3 Hz, 1H), 1.94 – 1.81 (m, 4H), 1.80 – 1.71 (m, 1H), 1.51 – 1.37 (m, 4H), 1.37 – 1.32 (m, 13H).

^{13}C NMR (101 MHz, $CDCl_3$) δ 151.5, 134.9, 126.3, 83.6, 44.8, 34.2, 26.8, 26.1, 24.8.

^{11}B NMR (128 MHz, $CDCl_3$) δ 30.95.

5-(Tetrahydrofuran-2-yl)-2-(trifluoromethyl)pyridine (35)

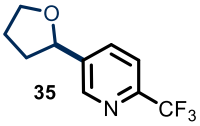

Prepared according to general procedure B, using tetrahydrofuran (193 µL, 2.5 mmol, 5 equiv.) and 5-bromo-2-(trifluoromethyl)pyridine (113 mg, 0.5 mmol, 1 equiv.). The crude mixture was purified by flash column chromatography (hexane 10:1 AcOEt to hexane 5:1 AcOEt) to afford product **35** (57 mg, 55% yield) as a colorless oil. The spectroscopic data are consistent with those reported previously.[2]

^{1}H NMR (400 MHz, $CDCl_3$) δ 8.66 (d, *J* = 2.1 Hz, 1H), 7.84 (dd, *J* = 8.1, 2.1 Hz, 1H), 7.64 (d, *J* = 8.1 Hz, 1H), 4.99 (t, *J* = 7.2 Hz, 1H), 4.10 (dt, *J* = 8.4, 6.8 Hz, 1H), 4.02 – 3.92 (m, 1H), 2.43 (dtd, *J* = 12.9, 7.1, 5.8 Hz, 1H), 2.04 (dddd, *J* = 13.8, 9.6, 6.7, 3.0 Hz, 2H), 1.78 (dq, *J* = 12.3, 7.8 Hz, 1H).

^{13}C NMR (101 MHz, $CDCl_3$) δ 147.9, 147.2 (q, *J* = 34.6 Hz), 142.5, 134.6, 121.7 (q, *J* = 273.8 Hz), 120.2 (q, *J* = 2.8 Hz), 78.0, 69.1, 34.7, 26.0.

^{19}F NMR (376 MHz, $CDCl_3$) δ -67.77.

***Tert*-butyl 2-(6-(trifluoromethyl)pyridin-3-yl)pyrrolidine-1-carboxylate (36)**

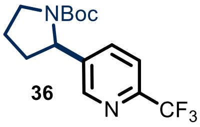

Prepared according to general procedure B, using tert-butyl pyrrolidine-1-carboxylate (438 µL, 2.5 mmol, 5 equiv.) and 5-bromo-2-(trifluoromethyl)pyridine (113 mg, 0.5 mmol, 1 equiv.). The crude mixture was purified by flash column chromatography (hexane 30:1 AcOEt to hexane 10:1 AcOEt) to afford product **36** (95 mg, 60% yield) as a colorless oil. The spectroscopic data are consistent with those reported previously.[2]

^{1}H NMR (400 MHz, $CDCl_3$) δ 8.57 (s, 1H), 7.70 – 7.59 (m, 2H), 5.08 – 4.79 (m, 1H), 3.70 – 3.52 (m, 2H), 2.41 (dt, *J* = 15.6, 7.5 Hz, 1H), 1.92 (p, *J* = 6.2 Hz, 2H), 1.82 (dt, *J* = 11.9, 6.2 Hz, 1H), 1.45 (s, 4H), 1.22 (d, *J* = 20.7 Hz, 5H).

^{13}C NMR (125 MHz, $CDCl^3$) δ 154.6 & 154.2 (rotameric singlets), 148.2 & 147.9 (rotameric singlets), 146.8 (q, J = 35.1 Hz), 143.9 & 142.9 (rotameric singlets), 134.5 & 134.3 (rotameric singlets), 121.6 (q, J = 273.7 Hz), 120.3 & 120.2 (broad rotameric singlets), 80.1, 59.1 & 58.8 (rotameric singlets), 47.5 & 47.3 (rotameric singlets), 35.9 & 34.6 (rotameric singlets), 28.6 & 28.3 (rotameric singlets), 23.8 & 23.5 (rotameric singlets).

^{19}F NMR *(*376 MHz, $CDCl_3$) δ -67.71.

[20] J. Wu, Z. Wang, X.-Y. Chen, Y. Wu, D. Wang, Q. Peng, P. Wang *Sci. China Chem.* **2020**, *63*, 336–340.

5-(2,3-Dihydro-1*H*-inden-1-yl)-2-(trifluoromethyl)pyridine (37)

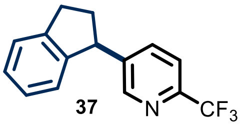

37

Prepared according to general procedure B, using 2,3-dihydro-1H-indene (306 µL, 2.5 mmol, 5 equiv.) and 5-bromo-2-(trifluoromethyl)pyridine (113 mg, 0.5 mmol, 1 equiv.). The crude mixture was purified by flash column chromatography (hexane 30:1 AcOEt to hexane 20:1 AcOEt) to afford product **37** (92 mg, 70% yield) as a yellow oil. This compound was previously unreported.

^{1}H NMR (400 MHz, $CDCl_3$) δ 8.64 (s, 1H), 7.63 (d, *J* = 1.5 Hz, 2H), 7.36 (d, *J* = 7.5 Hz, 1H), 7.26 (t, *J* = 7.4 Hz, 1H), 7.19 (t, *J* = 7.5 Hz, 1H), 6.95 (d, *J* = 7.5 Hz, 1H), 4.48 (t, *J* = 8.2 Hz, 1H), 3.18 – 2.98 (m, 2H), 2.75 – 2.62 (m, 1H), 2.05 (dqd, *J* = 12.7, 8.5, 1.2 Hz, 1H).

^{13}C NMR (101 MHz, $CDCl_3$) δ 150.0, 146.4 (q, *J* = 34.6 Hz), 144.7, 144.3, 144.2, 136.5, 127.3, 126.8, 124.7, 124.6, 122.1 (q, *J* = 273.7 Hz), 120.5 (q, *J* = 2.9 Hz), 48.7, 36.3, 31.7.

^{19}F NMR (376 MHz, $CDCl_3$) δ -67.68.

HRMS (FD) *m/z* calcd for $C_{15}H_{12}F_3N$: 263.0922; found: 263.0913.

Methyl 4-(bicyclo[2.2.1]heptan-1-yl)benzoate (38)

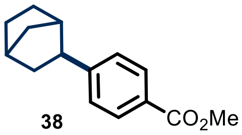

38

Prepared according to general procedure B, using bicyclo[2.2.1]heptane (240 mg, 2.5 mmol, 5 equiv.) and methyl 4-bromobenzoate (108 mg, 0.5 mmol, 1 equiv.). The crude mixture was purified by flash column chromatography (100% hexane to hexane 98:2 AcOEt) to afford product **38** (75 mg, 65% yield) as a colorless oil. This compound was previously unreported.

^{1}H NMR (300 MHz, $CDCl_3$) δ 7.86 (d, *J* = 8.4 Hz, 2H), 7.26 – 7.09 (m, 2H), 3.82 (s, 3H), 2.71 (dd, *J* = 9.1, 5.6 Hz, 1H), 2.39 – 2.24 (m, 2H), 1.72 (ddd, *J* = 11.6, 9.0, 2.3 Hz, 1H), 1.66 – 1.36 (m, 4H), 1.36 – 1.08 (m, 3H).

^{13}C NMR (75 MHz, $CDCl_3$) δ 167.3, 153.2, 129.7, 127.4, 127.2, 52.1, 47.6, 42.8, 39.3, 37.0, 36.3, 30.7, 28.9.

HRMS (FI) *m/z* calcd for $C_{15}H_{18}O_2$: 230.1307; found: 230.1295.

Methyl 4-((3aR,5aS,8S,9aS,9bR)-3a,6,6,9a-tetramethyl-2-oxododecahydronaphtho[2,1-b]furan-8-yl)benzoate (39)

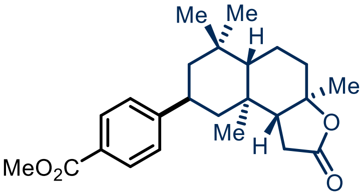

39

Prepared according to the general procedure, using sclareolide (626 mg, 2.5 mmol, 5 equiv.) and methyl 4-bromobenzoate (108 mg, 0.5 mmol, 1 equiv.). The crude mixture was purified by two consecutive run of flash column chromatography (first column, hexane 4:1 AcOEt, second column toluene 4:1 AcOEt) to afford product **39** (88 mg, 46% yield) as a white solid. This compound was previously unreported.

^{1}H NMR (400 MHz, $CDCl_3$) δ 7.99 (d, *J* = 8.3 Hz, 2H), 7.30 (d, *J* = 8.4 Hz, 2H), 3.92 (s, 3H), 3.05 (td, *J* = 11.1, 6.3 Hz, 1H), 2.53 – 2.38 (m, 1H), 2.25 (dd, *J* = 16.2, 6.4 Hz, 1H), 2.15 (dt, *J* = 11.8, 3.1 Hz, 1H), 2.11 – 2.03 (m, 1H), 2.00 – 1.93 (m, 1H), 1.76 (td, *J* = 12.4, 3.9 Hz, 1H), 1.71 – 1.58 (m, 2H), 1.46 (d, *J* = 13.1 Hz, 1H), 1.39 (s, 3H), 1.34 – 1.15 (m, 3H), 1.08 (s, 3H), 0.99 (s, 6H).

^{13}C NMR (101 MHz, $CDCl_3$) δ 176.6, 167.1, 151.7, 130.0, 128.3, 127.2, 86.3, 77.5, 77.2, 76.8, 59.2, 56.5, 52.1, 49.7, 46.9, 38.8, 37.0, 35.9, 34.2, 33.2, 28.8, 21.8, 21.4, 20.6, 15.9.

HRMS (FD) *m/z* calcd for $C_{24}H_{32}O_4$: 384.2301; found: 384.2311.

5-((3a*R*,5a*S*,9a*S*,9b*R*)-3a,6,6,9a-Tetramethyldodecahydronaphtho[2,1-*b*]furan-2-yl)-2-(trifluoromethyl)pyridine (40)

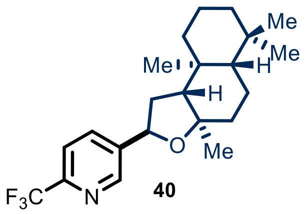

40

Prepared according to general procedure B, using ambroxide((591 mg, 2.5 mmol, 5 equiv.) and 5-bromo-2-(trifluoromethyl)pyridine (113 mg, 0.5 mmol, 1 equiv.). Final aryl bromide concentration was lowered to 0.05M in a single feed. The crude mixture was purified by flash column chromatography (100% hexane to hexane 30:1 AcOEt) to afford product **40** (112 mg, 59% yield) as a yellow oil. This compound was previously unreported.

^{1}H NMR (400 MHz, $CDCl_3$) δ 8.68 (s, 1H), 7.87 – 7.82 (m, 1H), 7.63 (d, J = 8.1 Hz, 1H), 5.24 – 5.19 (m, 1H), 2.38 (ddd, J = 13.5, 11.6, 9.7 Hz, 1H), 2.08 (dt, J = 11.8, 3.2 Hz, 1H), 1.83 (dd, J = 13.9, 3.4 Hz, 1H), 1.69 – 1.49 (m, 5H), 1.45 – 1.33 (m, 4H), 1.26 (s, 3H), 1.22 – 1.09 (m, 2H), 0.99 (dd, J = 12.5, 2.6 Hz, 1H), 0.88 (d, J = 4.8 Hz, 6H), 0.84 (s, 3H).

^{13}C NMR (101 MHz, $CDCl_3$) δ 148.1, 146.9 (q, J = 34.8 Hz), 144.1, 134.6, 121.8 (q, J = 273.6 Hz), 120.2 (q, J = 2.8 Hz), 82.7, 74.7, 58.7, 57.5, 42.5, 40.0, 39.9, 36.4, 33.7, 33.2, 32.3, 21.8, 21.2, 20.8, 18.4, 15.2.

^{19}F NMR (376 MHz, $CDCl_3$) δ -67.78.

HRMS (FD) *m/z* calcd for $C_{22}H_{30}F_3NO$: 381.2279; found: 381.2262.

4. Limitation of the Scope

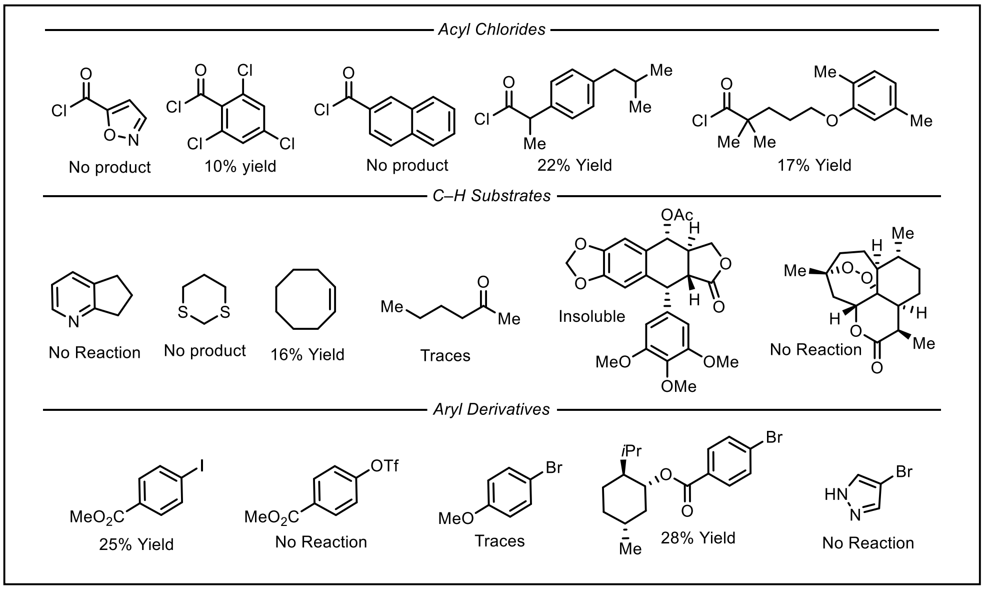

Figure S4: Overview of some unsuccessful substrates due to low yield or no reaction.

5. Kinetic Studies

5.1. Reaction Profile

- Profile of the reaction in flow

Seven flame-dried argon-purged screw-capped vials, fitted with a rubber septum, containing the nickel complex **I** (9.7 mg, 10 mol%) and TBADT (19.9 mg, 3 mol%), were charged with 4-methoxy benzoyl chloride (27 µL, 0.2 mmol, 1 equiv.) and cyclohexane (108 µL, 5 equiv.) in CH_3CN (1 mL, 0.20 M). Other seven flame-dried argon-purged screw-capped vials fitted with a rubber septum were charged with 2,6-dimethylpyridine (26 µL, 0.22 mmol, 1.1 equiv.) and CH_3CN (1 mL, 0.22 M). For each of the seven points, these solutions were taken separately with 3 mL syringes (9.8 mm of diameter), positioned on a syringe pump and connected to a T-mixer connected to the Signify reactor equipped with the 5 mL coil, set at 144 W. The two solutions were pumped, with a residence time of 2.5, 5, 10, 30, 60, 120 and 300 seconds, and collected at the end of the reactor. The crude mixture of each reaction was then concentrated in vacuo, suspended in ethyl acetate and filtered on a pad of silica to ensure removal of the inorganic salts. Trichloroethylene (18 µL, 0.2 mmol) was added as the internal standard and an aliquot of the resulting solution was taken to be directly analyzed by ^1H NMR.

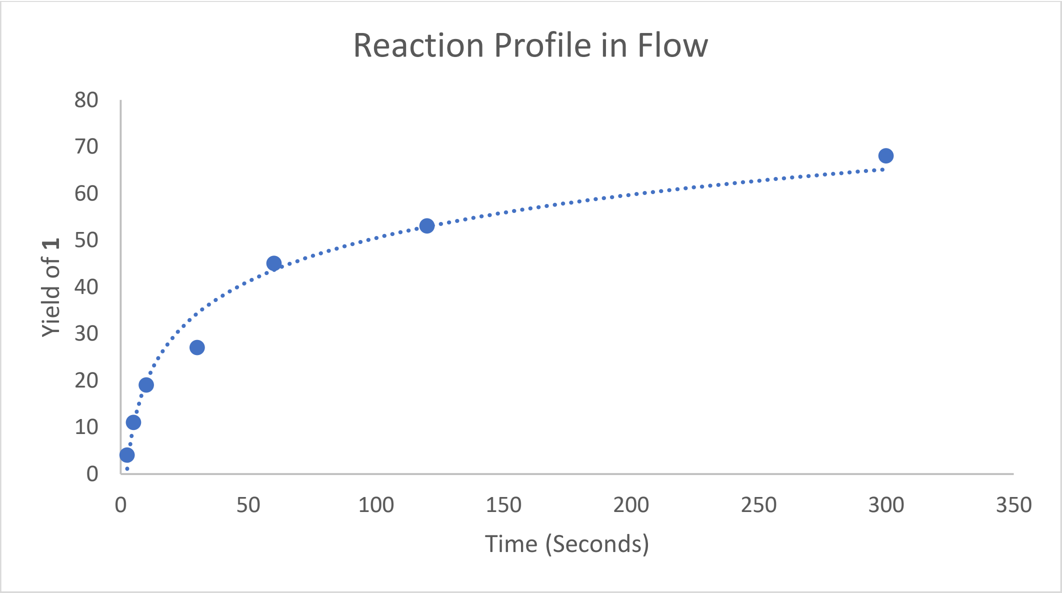

Figure S5: Reaction profile of the acylation coupling reaction between 4-methoxy benzoyl chloride and cyclohexane in flow.

- Profile of the reaction in Batch

Seven flame-dried argon-purged screw-capped vials, fitted with a rubber septum, containing the nickel complex **I** (9.7 mg, 10 mol%) and TBADT (19.9 mg, 3 mol%), were charged with 4-methoxy benzoyl chloride (27 µL, 0.2 mmol, 1 equiv.), 2,6-dimethylpyridine (26 µL, 1.1 equiv.) and cyclohexane (108 µL, 5 equiv.) in CH_3CN (2 mL, 0.1 M). The vials were further flushed with argon, sealed with Parafilm, and then placed 5 cm away from two 34W 390 nm Kessil Lamps with adequate fans to keep the reaction mixture at 35 °C. The reactions were stirred under irradiation for 5, 10, 30, 60, 180, 420 and 720 minutes, respectively. The crude mixture of each reaction was then concentrated in vacuo, suspended in ethyl acetate and filtered on a pad of silica to ensure removal of the inorganic salts. Trichloroethylene (18 µL, 0.2 mmol) was added as the internal standard and an aliquot of the resulting solution was taken to be directly analyzed by ^{1}H NMR.

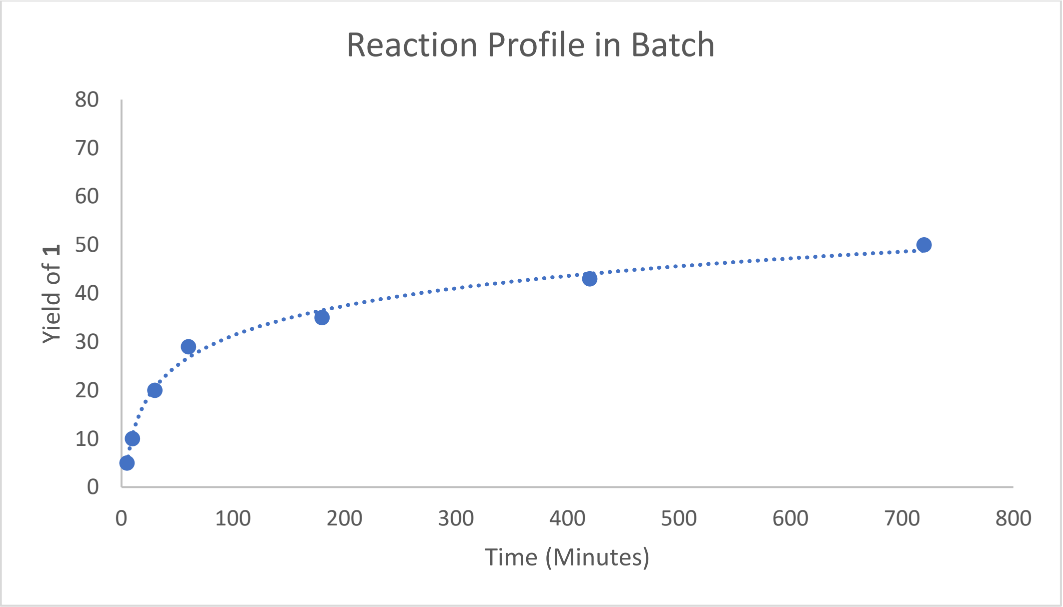

Figure S6: Reaction profile of the acylation coupling reaction between 4-methoxy benzoyl chloride and cyclohexane in batch.

5.2. Light Intensity Assessment

A flame-dried argon-purged screw-capped vial, fitted with a rubber septum, containing the nickel complex **I** (9.7 mg, 10 mol%) and TBADT (19.9 mg, 3 mol%), was charged with 4-methoxy benzoyl chloride (27 µL, 0.2 mmol, 1 equiv.) and cyclohexane (108 µL, 5 equiv.) in CH_3CN (1 mL, 0.20 M). A second flame-dried argon-purged screw-capped vial fitted with a rubber septum was charged with 2,6-dimethylpyridine (26 µL, 0.22 mmol, 1.1 equiv.) and CH_3CN (1 mL, 0.22 M). These two solutions were taken separately with 3 mL syringes (9.8 mm of diameter), positioned on a syringe pump and connected to a T-mixer connected to the Signify reactor equipped with the 0.23 mL coil (Figure S3 right). The two solutions were pumped, with a residence time of 2.5, 5, 7.5 and 10 seconds, and collected at the end of the reactor. The crude mixture was then concentrated in vacuo, suspended in ethyl acetate and filtered on a pad of silica to ensure removal of the inorganic salts. Trichloroethylene (18 µL, 0.2 mmol) was added as the internal standard and an aliquot of the resulting solution was taken to be directly analyzed by ^{1}H NMR. This was repeated screening different light intensities: 24, 71, 112 and 144 W.

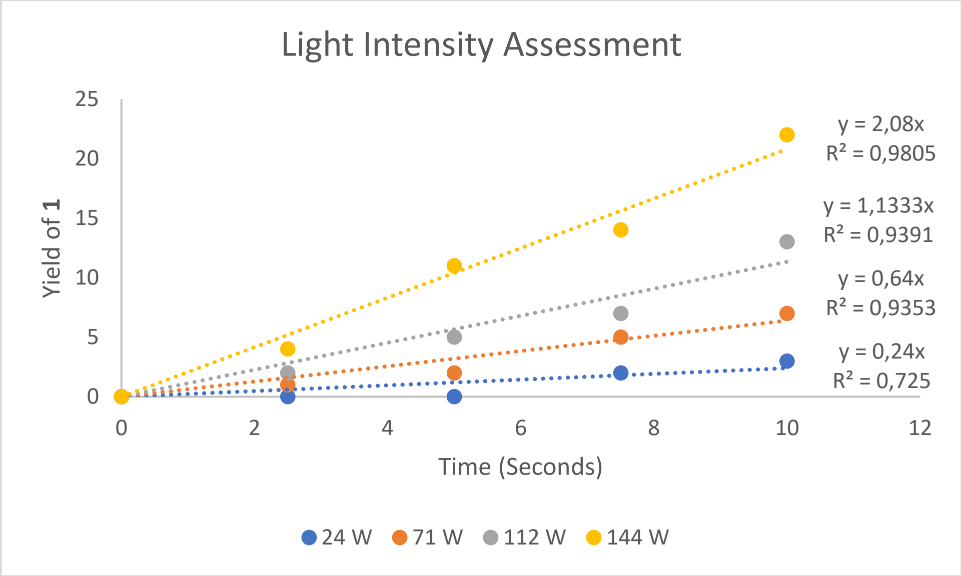

Figure S7: Evaluation of the influence of the light intensity over the rate of the acylation coupling reaction between 4-methoxy benzoyl chloride and cyclohexane performed under microfluidic conditions.

5.3 Kinetic Isotope Effect Studies

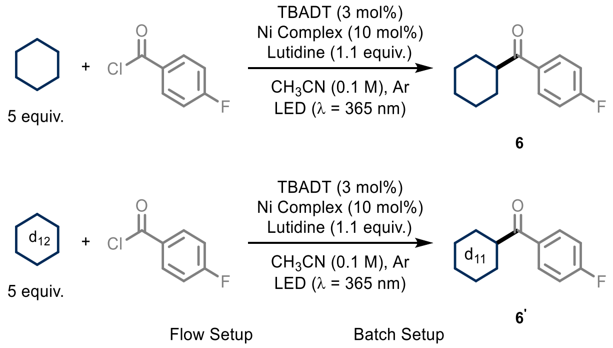

Flow Setup
K_H/K_D :1.8

Batch Setup
K_H/K_D :1.8

- Kinetic Isotope Effect in Flow

A flame-dried argon-purged screw-capped vial, fitted with a rubber septum, containing the nickel complex **I** (9.7 mg, 10 mol%) and TBADT (19.9 mg, 3 mol%), was charged with 4-fluoro benzoyl chloride (24 µL, 0.2 mmol, 1 equiv.) and cyclohexane or cyclohexane-d_{12} (108 µL, 5 equiv.) in CH_3CN (1 mL, 0.20 M). A second flame-dried argon-purged screw-capped vial fitted with a rubber septum was charged with 2,6-dimethylpyridine (26 µL, 0.22 mmol, 1.1 equiv.) and CH_3CN (1 mL, 0.22 M). These two solutions were taken separately with 3 mL syringes (9.8 mm of diameter), positioned on a syringe pump and connected to a T-mixer connected to the Signify reactor equipped with the 0.23 mL coil (Figure S3 right) set at 144 W. The two solutions were pumped, with a residence time of 2.5, 5, and 7.5 seconds, and collected at the end of the reactor. The crude mixture was then concentrated in vacuo, suspended in ethyl acetate and filtered on a pad of silica to ensure removal of the inorganic salts. 1,2-difluorobenzene (20 µL, 0.2 mmol) was added as the internal standard and an aliquot of the resulting solution was taken to be directly analyzed by ^{19}F NMR. The magnitude of the kinetic isotope effect, calculated plotting the kinetic profile of the two reactions (with cyclohexane and cyclohexane-d_{12}, respectively), was determined to be 1.8.

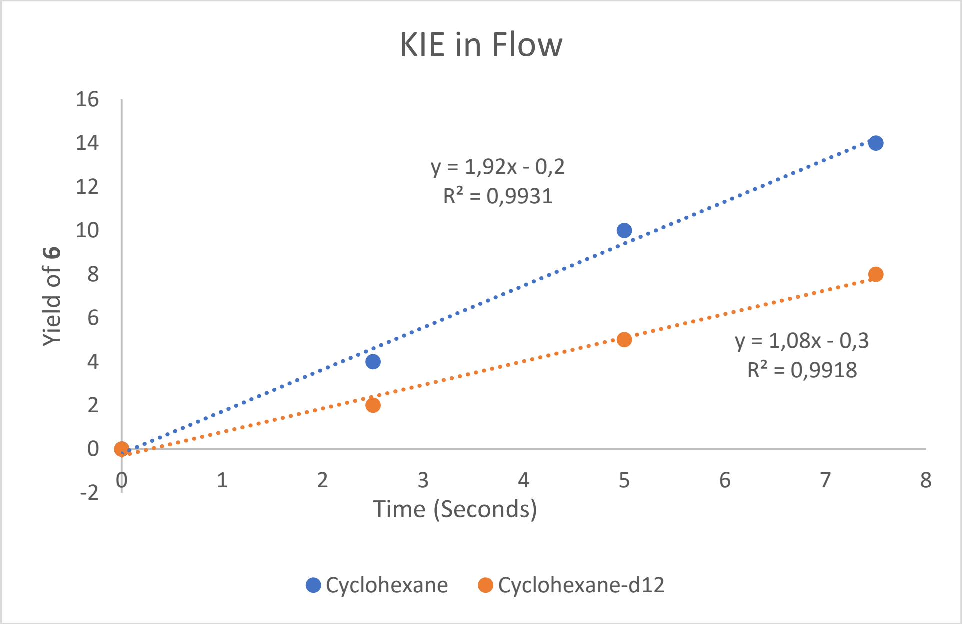

Figure S8: Kinetic isotope experiments performed on the reaction involving 4-fluoro benzoyl chloride and cyclohexane or cyclohexane-d_{12} in flow.

- Kinetic Isotope Effect in Batch

Two flame-dried argon-purged screw-capped vials, fitted with a rubber septum, containing the nickel complex **I** (9.7 mg, 10 mol%) and TBADT (19.9 mg, 3 mol%), were charged with 4-fluoro benzoyl chloride (24 µL, 0.2 mmol, 1 equiv.), 2,6-dimethylpyridine (26 µL, 1.1 equiv.) and cyclohexane or cyclohexane-d_{12} (108 µL, 5 equiv.) in CH_3CN (2 mL, 0.1 M). The vials were further flushed with argon, sealed with Parafilm, and then simultaneously placed 5 cm away from two 34W 390 nm Kessil Lamps with adequate fans to keep the reaction mixture at 35 °C. The reactions were stirred under irradiation for the time stated for each experiment. The crude mixture was then concentrated in vacuo, suspended in ethyl acetate and filtered on a pad of silica to ensure removal of the inorganic salts. 1,2-difluorobenzene (20 µL, 0.2 mmol) was added as the internal standard and an aliquot of the resulting solution was taken to be directly analyzed by ^{19}F NMR. This was performed with a reaction time of 5, 7.5 and 10 minutes. The magnitude of the kinetic isotope effect, calculated plotting the kinetic profile of the two reactions (with cyclohexane and cyclohexane-d_{12}, respectively), was determined to be 1.8.

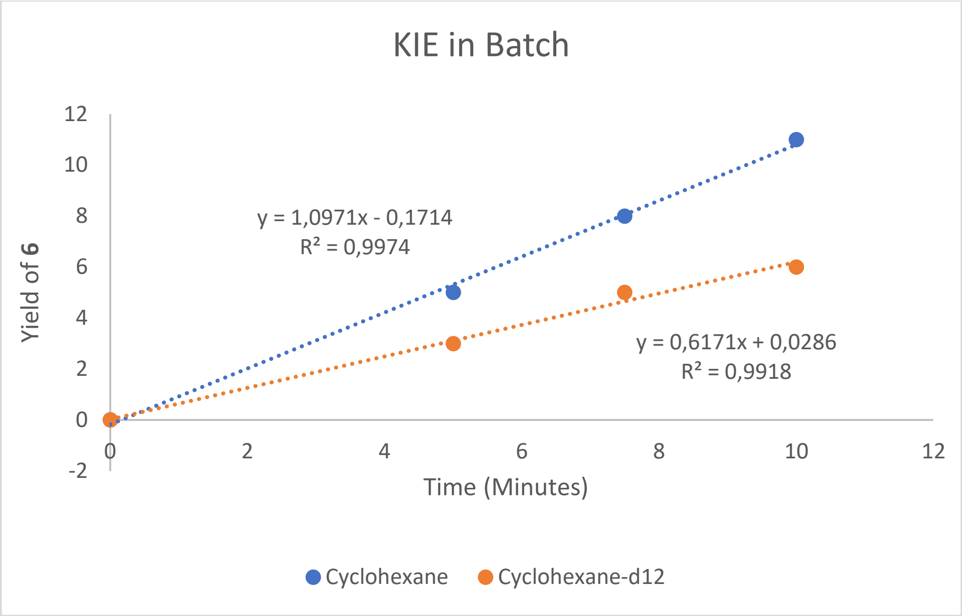

Figure S9: Kinetic isotope experiments performed on the reaction involving 4-fluoro benzoyl chloride and cyclohexane or cyclohexane-d_{12} in batch.

6. NMR Spectra

^{1}H NMR (400 MHz, $CDCl_3$)

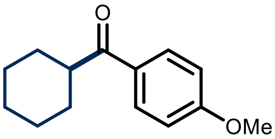

1

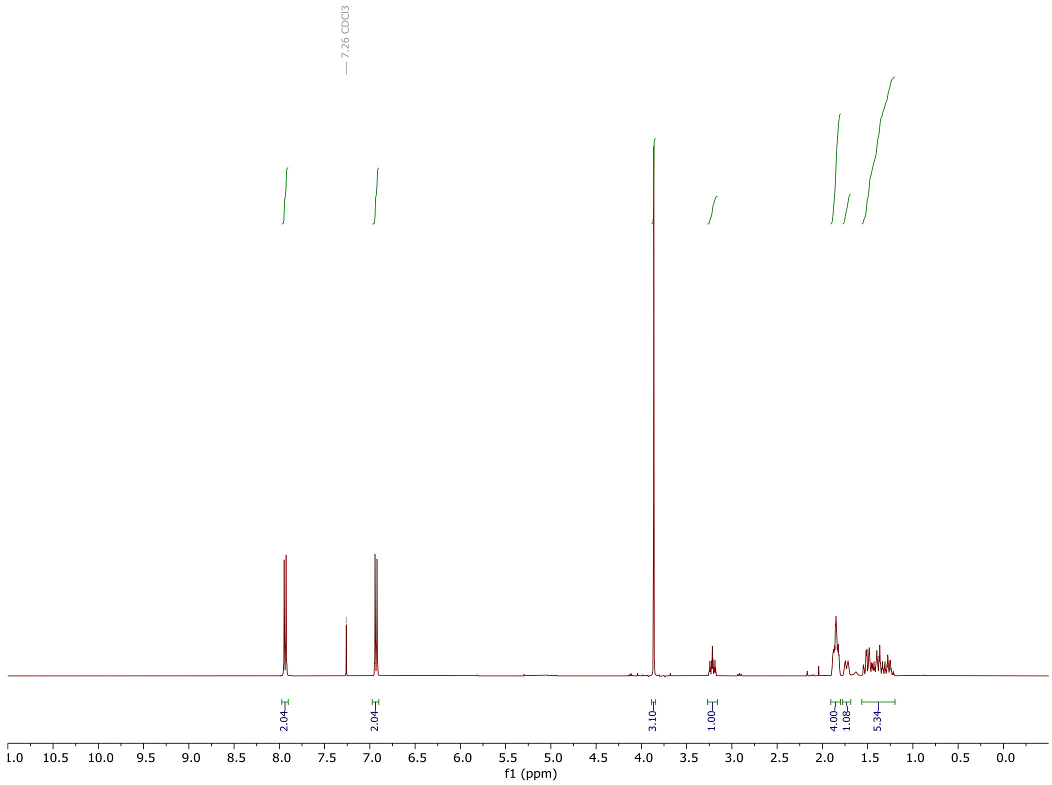

^{13}C NMR (101 MHz, $CDCl_3$)

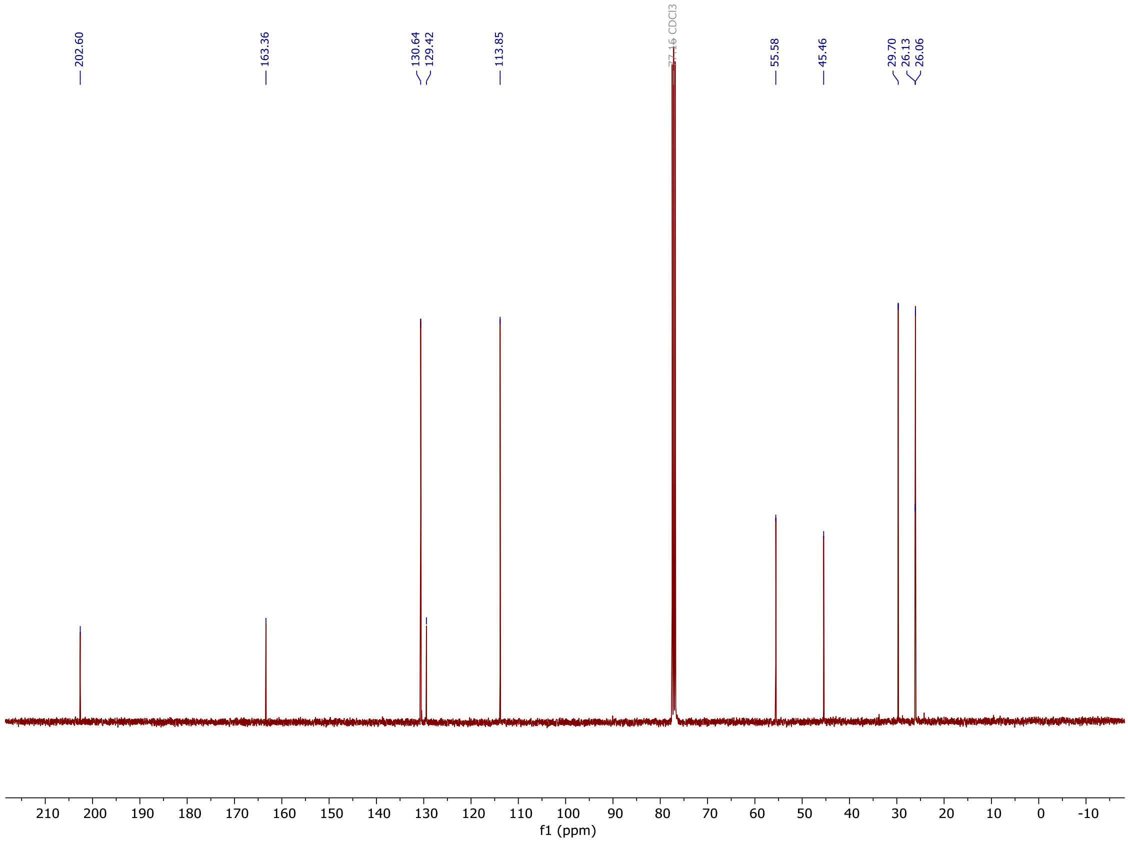

^{1}H NMR (400 MHz, $CDCl_3$)

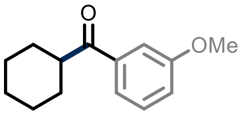

2

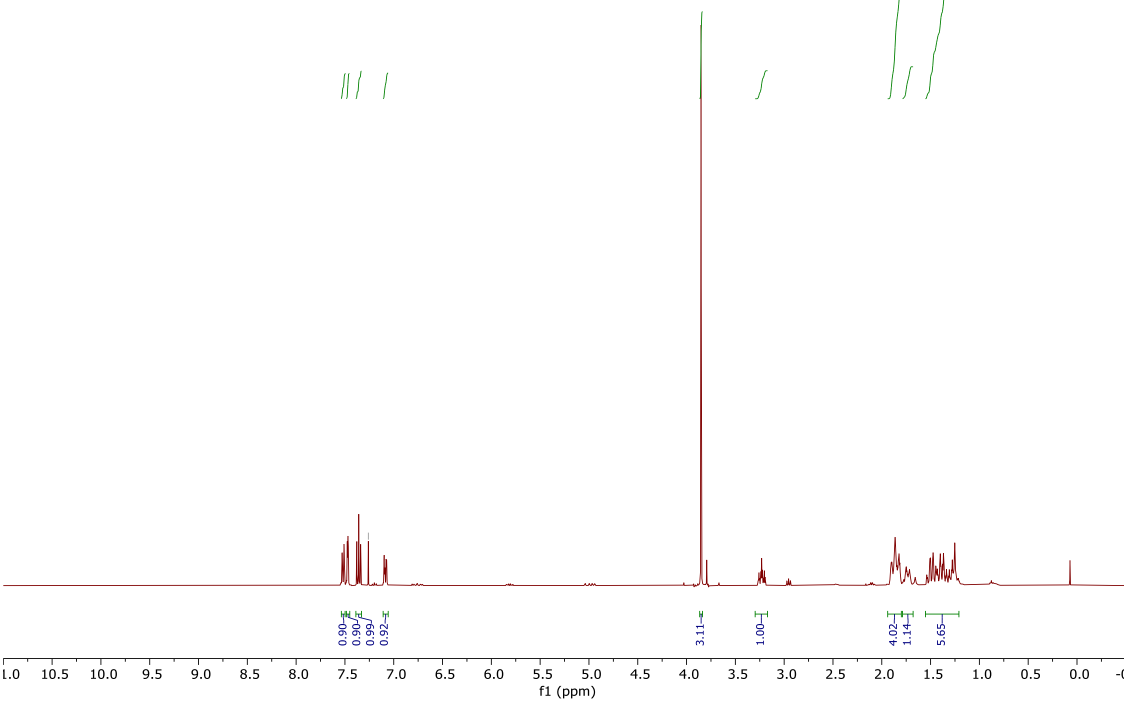

^{13}C NMR (101 MHz, $CDCl_3$)

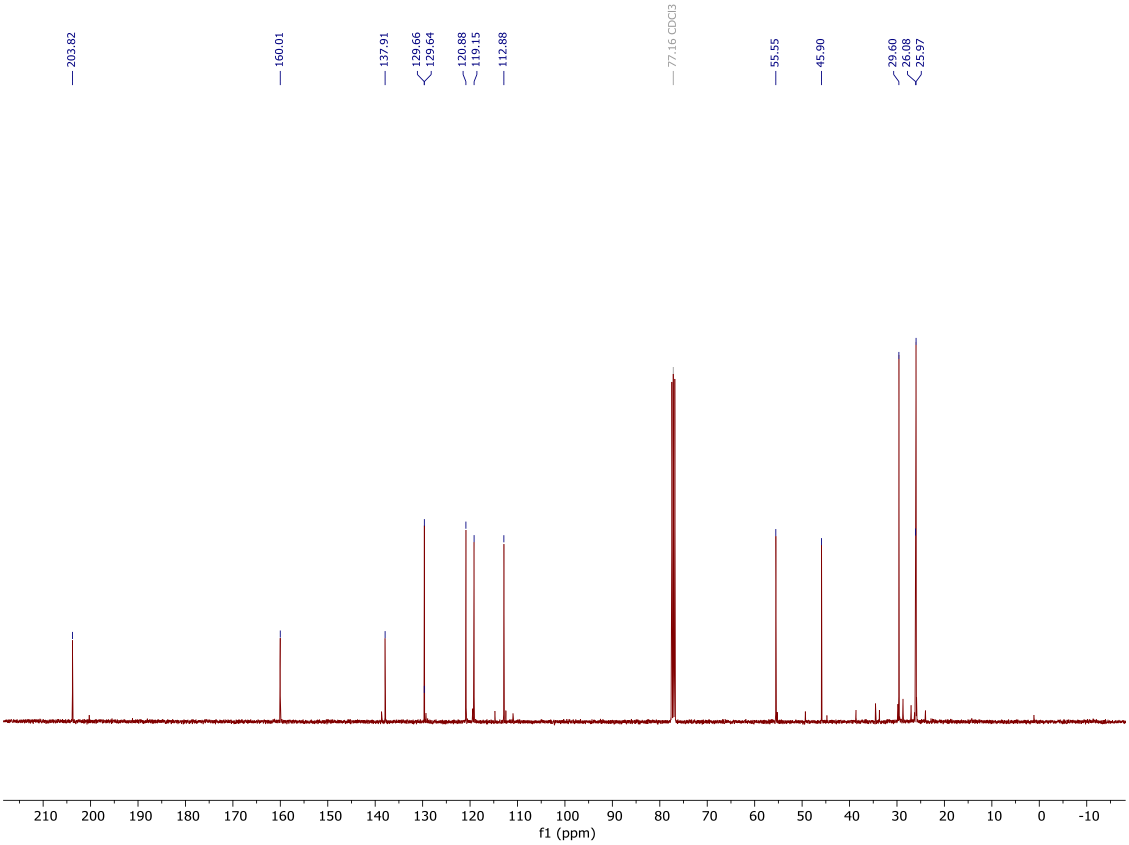

^{1}H NMR (300 MHz, $CDCl_3$)

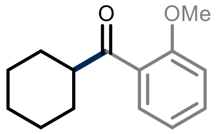

3

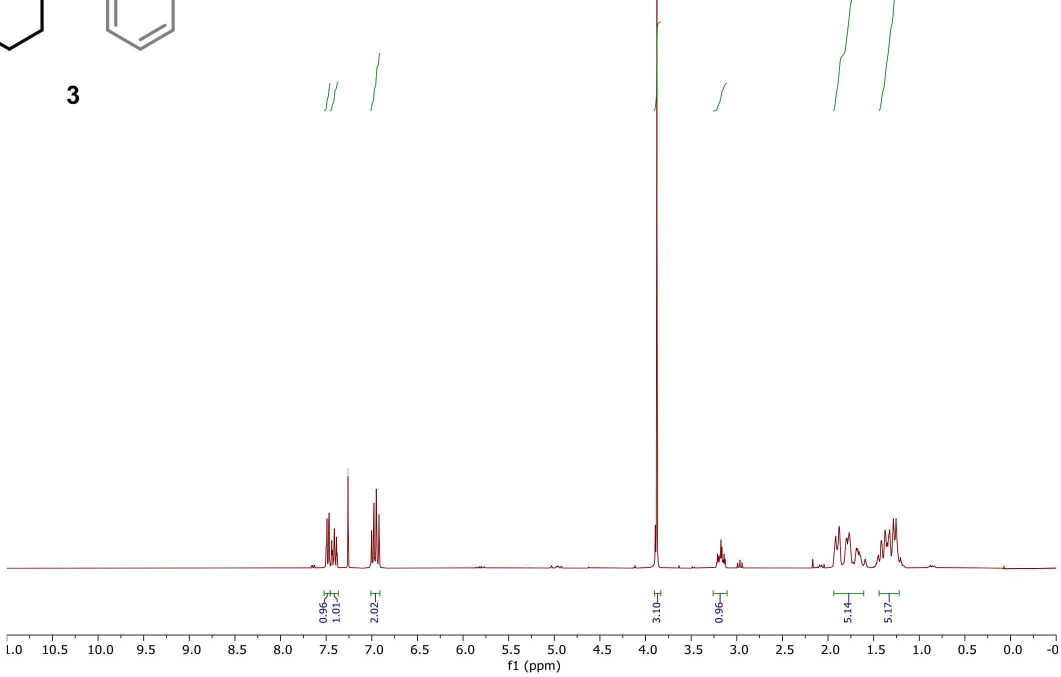

^{13}C NMR (101 MHz, $CDCl_3$)

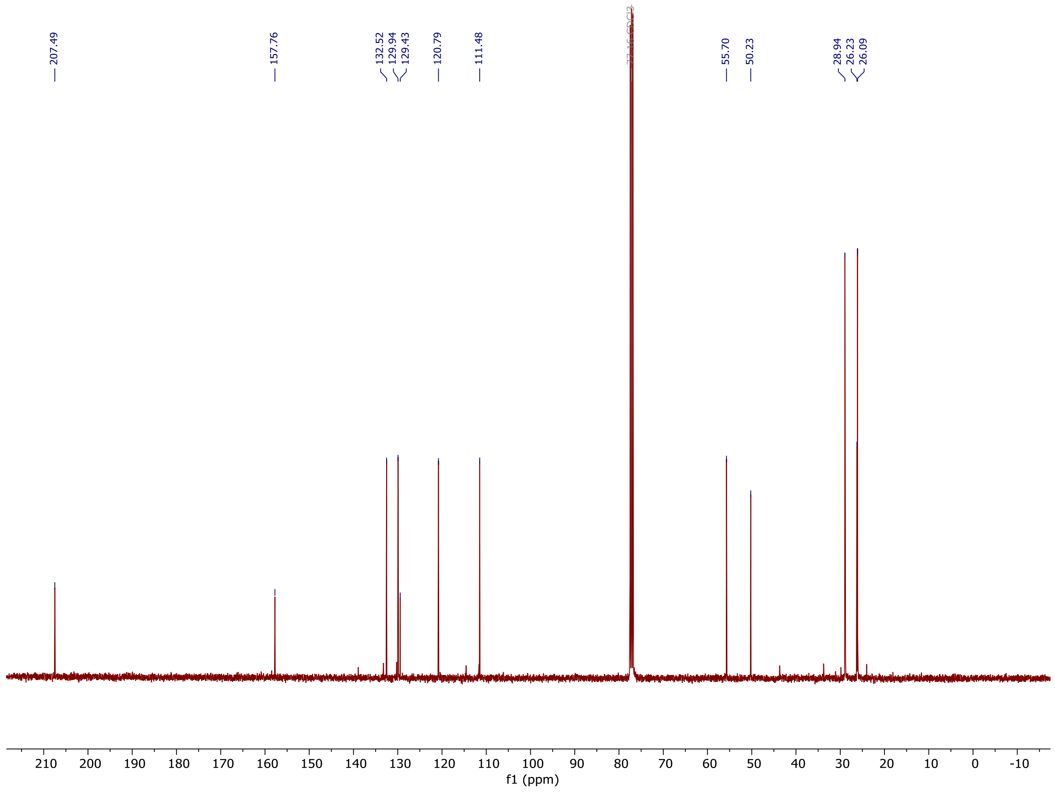

^{1}H NMR (400 MHz, $CDCl_3$)

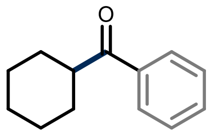

4

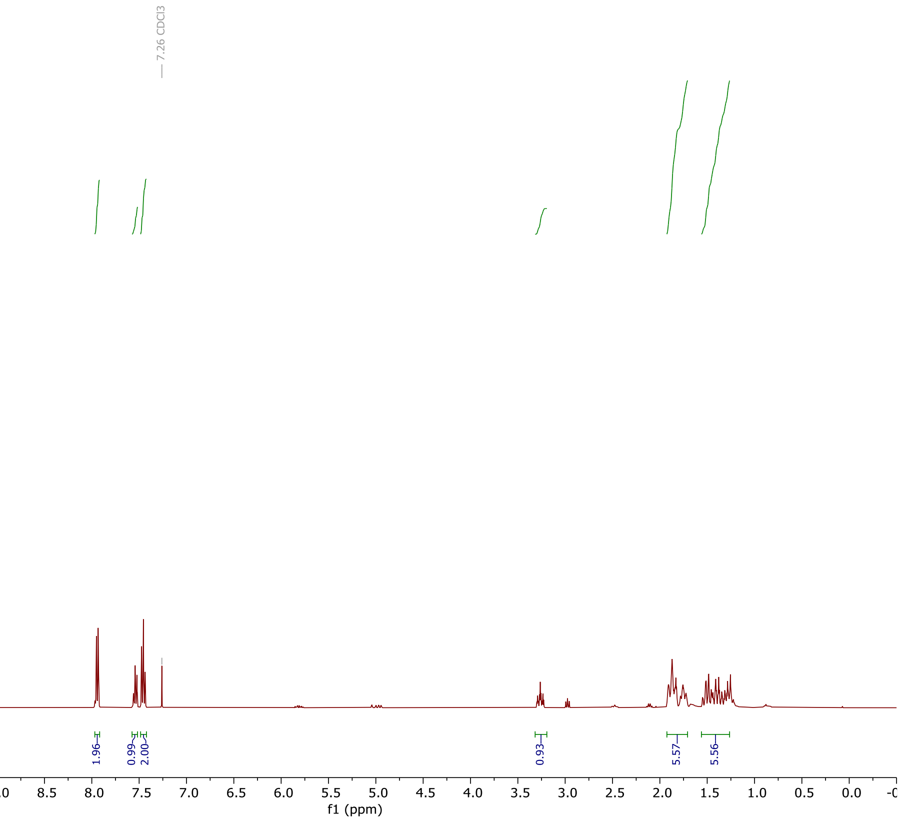

^{13}C NMR (101 MHz, $CDCl_3$)

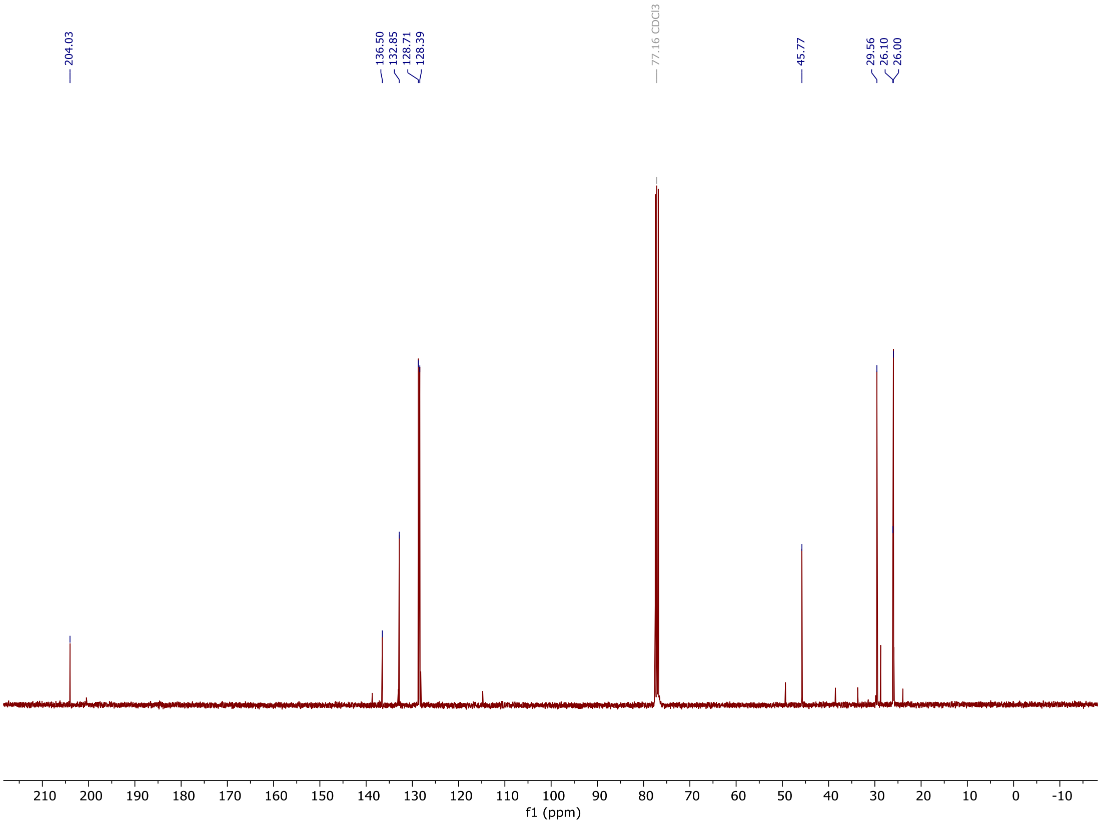

^{1}H NMR (400 MHz, $CDCl_3$)

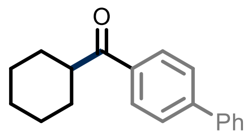

5

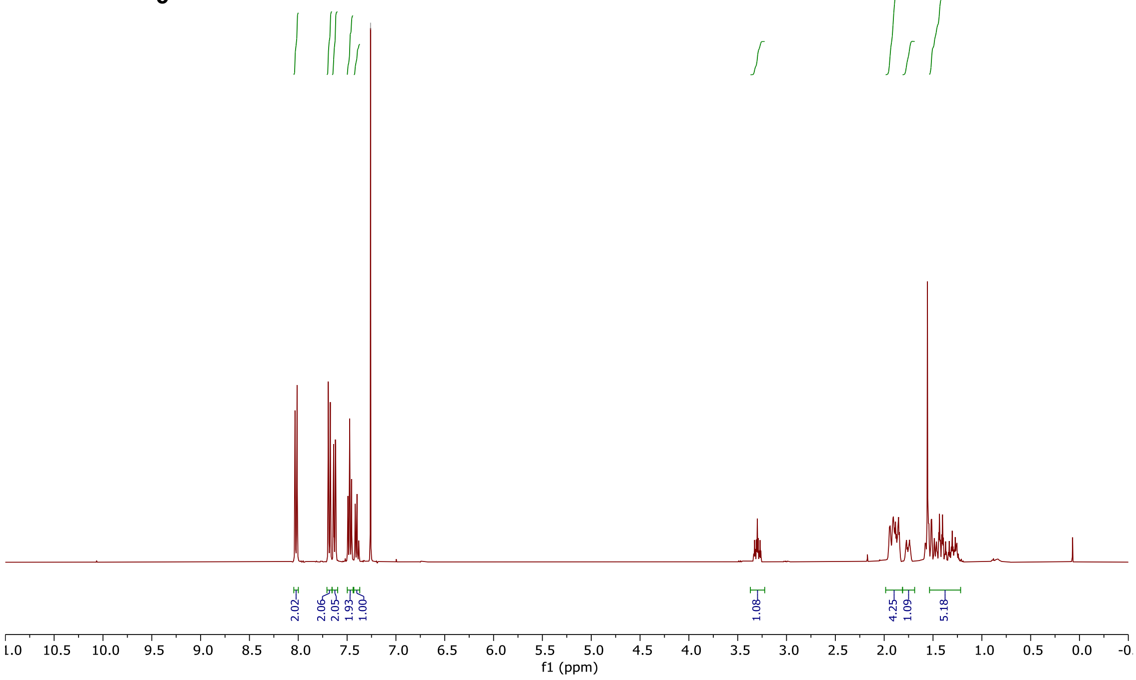

^{13}C NMR (101 MHz, $CDCl_3$)

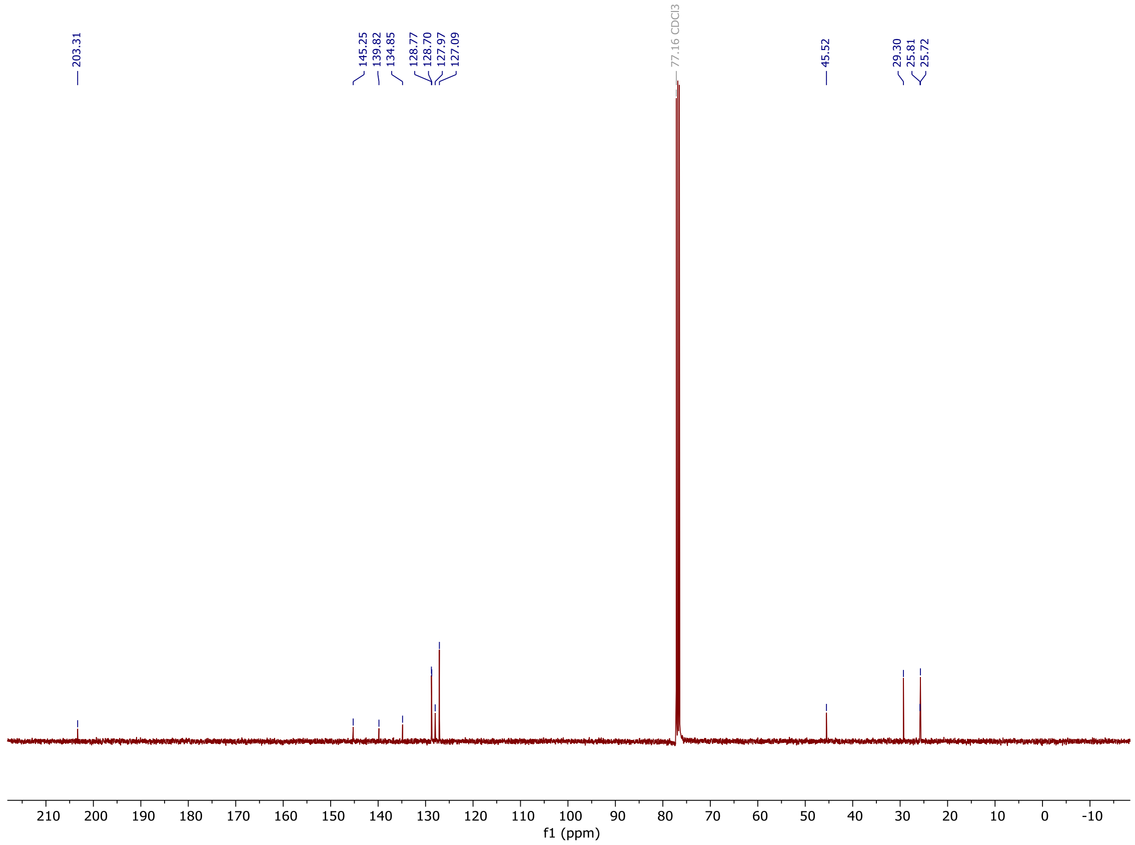

^{1}H NMR (400 MHz, $CDCl_3$)

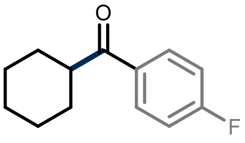

6

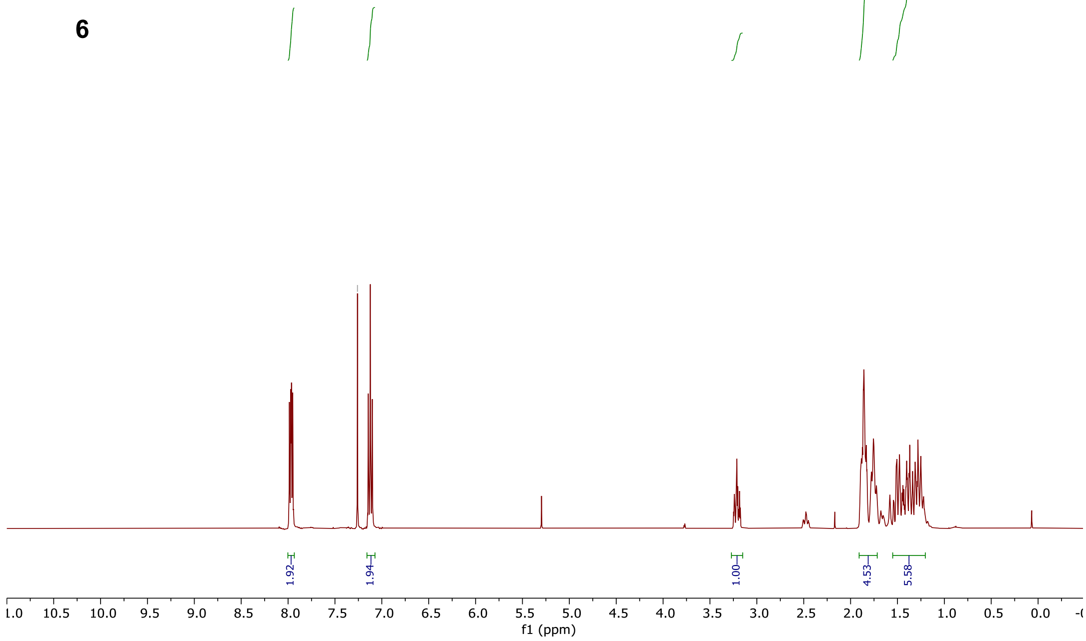

^{13}C NMR (101 MHz, $CDCl_3$)

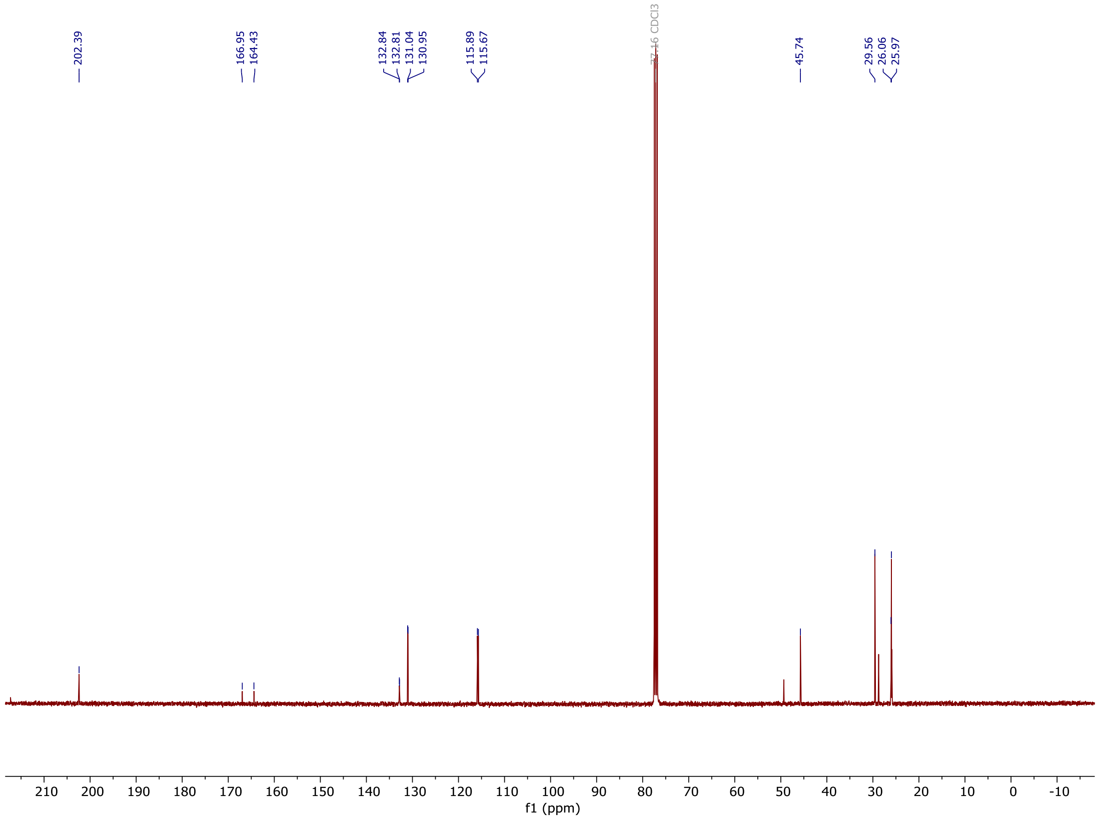

^{19}F NMR (376 MHz, $CDCl_3$)

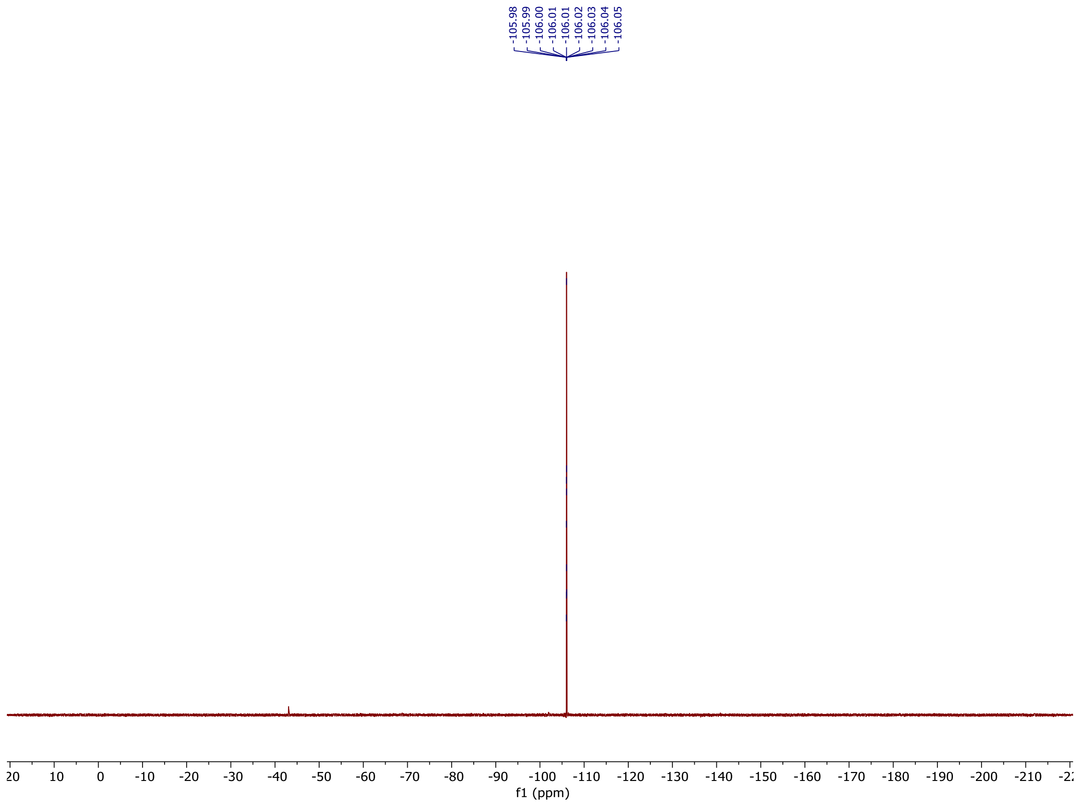

^{1}H NMR (400 MHz, $CDCl_3$)

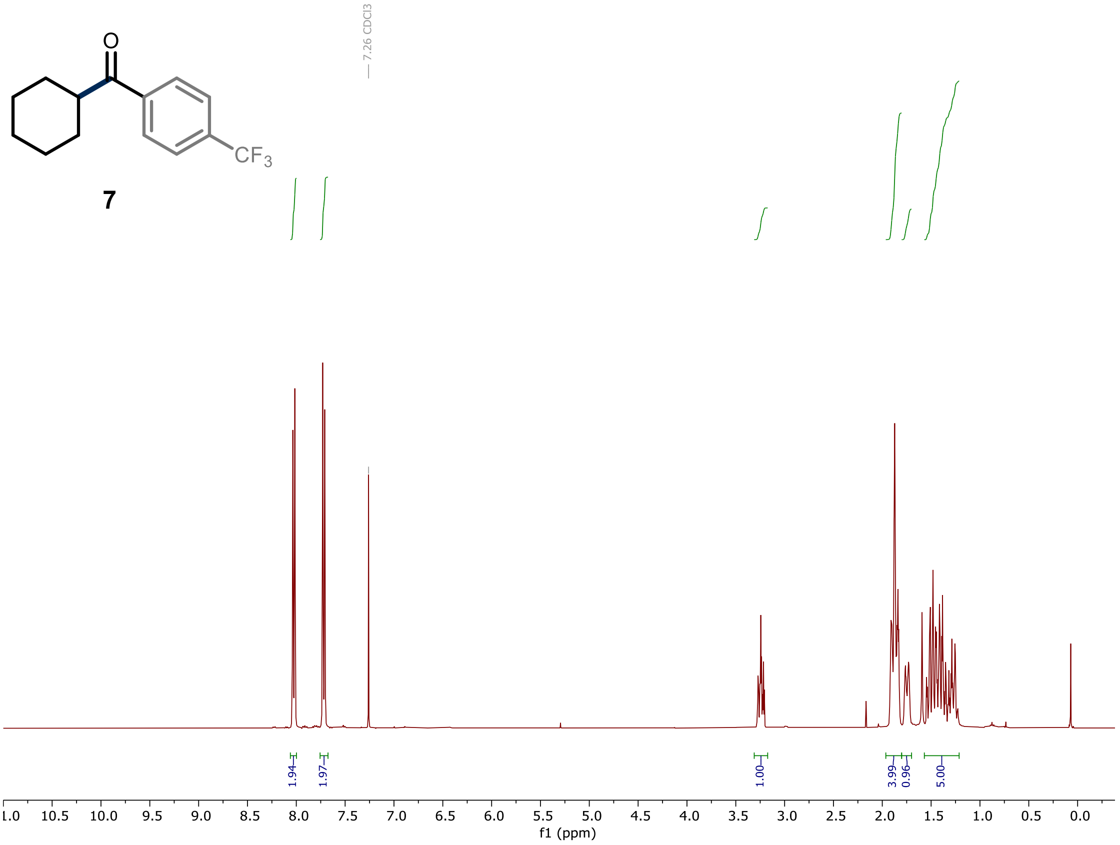

^{13}C NMR (101 MHz, $CDCl_3$)

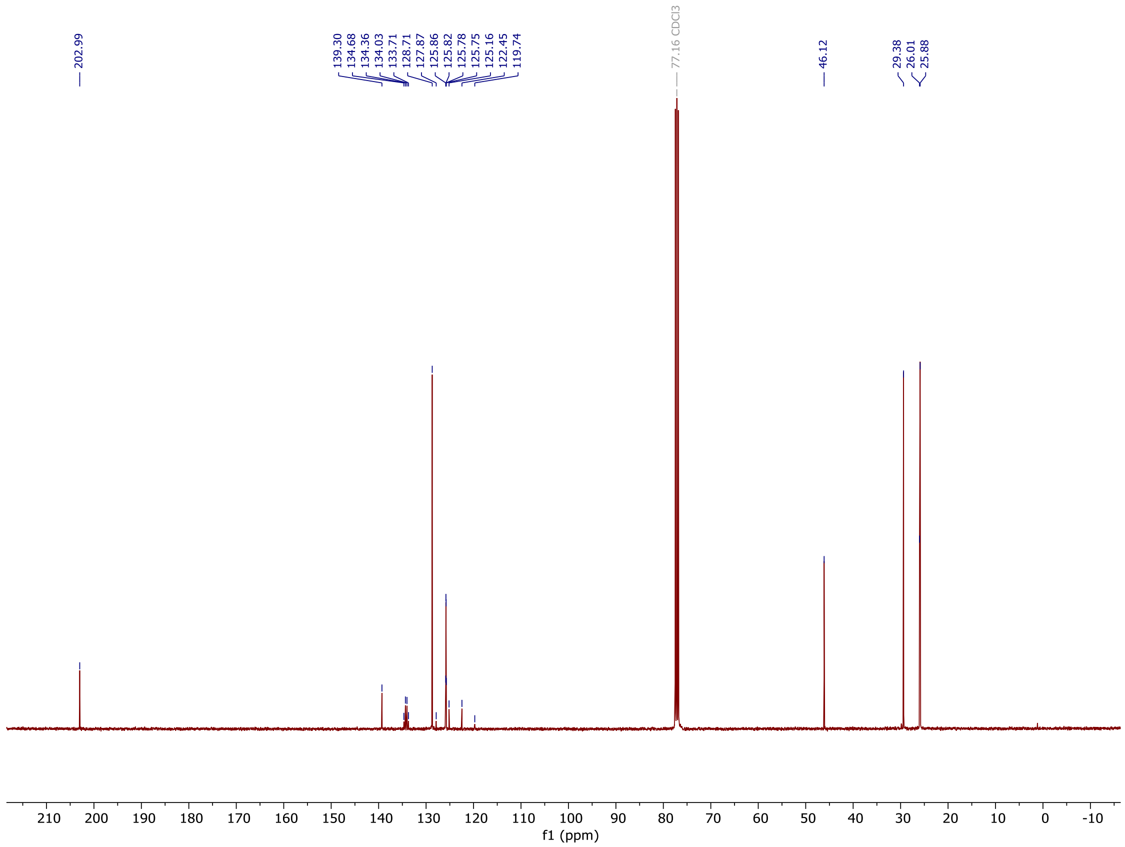

^{19}F NMR (376 MHz, $CDCl_3$)

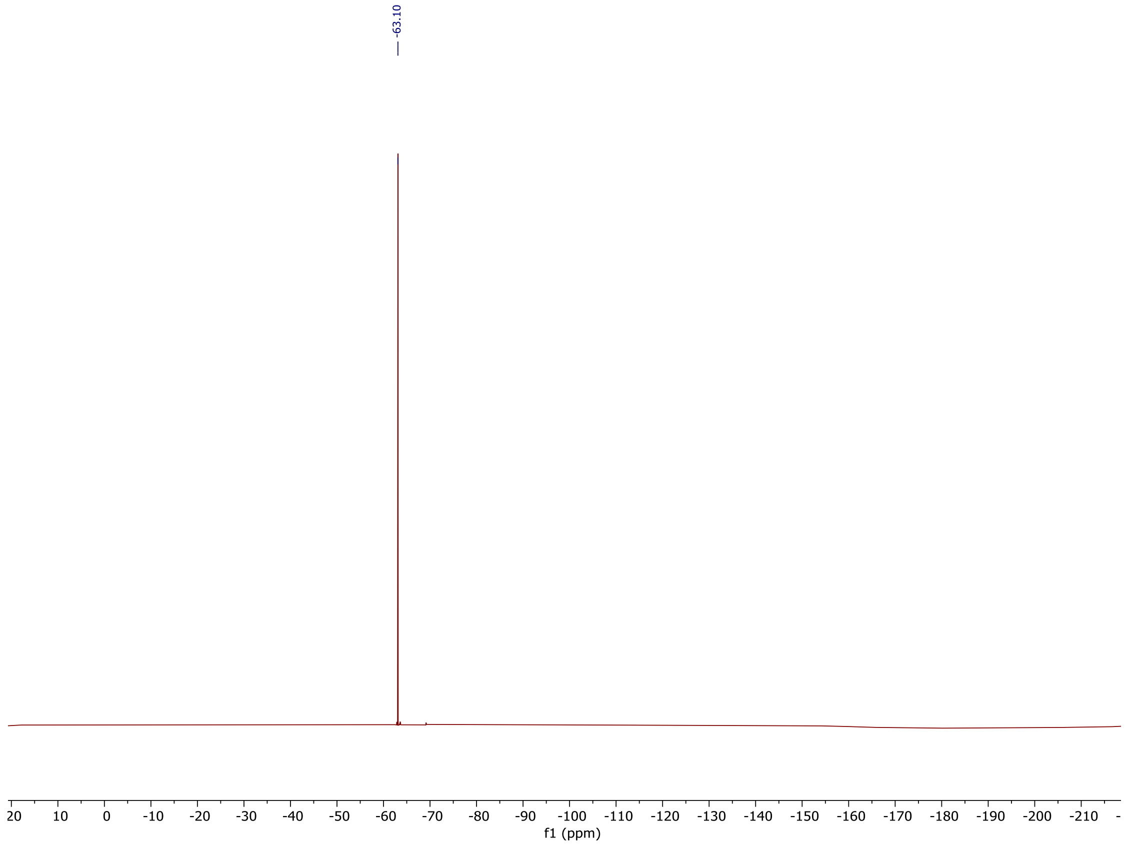

^{1}H NMR (400 MHz, $CDCl_3$)

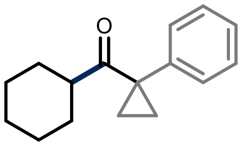

8

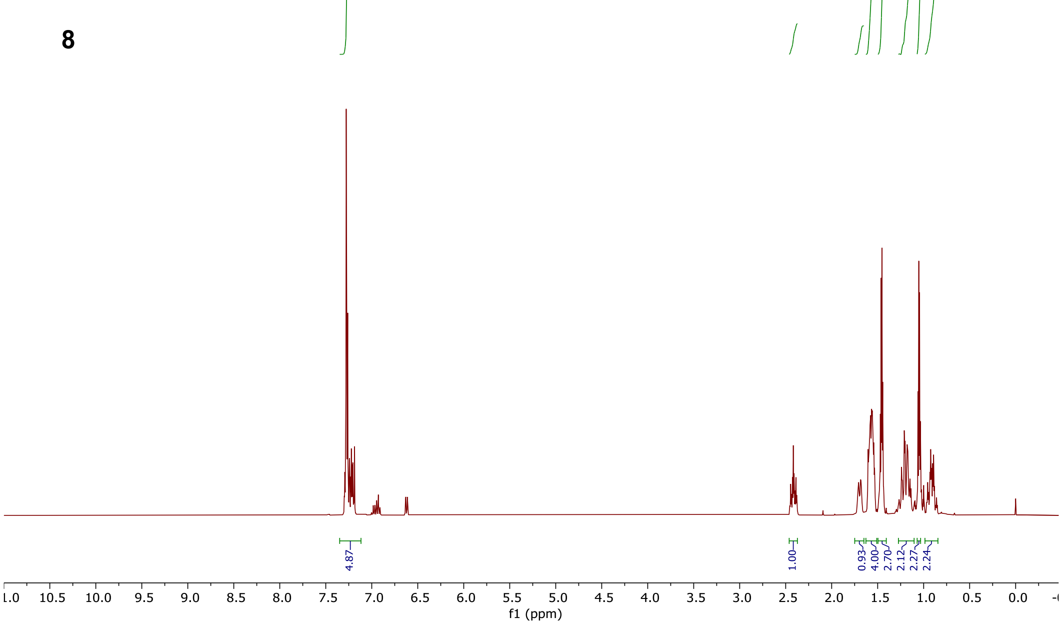

^{13}C NMR (101 MHz, $CDCl_3$)

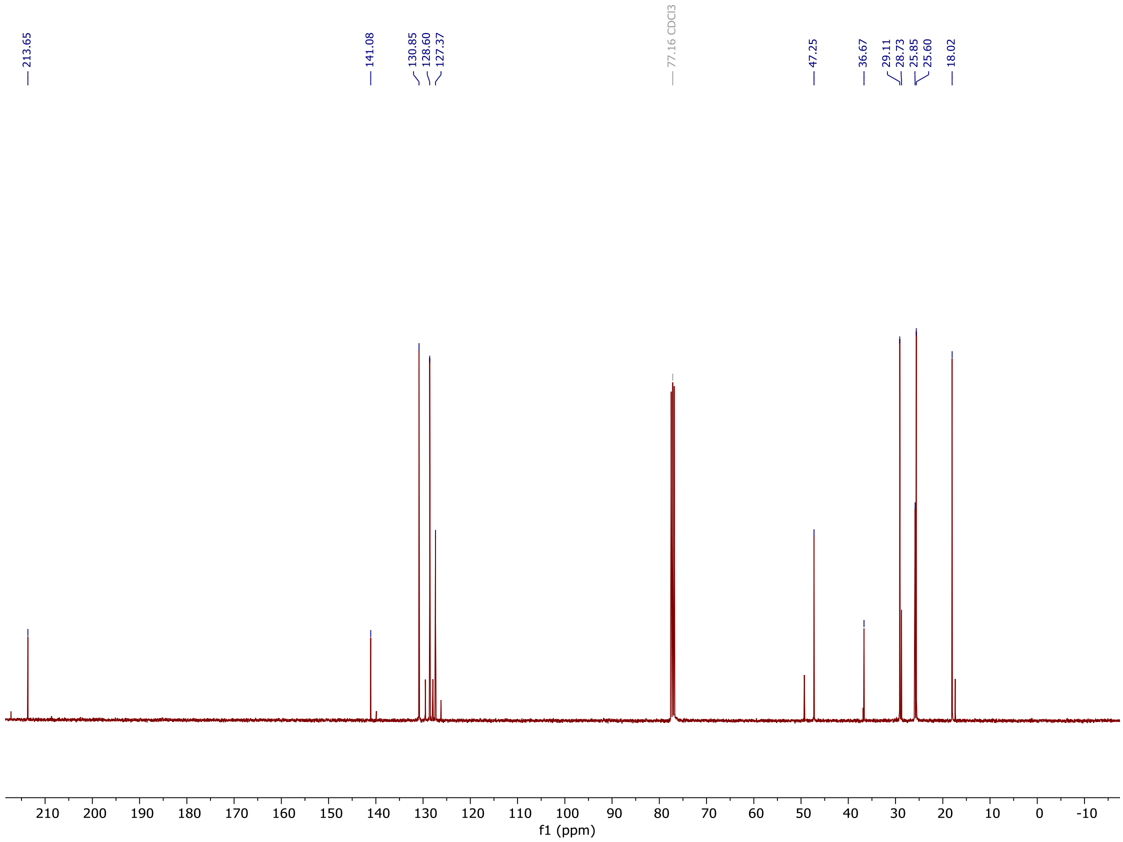

^{1}H NMR (400 MHz, $CDCl_3$)

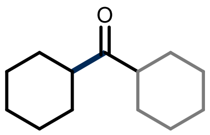

9

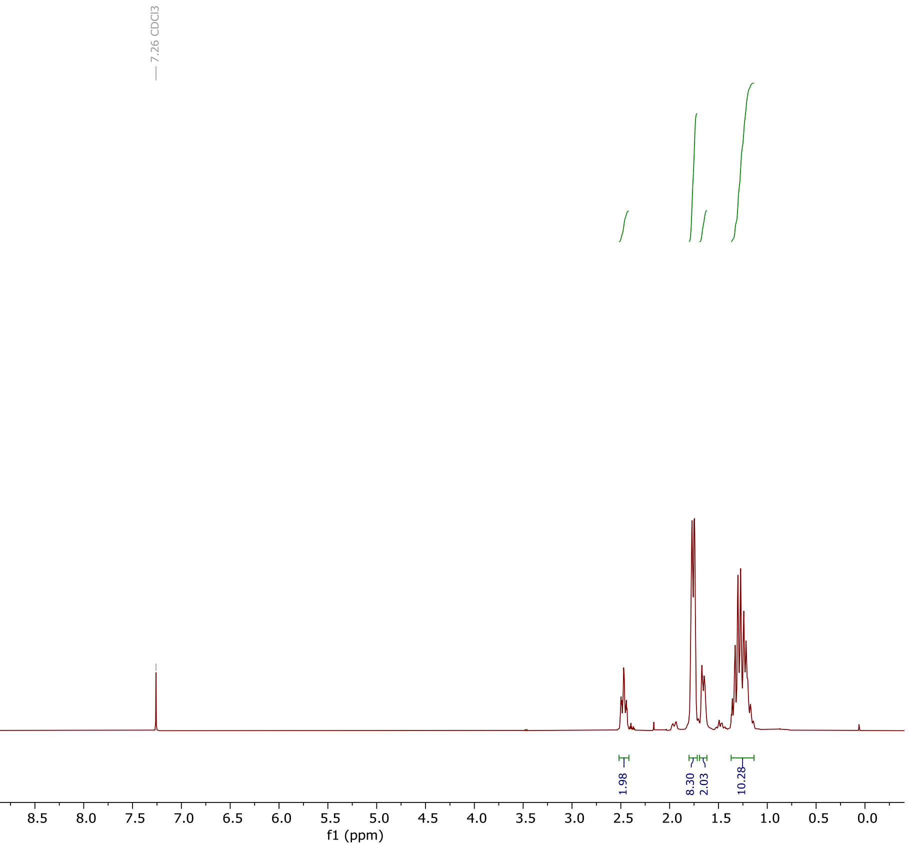

^{13}C NMR (101 MHz, $CDCl_3$)

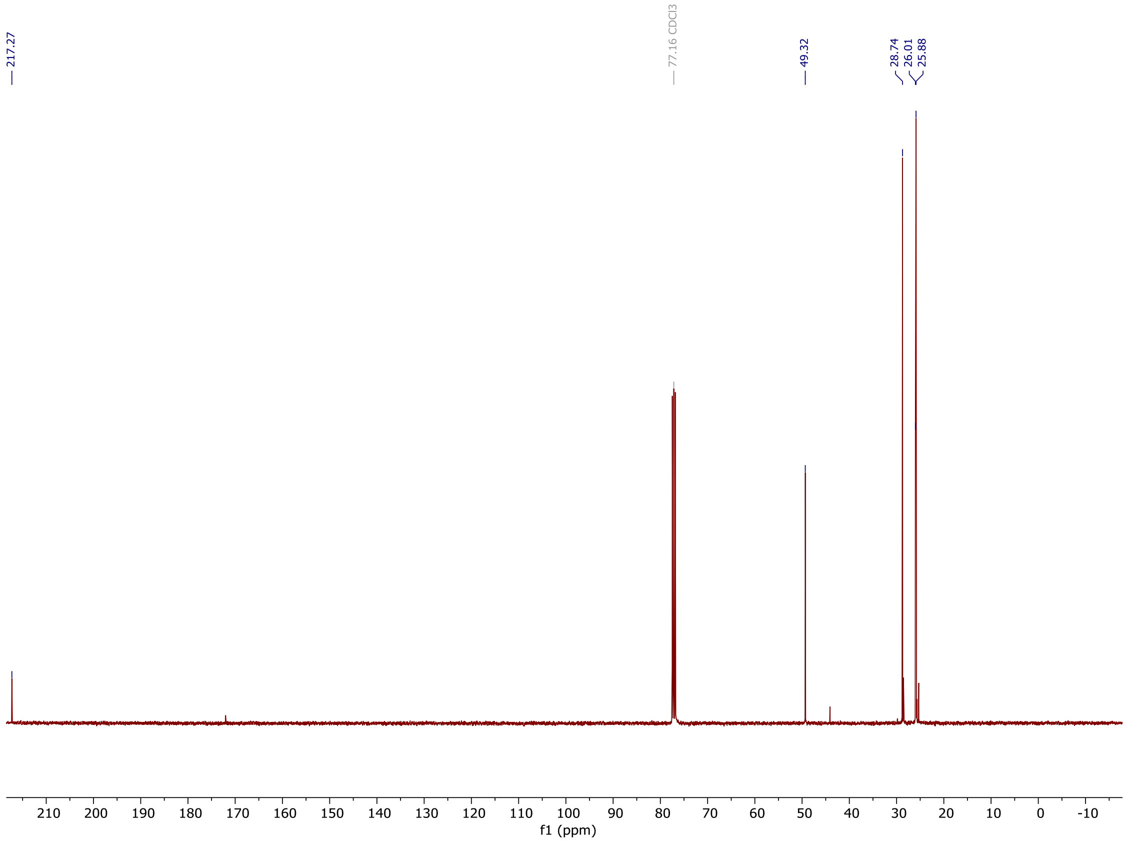

^{1}H NMR (400 MHz, $CDCl_3$)

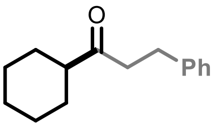

10

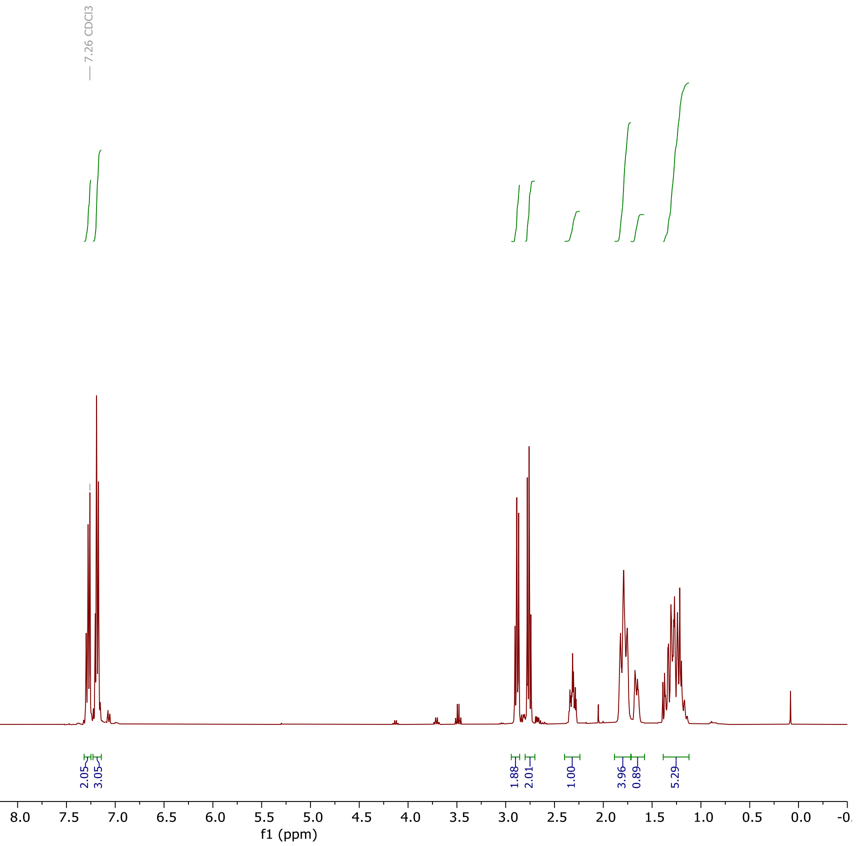

^{13}C NMR (101 MHz, $CDCl_3$)

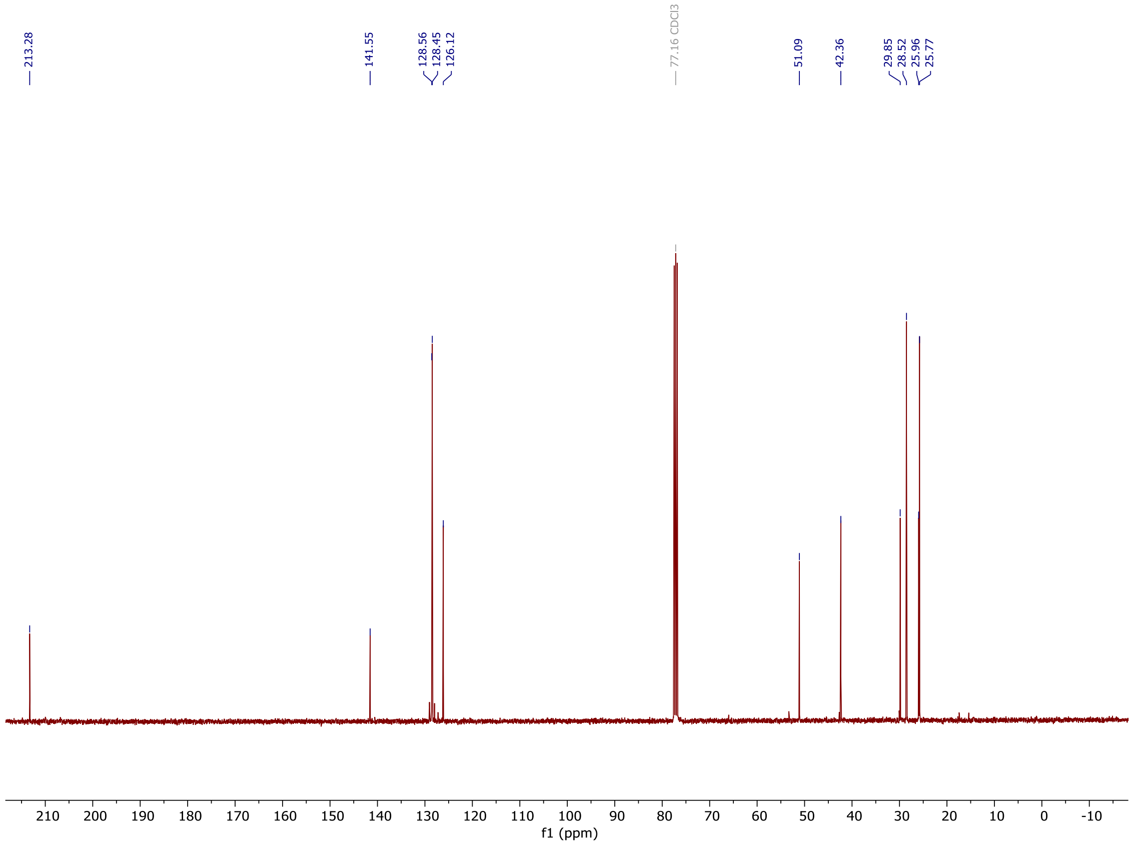

^{1}H NMR (400 MHz, $CDCl_3$)

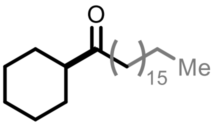

11

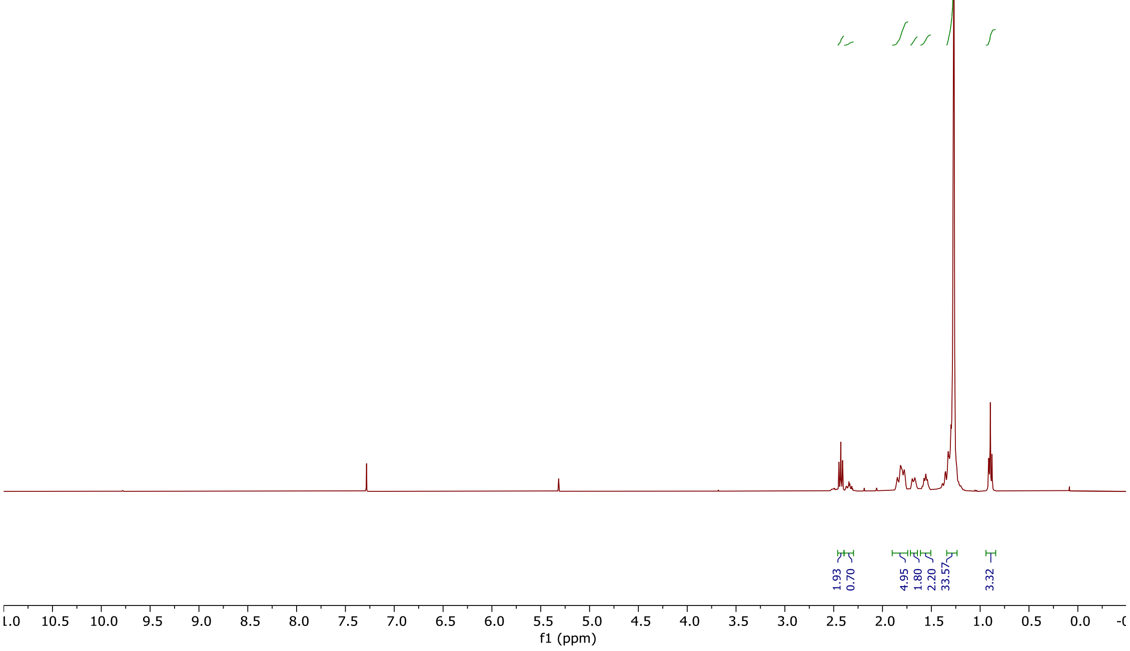

^{13}C NMR (101 MHz, $CDCl_3$)

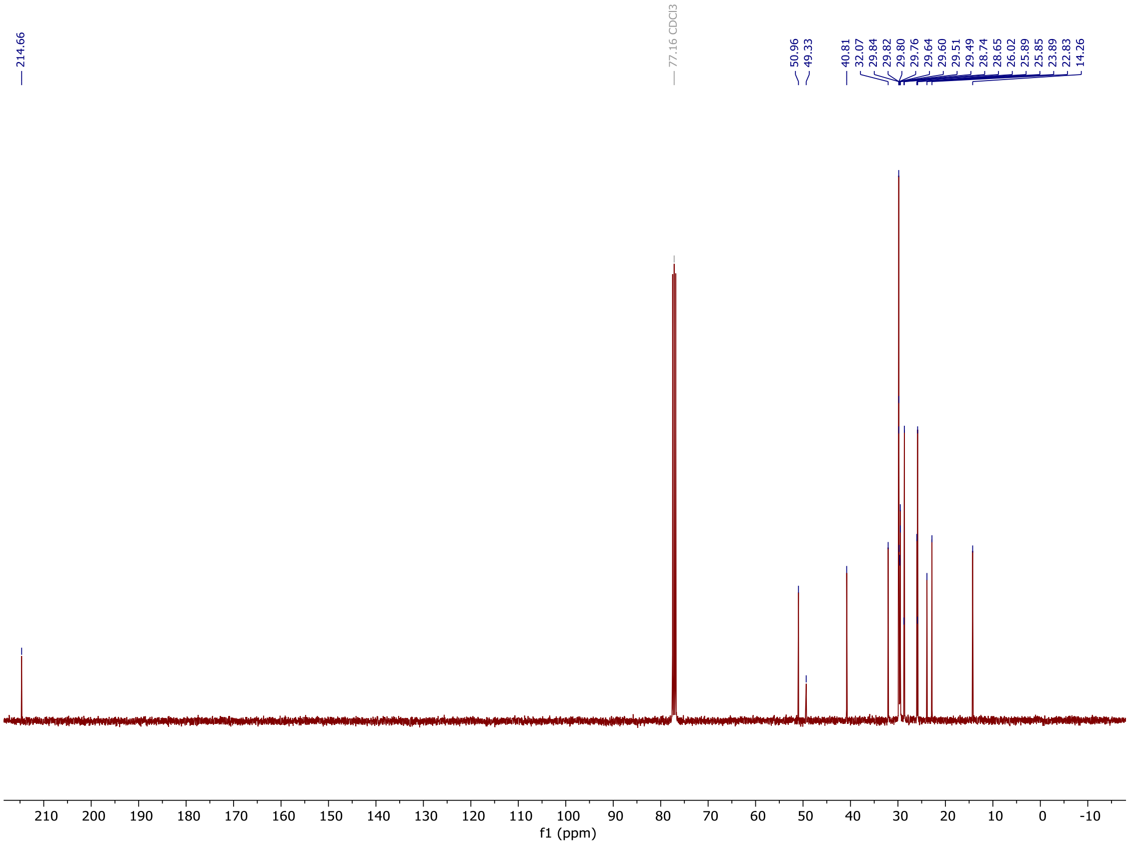

^{1}H NMR (400 MHz, $CDCl_3$)

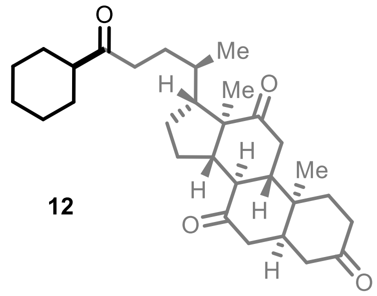

12

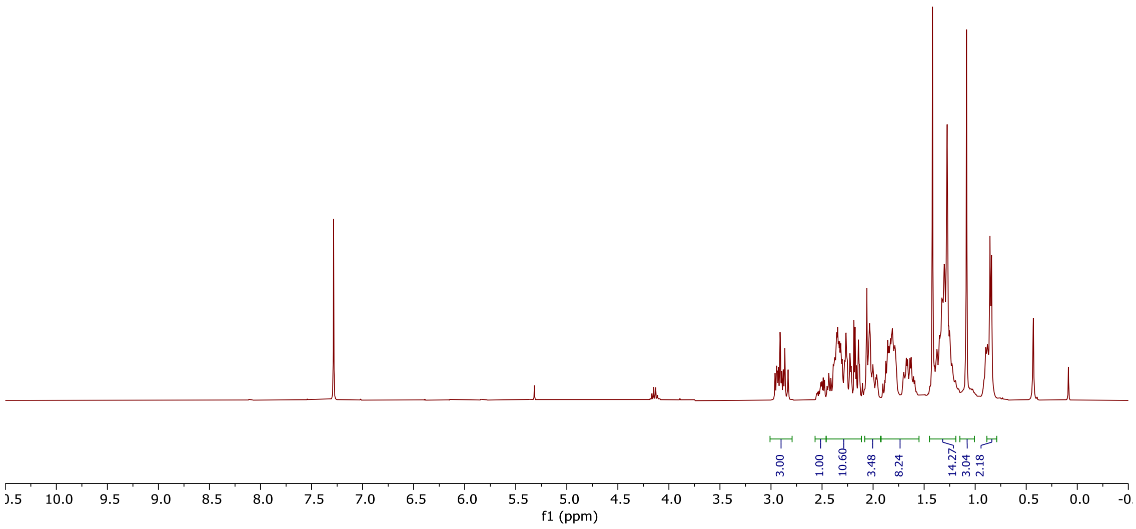

^{13}C NMR (101 MHz, $CDCl_3$)

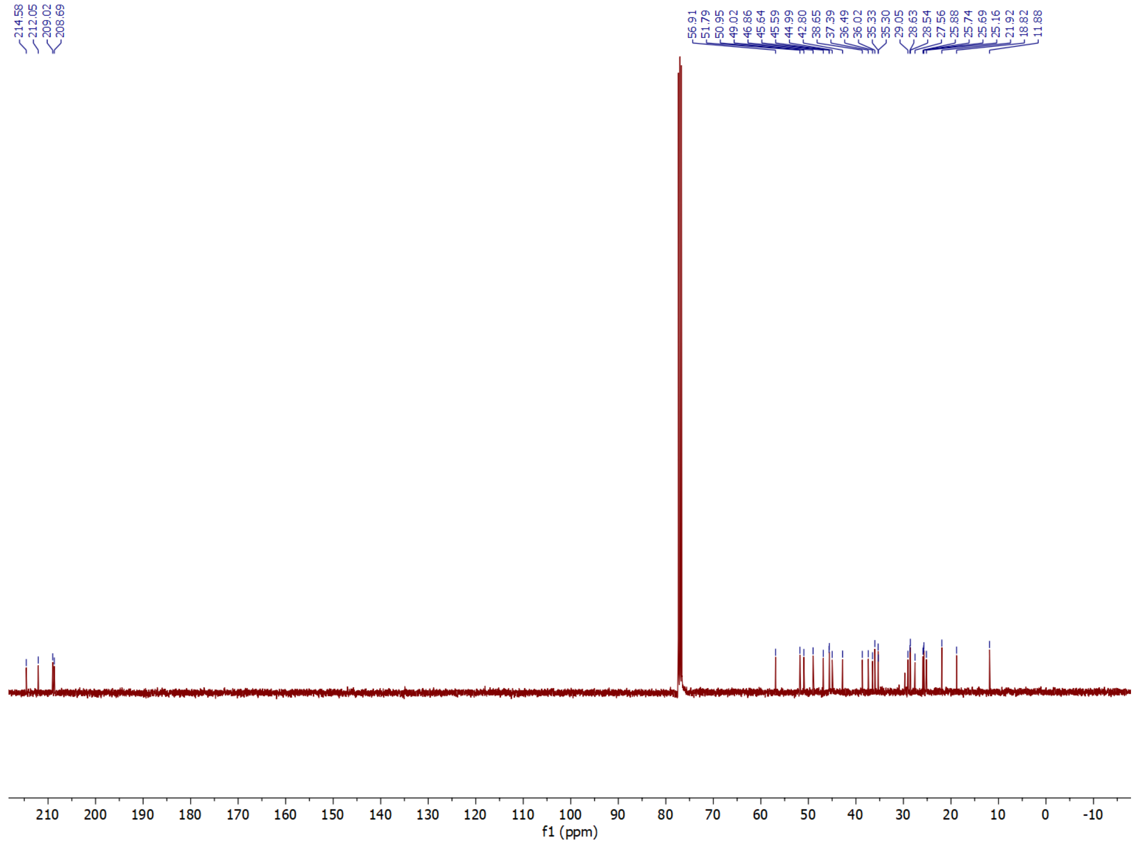

^{1}H NMR (400 MHz, $CDCl_3$)

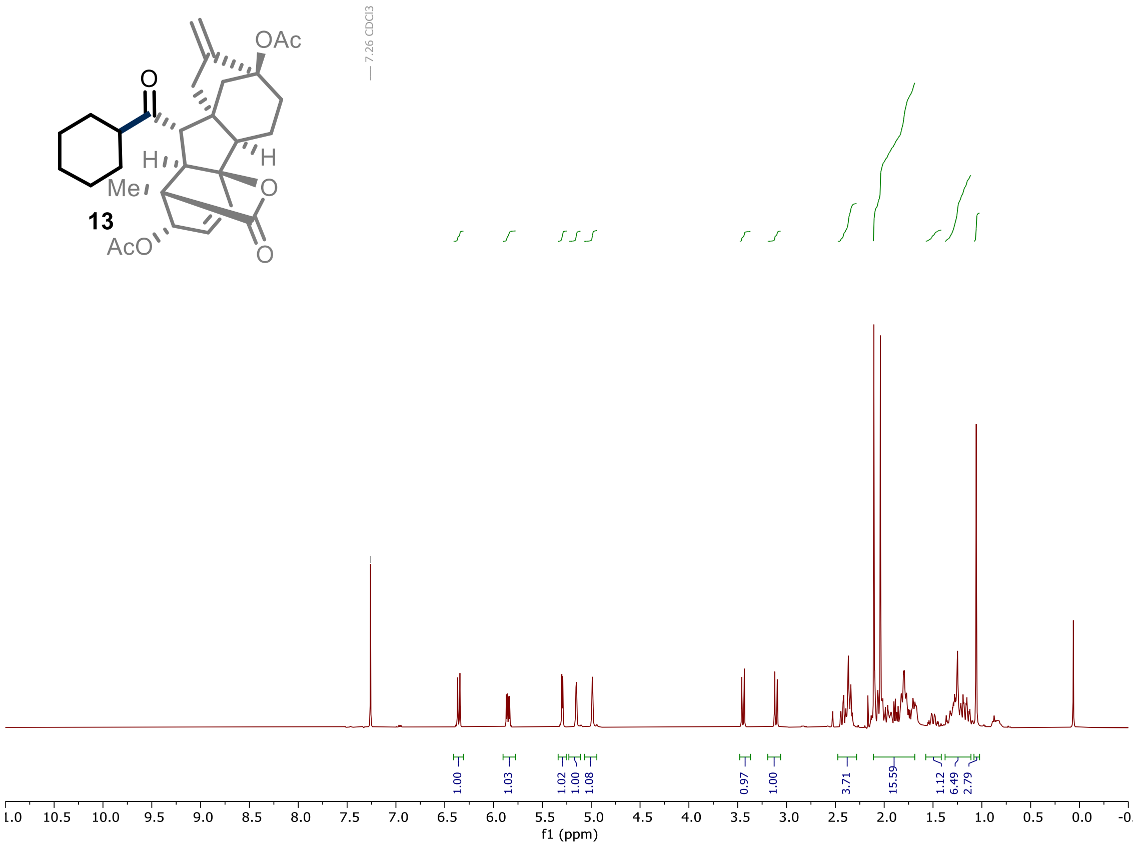

13

^{13}C NMR (101 MHz, $CDCl_3$)

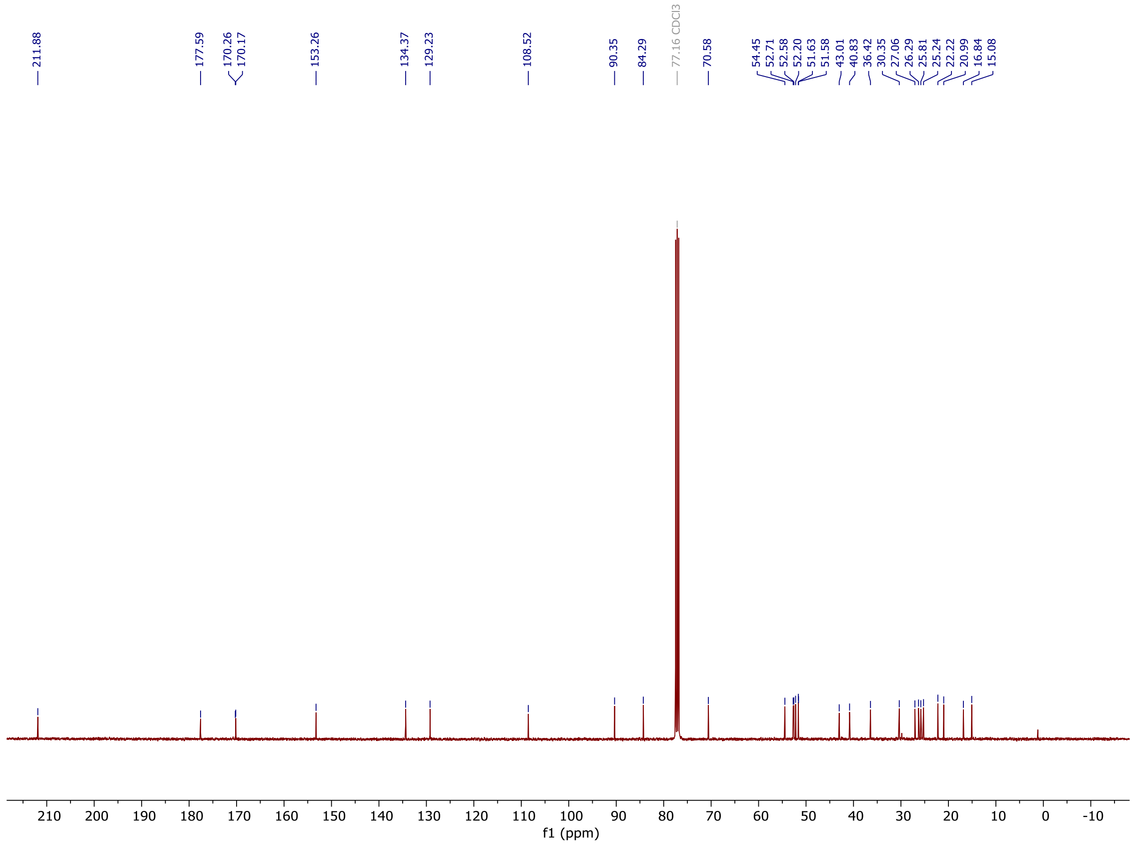

^{1}H NMR (400 MHz, $CDCl_3$)

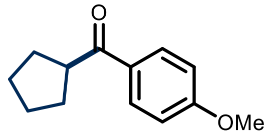

14

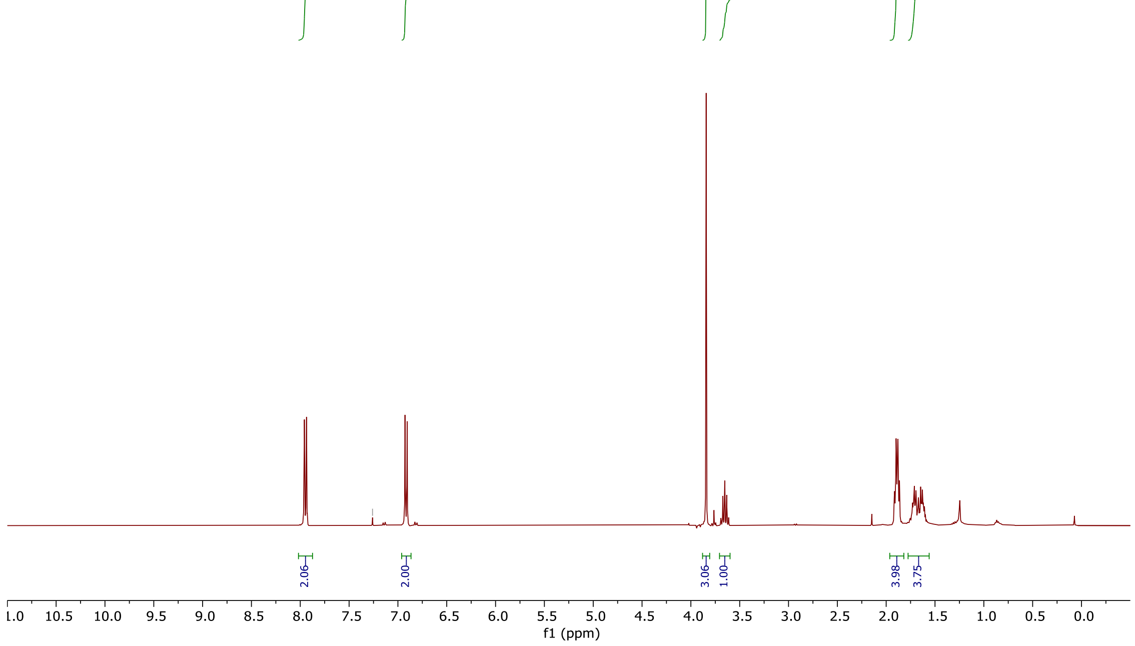

^{13}C NMR (101 MHz, $CDCl_3$)

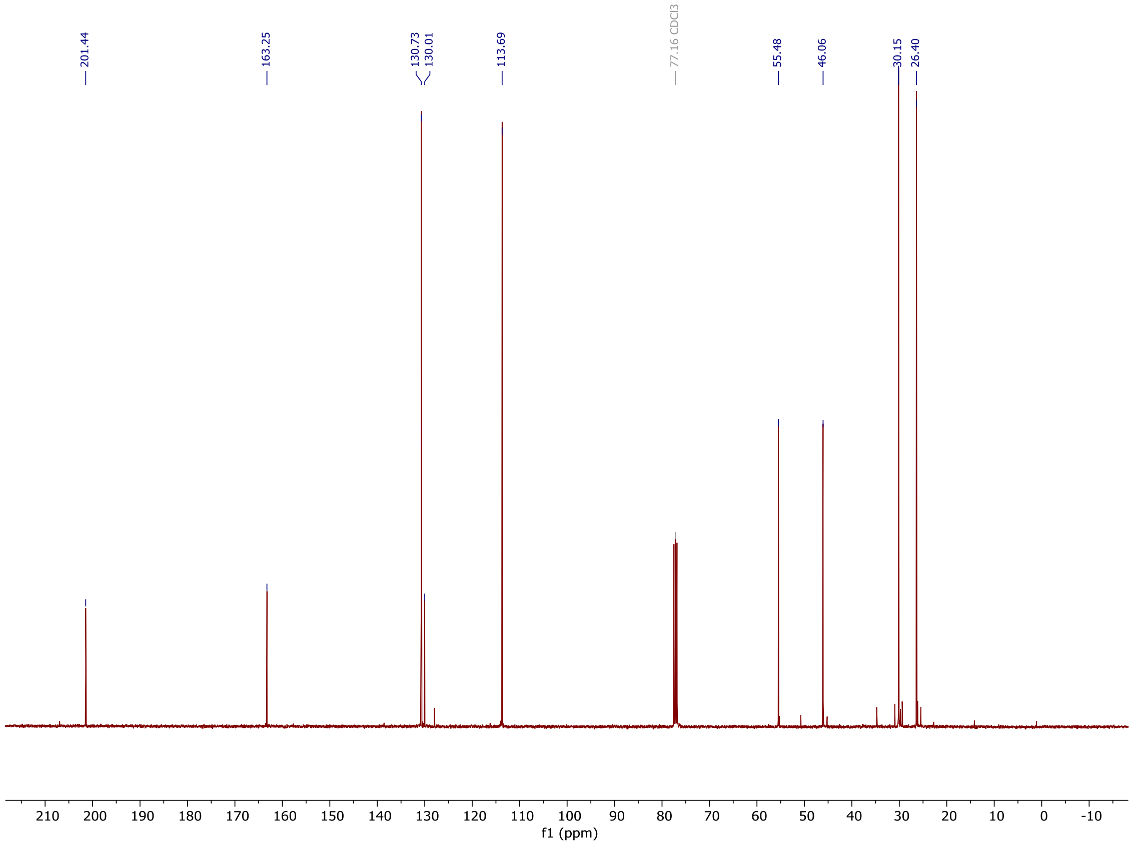

^{1}H NMR (400 MHz, $CDCl_3$)

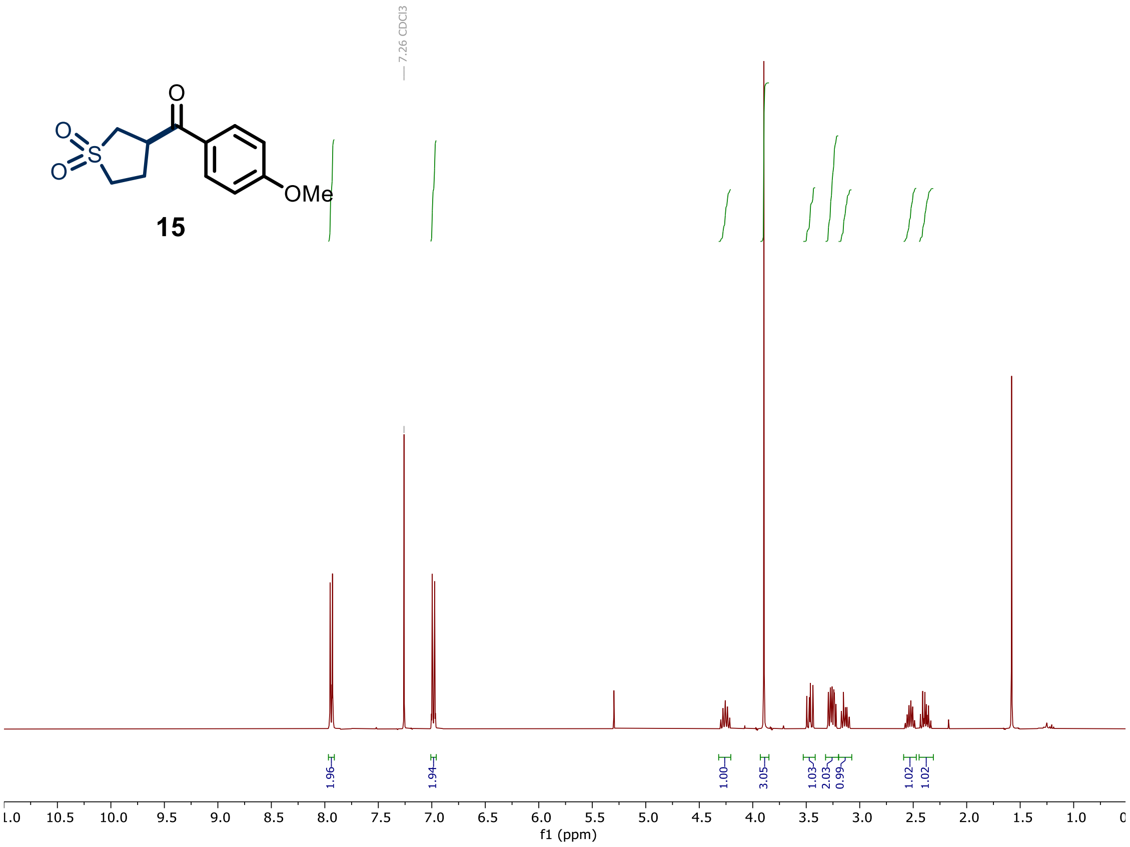

^{13}C NMR (101 MHz, $CDCl_3$)

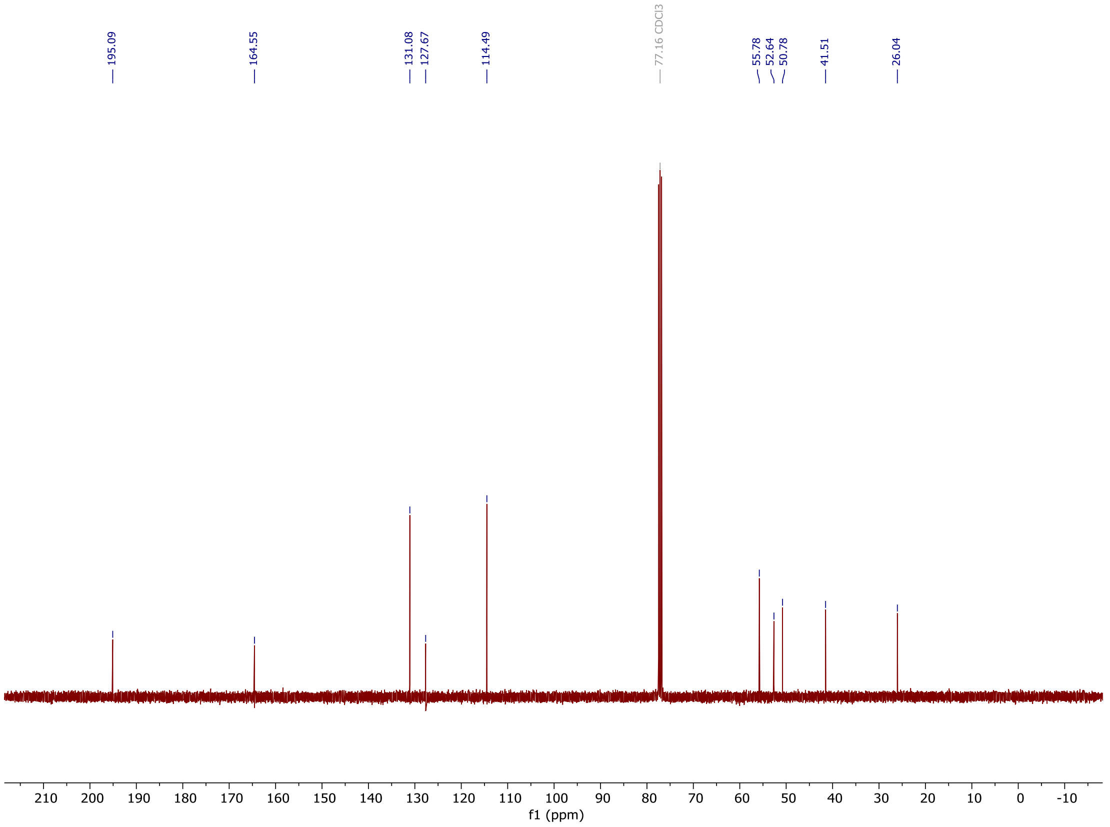

^{1}H NMR (400 MHz, $CDCl_3$)

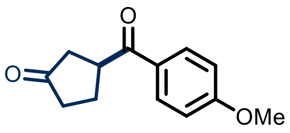

16

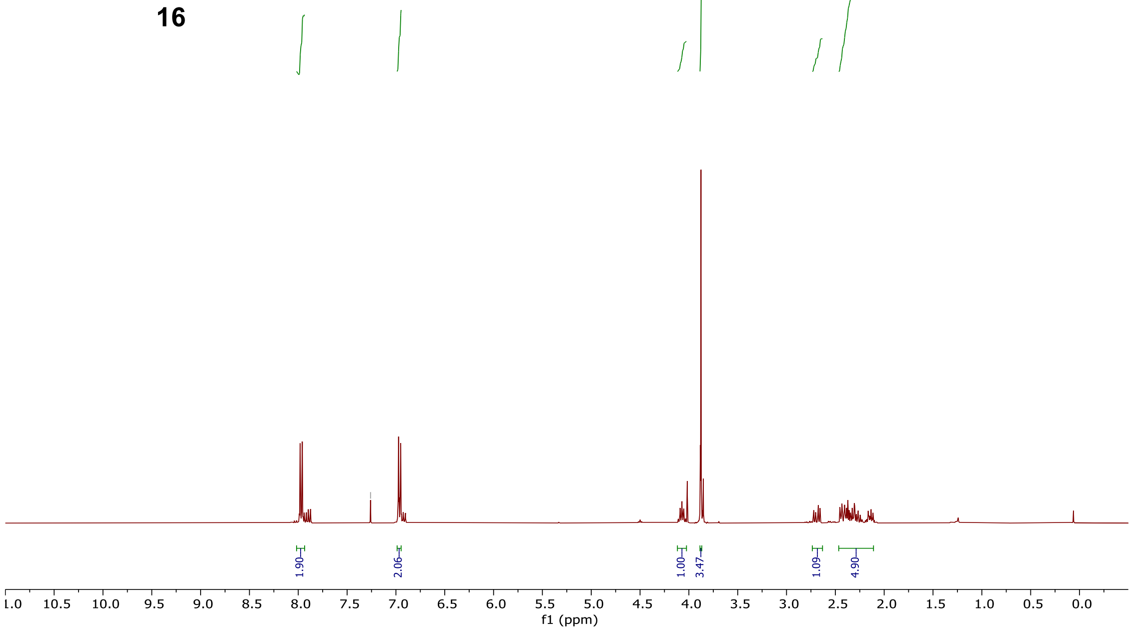

^{13}C NMR (101 MHz, $CDCl_3$)

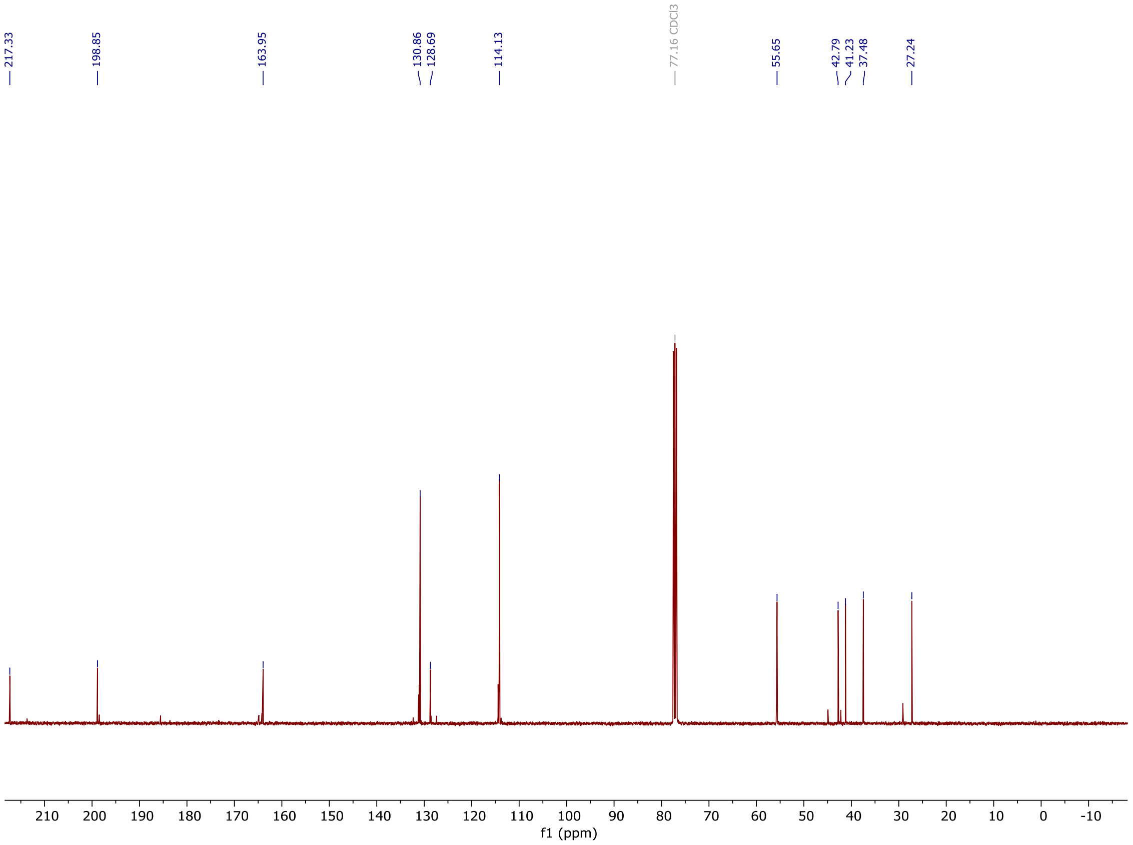

^{1}H NMR (400 MHz, $CDCl_3$)

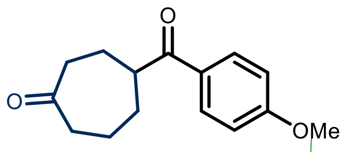

17a

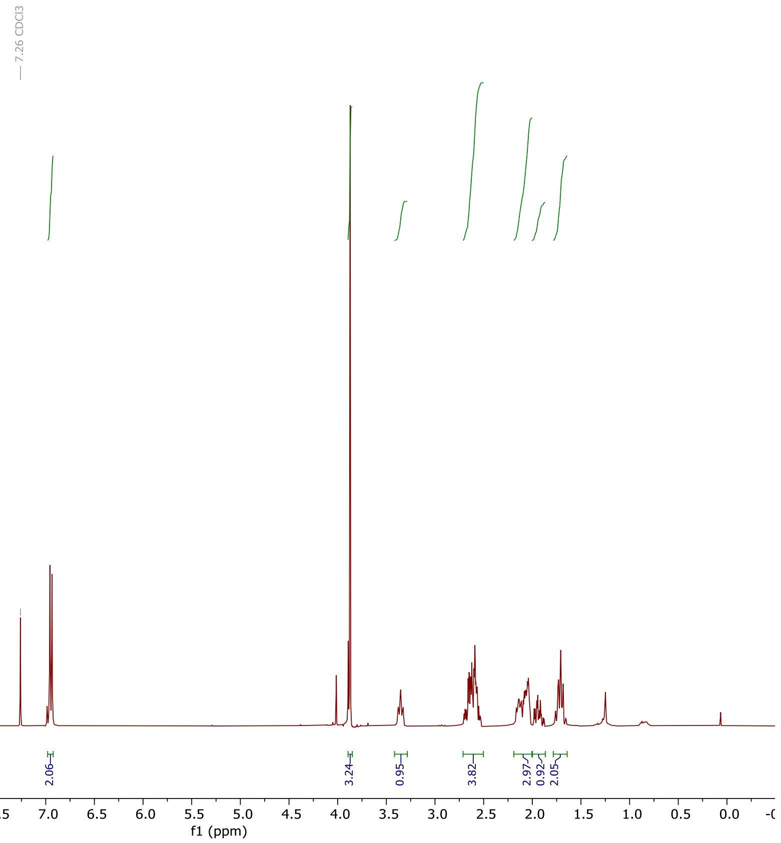

^{13}C NMR (101 MHz, $CDCl_3$)

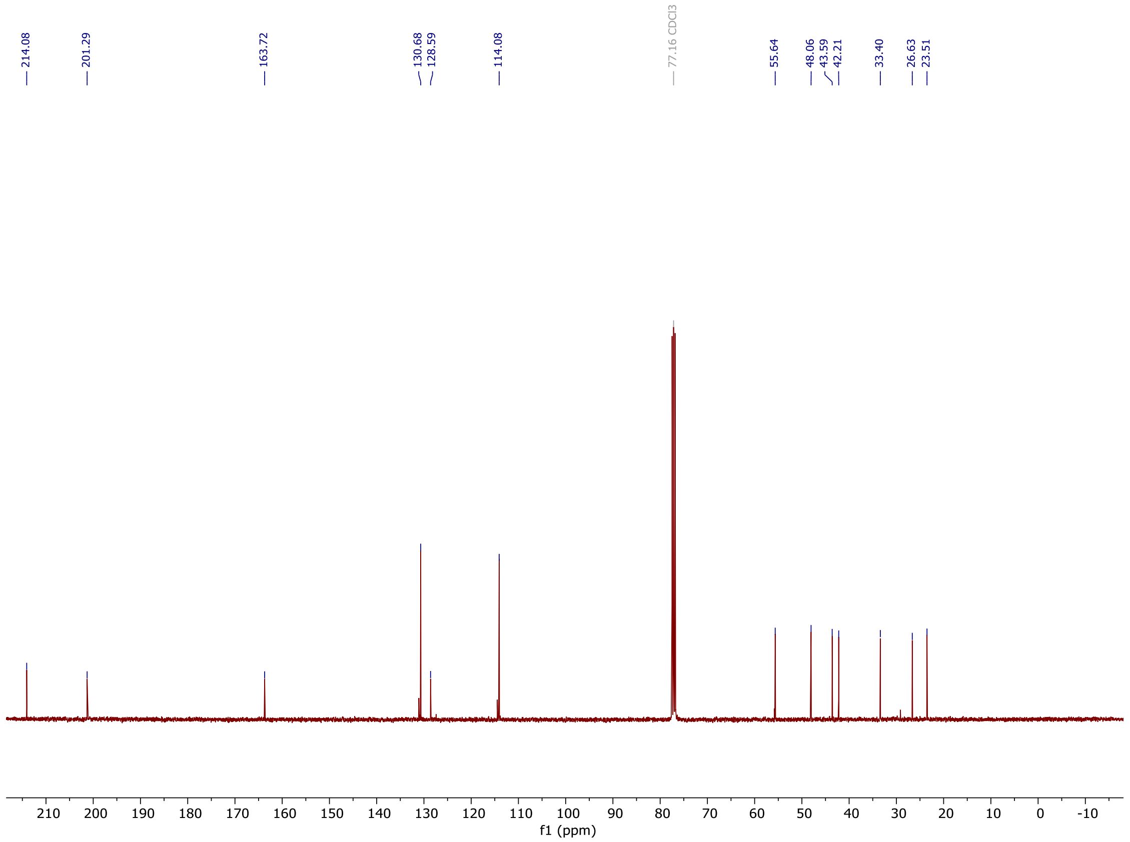

HSQC **17a**

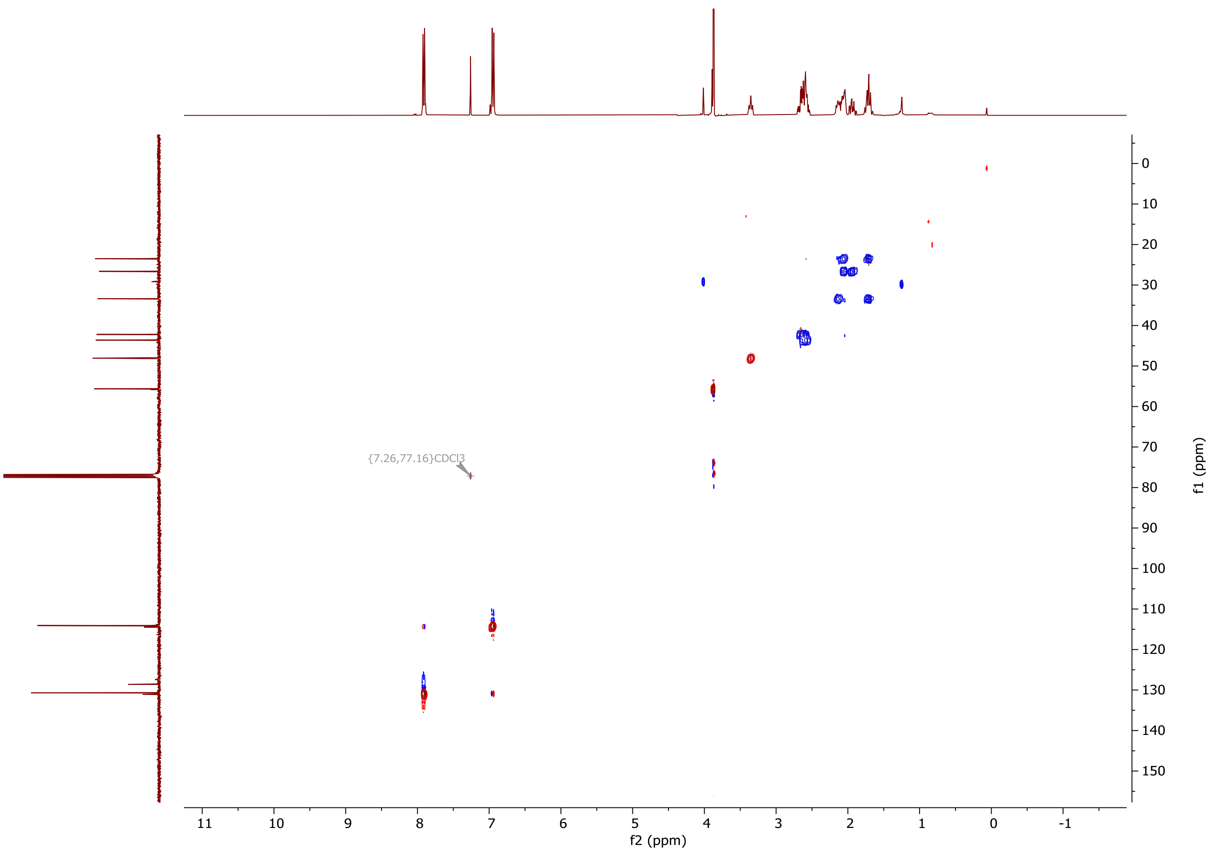

HMBC **17a**

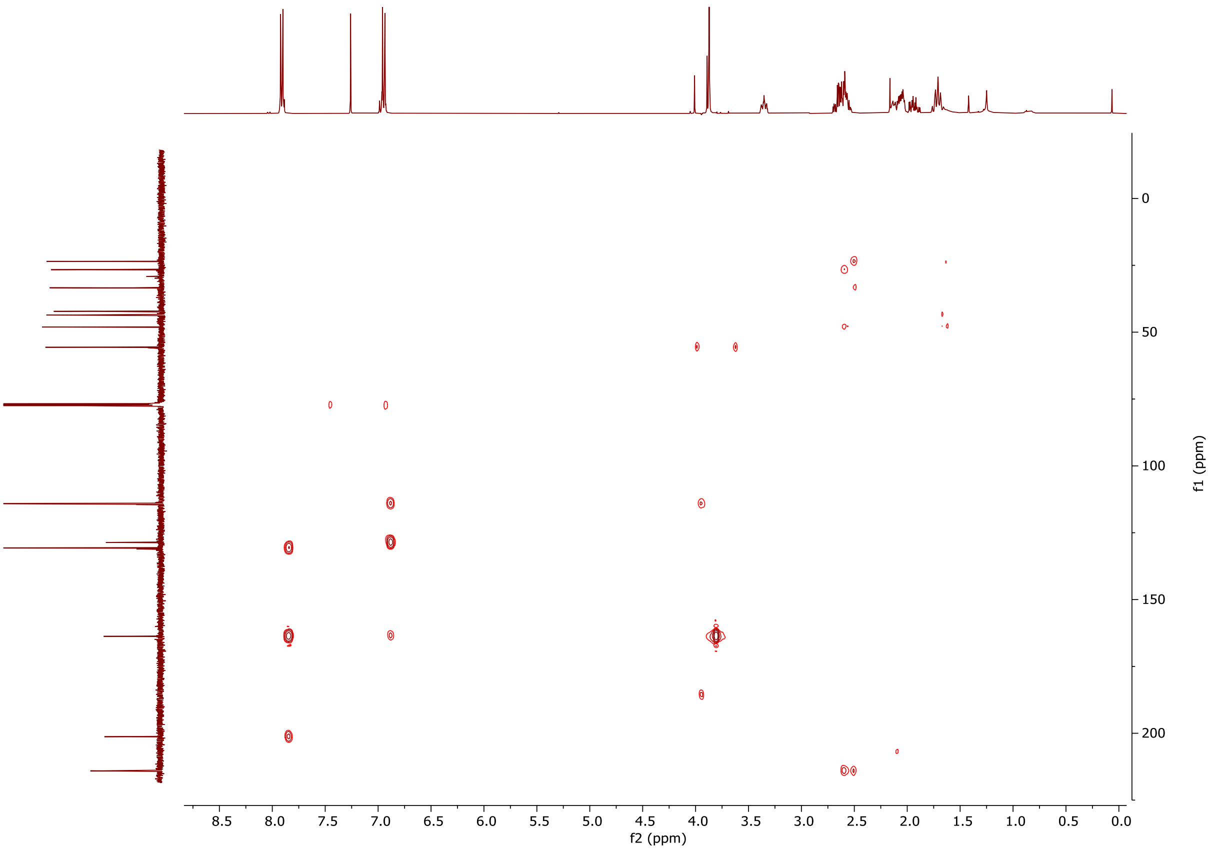

^{1}H NMR (400 MHz, $CDCl_3$)

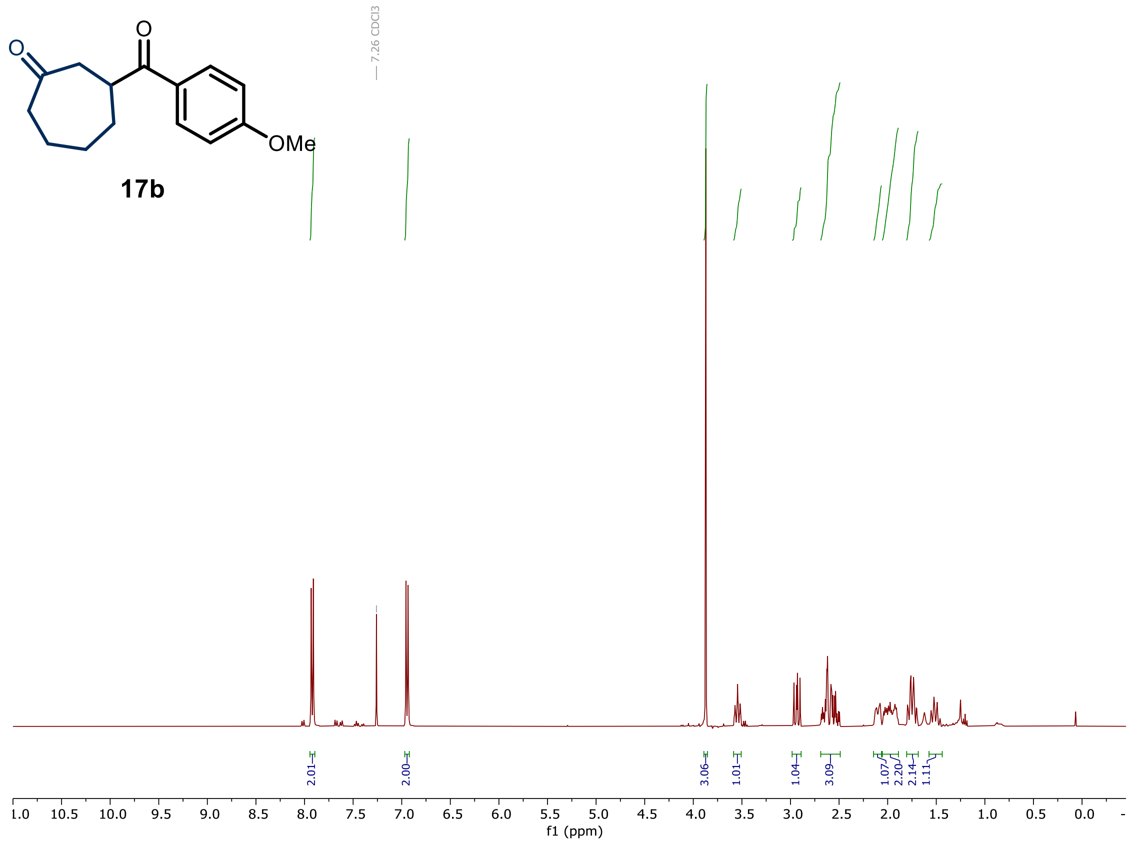

^{13}C NMR (101 MHz, $CDCl_3$)

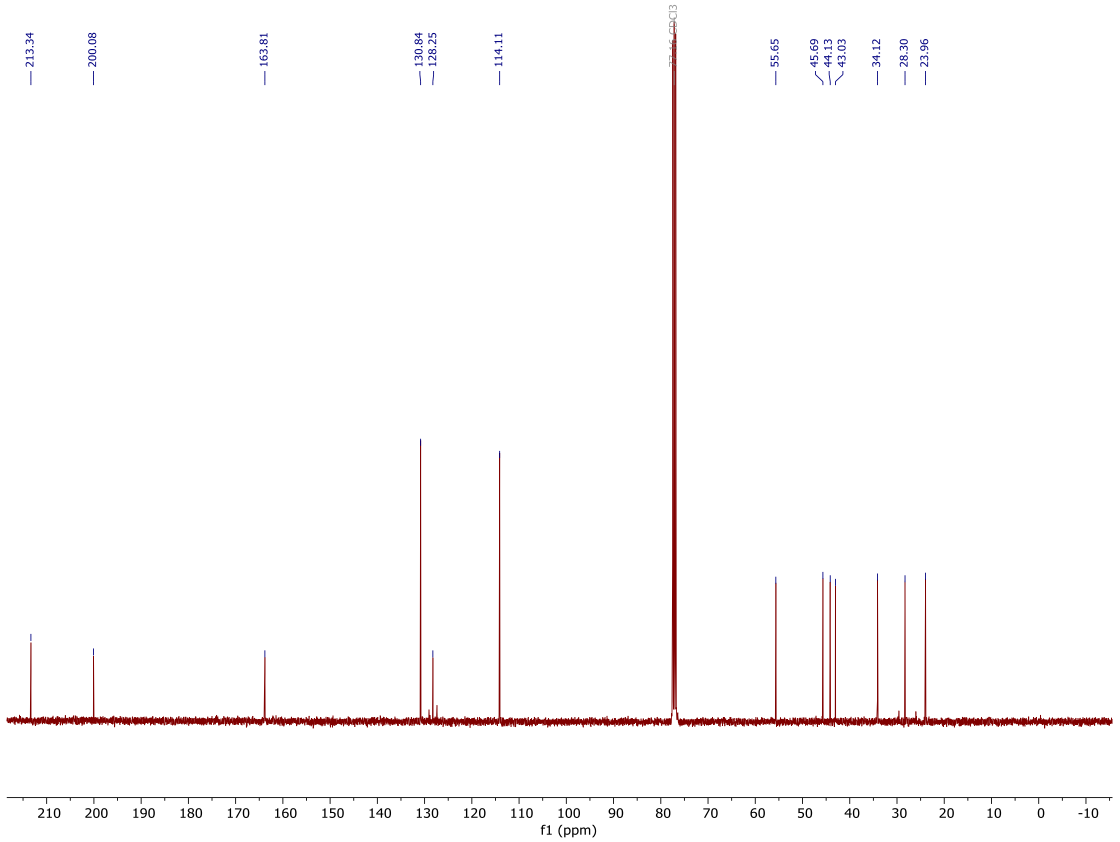

COSY **17b**

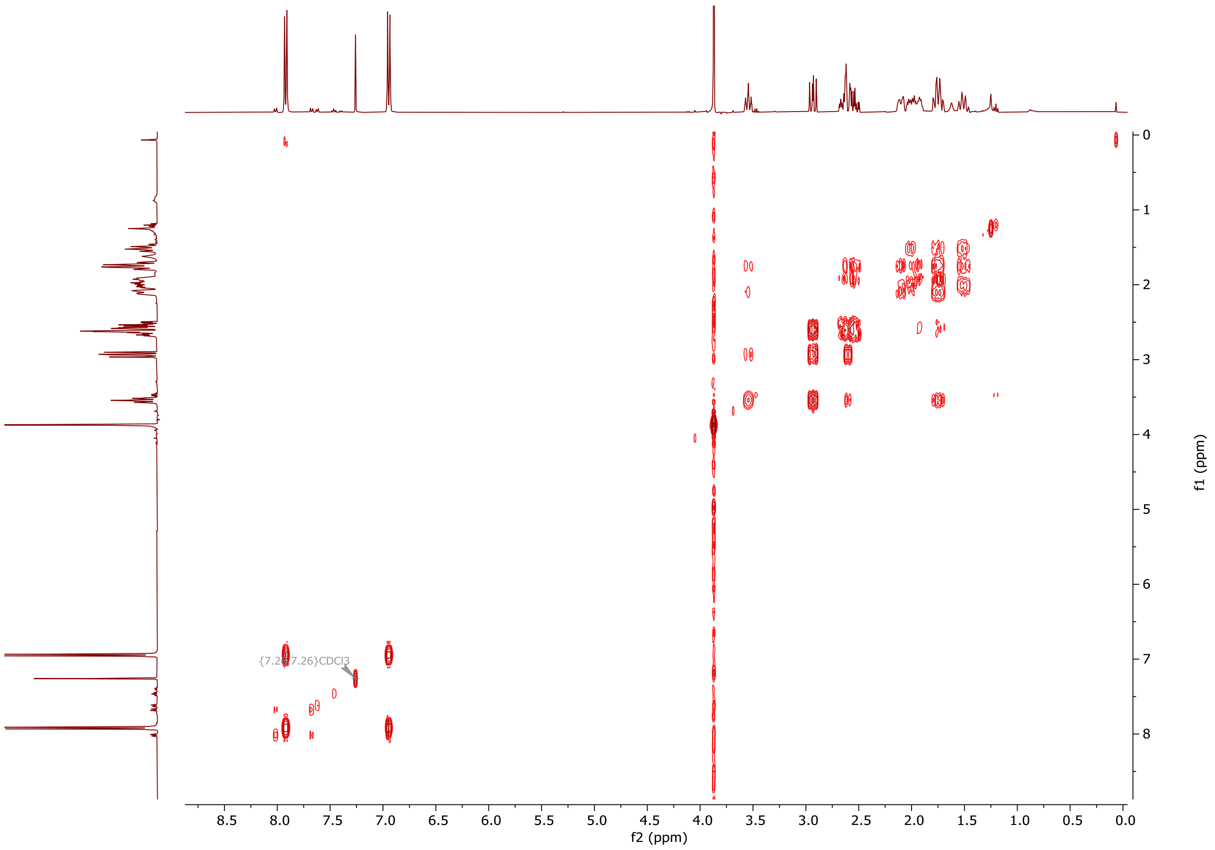

HMBC **17b**

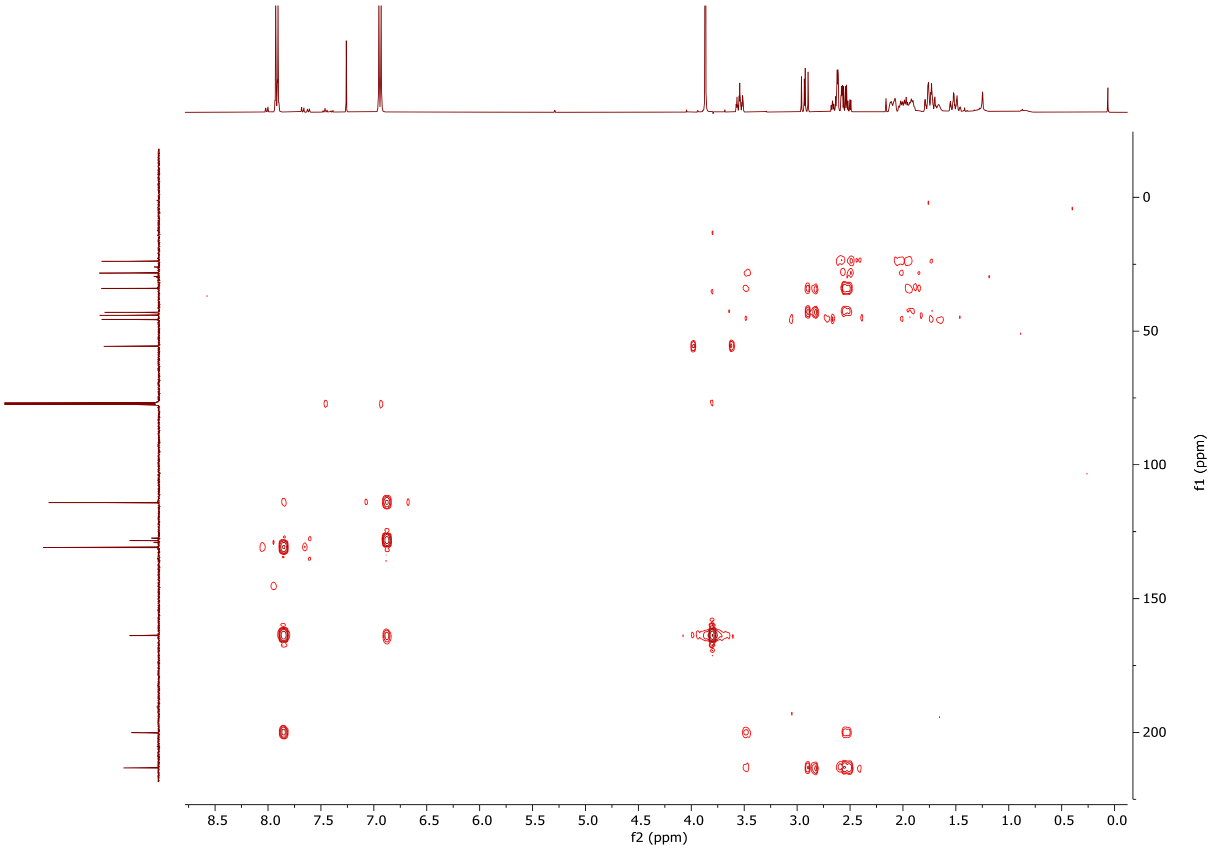

^{1}H NMR (400 MHz, $CDCl_3$)

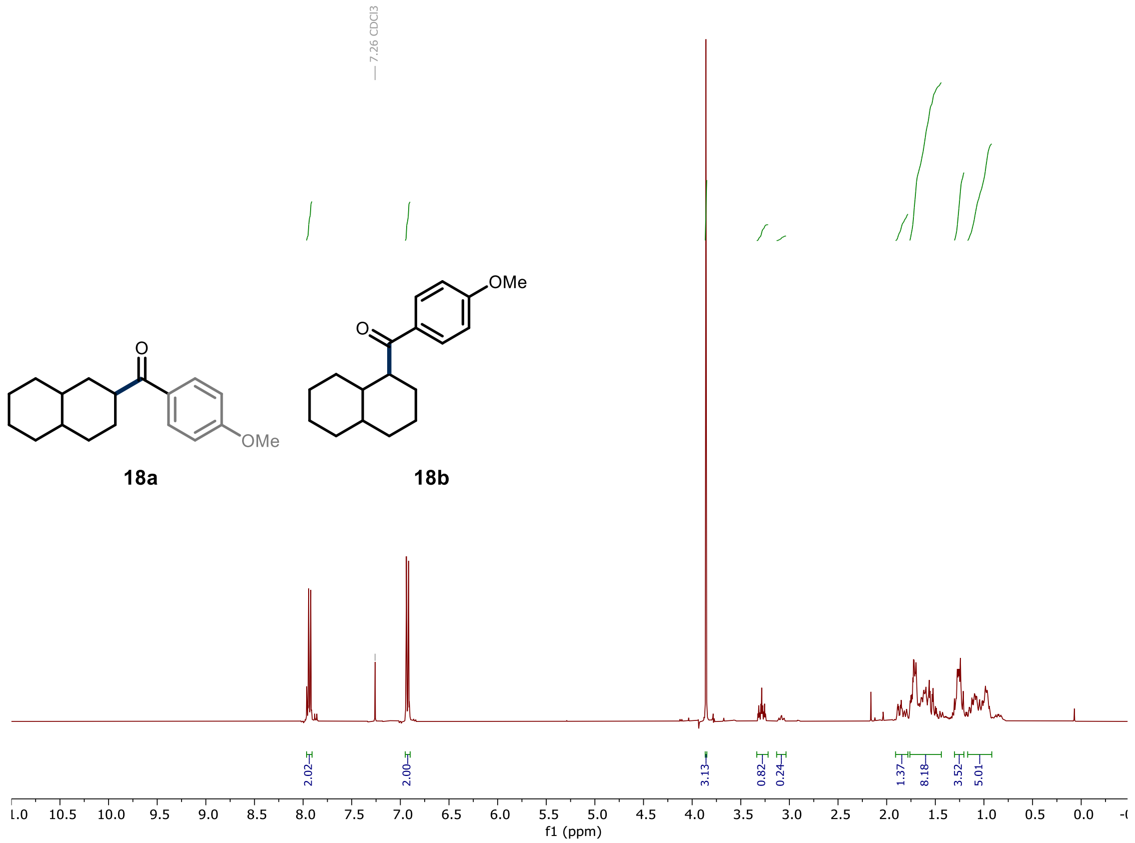

^{13}C NMR (101 MHz, $CDCl_3$)

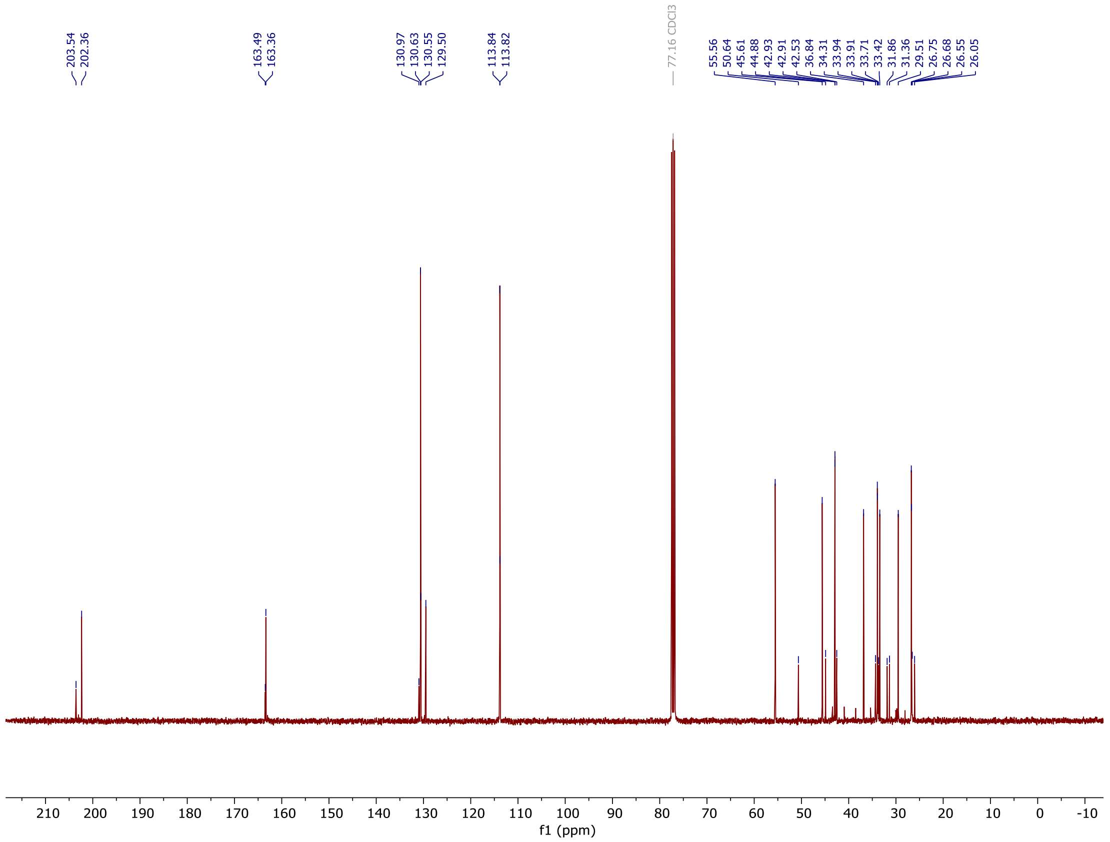

^{1}H NMR (300 MHz, $CDCl_3$)

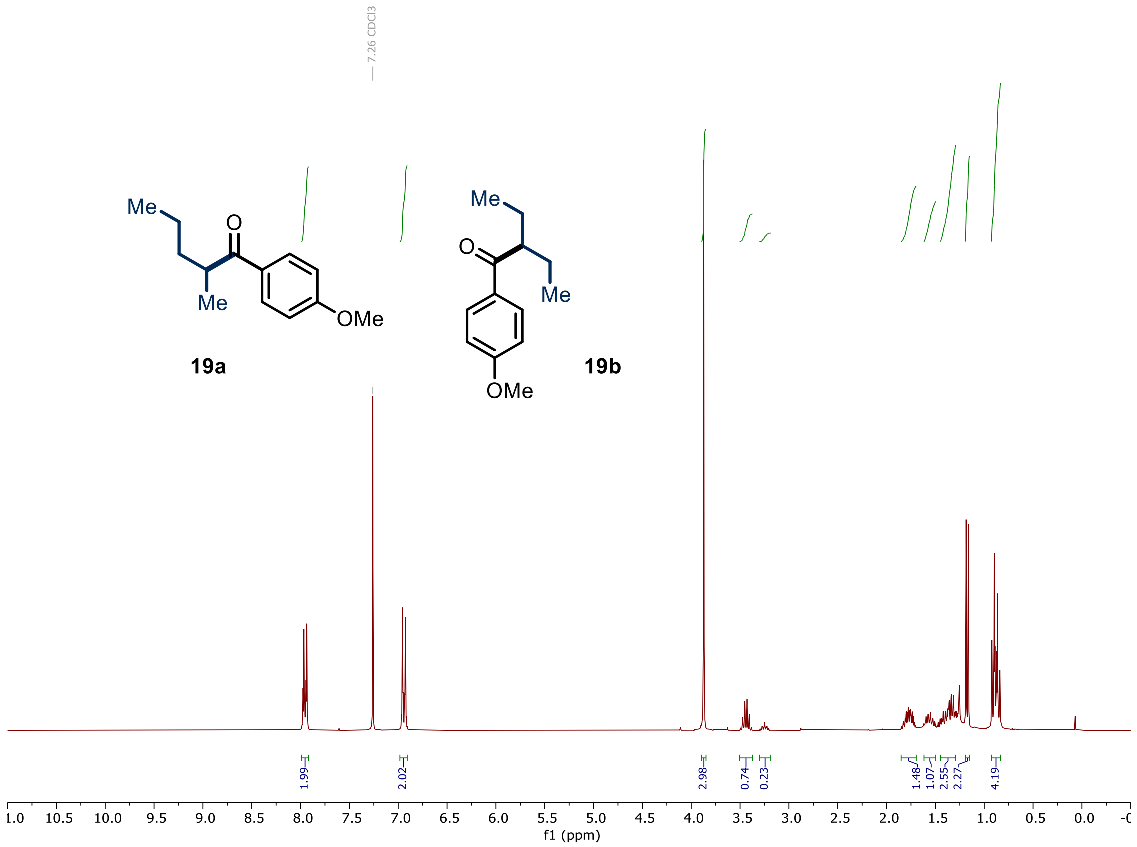

^{13}C NMR (101 MHz, $CDCl_3$)

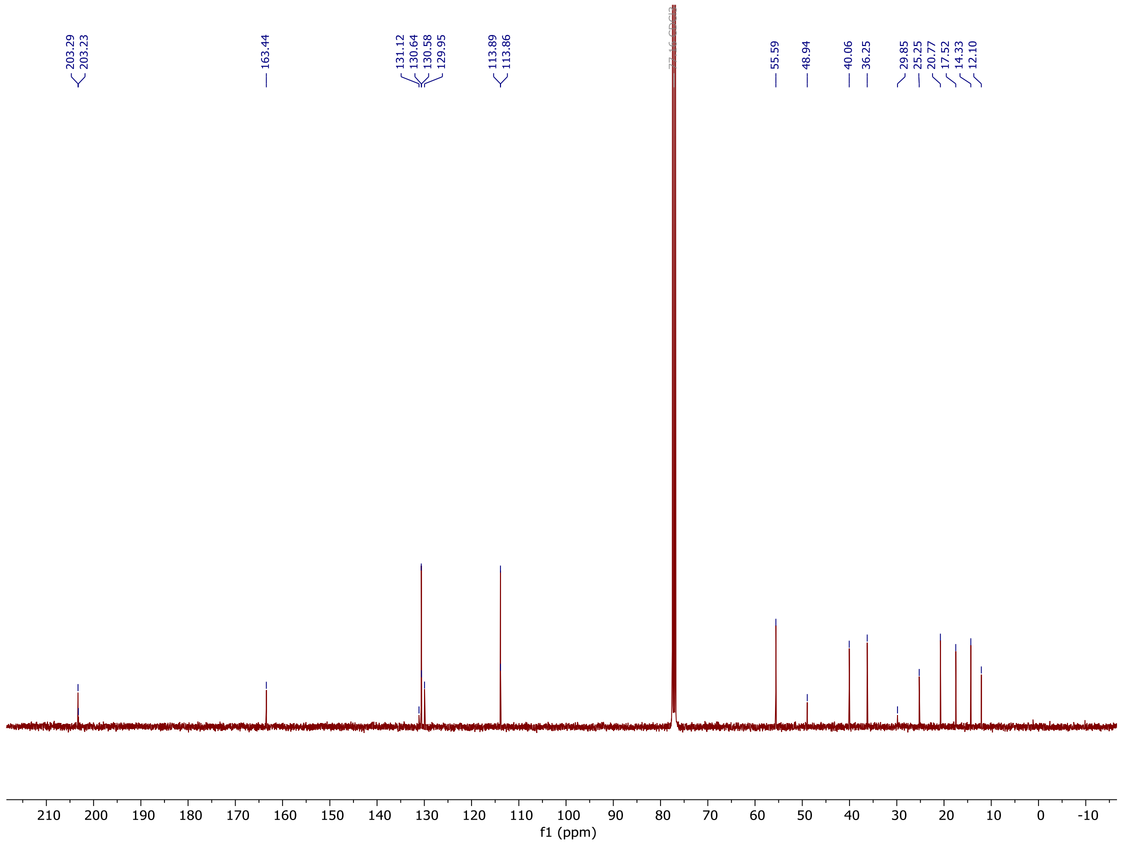

COSY **19a** and **19b**

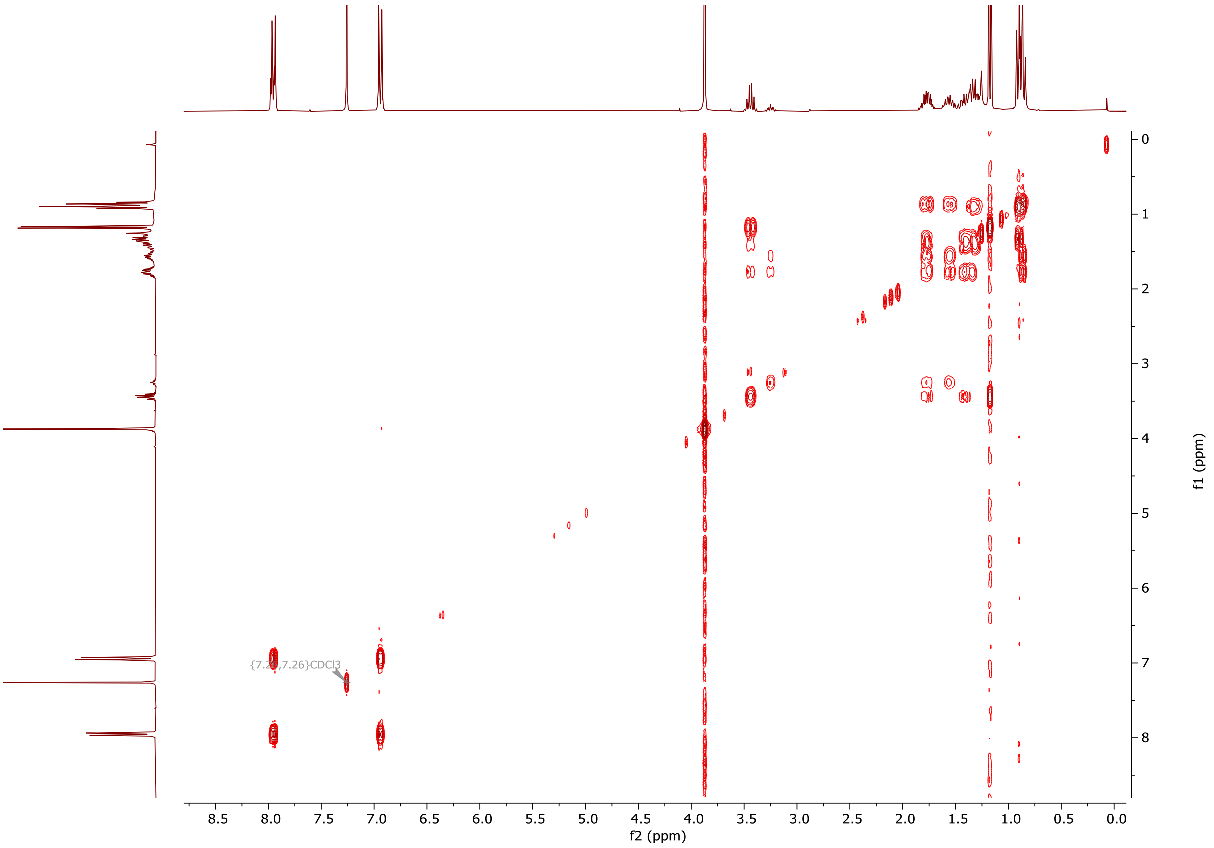

^1H NMR (400 MHz, $CDCl_3$)

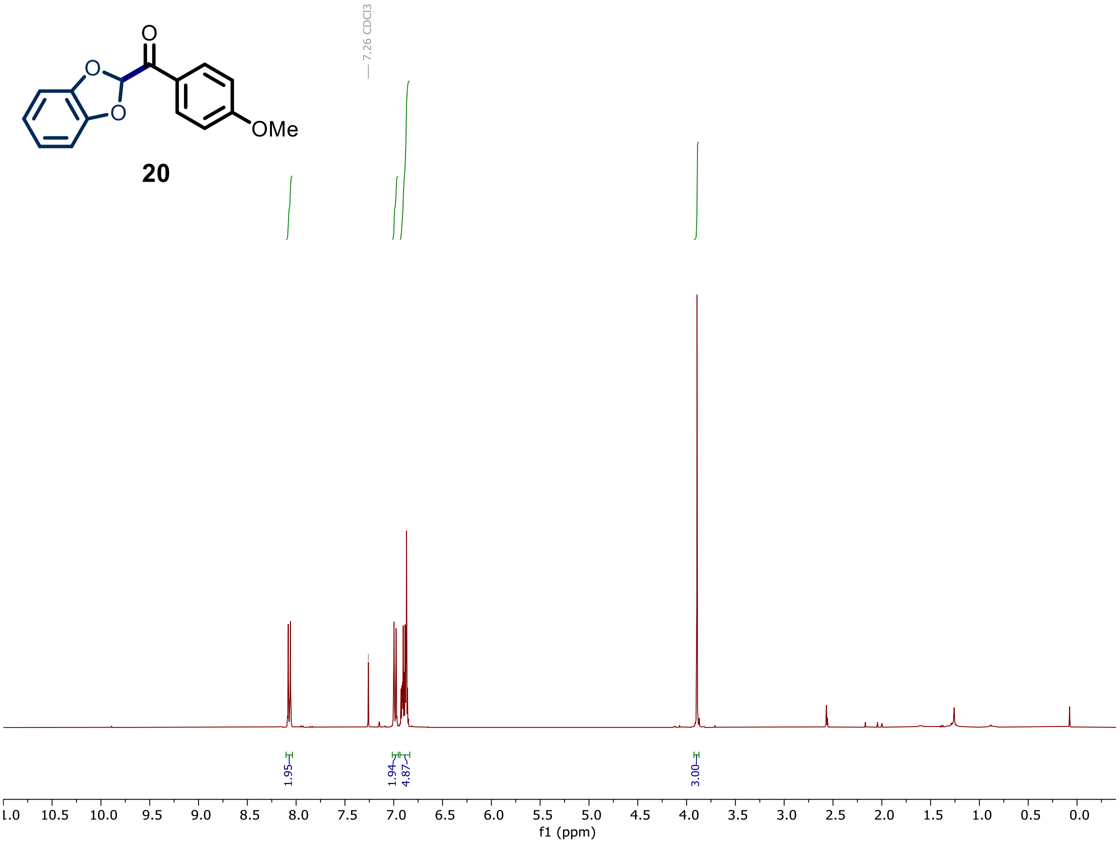

^{13}C NMR (101 MHz, $CDCl_3$)

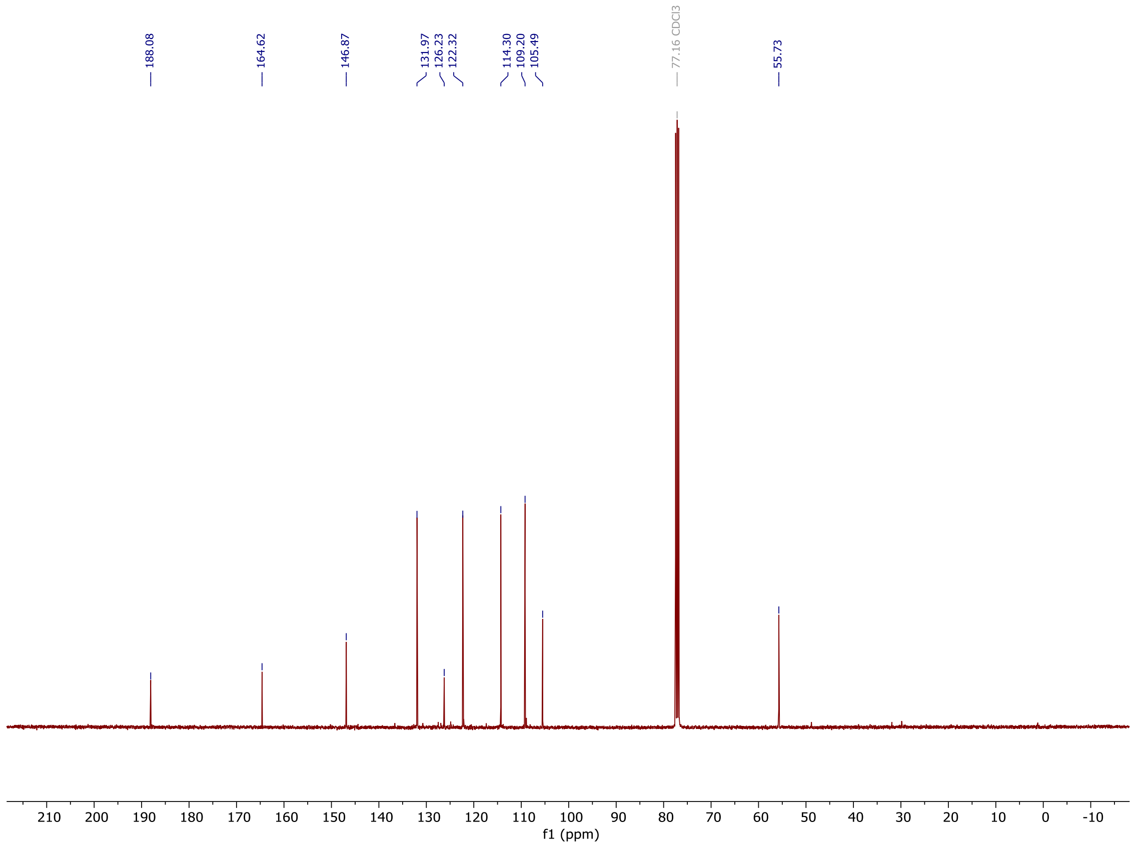

^{1}H NMR (400 MHz, $CDCl_3$)

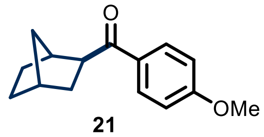

21

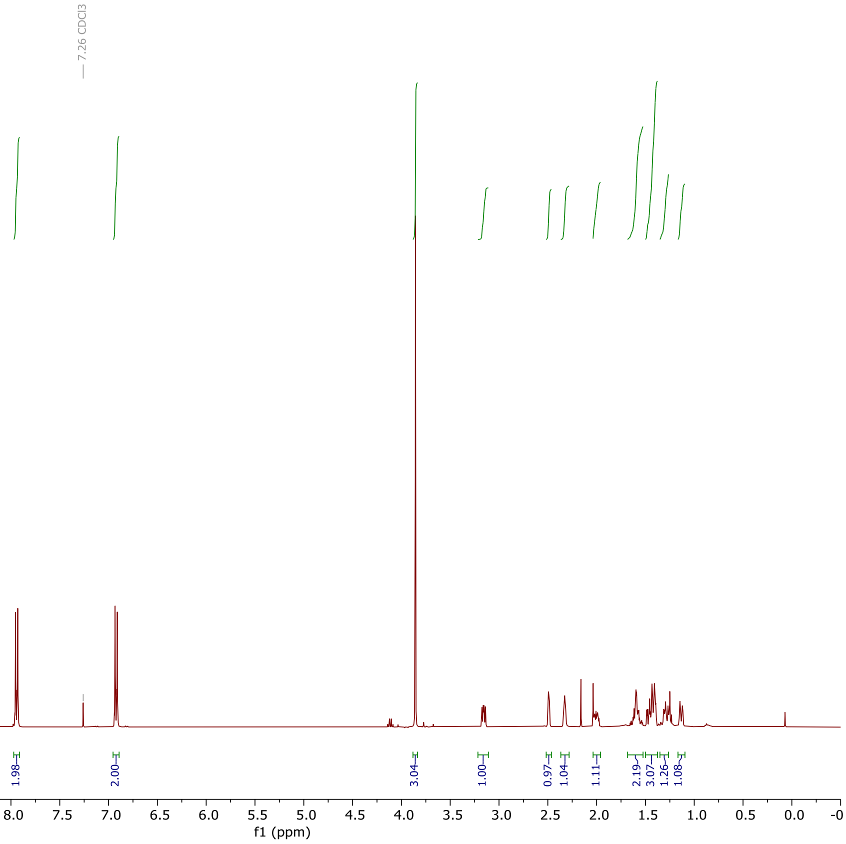

^{13}C NMR (101 MHz, $CDCl_3$)

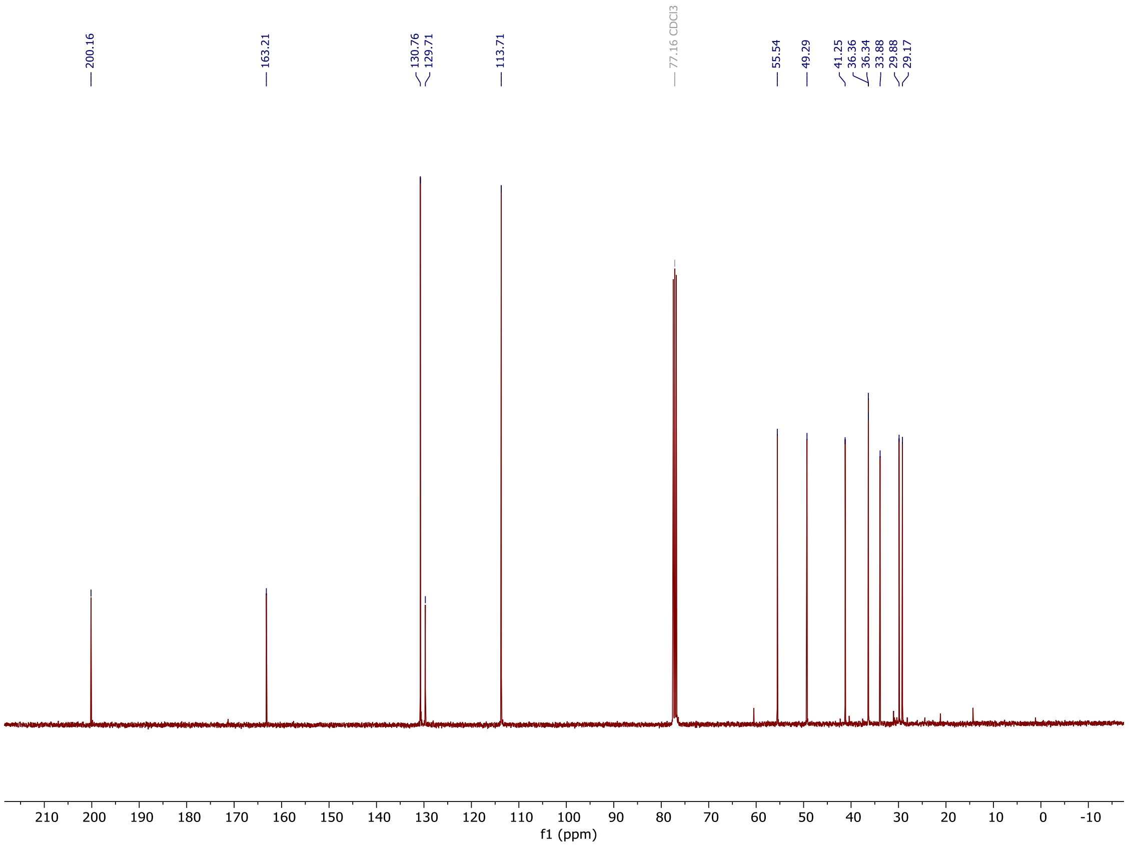

^{1}H NMR (400 MHz, $CDCl_3$)

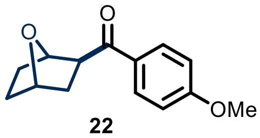

22

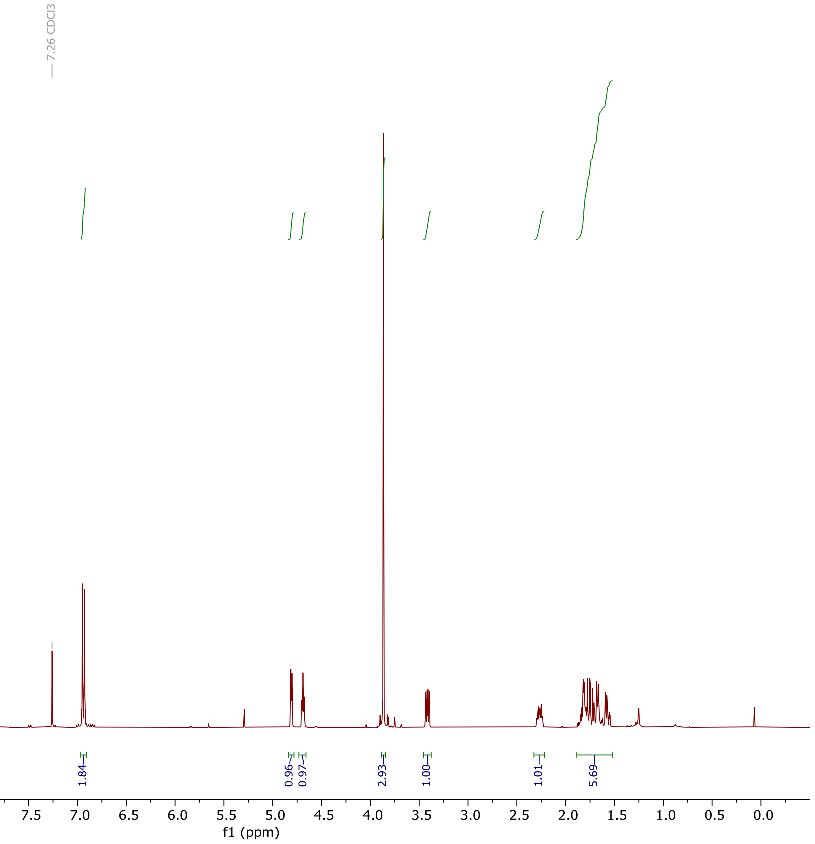

^{13}C NMR (101 MHz, $CDCl_3$)

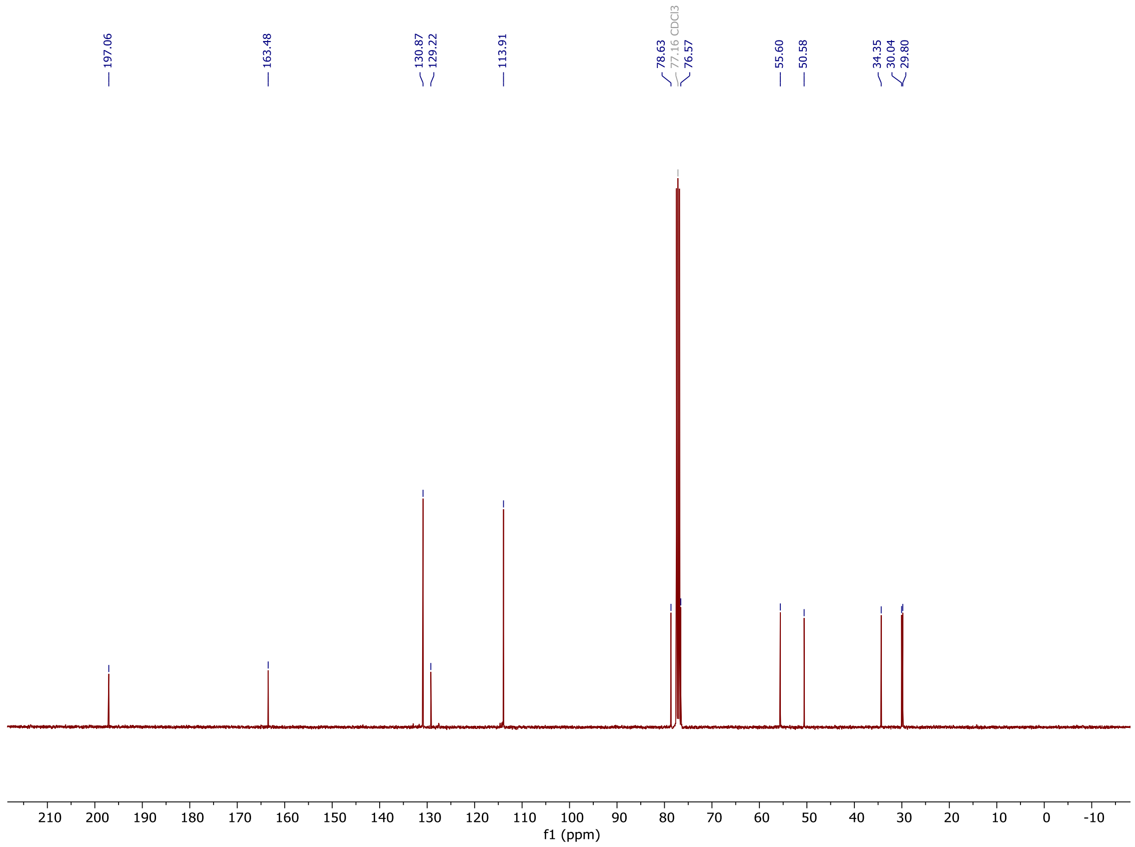

^{1}H NMR (400 MHz, $CDCl_3$)

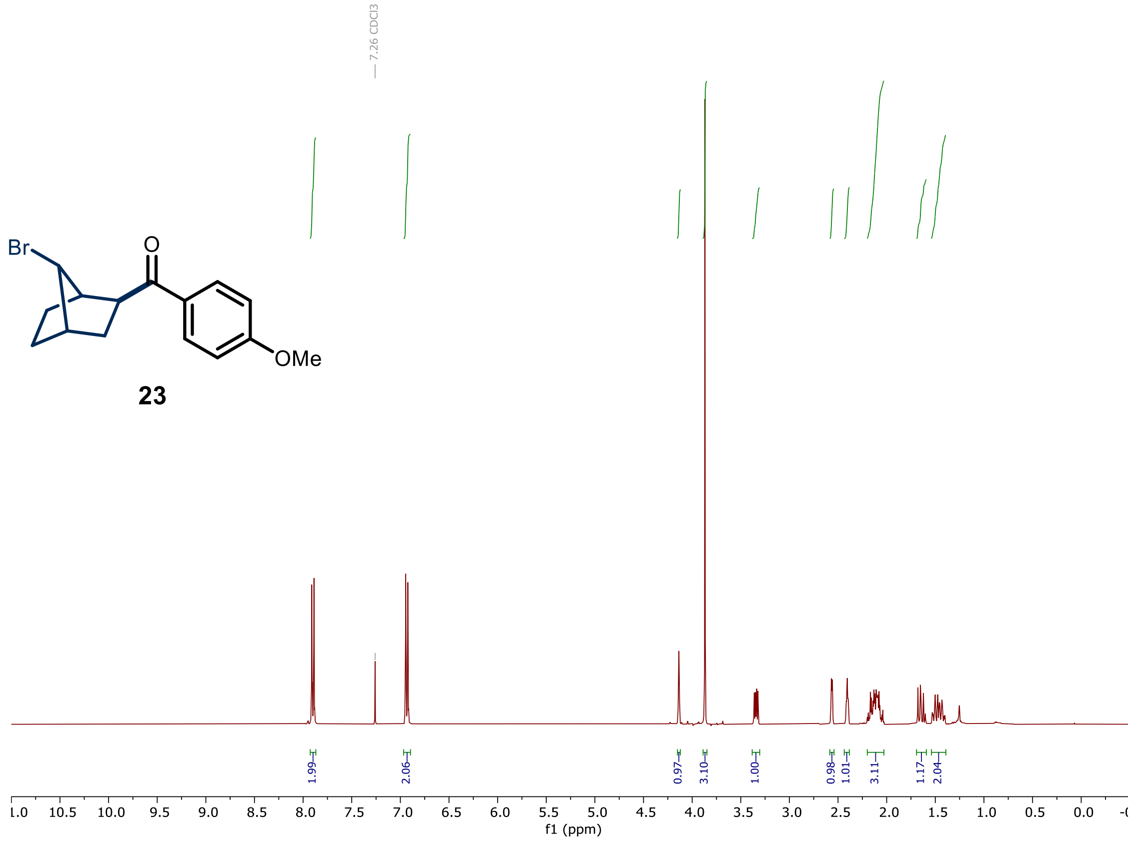

^{13}C NMR (101 MHz, $CDCl_3$)

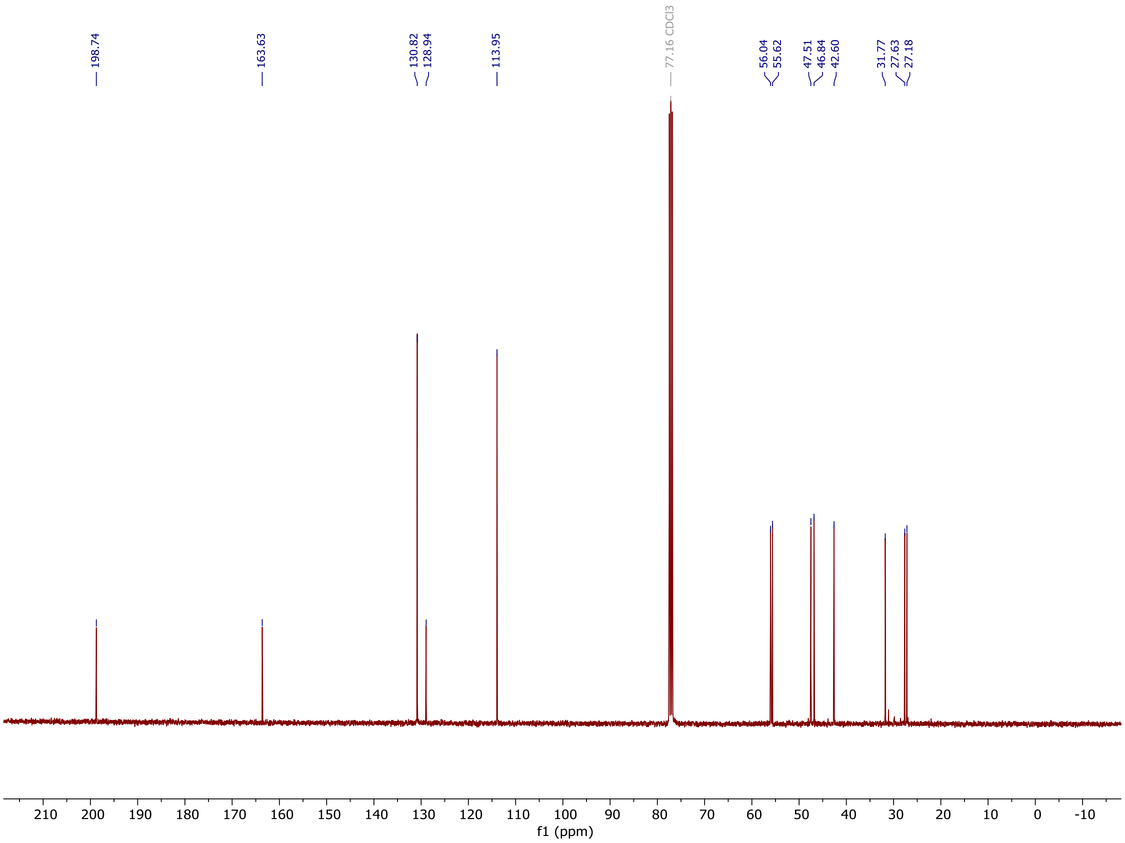

COSY **25a**

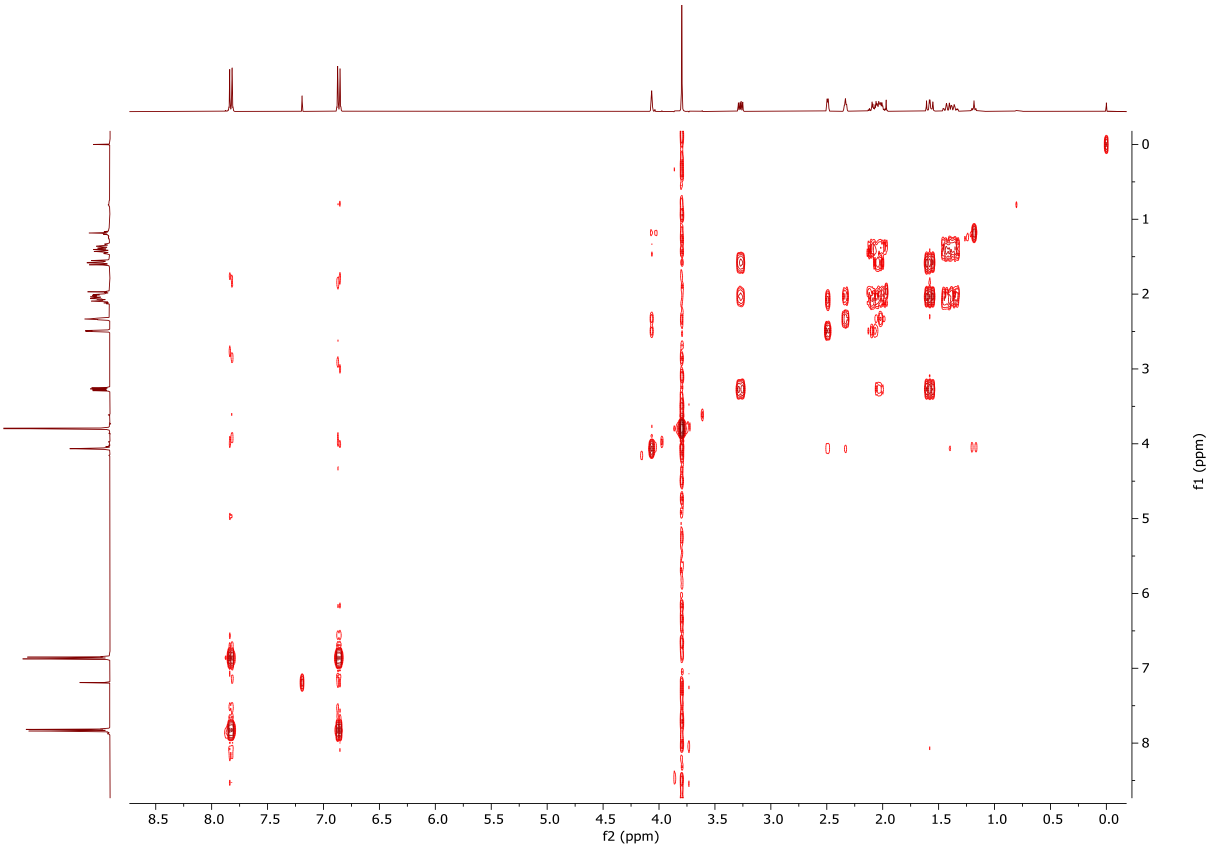

NOESY **25a**

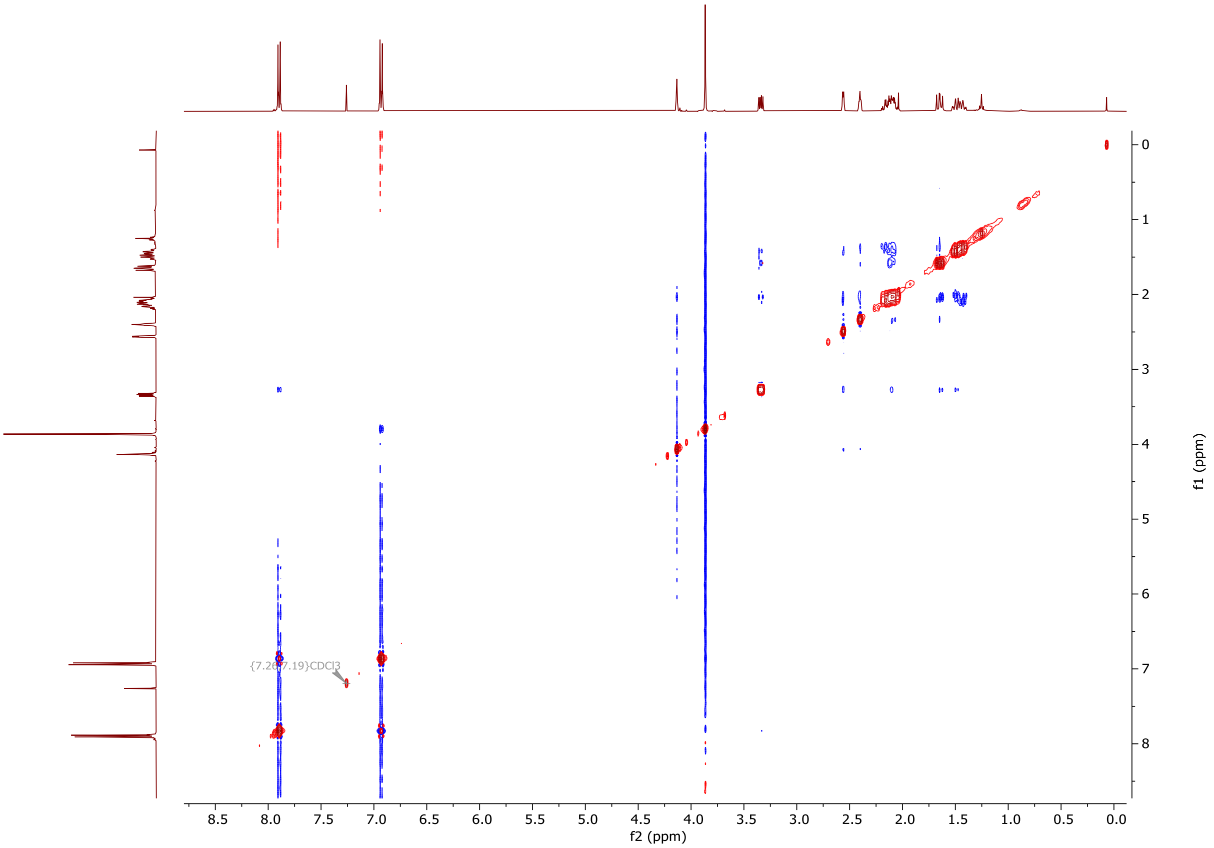

^{1}H NMR (400 MHz, $CDCl_3$)

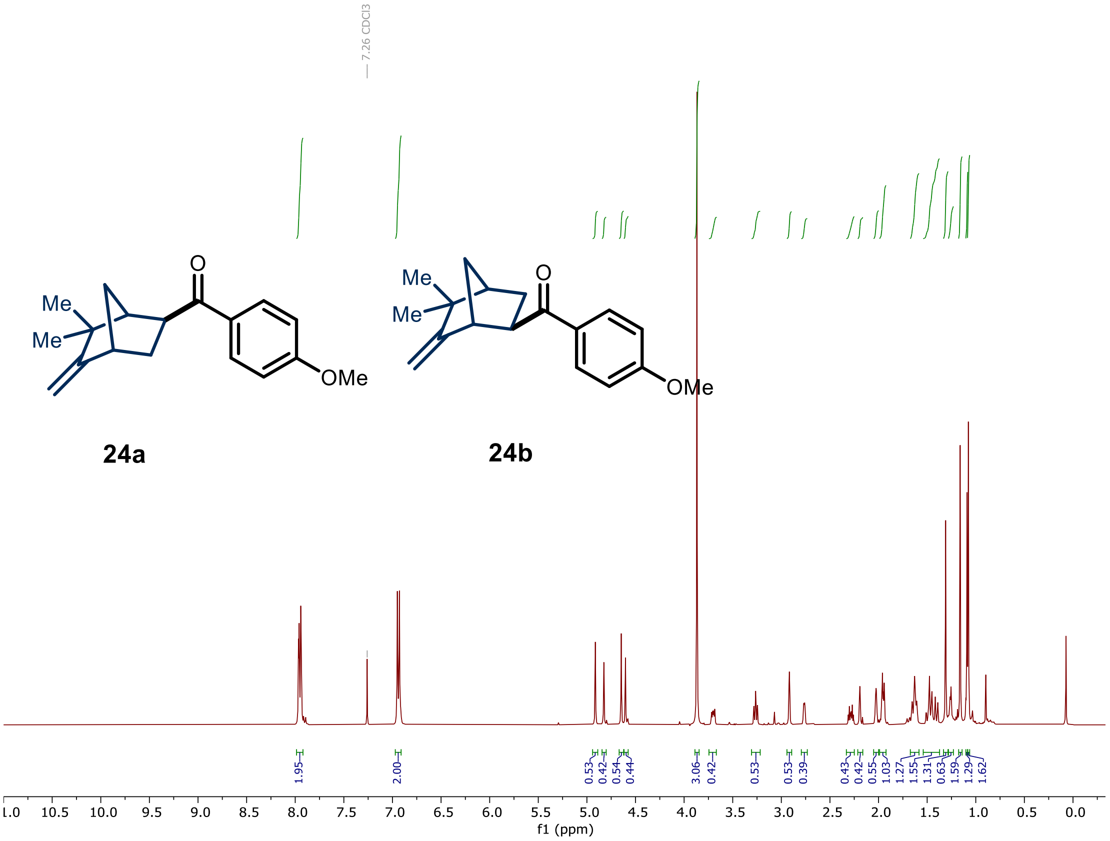

^{13}C NMR (101 MHz, $CDCl_3$)

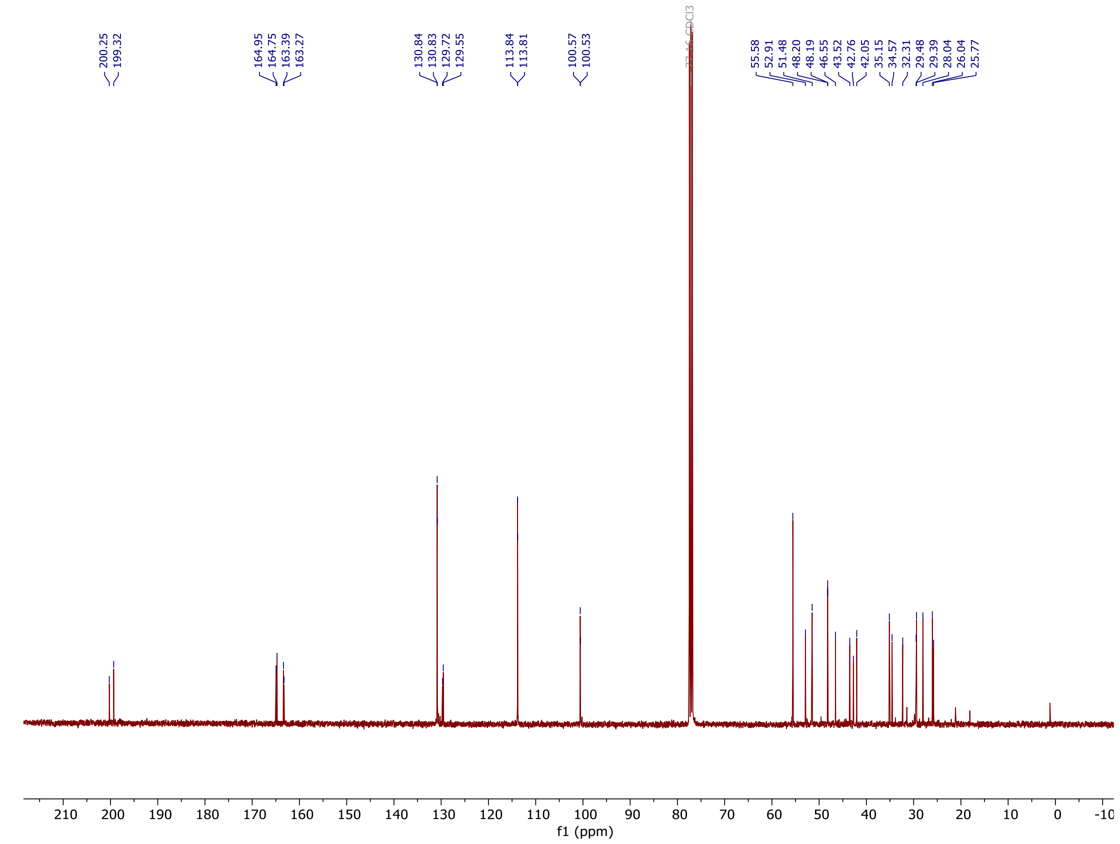

HSQC **24a** and **24b**

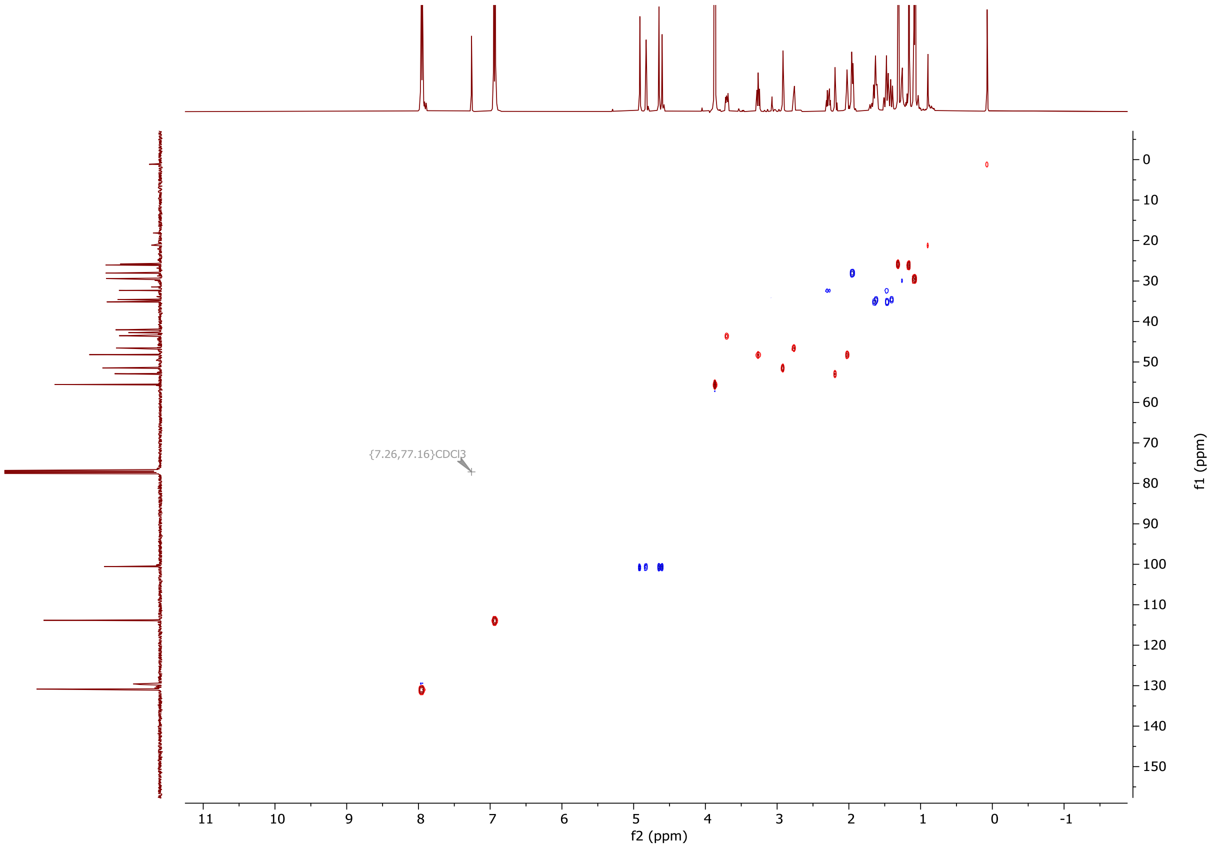

HMBC **24a** and **24b**

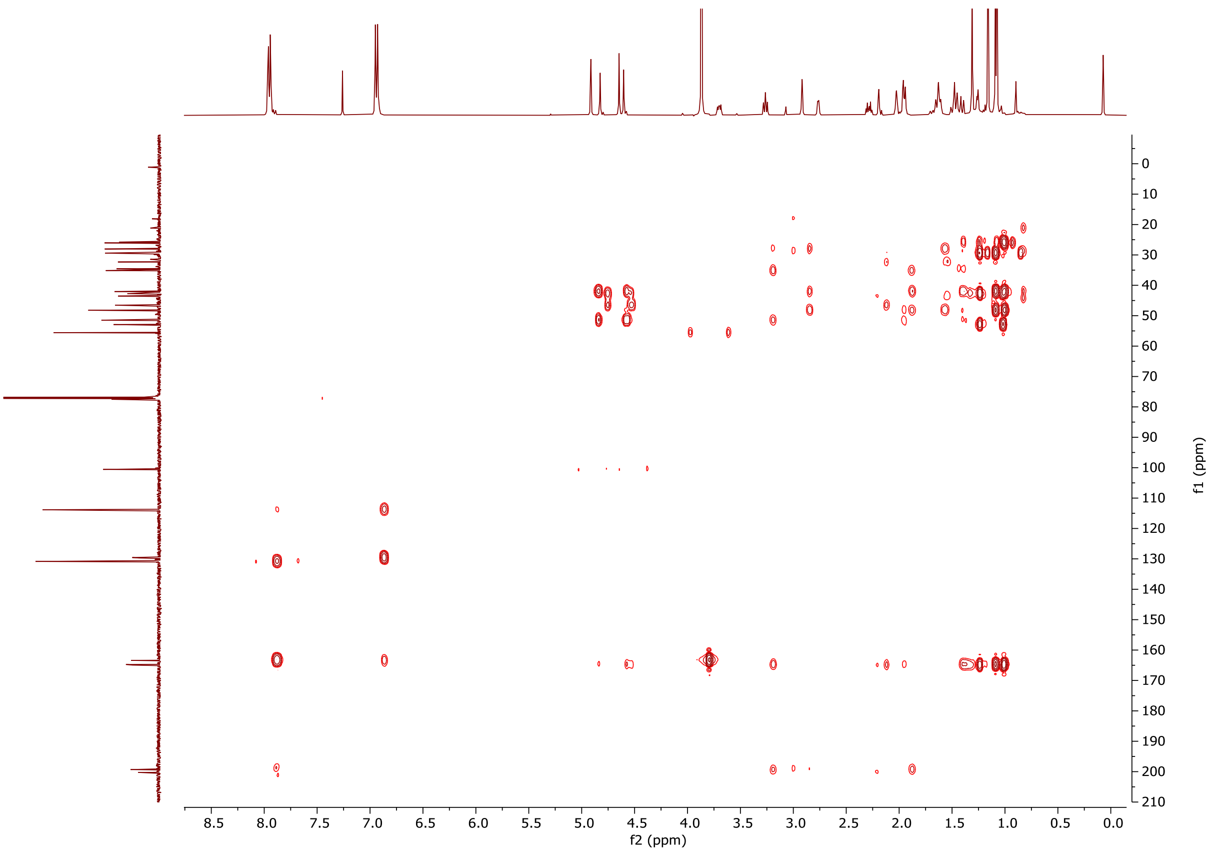

NOESY **24a** and **24b**

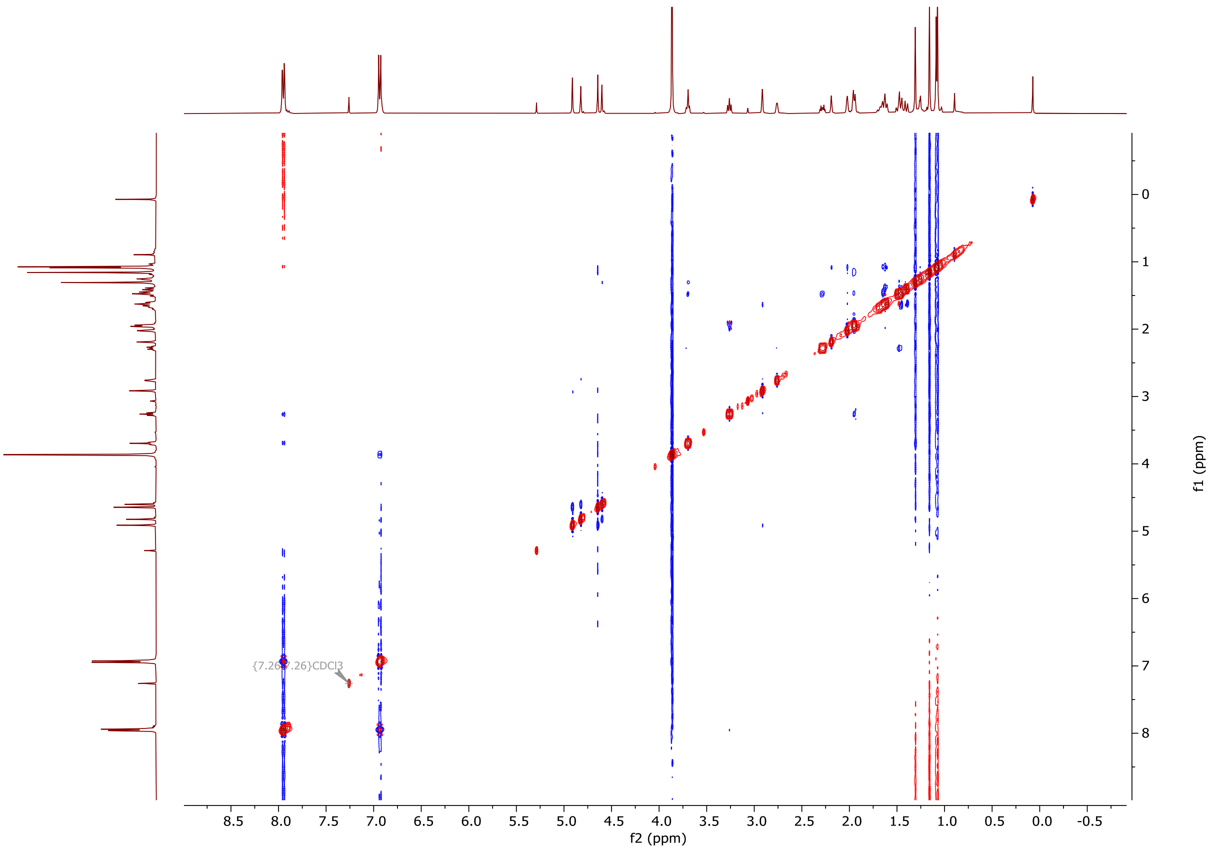

^{1}H NMR (400 MHz, $CDCl_3$)

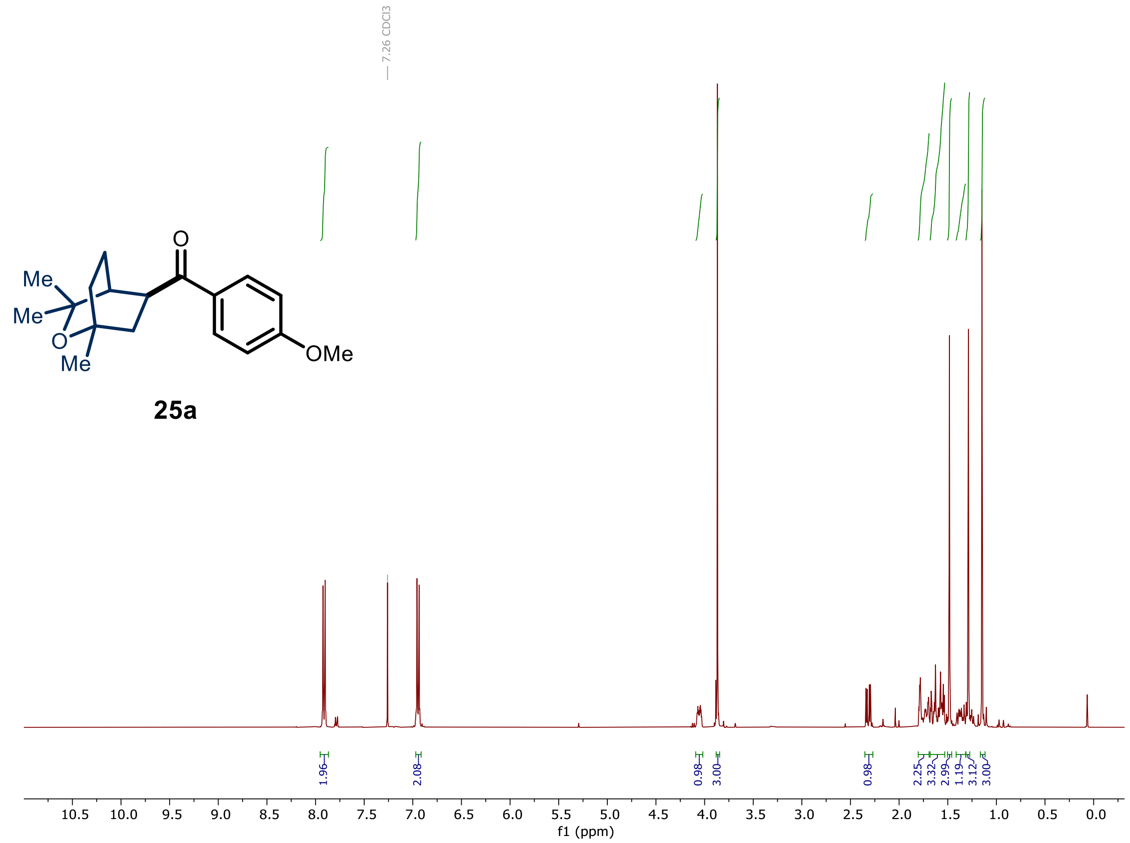

25a

^{13}C NMR (101 MHz, $CDCl_3$)

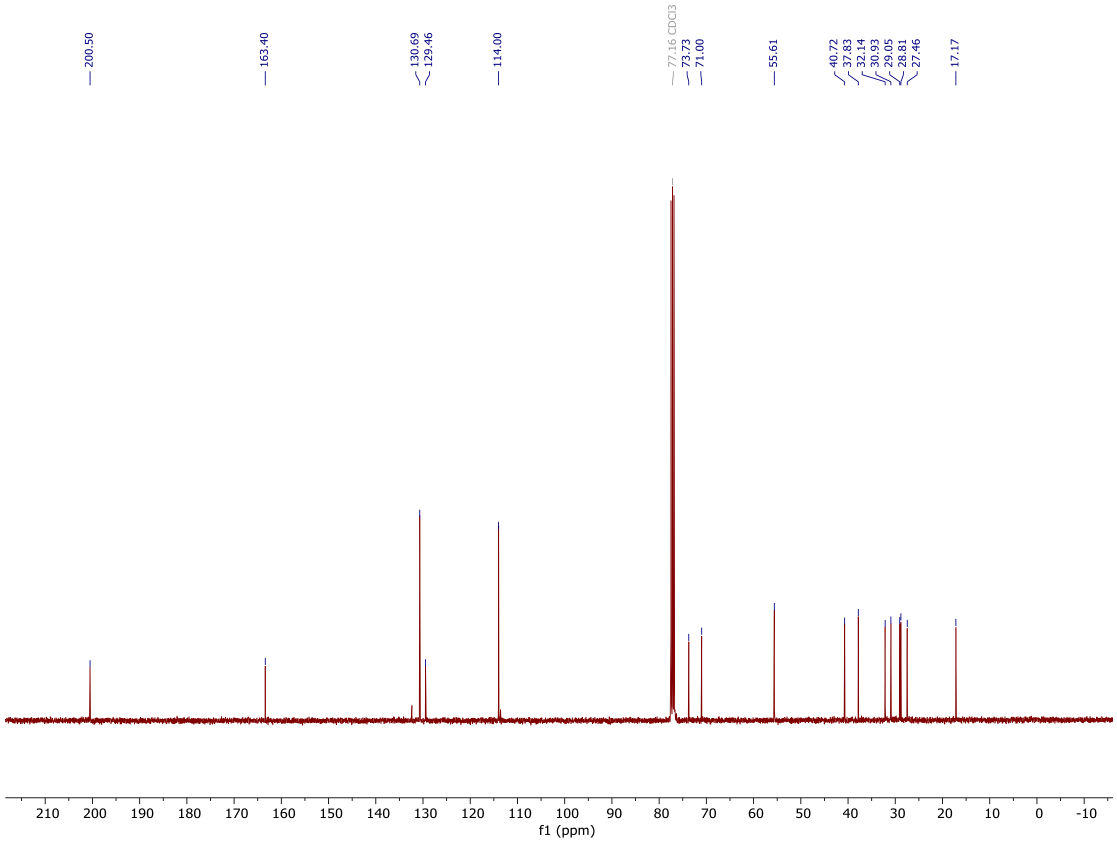

COSY **25a**

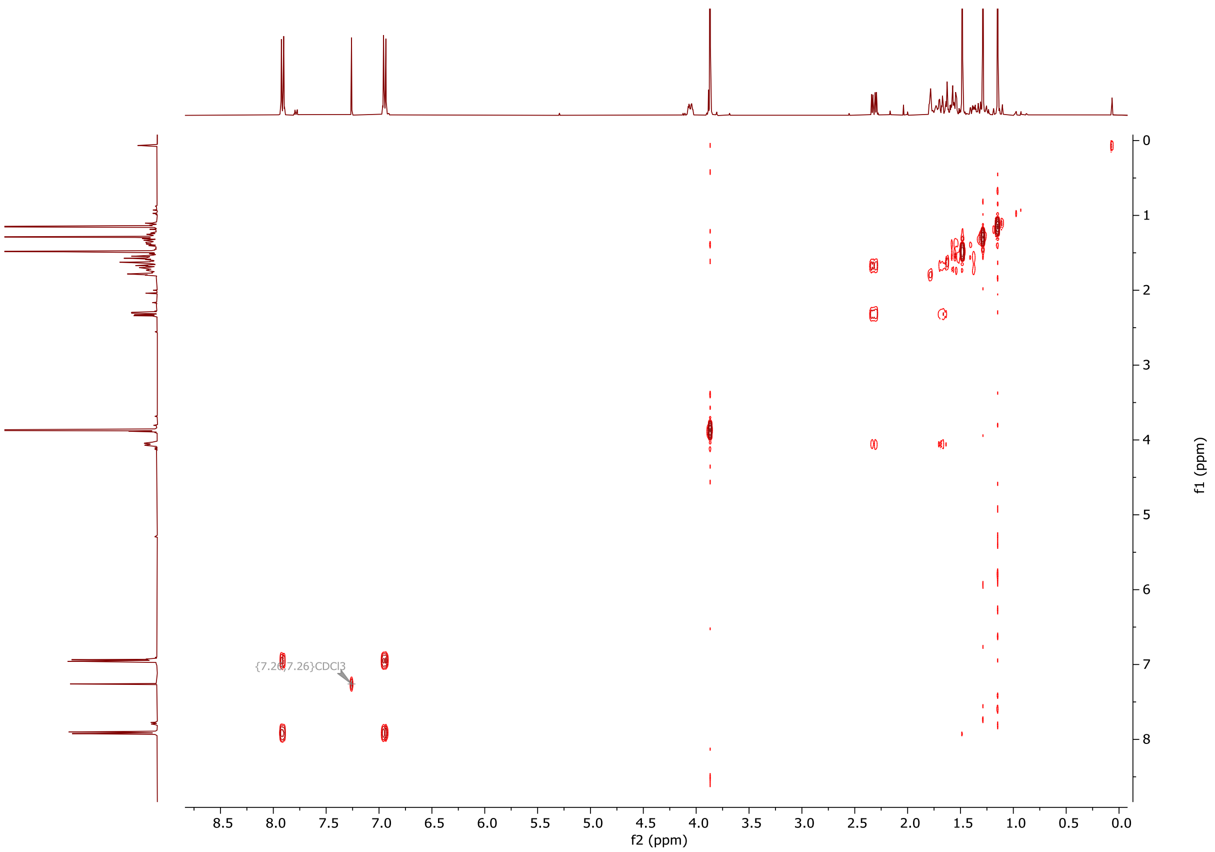

HSQC **25a**

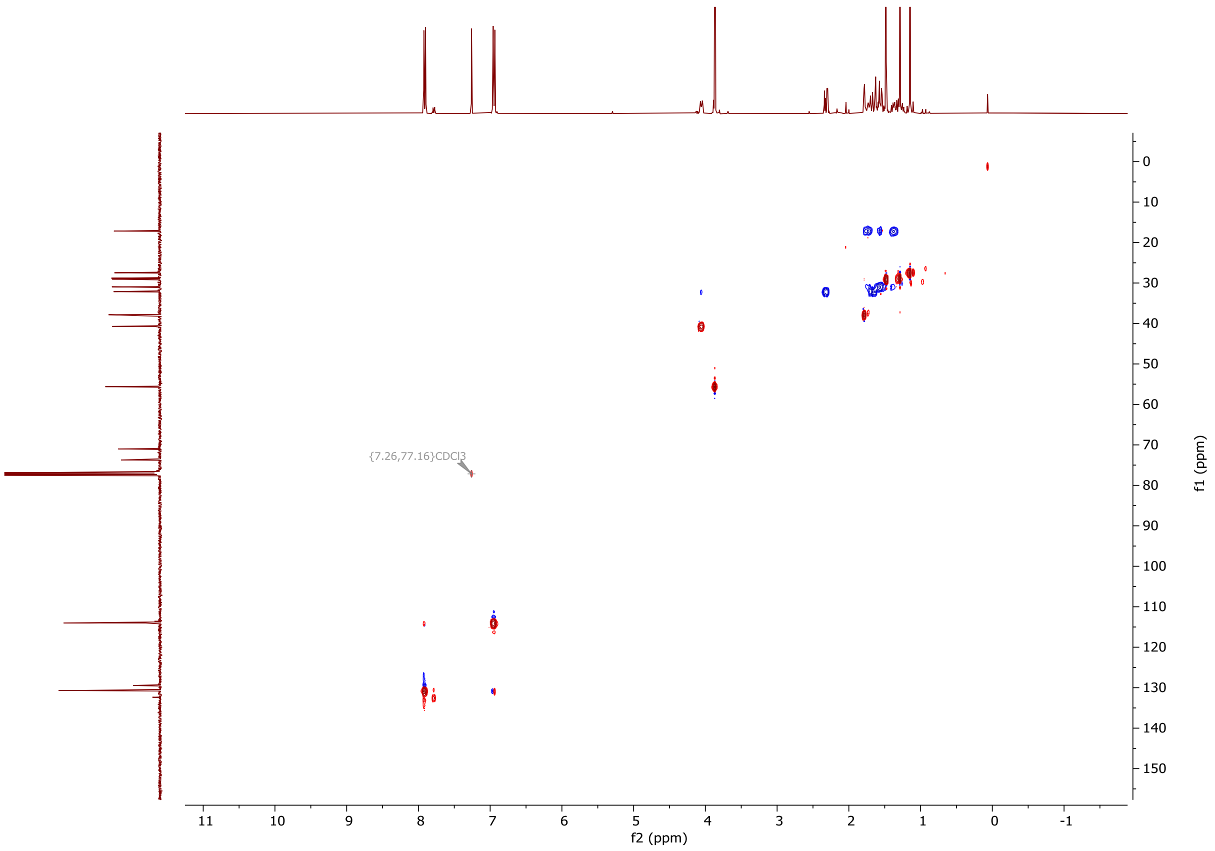

HMBC **25a**

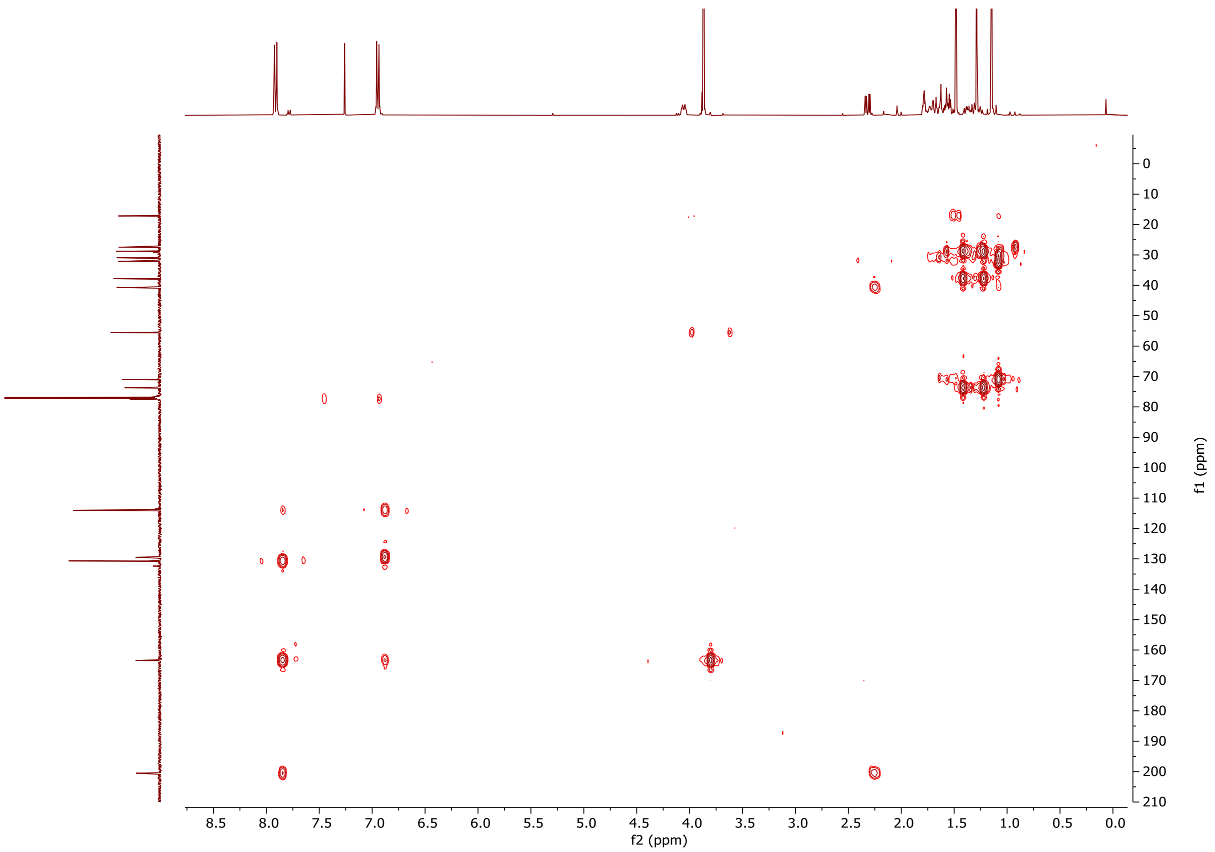

^{1}H NMR (400 MHz, $CDCl_3$)

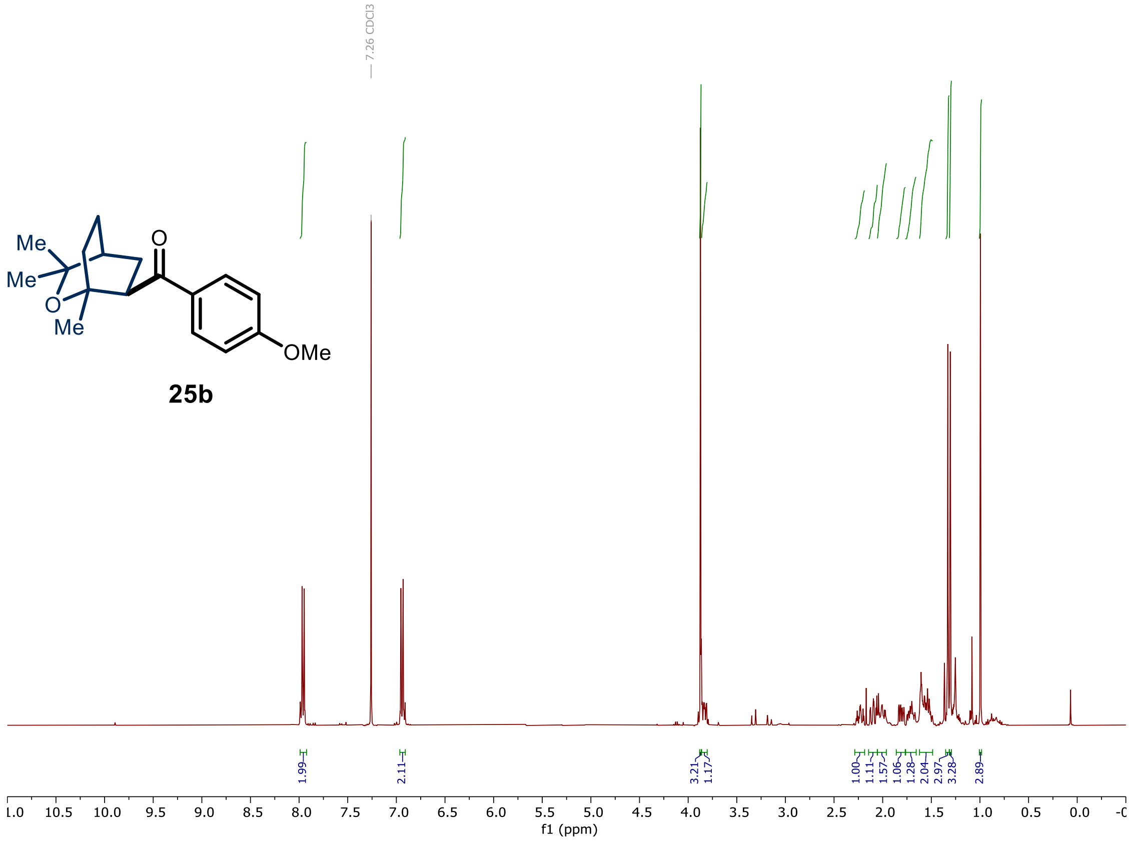

^{13}C NMR (101 MHz, $CDCl_3$)

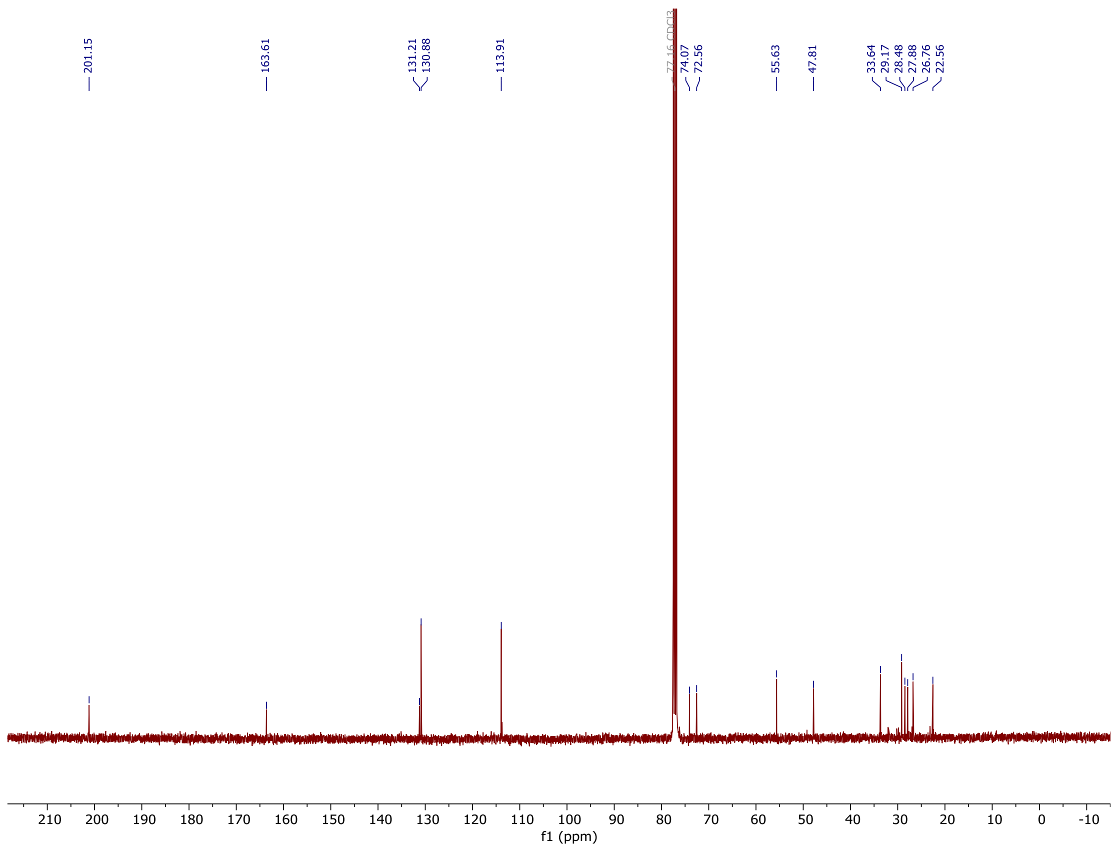

HSQC **25b**

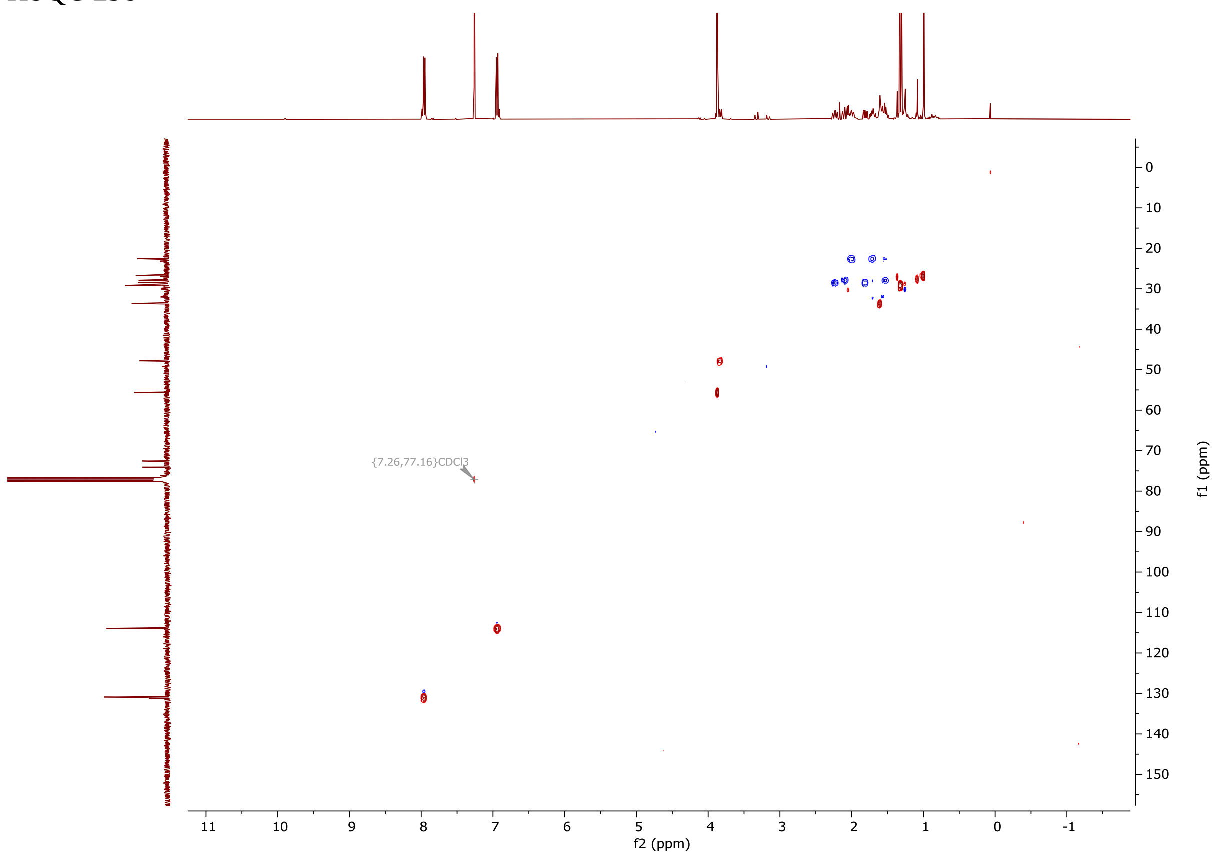

HMBC **25b**

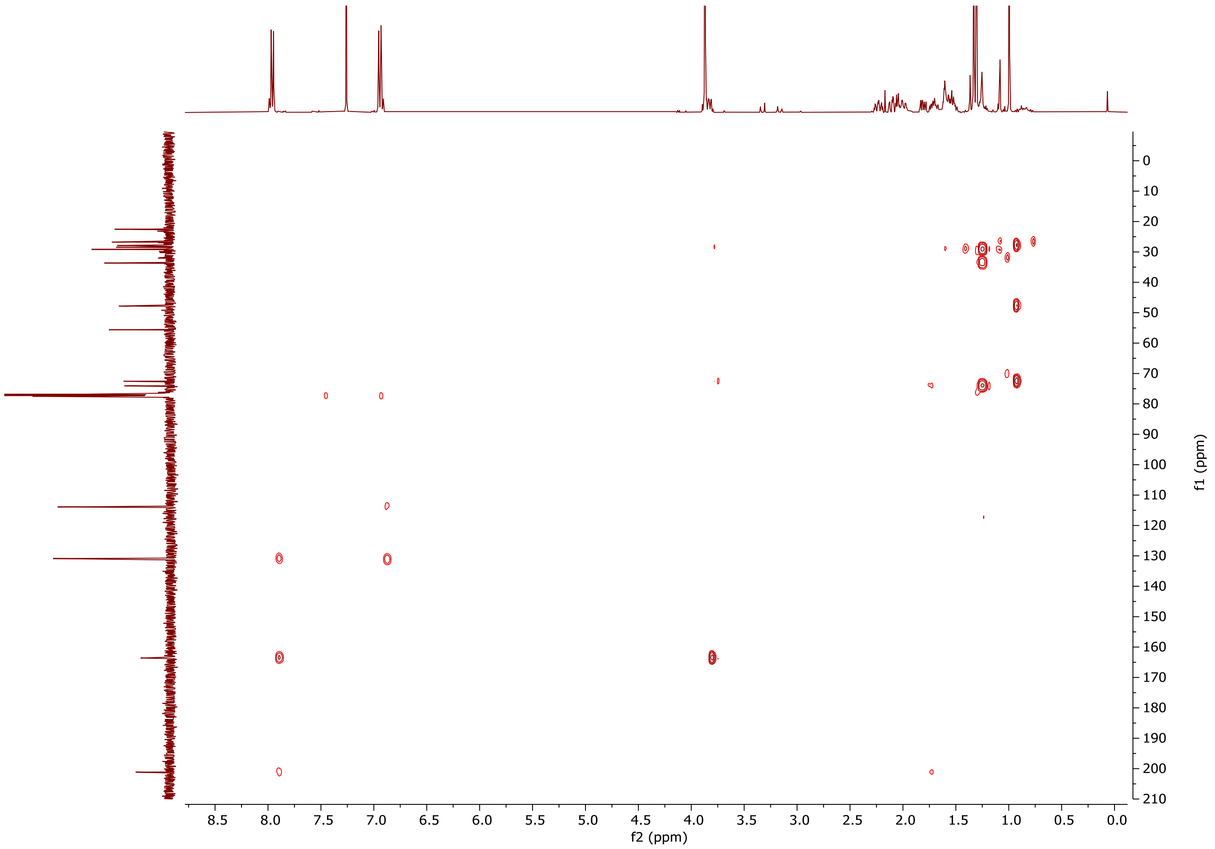

^{1}H NMR (400 MHz, $CDCl_3$)

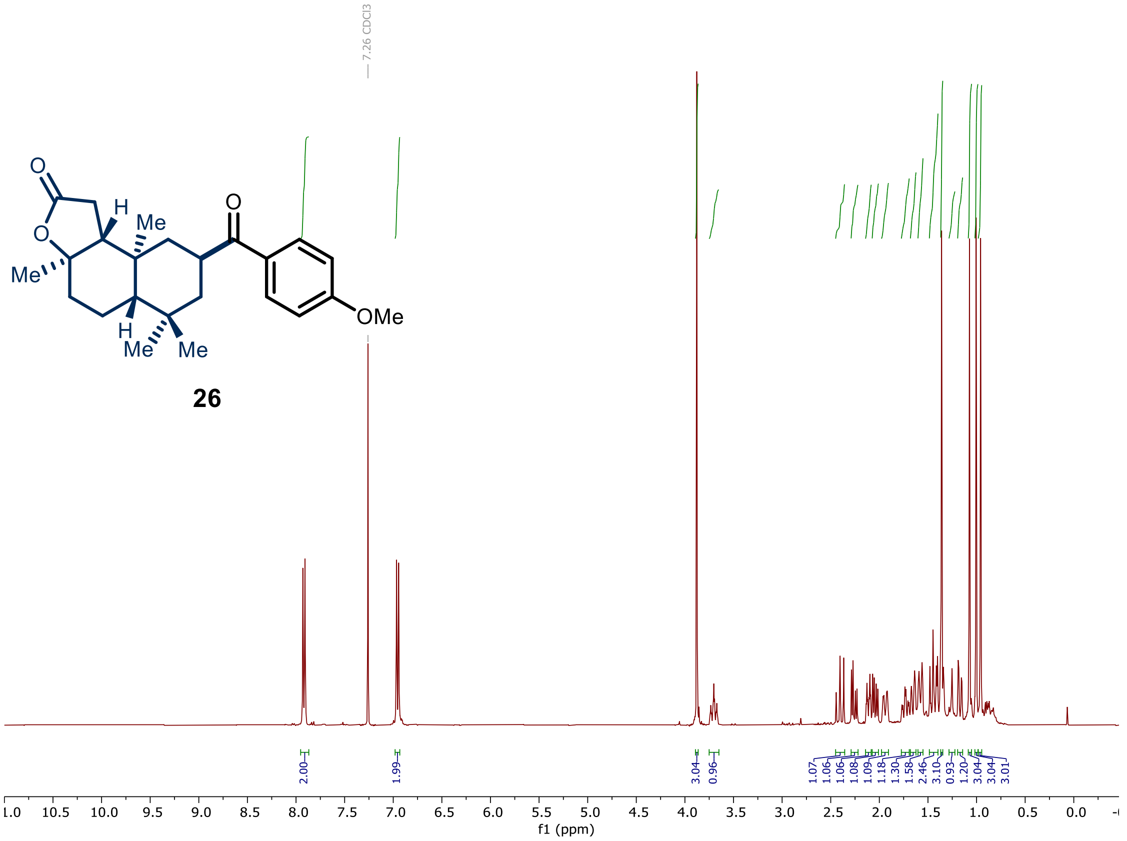

^{13}C NMR (101 MHz, $CDCl_3$)

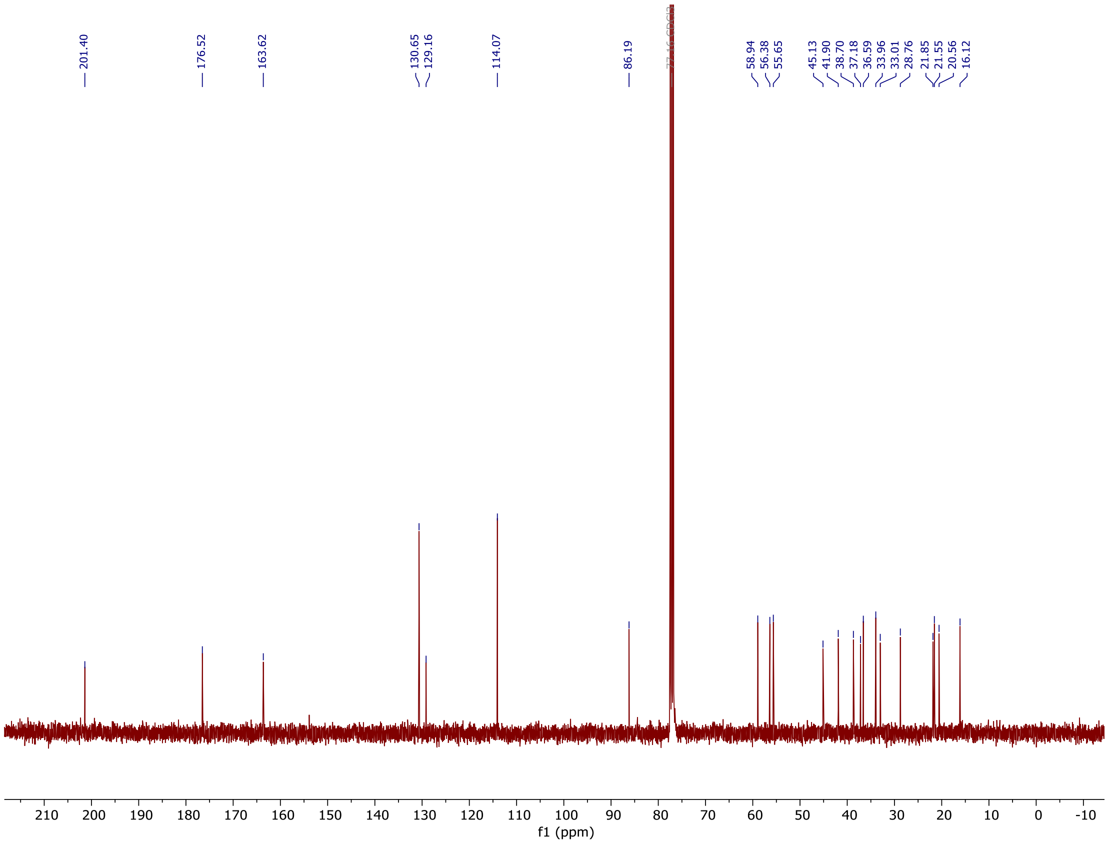

NOESY **26**

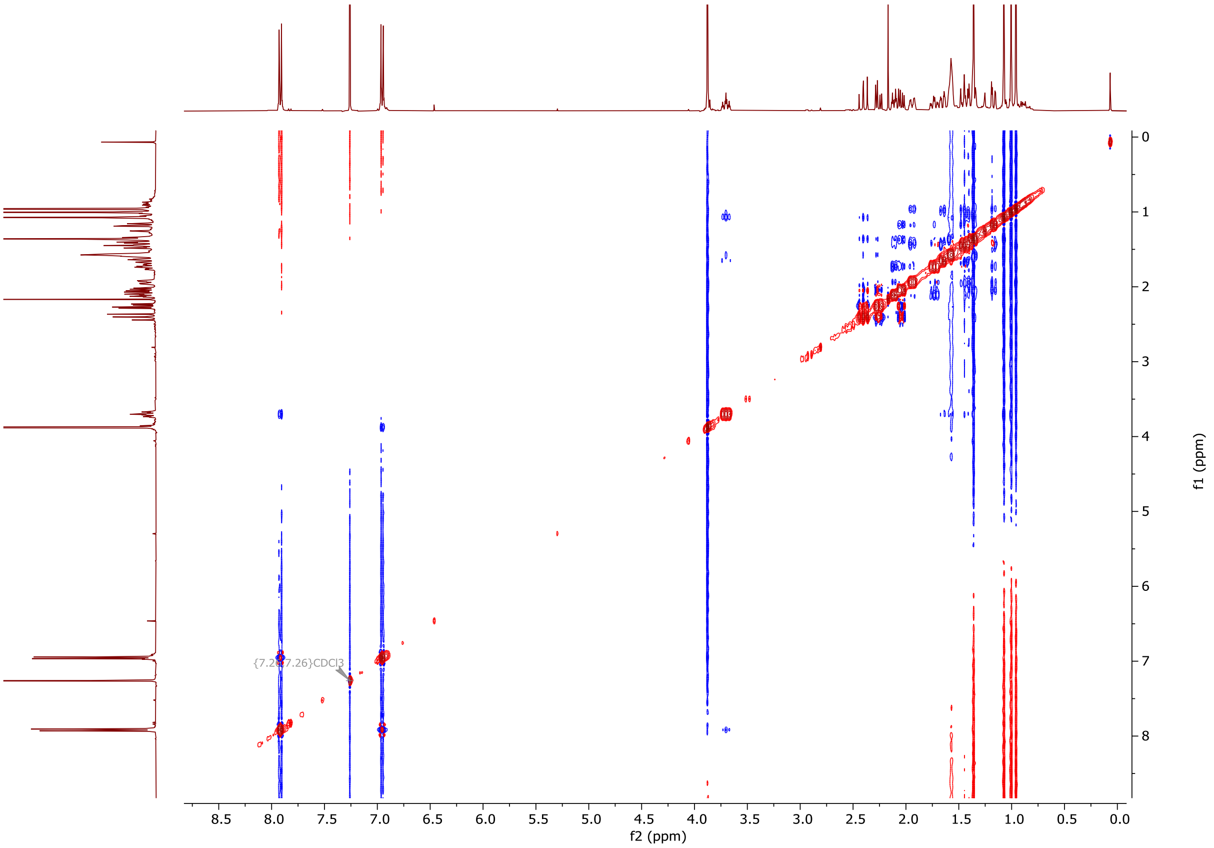

^{1}H NMR (400 MHz, $CDCl_3$)

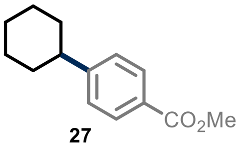

27

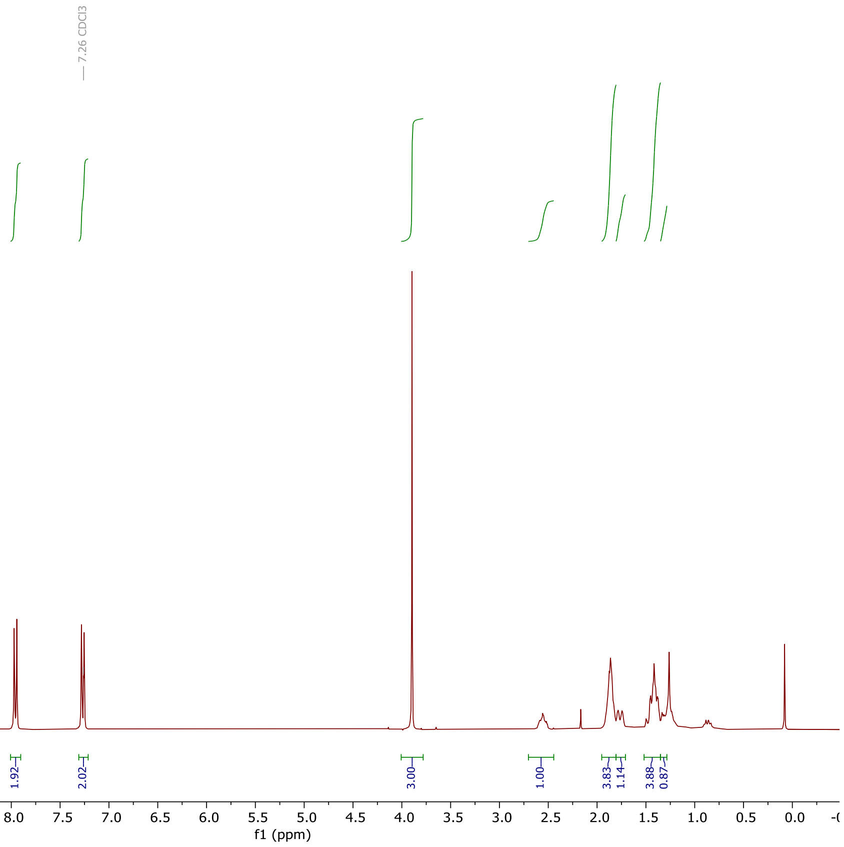

^{13}C NMR (101 MHz, CDCl$_3$)

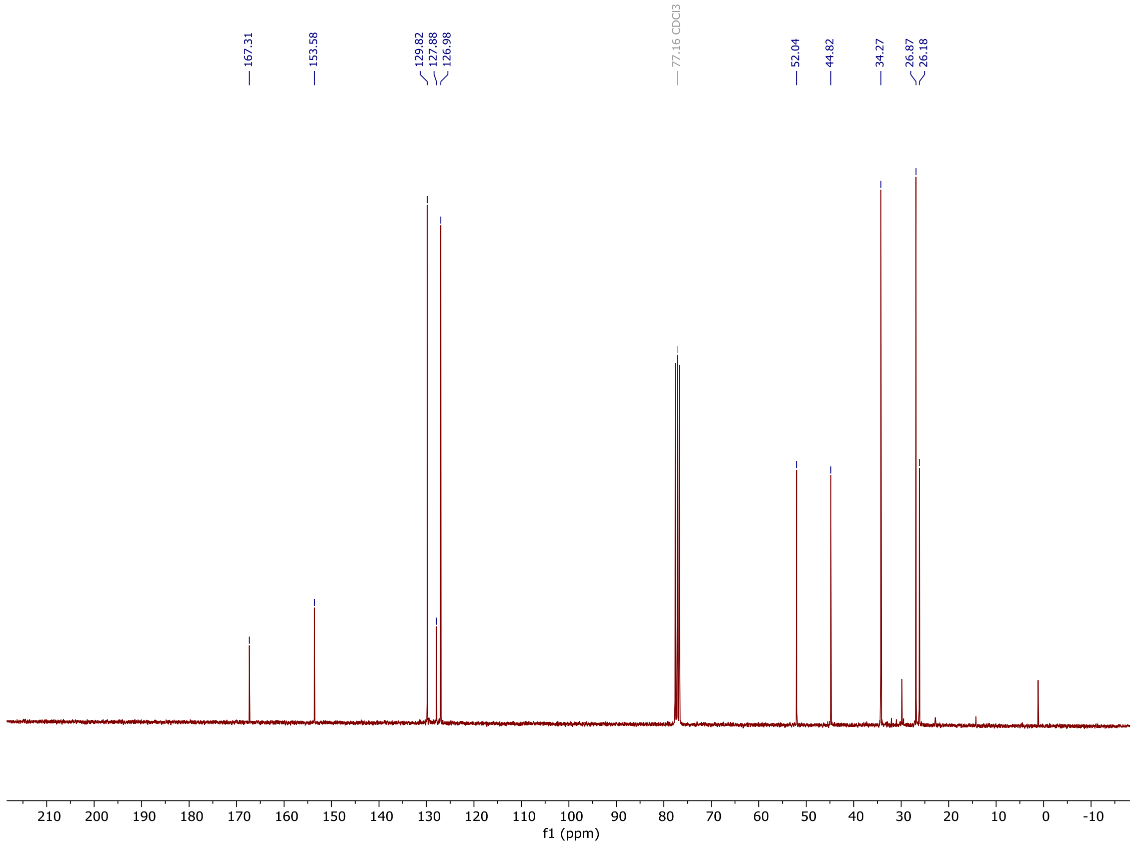

^{1}H NMR (400 MHz, CDCl$_3$)

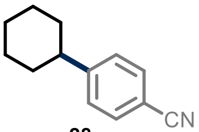

28

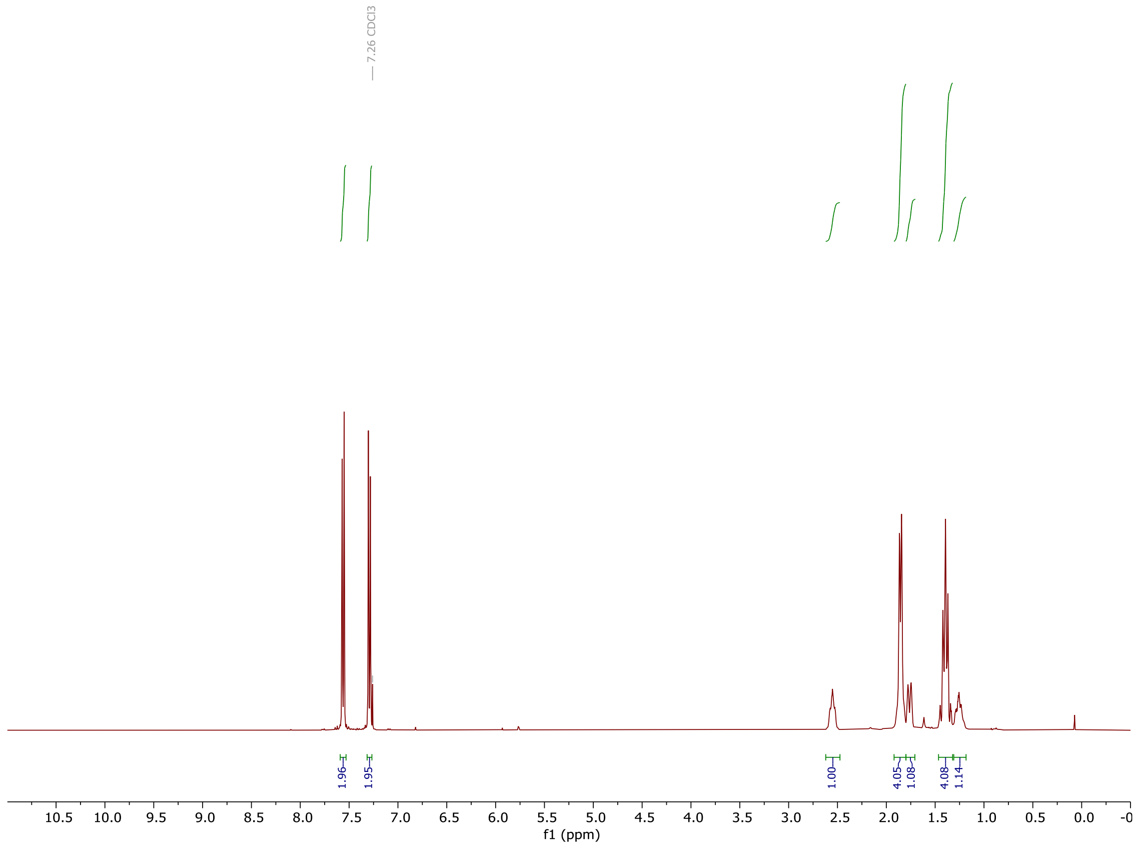

^{13}C NMR (101 MHz, $CDCl_3$)

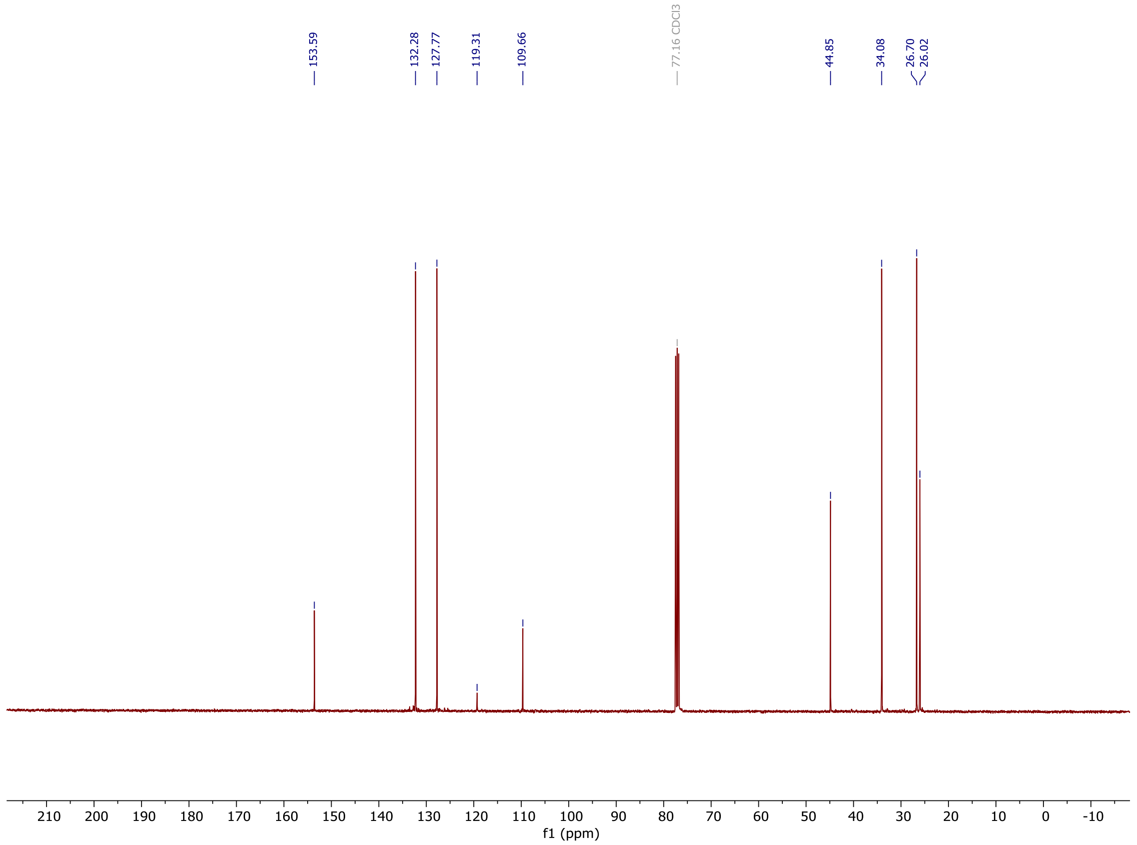

^{1}H NMR (400 MHz, $CDCl_3$)

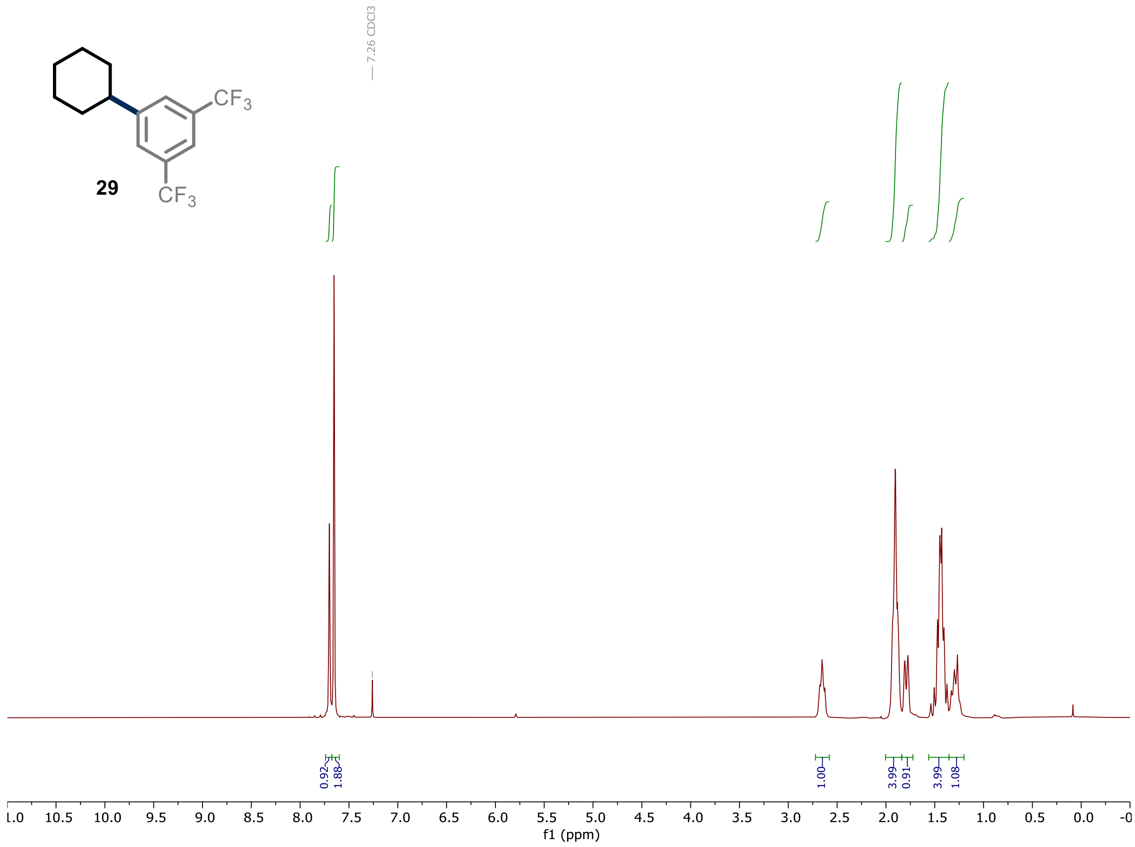

^{13}C NMR (101 MHz, $CDCl_3$)

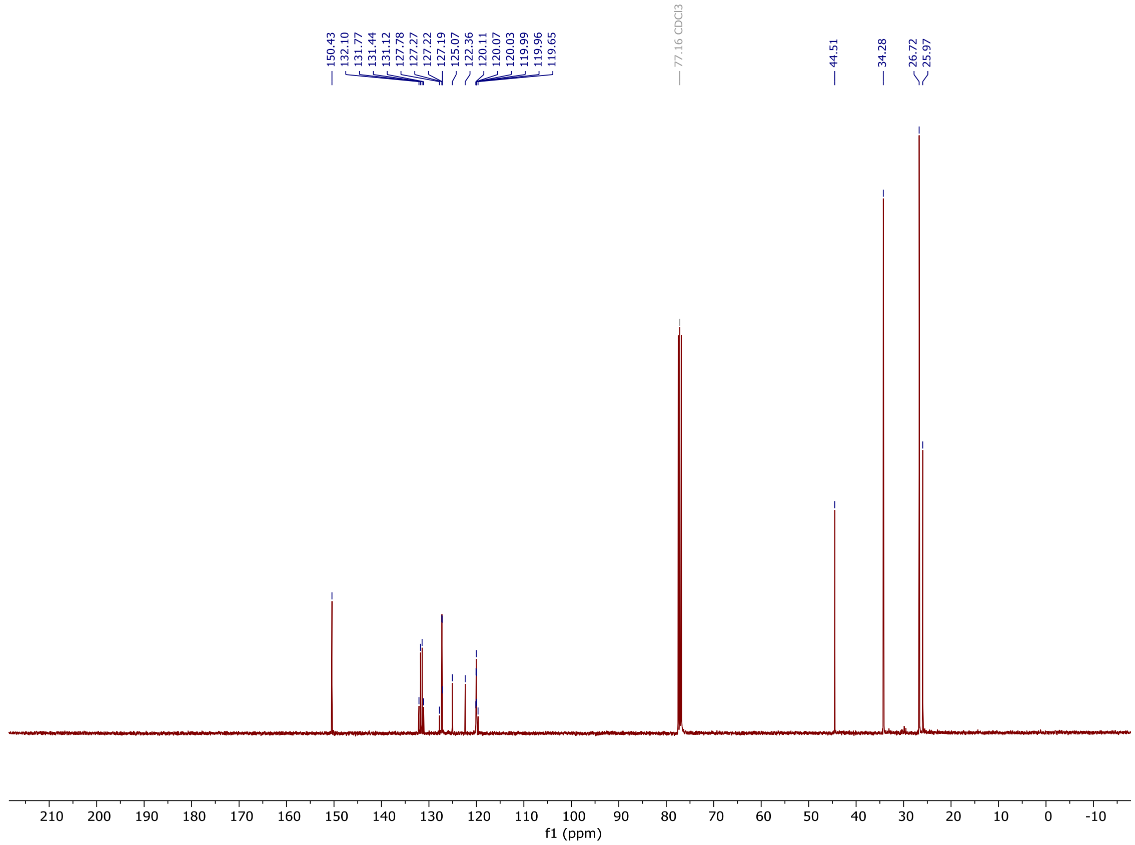

^{19}F NMR (376 MHz, $CDCl_3$)

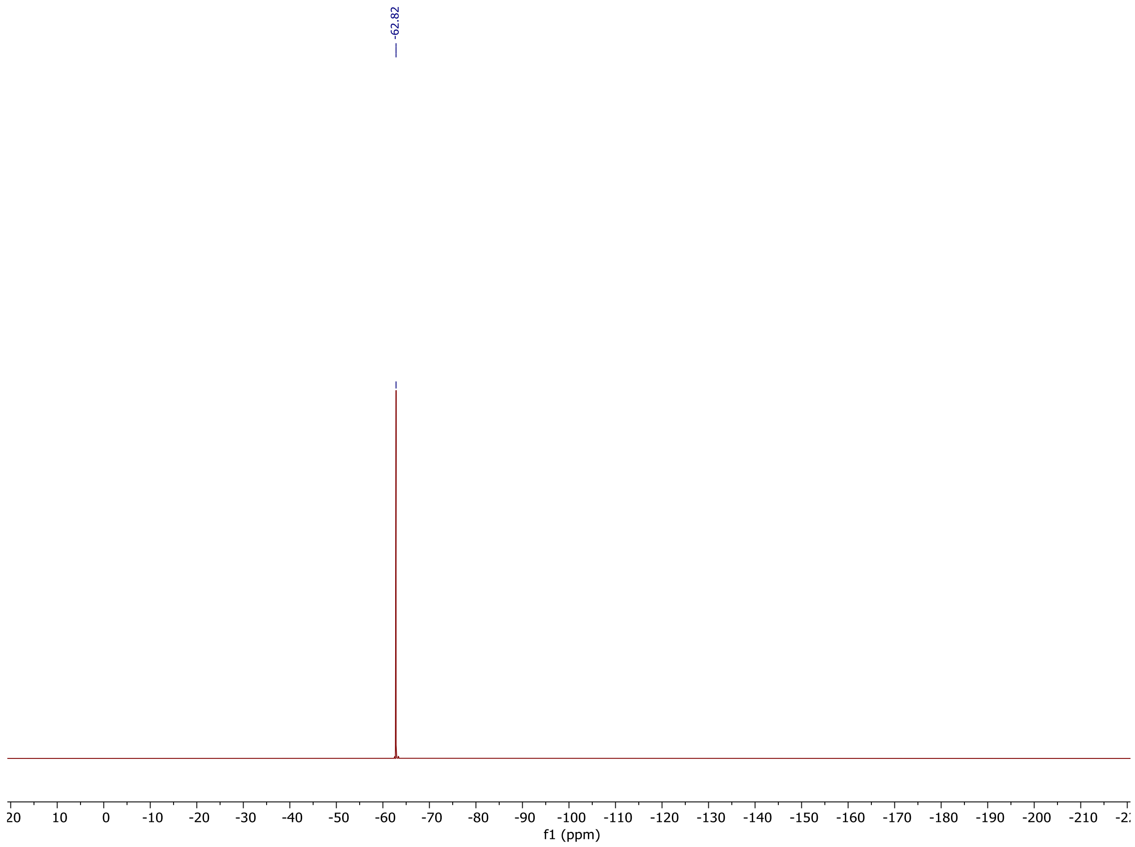

^{1}H NMR (400 MHz, $CDCl_3$)

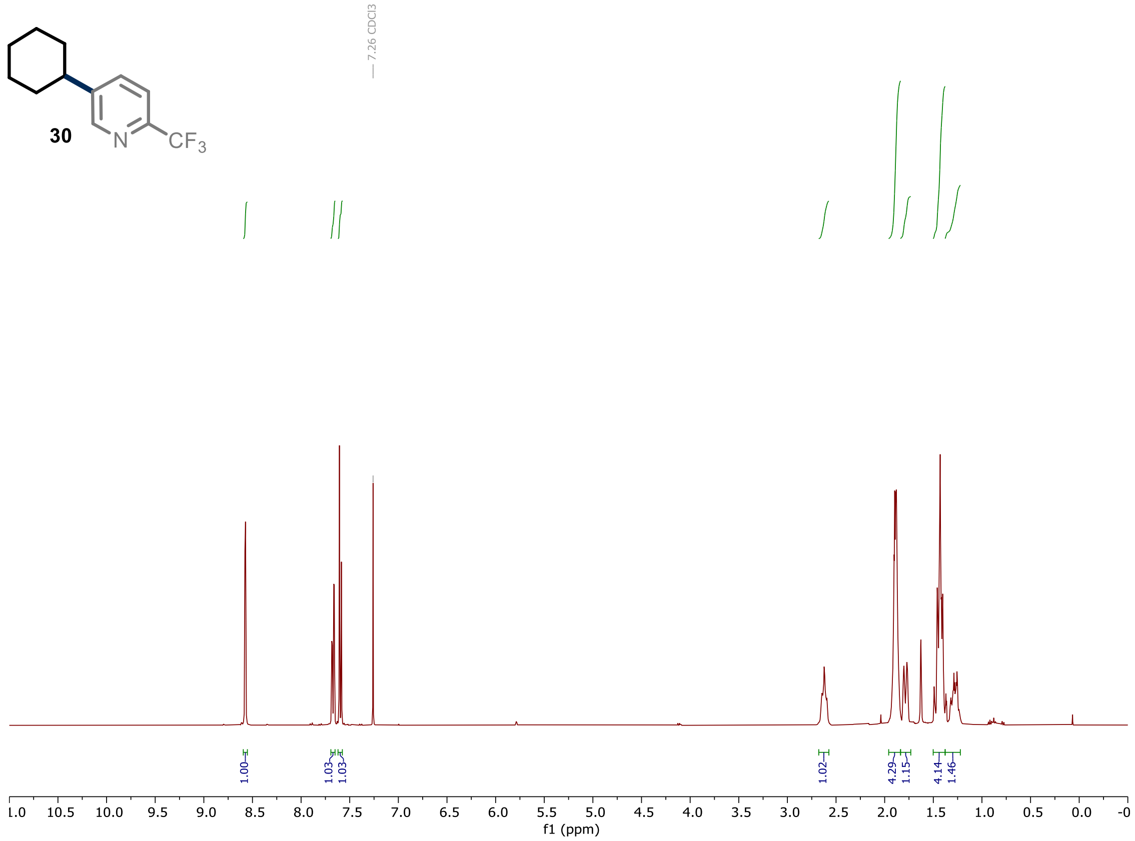

^{13}C NMR (101 MHz, $CDCl_3$)

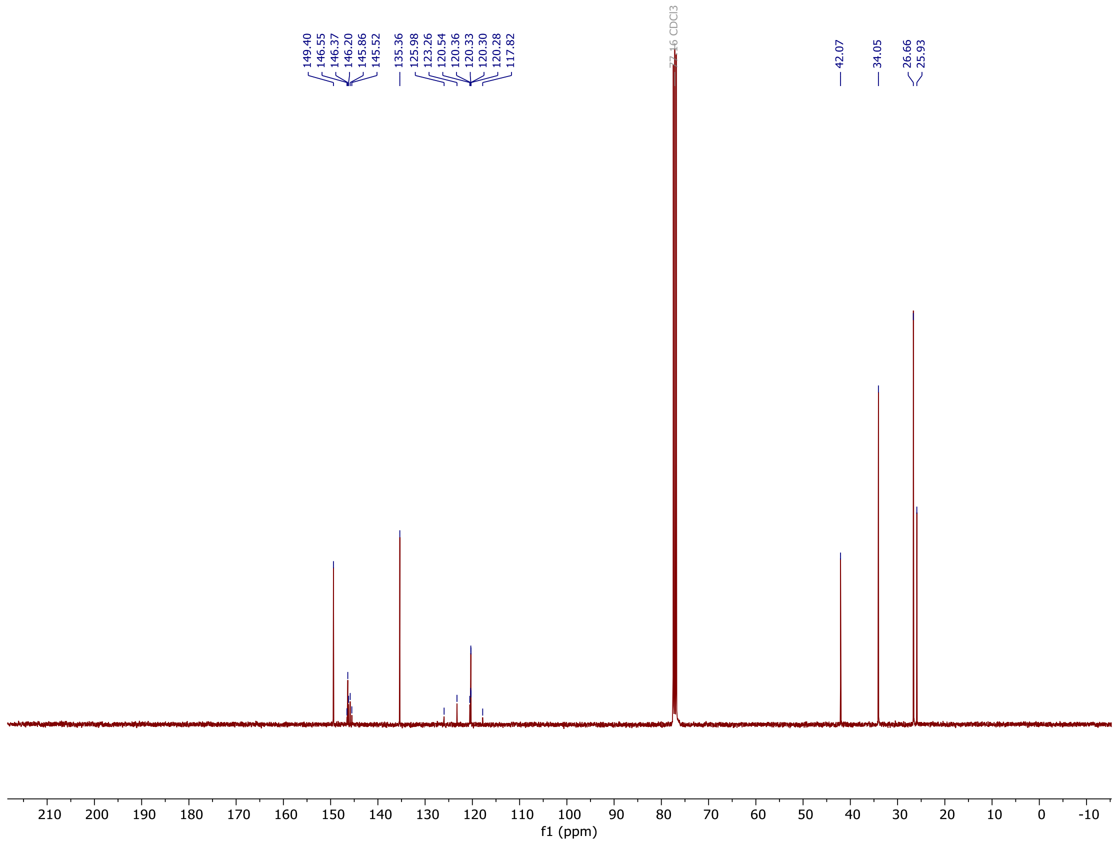

^{19}F NMR (376 MHz, $CDCl_3$)

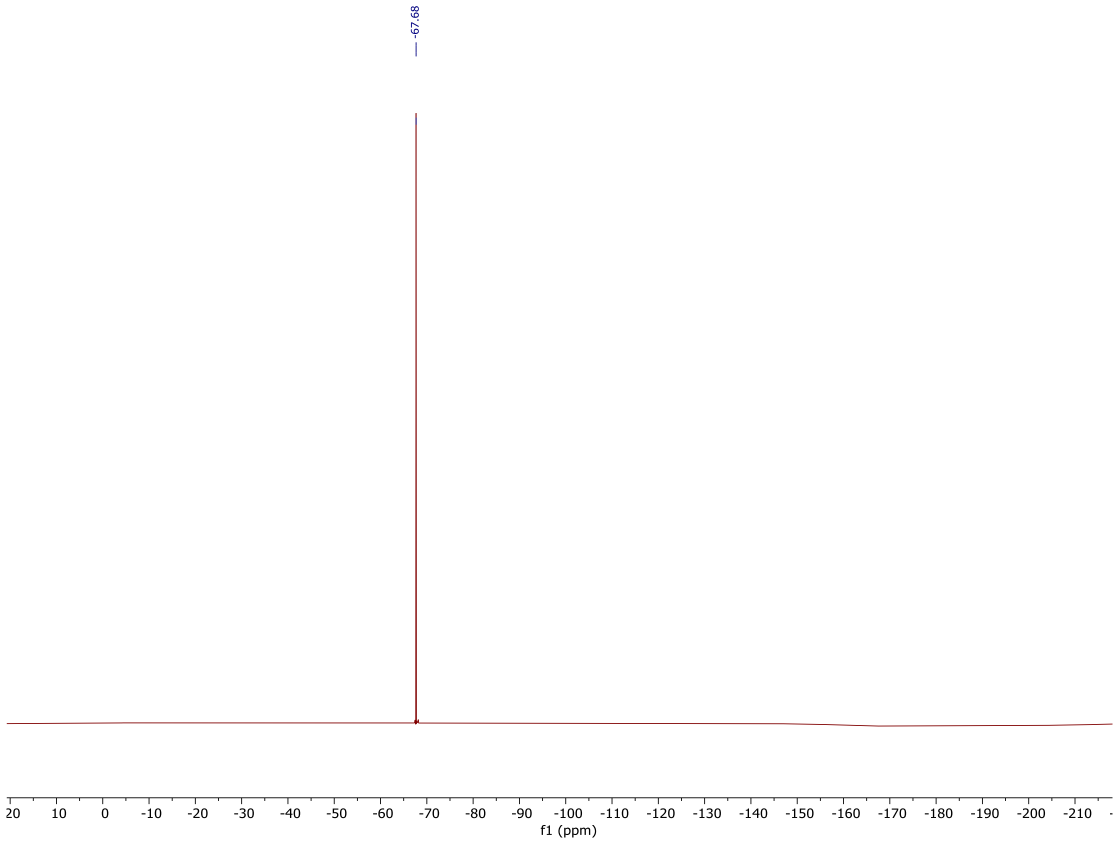

^{1}H NMR (400 MHz, $CDCl_3$)

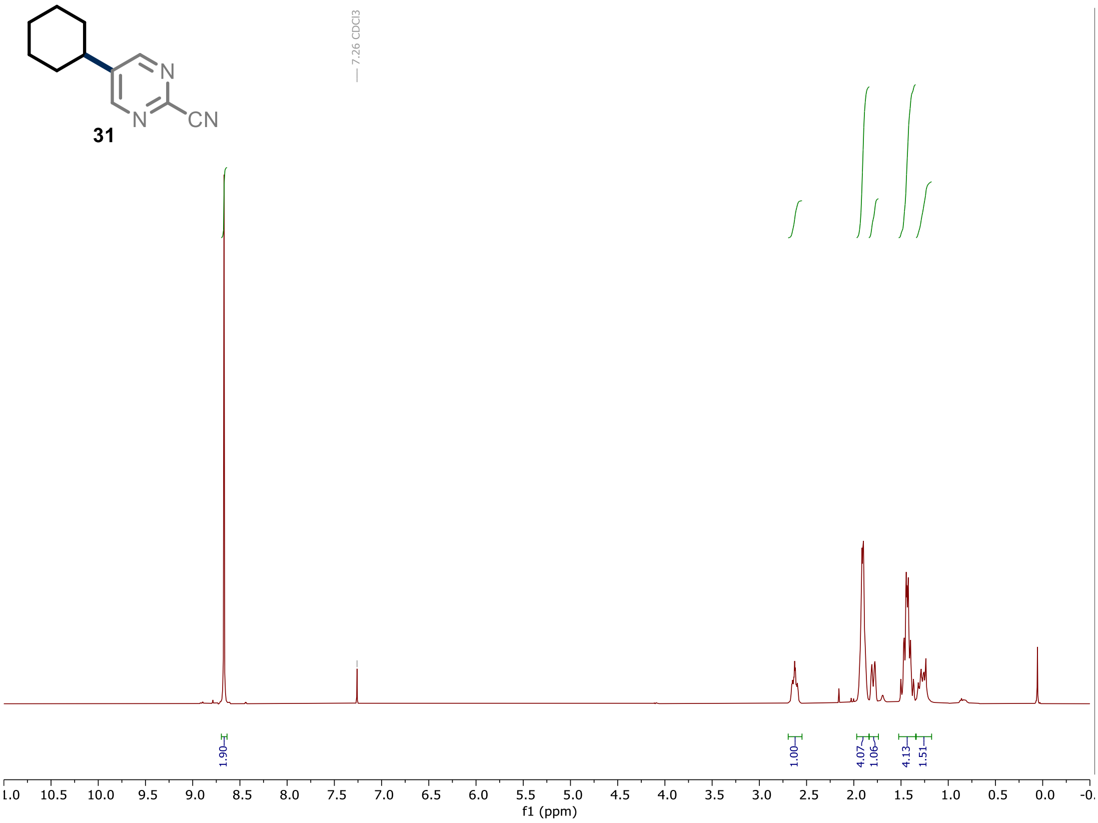

^{13}C NMR (101 MHz, $CDCl_3$)

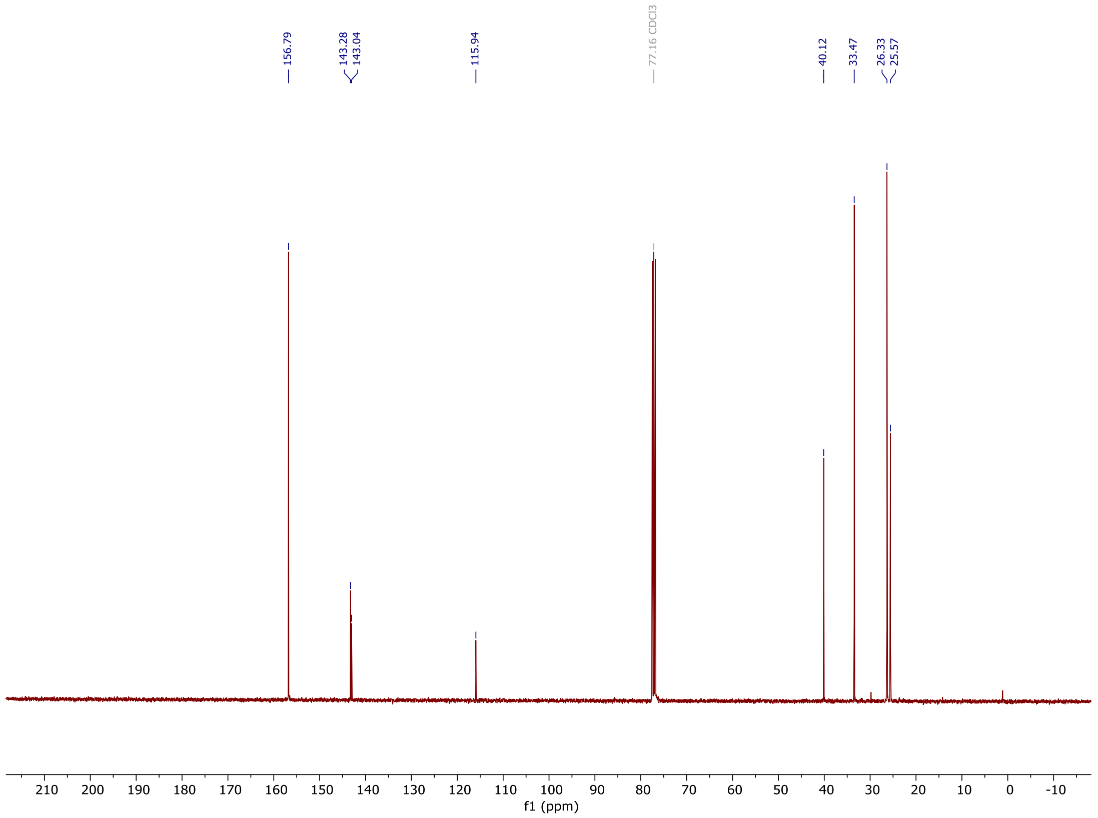

^{1}H NMR (400 MHz, $CDCl_3$)

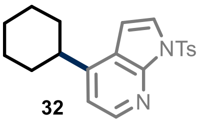

32

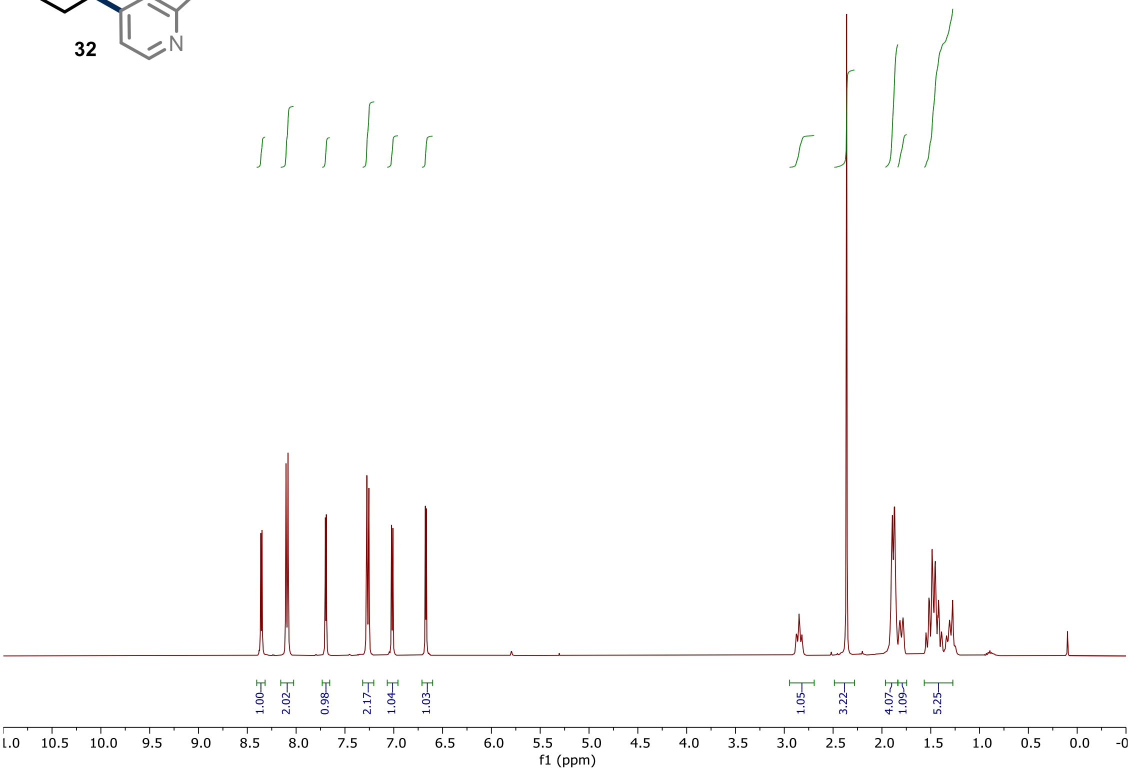

^{13}C NMR (101 MHz, $CDCl_3$)

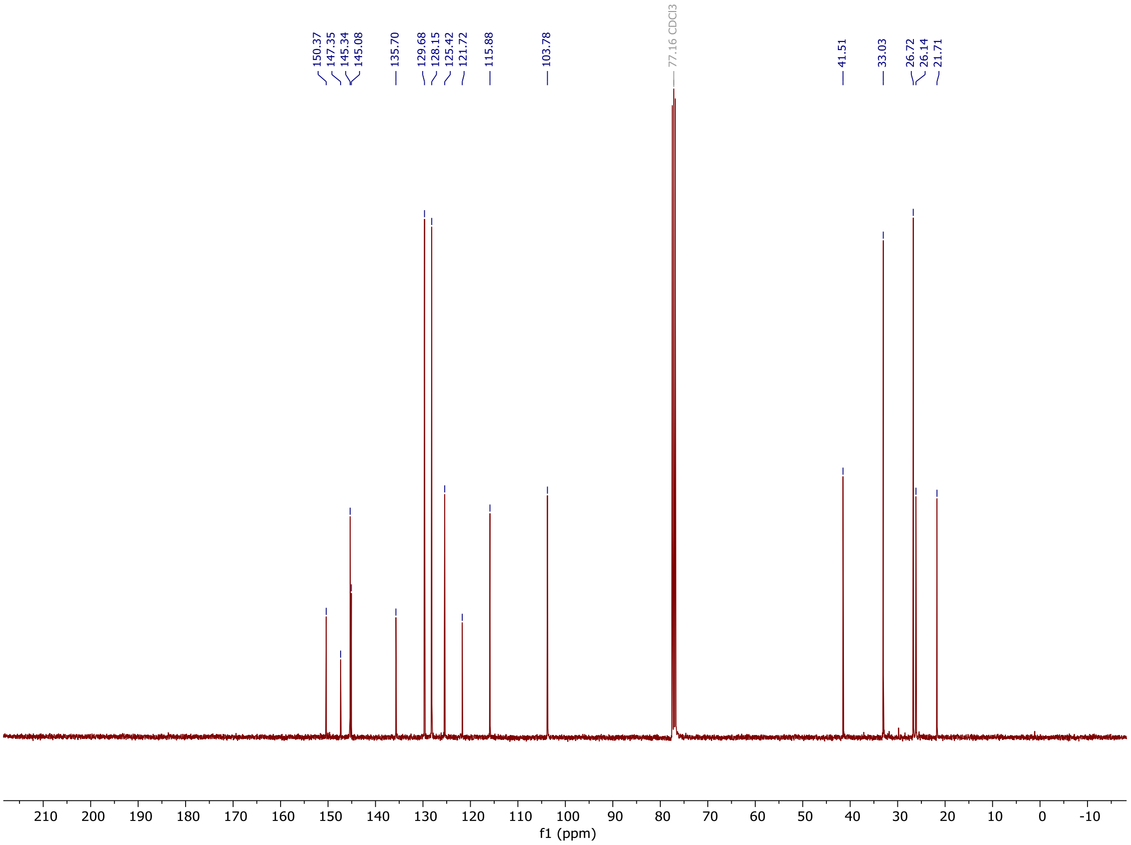

^{1}H NMR (400 MHz, $CDCl_3$)

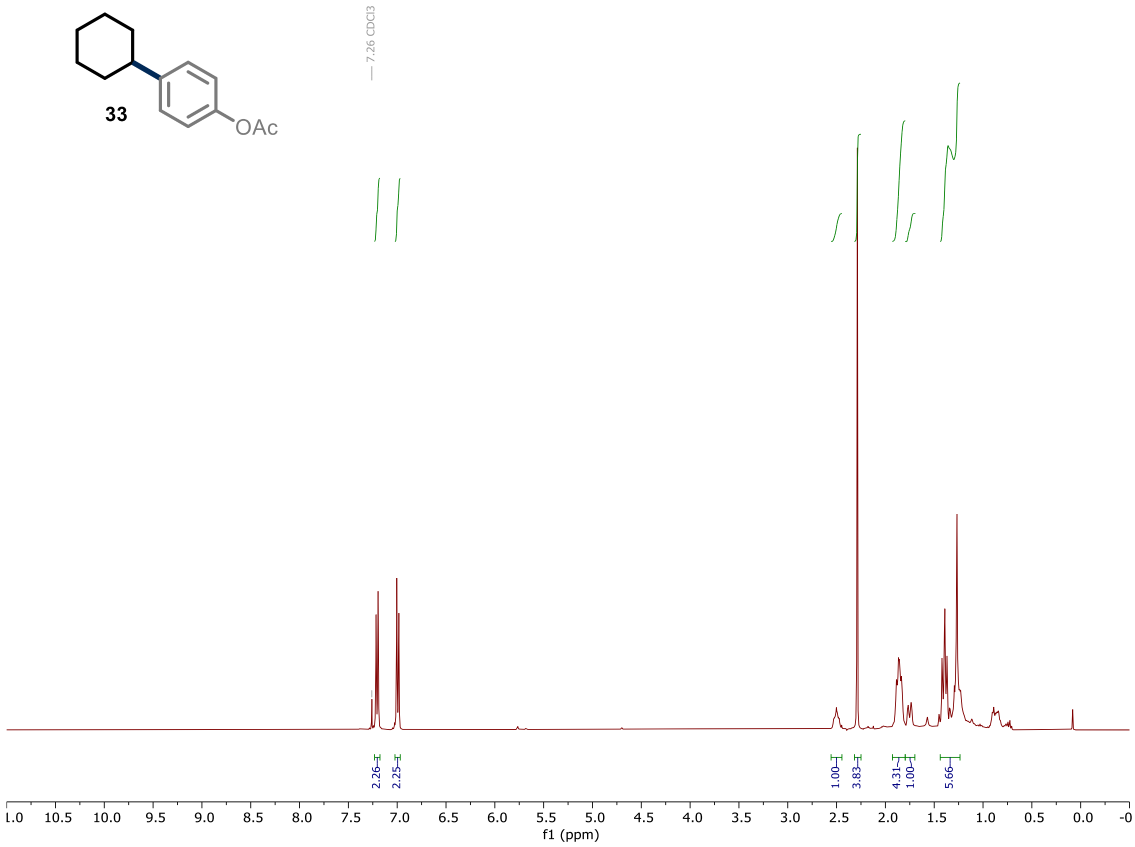

^{13}C NMR (101 MHz, $CDCl_3$)

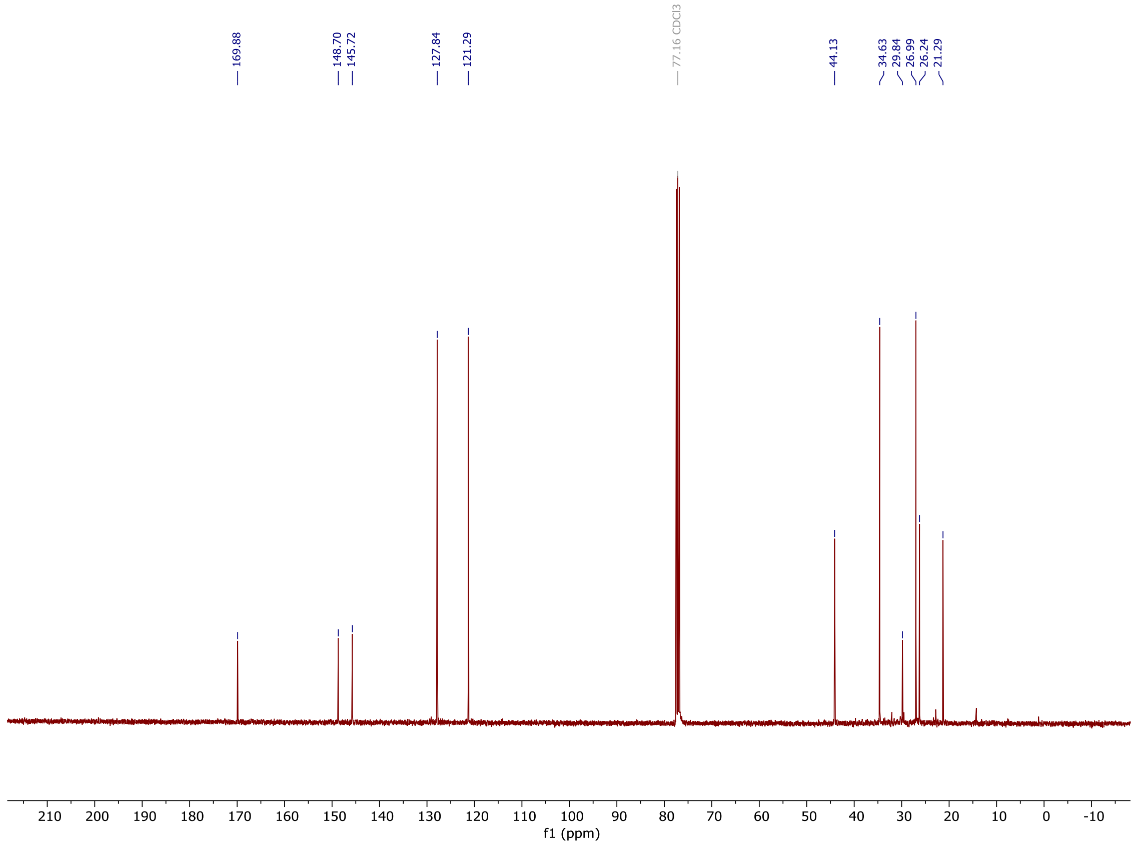

^{1}H NMR (400 MHz, $CDCl_3$)

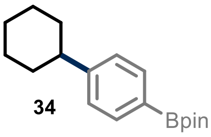

34

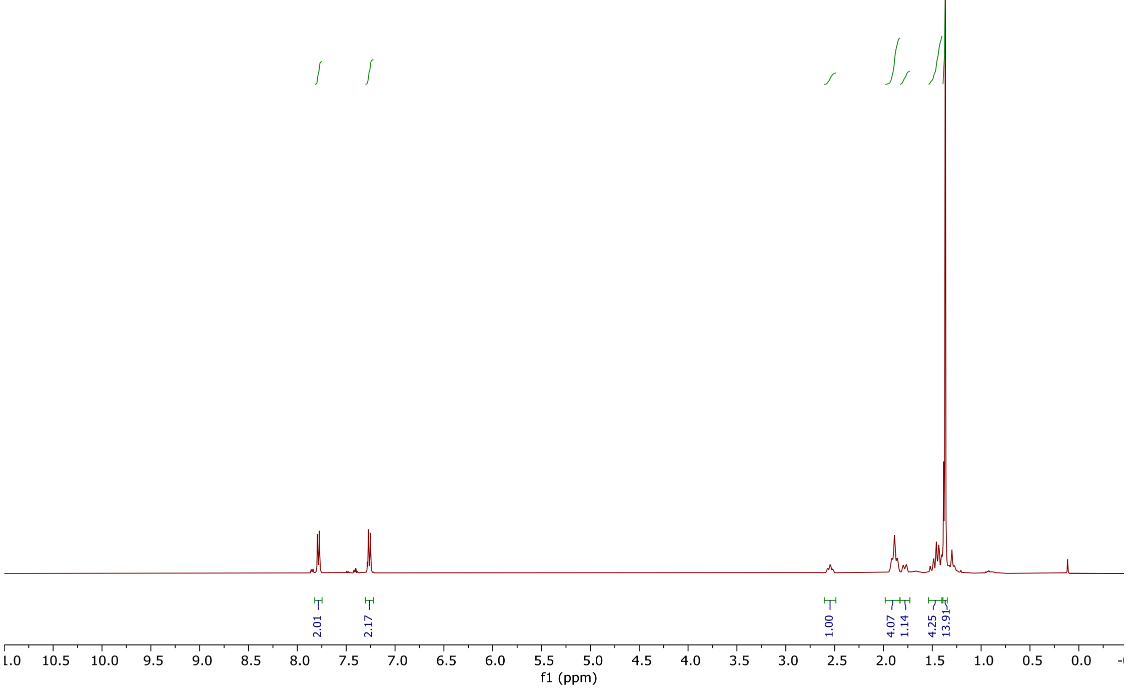

^{13}C NMR (101 MHz, $CDCl_3$)

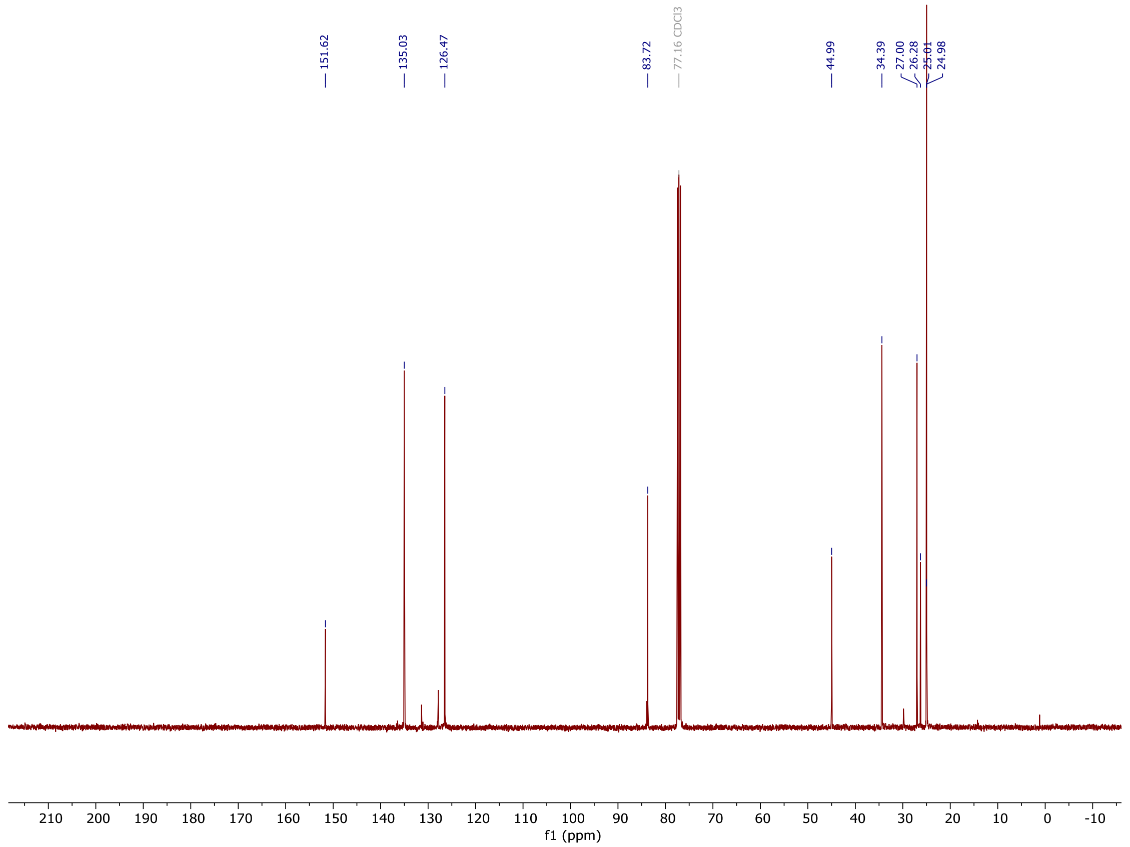

^{11}B NMR (128 MHz, $CDCl_3$)

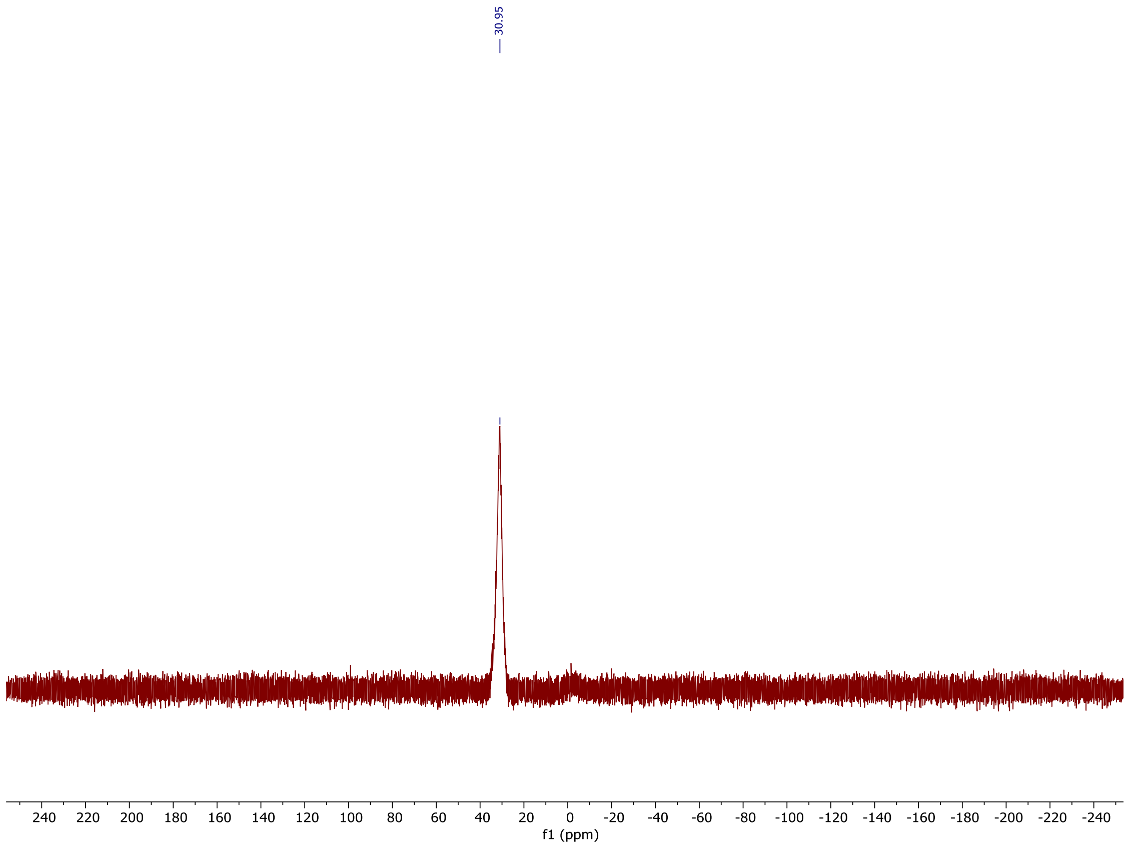

^{1}H NMR (400 MHz, $CDCl_3$)

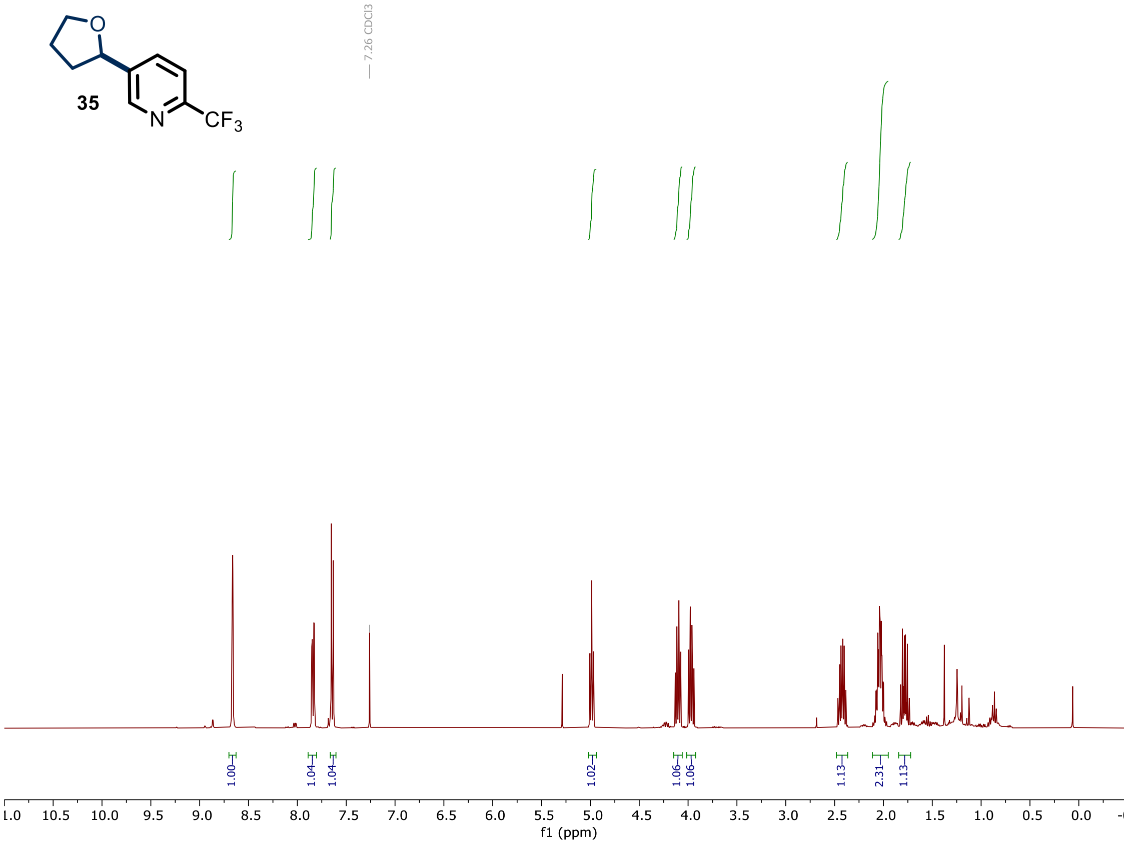

^{13}C NMR (101 MHz, $CDCl_3$)

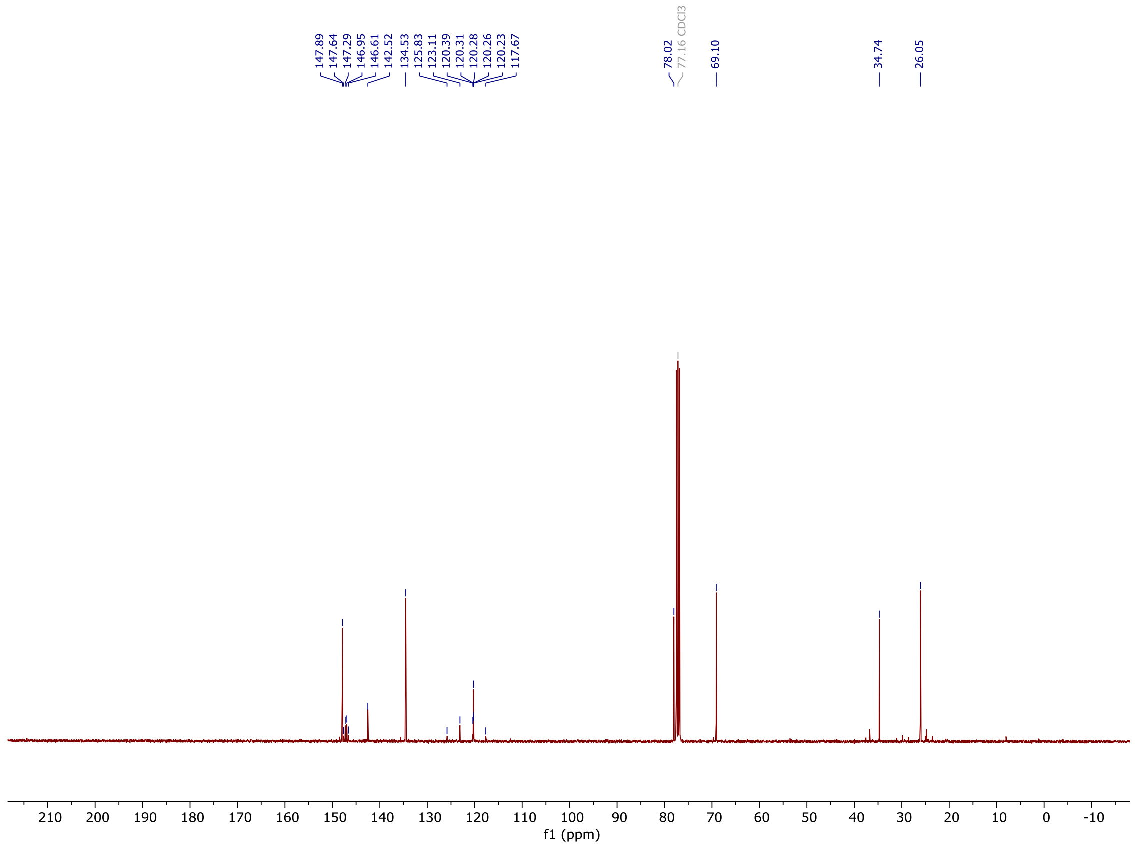

^{19}F NMR (376 MHz, $CDCl_3$)

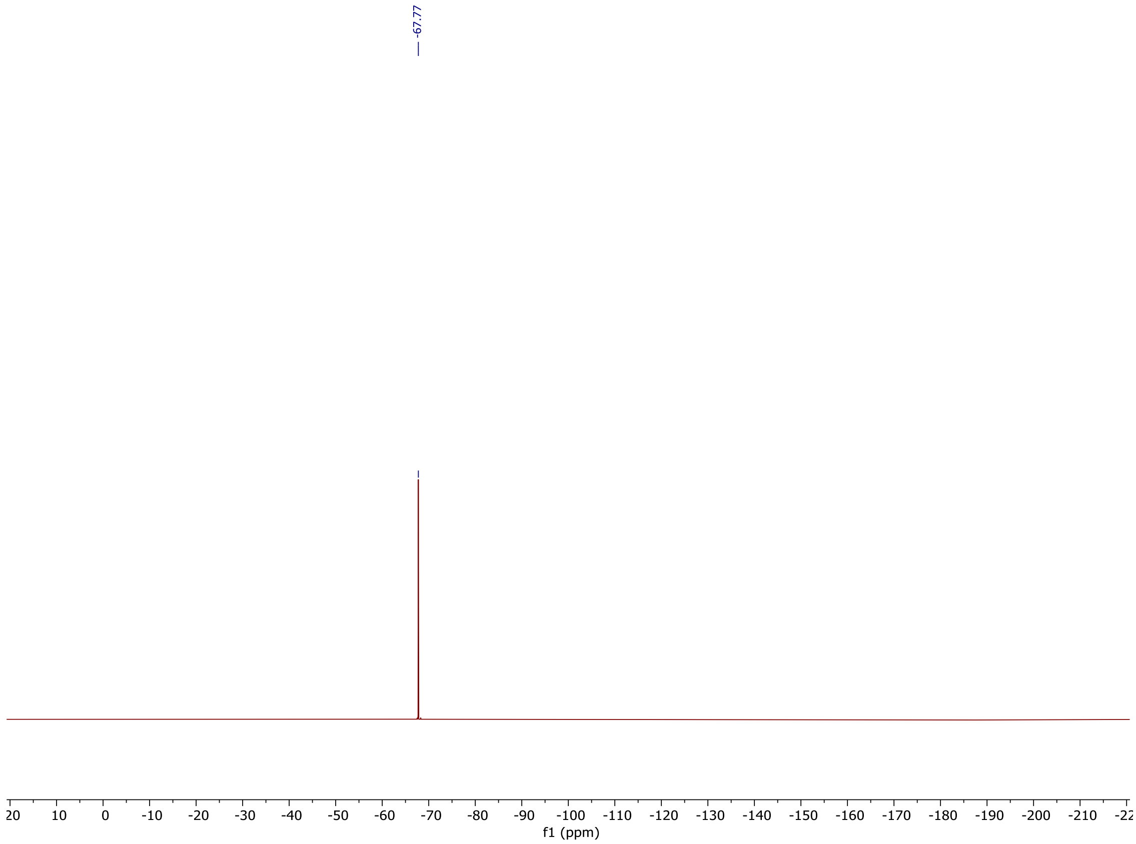

^{1}H NMR (400 MHz, $CDCl_3$)

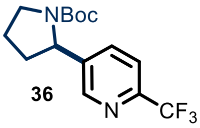

36

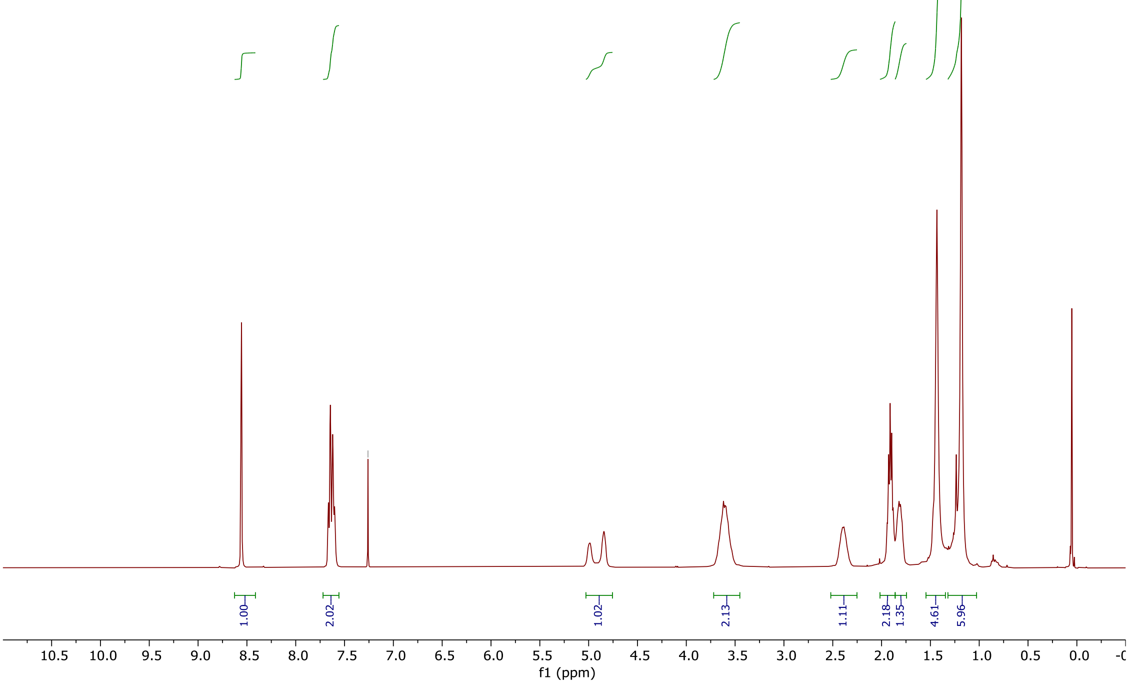

^{13}C NMR (128 MHz, $CDCl_3$)

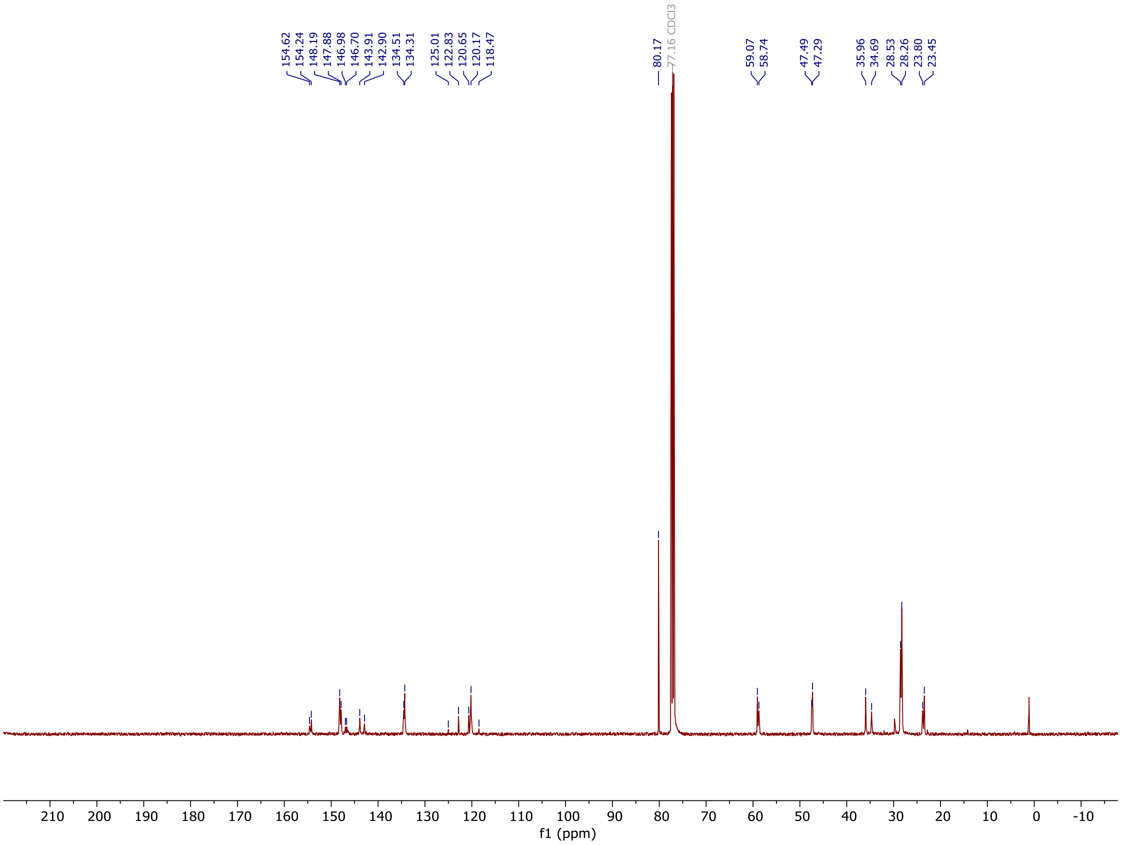

^{19}F NMR (376 MHz, $CDCl_3$)

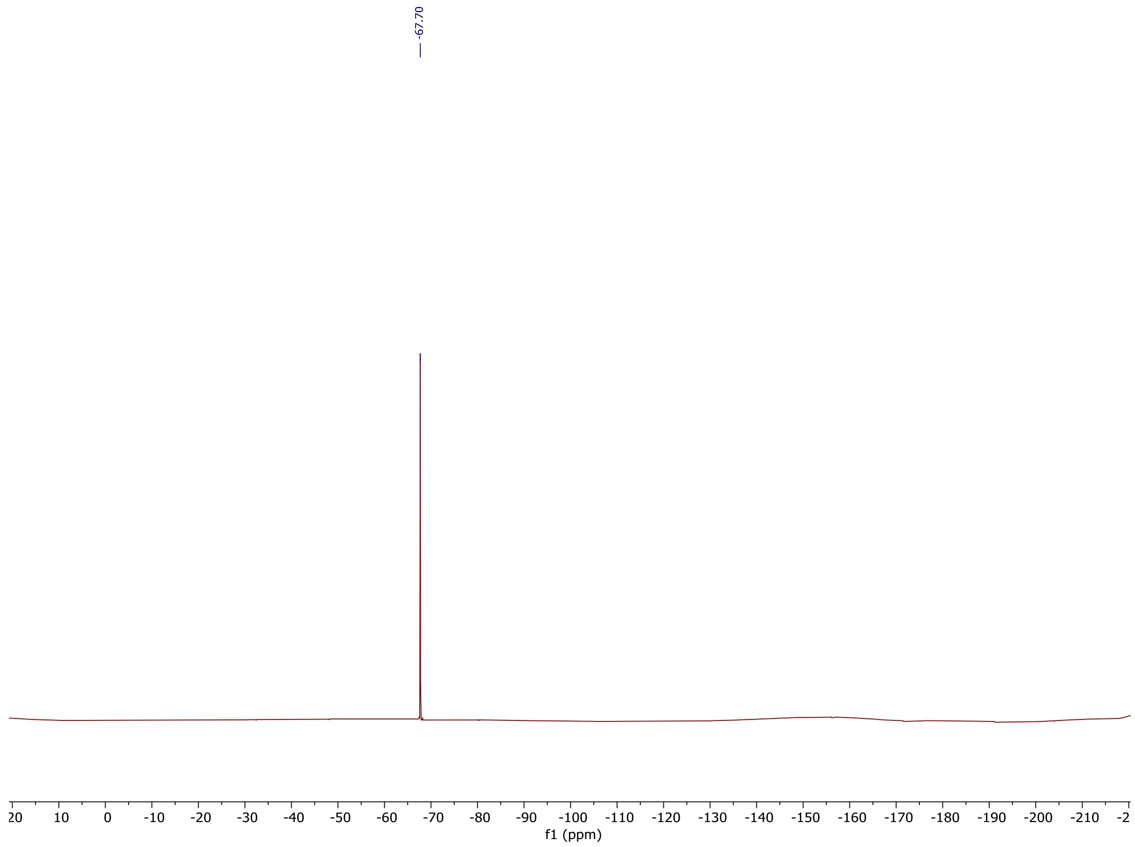

^{1}H NMR (400 MHz, $CDCl_3$)

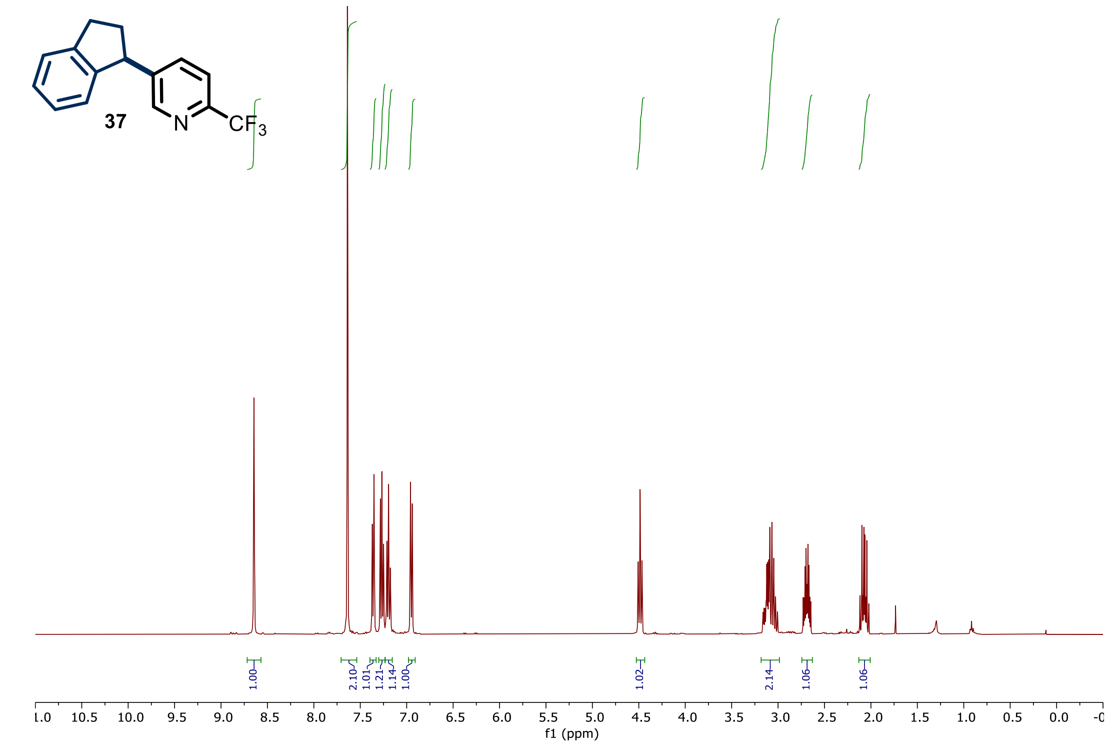

^{13}C NMR (101 MHz, $CDCl_3$)

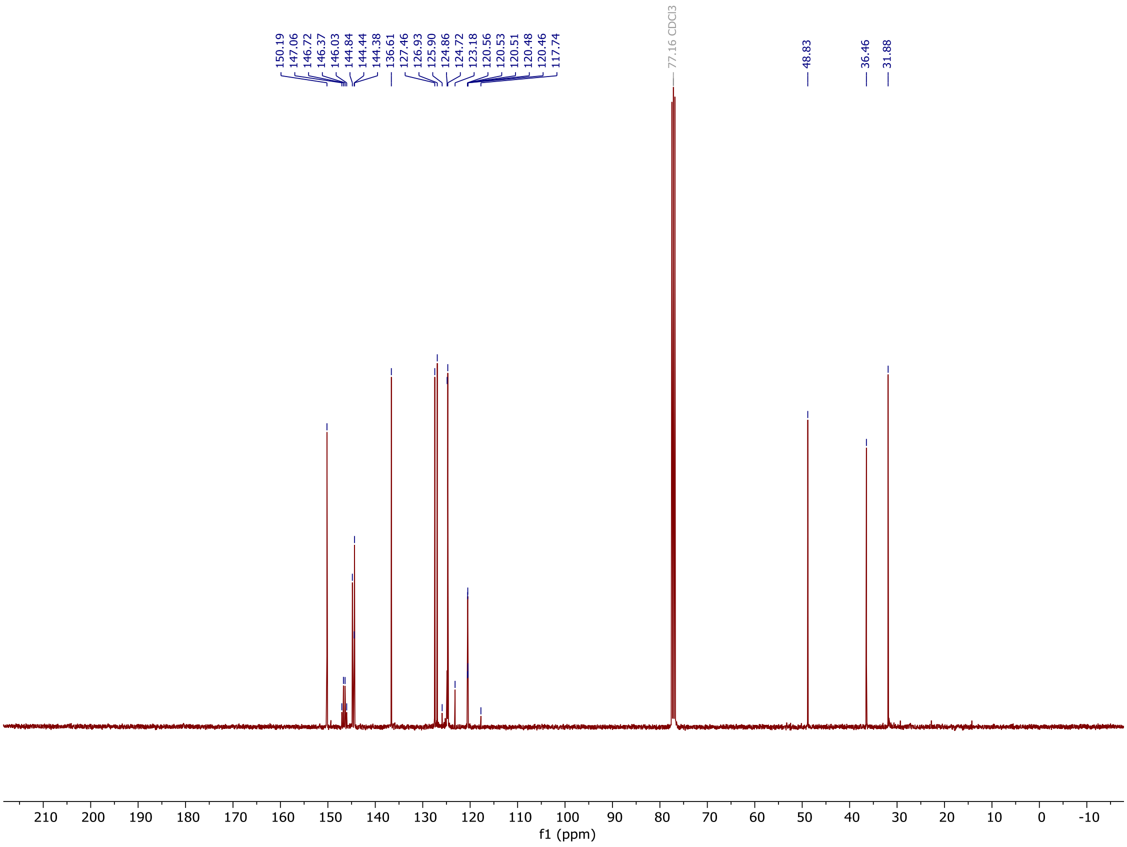

^{19}F NMR (376 MHz, $CDCl_3$)

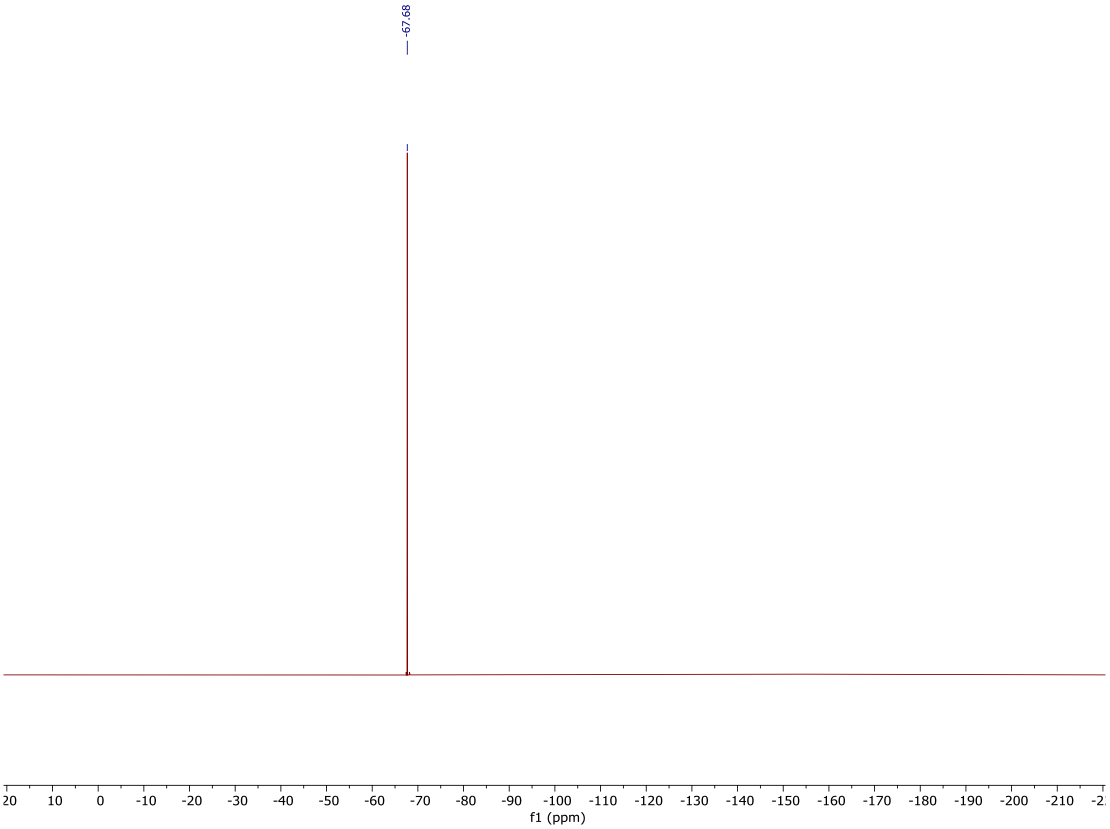

^{1}H NMR (400 MHz, $CDCl_3$)

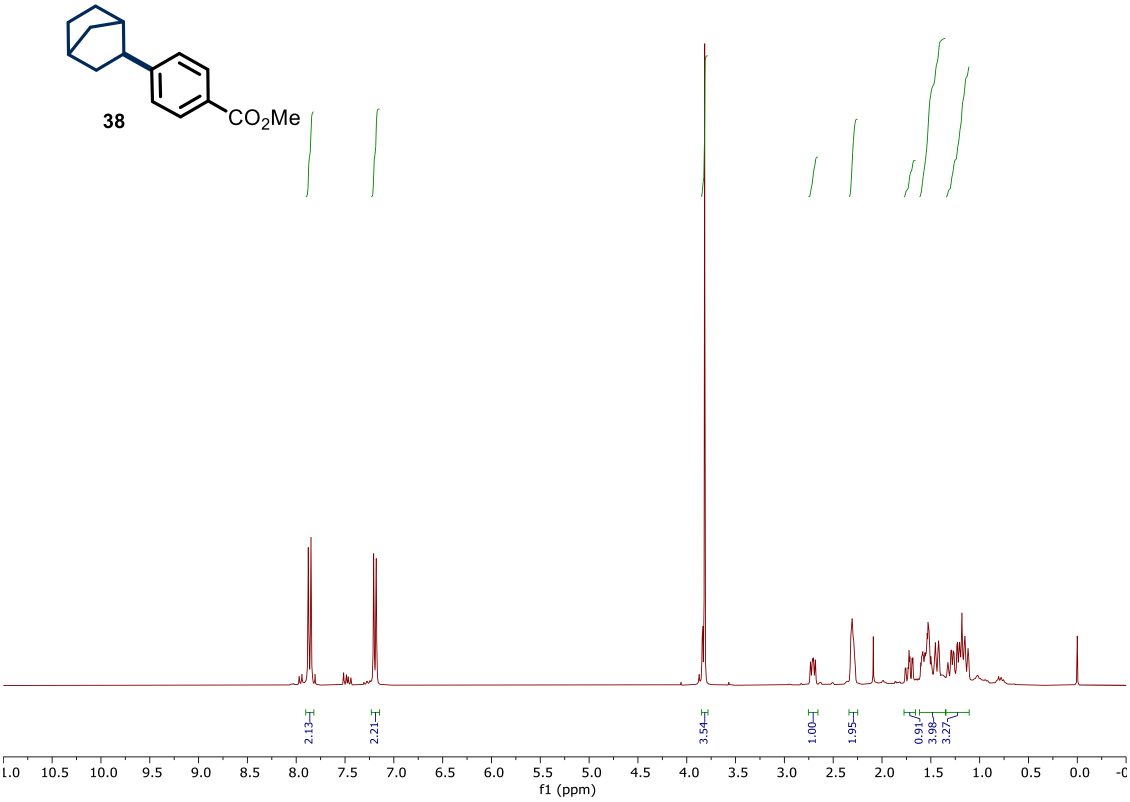

^{13}C NMR (101 MHz, $CDCl_3$)

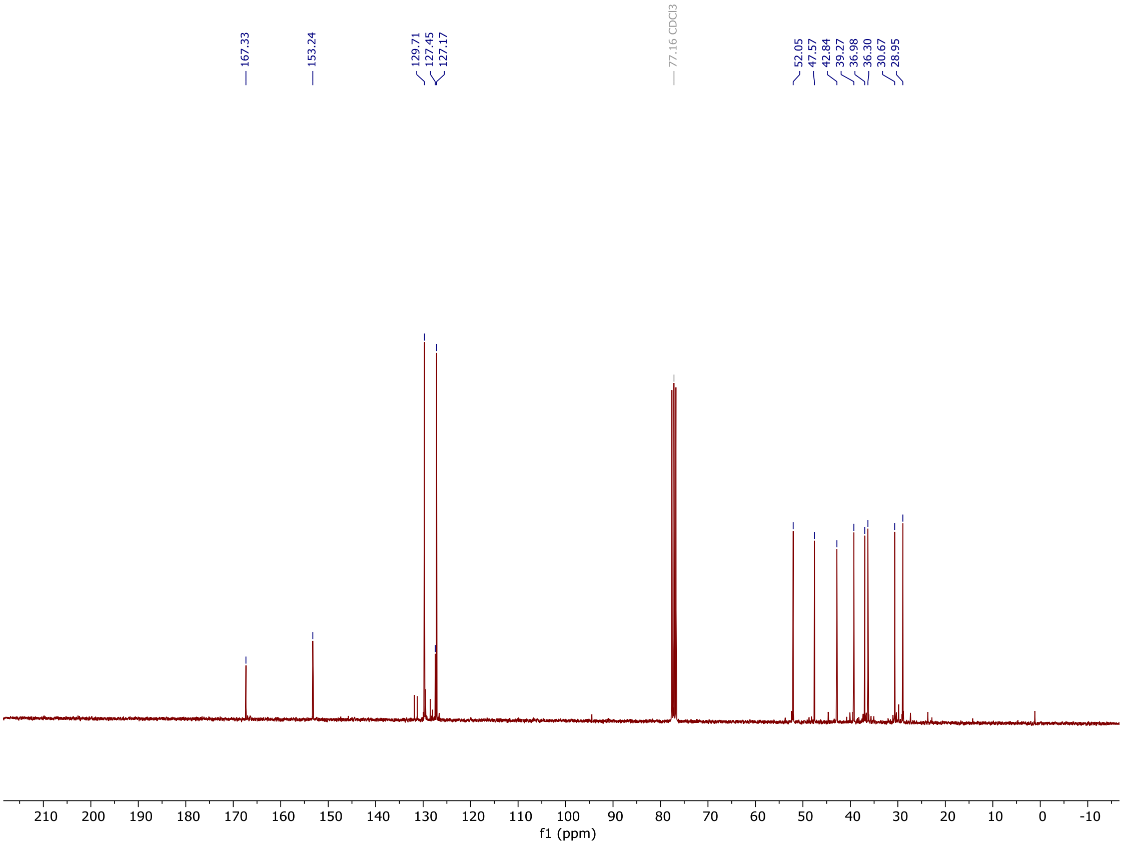

^{1}H NMR (400 MHz, $CDCl_3$)

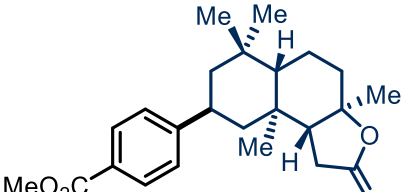

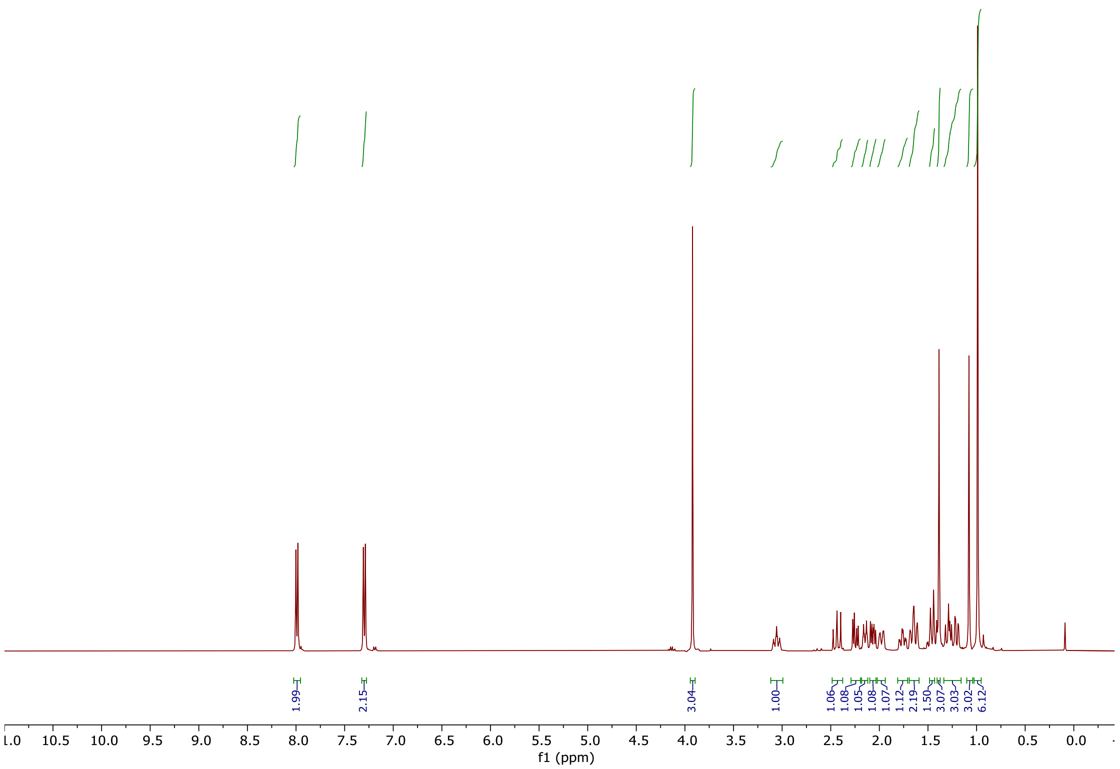

^{13}C NMR (101 MHz, $CDCl_3$)

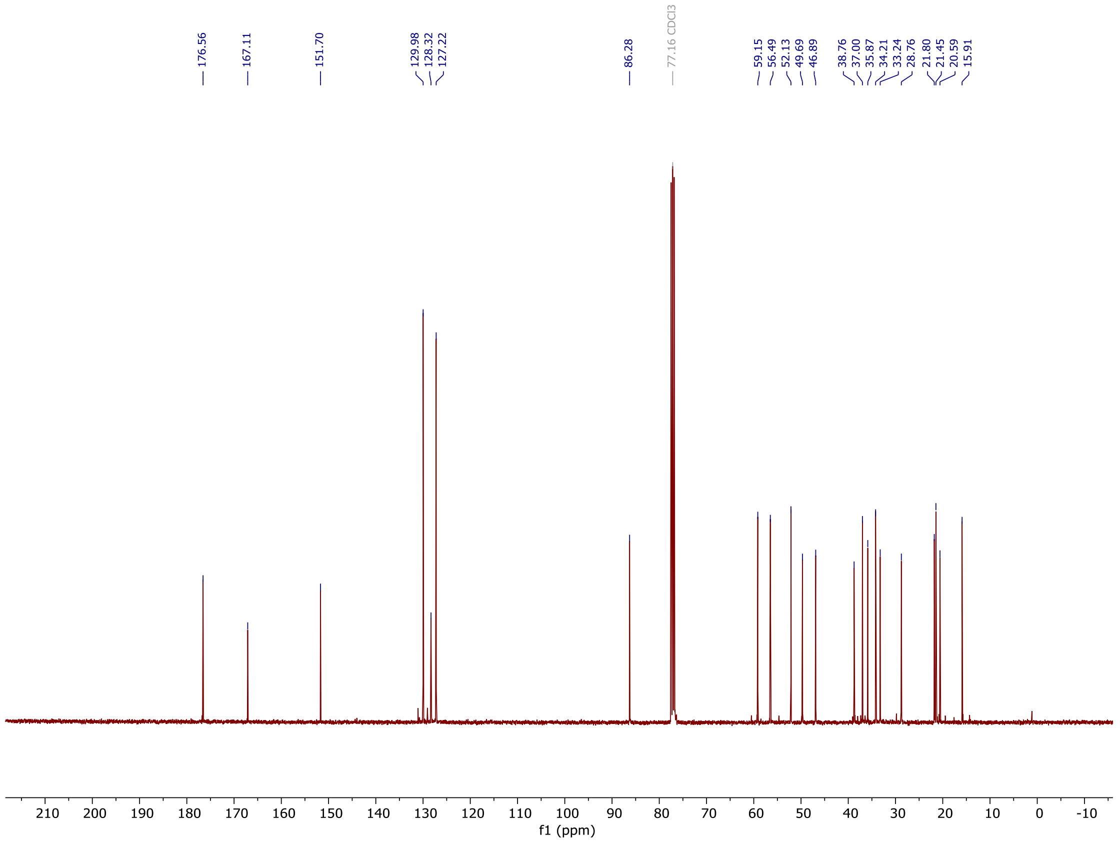

^{1}H NMR (400 MHz, $CDCl_3$)

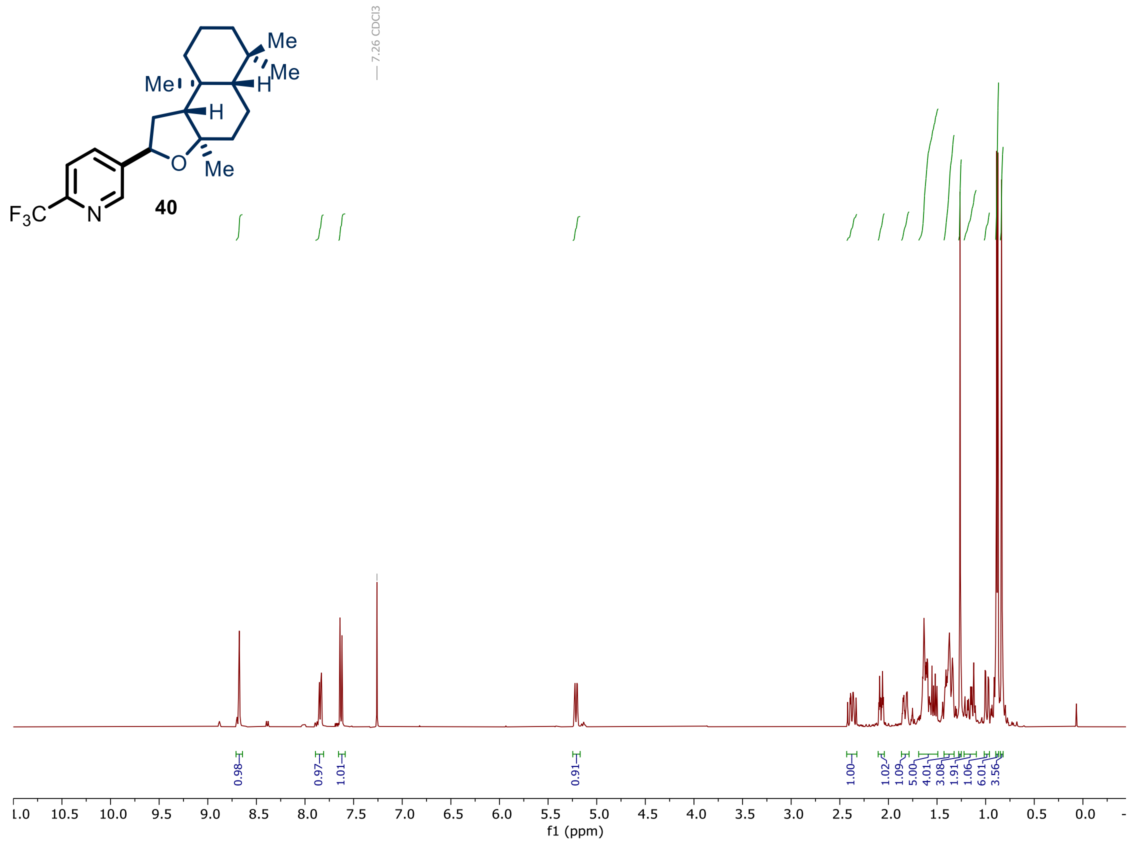

^{13}C NMR (101 MHz, $CDCl_3$)

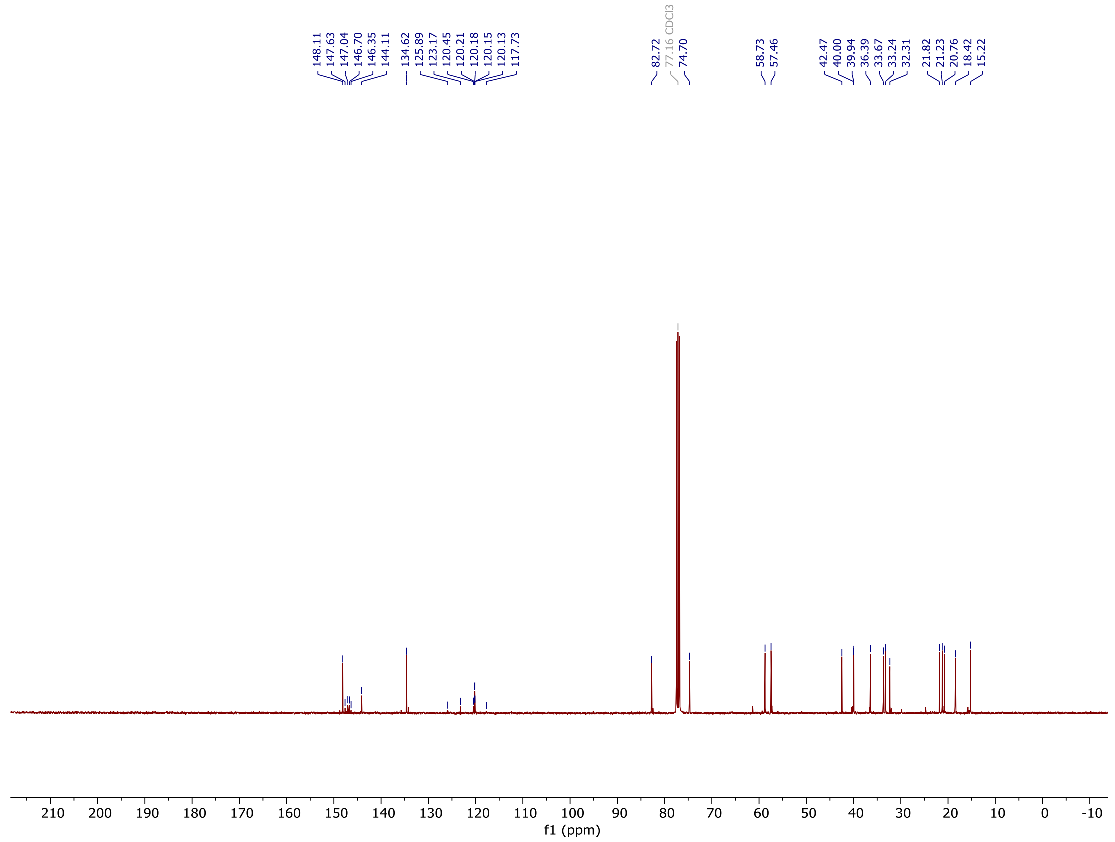

NOESY **40**

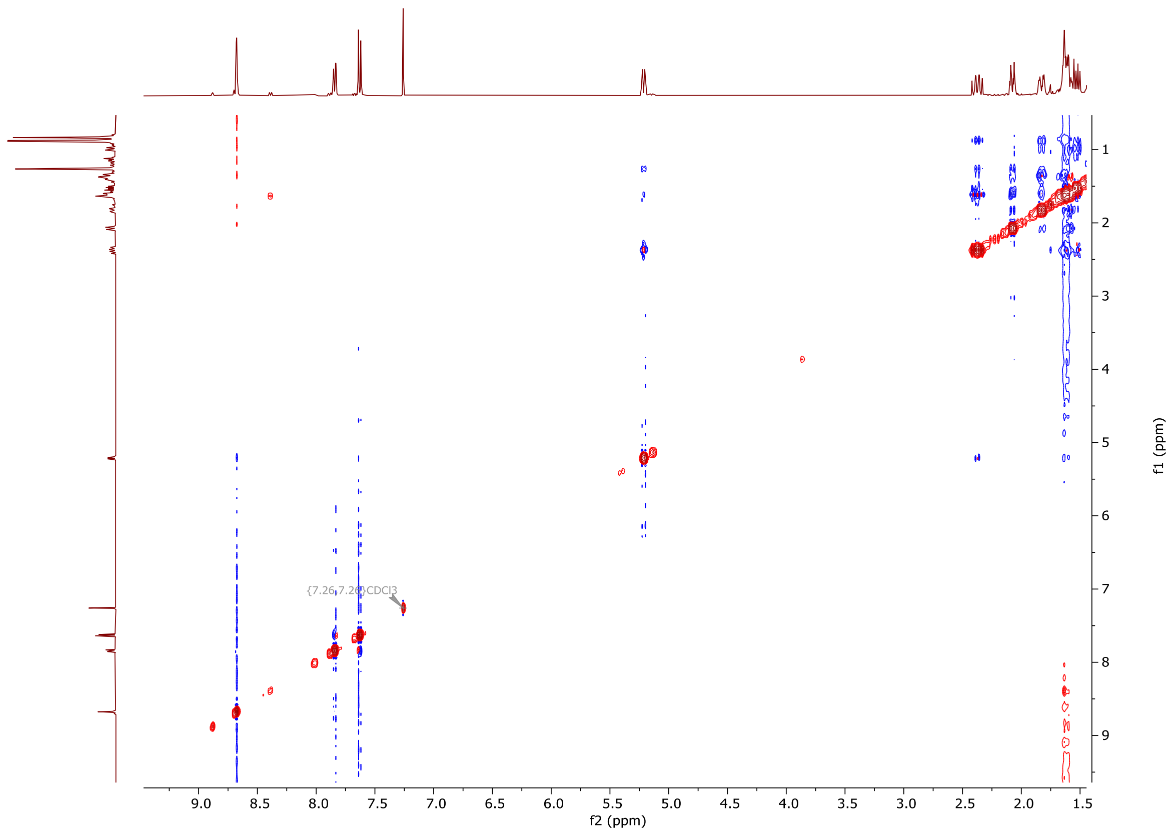

HSQC **40**

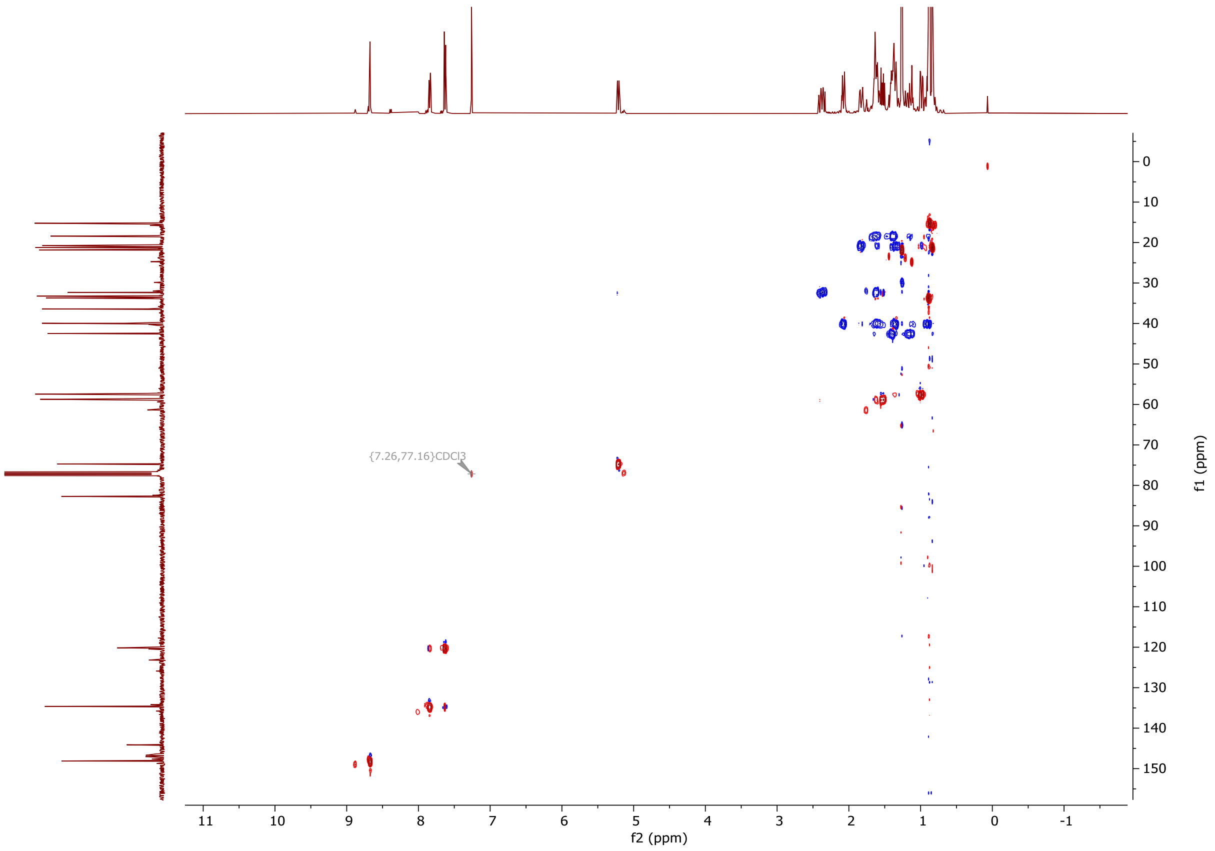

HMBC **40**

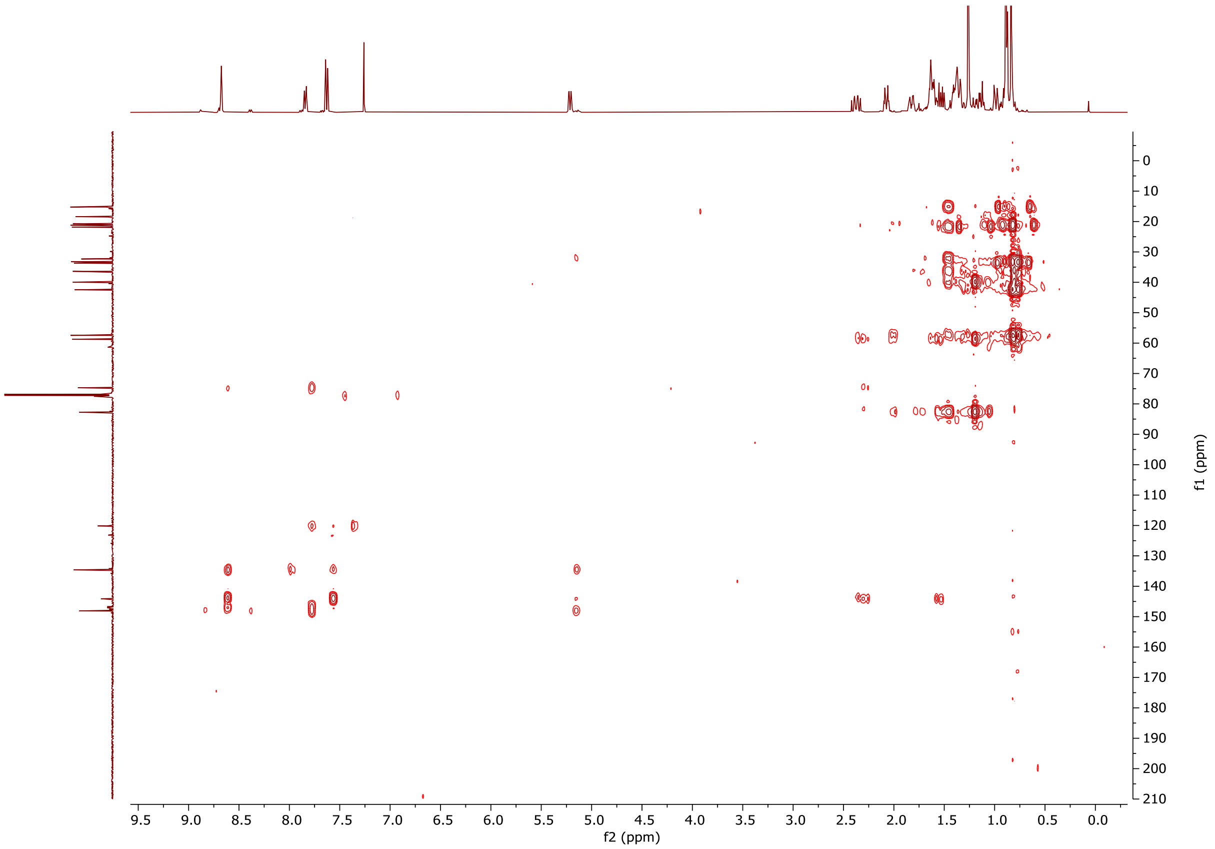

^{19}F NMR (376 MHz, $CDCl_3$)

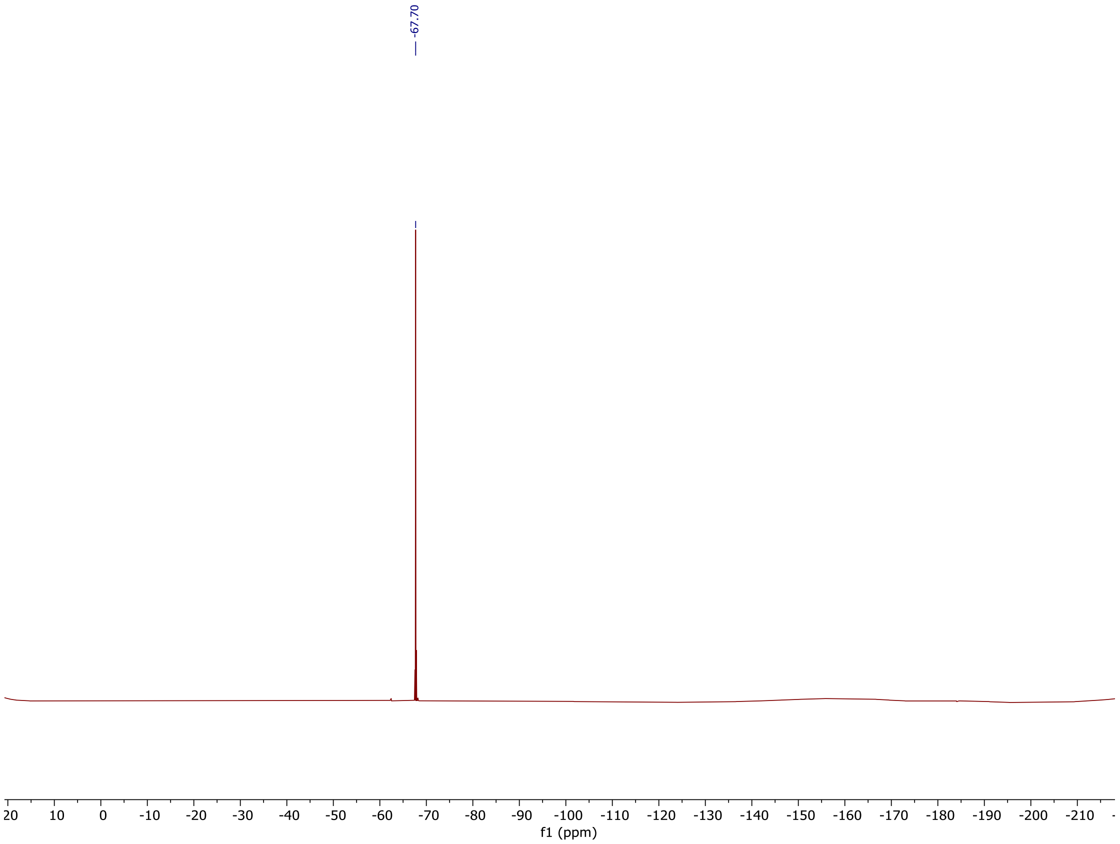

Supplement: Supplementary file 1 — Supporting Information [file ANIE-60-21277-s001.pdf]
